# New Charged Cholinesterase Inhibitors: Design, Synthesis, and Characterization

**DOI:** 10.3390/molecules29071622

**Published:** 2024-04-04

**Authors:** Milena Mlakić, Danijela Barić, Ana Ratković, Ivana Šagud, Ivona Čipor, Ivo Piantanida, Ilijana Odak, Irena Škorić

**Affiliations:** 1 Department of Organic Chemistry, Faculty of Chemical Engineering and Technology, University of Zagreb, Trg Marka Marulića 19, HR-10 000 Zagreb, Croatia; mdragojev@fkit.unizg.hr; 2 Group for Computational Life Sciences, Division of Physical Chemistry, Ruđer Bošković Institute, Bijenička Cesta 54, HR-10 000 Zagreb, Croatia; dbaric@irb.hr; 3 Chemistry, Selvita Ltd., Prilaz Baruna Filipovića 29, HR-10 000 Zagreb, Croatia; ana.ratkovic@selvita.com; 4 Croatian Agency for Medicinal Products and Medical Devices, Ksaverska Cesta 4, HR-10 000 Zagreb, Croatia; ivana.sagud@halmed.hr; 5 Division of Organic Chemistry and Biochemistry, Ruđer Bošković Institute, Bijenička Cesta 54, HR-10 000 Zagreb, Croatia; icipor@irb.hr (I.Č.); ivo.piantanida@irb.hr (I.P.); 6 Department of Chemistry, Faculty of Science and Education, University of Mostar, Matice Hrvatske bb, 88000 Mostar, Bosnia and Herzegovina

**Keywords:** cholinesterase inhibitors, BChE, AChE, synthesis, triazoles, triazolium salts, docking, genotoxicity

## Abstract

Triazoles and triazolium salts are very common subunits in the structures of various drugs. Medicaments with a characteristic 1,2,3-triazole core are also being developed to treat neurodegenerative disorders associated with cholinesterase enzyme activity. Several naphtho- and thienobenzo-triazoles from our previous research emerged as being particularly promising in that sense. For this reason, in this research, new naphtho- and thienobenzo-triazoles **23**–**34**, as well as 1,2,3-triazolium salts **44**–**51,** were synthesized and tested. Triazolium salts **44**–**46** showed excellent activity while salts **47** and **49** showed very good inhibition toward both butyrylcholinesterase (BChE) and acetylcholinesterase (AChE) enzymes. In contrast, neutral photoproducts were shown to be selective towards BChE but with very good inhibition potential as molecules **24**–**27**. The representative of newly prepared compounds, **45** and **50**, were stable in aqueous solution and revealed intriguing fluorimetric properties, characterized by a strong Stokes shift of >160 nm. Despite their condensed polycyclic structure shaped similarly to well-known DNA-intercalator ethidium bromide, the studied compounds did not show any interaction with ds-DNA, likely due to the unfavorable steric hindrance of substituents. However, the studied dyes bind proteins, particularly showing very diverse inhibition properties toward AChE and BChE. In contrast, neutral photoproducts were shown to be selective towards a certain enzyme but with moderate inhibition potential. The molecular docking of the best-performing candidates to cholinesterases’ active sites identified cation–π interactions as the most responsible for the stability of the enzyme–ligand complexes. As genotoxicity studies are crucial when developing new active substances and finished drug forms, in silico studies for all the compounds synthesized have been performed.

## 1. Introduction

DNA, RNA, and proteins are biomolecules that govern living organisms, are included in various biological processes, and are often the targets of novel drugs. Binding small molecules to these biomolecules can result in changes in biological properties, resulting in various biological activities [1]. Triazoles constitute an important class of heterocyclic molecules that exhibit a wide range of pharmacological activities [2]. A wide variety of drugs containing 1,2,3-triazole as a central heterocyclic structural component prove its pharmacological importance, such as anticonvulsants [3], antimalarials [4], antimicrobials [5,6], antivirals [7], antiproliferative drugs [8], antitumor drugs [9], analgesics [10], and antidiabetics [11]. This diversity of the triazole core has stimulated scientists’ interest in developing new triazole molecules with promising biological activities [12]. Drugs with a characteristic 1,2,3-triazole core are also being developed for the treatment of neurodegenerative diseases such as Alzheimer’s disease (AD) [13].

Positively charged 1,2,3-triazolium salts are also important bioactive scaffolds that have attracted special attention recently due to their specific properties compared to the uncharged 1,2,3-triazole unit [14]. 1,2,3-triazolium units have been proven to show a diversity of biological activities, which include antibacterial [15,16], antifungal [17], anticancer [18], and antileishmanial [19] properties. Many triazolium salts were highly potent in *Plasmodium falciparum* cultures [20], while some were used to stabilize gold nanoparticles [21]. The highly stable dye-sensitized solar cells based on 1,2,3-triazolium ionic liquids have also been also published [22], as well as the spectroscopic properties of 1,2,3-triazole BOPAHY dyes and their triazolium salts [23]. Additionally, they possess distinctive chemical properties, such as a high stability and adjustable reactivity [24]. Given the potentially broad application of 1,2,3-triazolium salts, there is a further need to find new biological applications for this charged heterocyclic subunit. 

**Figure 1:**
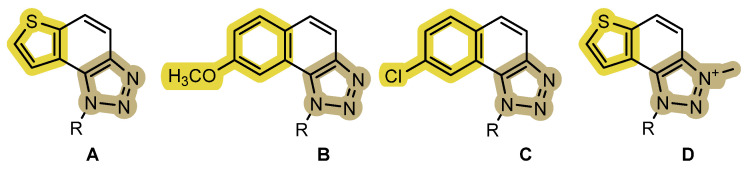
The basic skeleton of some previously synthesized thienobenzo- and naphtho-triazoles (structures **A** and **B**) and newly prepared uncharged and charged analogs in this work (structures **C** and **D**).

In the last few years, a broad spectrum of naphtho- and thienobenzo-triazoles has been prepared mostly photochemically (Figure 1, structures A and B) to analyze their potential inhibitory activity towards enzymes acetyl- (AChE) and butyrylcholinesterase (BChE). AChE has an important physiological role in the body because it controls the transmission of nerve impulses in cholinergic synapses of the nervous system by hydrolysis of the positively charged neurotransmitter acetylcholine [25]. BChE serves as a co-regulator of cholinergic neurotransmission and is able to catalyze the hydrolysis of acetylcholine, and high levels of BChE are associated with the neuropathological hallmarks of AD [26]. Several compounds from previous studies emerged as being particularly promising [27,28], and some displayed selectivity toward BChE. Based on these results, and taking into account the potential of 1,2,3-triazolium salts, a new series of naphthotriazoles (Figure 1, structure C) was synthesized in this work to examine the influence of the halogen as a substituent on the inhibitory potential towards cholinesterases. Additionally, for the first time, a series of charged thienobenzo-triazolium salts (Figure 1, structure D) was also prepared to see the effect of charge compared to their uncharged analogs (aromatic and non-aromatic) [29]. It was also challenging to see the influence of the position of the substituent on the aromatic ring on triazole, the influence of the nature of the substituent (OCH_3_ group in comparison to Cl), and the influence of the new dimethylamino group.

## 2. Results and Discussion

### 2.1. Synthesis of New 1,2,3-Triazolo-Stilbenes ***10**–**22***

**Scheme 1:**
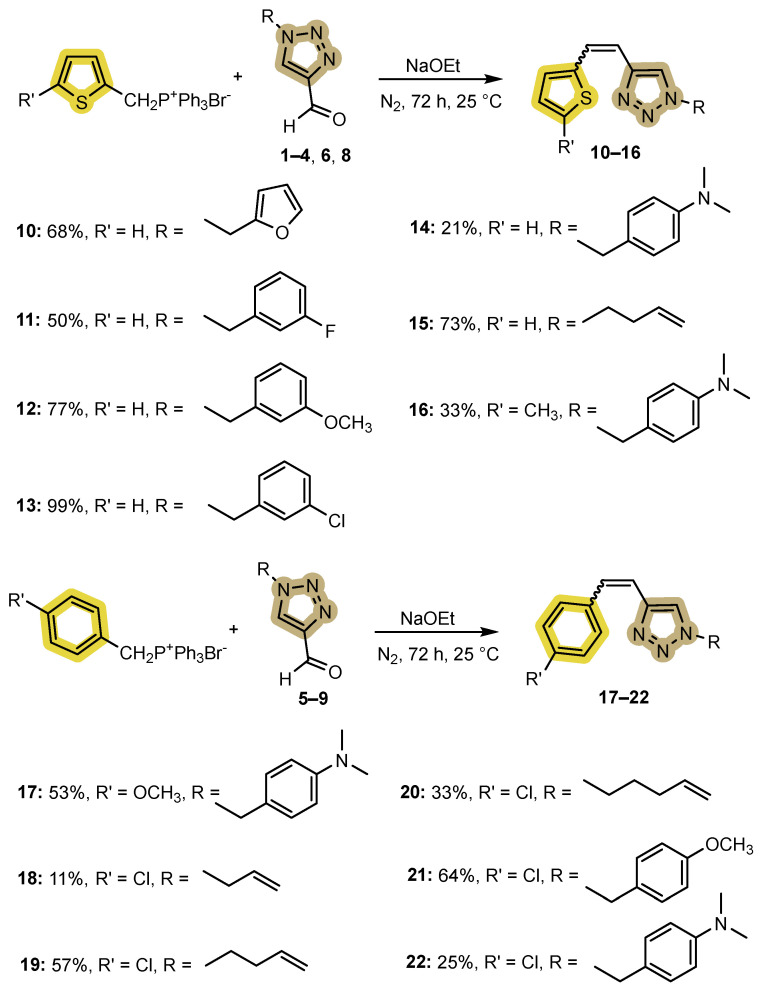
Synthetic route to new 1,2,3-triazolo-stilbenes **10**–**22** as mixtures of isomers. The indicated percentages represent isolated yields of mixtures of isomers.

The target compounds from the first step of the synthesis were 1,2,3-triazolostilbenes **10**–**22** as mixtures of isomers (Scheme 1), which were synthesized by the Wittig reaction of triphenylphosphonium salts with various 1-substituted 1,2,3-triazole-4-carbaldehydes **1**–**9**. For the synthesis of 1,2,3-triazolostilbene **10**–**22**, it was necessary to prepare the corresponding 1,2,3-triazole-4-carbaldehydes **1**–**9** by the reaction of 1-(4-nitrophenyl)-1*H*-1,2,3-triazole-4-carbaldehyde and the corresponding amine, according to the known procedure [30].

The Wittig reaction produced two configurational isomers, each of 1,2,3-triazolo-stilbenes **10**–**22**, with different ratios of *cis*- and *trans*-isomers depending on the substituents. The obtained mixtures of isomers were purified by extraction and column chromatography, where the petroleum ether/ether (PE/E) solvent system of variable proportions was used. In the resulting mixture of isomers, the *cis*-isomer eluted first, and the *trans*-isomer last at almost the same rate as the traces of the starting aldehyde. The residual *p*-nitroaniline was successfully separated if it was introduced into the reaction mixture with aldehyde during the synthesis, which is also where it is formed. For the synthesis of triazolostilbenes **10**–**16**, (thiophen-2-ylmethyl)triphenylphosphonium salt was used, while in the case of **17**–**22**, the (4-methoxybenzyl)- or (4-chlorobenzyl)triphenylphosphonium, salt, along the addition of base sodium ethoxide, was used.

### 2.2. Photocyclization of 1,2,3-Triazolo-Stilbenes ***10**–**22*** to the Targeted Final Photoproducts ***23**–**35***

**Scheme 2:**
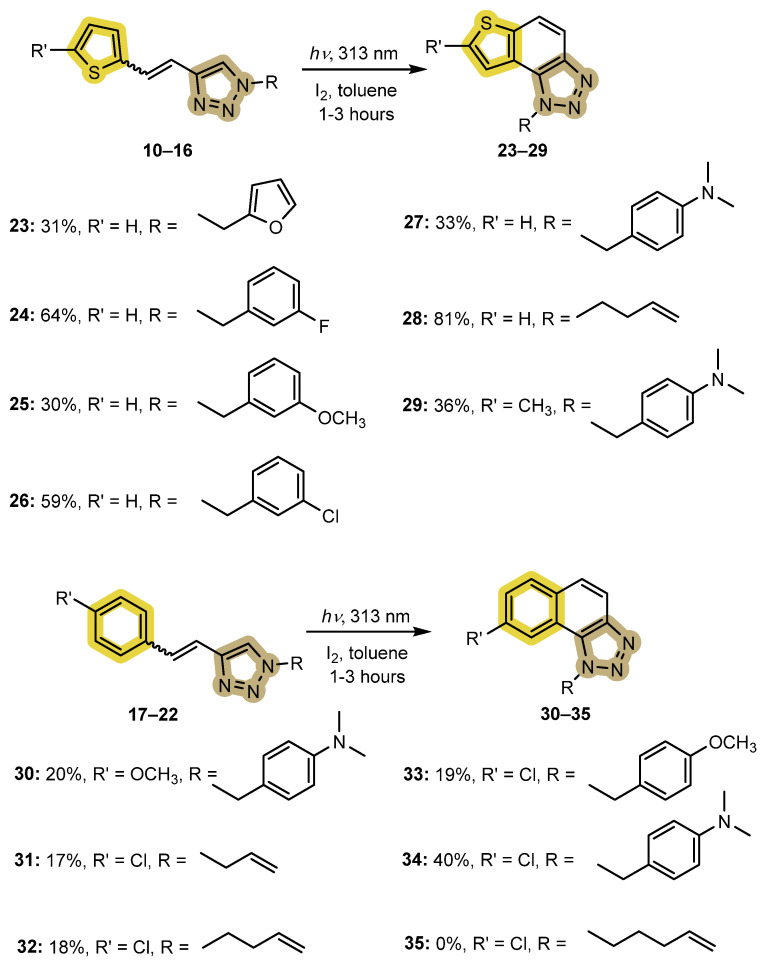
Photochemical cyclization of 1,2,3-triazolo-stilbenes **10**–**22** to the targeted thienobenzo- and naphtho-triazoles **23**–**35**. The indicated percentages represent isolated yields.

Thienobenzo- and naphthotriazoles **23**–**34** were synthesized by photochemical reactions of triazolostilbenes **10**–**21** (Scheme 2). Given that the wavelength ranges in which thienobenzo- and naphtho-triazoles absorb are known from previous research, a wavelength of 313 nm was used for photochemical cyclization reactions. The corresponding triazolostilbenes **10**–**22** were dissolved in toluene and transferred to a light-transmitting quartz tube. Iodine, which serves as an oxidizing agent, was added to the mixture. The course of the reaction was monitored using thin-layer chromatography in the PE/E solvent system. In the case of triazolo-stilbene **20**, naphthotriazole **35** was not formed.

### 2.3. Synthesis of the Triazolium Salts ***44**–**51***

**Scheme 3:**
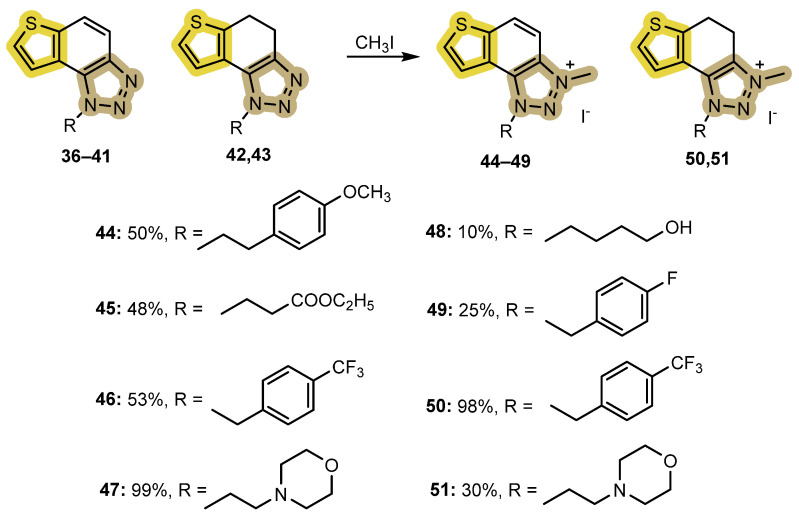
Preparation of charged triazoles **44**–**51** by methylation reaction from uncharged analogs. The indicated percentages represent isolated yields.

Thienobenzo-triazolium salts **44**–**51** were synthesized in dry dichloromethane using iodomethane from the previously published triazoles **36**–**43** [29] over a period of 24 h at 60 °C (Scheme 3). After the workup, the crude powder was dried under a high vacuum to afford the pure triazolium salts **44**–**51**.

All the synthesized 1,2,3-triazolo-stilbenes **10**–**22** and the targeted thienobenzo- and naphtho-triazoles **23**–**34,** as well as 1,2,3-triazolium salts **44**–**51**, have been fully proven by NMR, MS, and HRMS analyses (Figures S1–S109).

### 2.4. Spectroscopic Characterization in Biorelevant Medium and DNA-Binding Ability of Triazolium Salts

Prior to any biological studies, novel compounds should be evaluated for their stability in biorelevant conditions, whereby spectrophotometric characterization is the most common approach. Also, such characterization would often reveal beneficial properties of compounds, such as fluorescence, which could eventually be applied to studying interactions with biological targets.

**Figure 2:**
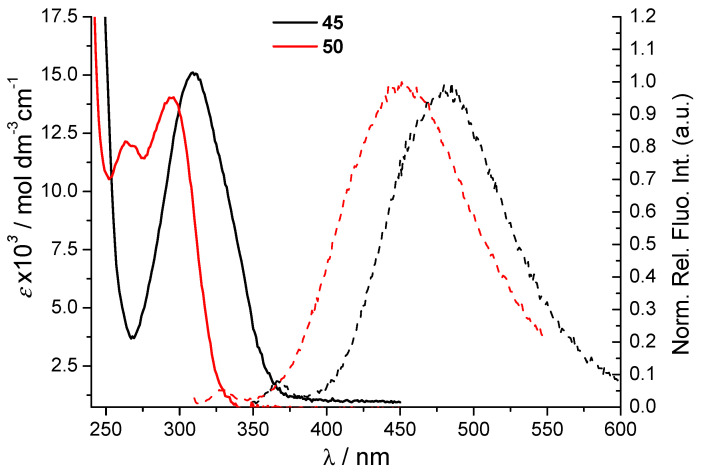
UV/Vis (solid) and emission (dashed) spectra of compounds **45** and **50** in sodium cacodylate buffer (pH = 7.0, *I* = 0.05 M).

The stock solutions of the compounds were prepared in DMSO at 1 mM concentration and further diluted in aqueous solutions prior to the experiment. The stock solutions were kept at +4 °C, and working aliquots were kept at +25 °C. No visible precipitation or change in the UV spectra of working solutions was noticed, pointing to the high stability of compounds. Experiments were further conducted in a biologically relevant aqueous medium at pH = 7.0 (sodium cacodylate buffer, *I* = 0.05 M). Both compounds show adherence to the Beer–Lambert law to up to 10 μM concentrations (Figures S110 and S111), excluding aggregation in water. The difference in the UV/Vis spectra (Figure 2, Table 1) agrees well with the structures of the compounds. Namely, the absorption maximum of condensed aromatic compound **45** is bathochromically shifted with respect to compound **50**; the latter shows separate absorption maxima that could be related to two moieties, the thieno-benzo core and the CF_3_-substituted benzyl moiety. Both studied compounds exhibit fluorescence, the condensed aromatic analog **45** showing a bathochromically shifted emission maximum with respect to **50**, but **50** has a higher fluorescence intensity (Figure 2, Figures S112 and S113, Table 1). The emission intensity of **45** and **50** is proportional to the concentration at up to 2 μM concentrations (Figures S112 and S113). The excitation maxima correlate with the absorption maxima.

Emission was stable upon heating and cooling the samples (Figure S114). Both compounds exhibit a large Stokes shift (Table 1, Figure S115), which is generally favorable for fluorescent dyes, excluding self-absorption.

Another feasible spectroscopic property is solvatochromism, which can be employed in water-exclusion sensing [31,32] or lipid-membrane studies [33]. UV/Vis and fluorescence spectra were also taken in 1-octanol as a referent nonpolar solvent to evaluate potential solvatochromic effects. For both **45** and **50**, emission in 1-octanol was hypsochromically shifted for 20 nm (Figures S116–S118); such a minor change is not applicable for fine water-content sensing.

Compounds **44**–**51** are cationic polycyclic structures, and particularly **44**–**49** are also condensed heteroaromatics rich in nitrogen atoms, with a crescent shape very similar to ethidium bromide, thus pointing toward possible interactions with ds-DNA [34]. Therefore, we have chosen two triazolium salts, condensed aromatic **45** and its non-condensed analog **50**, to study their DNA-binding ability.

To preliminarily probe the binding ability of **45** and **50** to ds-DNA, we used calf thymus ct-DNA as a representative of the B-helical secondary structure and containing equal amounts of AT- and GC-base pairs. The additions of ct-DNA to aqueous solutions of **45** and **50** at up to 50-fold excess did not yield any change in the UV/Vis spectra of compound **45,** as well as yielding no change in the emission spectra of both compounds (Figures S119–S121), thus indicating no bio-relevant affinity of the dyes towards ds-DNA. Further, employing the fluorescence of the compounds, we performed fluorimetric titrations of the compounds with either bovine serum albumin (BSA) or human serum albumin (HSA) as common carrier proteins included in the transfer of numerous small molecules [35]. The results showed an indication of binding; however, at given experimental conditions, the autofluorescence of the proteins hampered the accurate determination of binding constants.

The studied compounds did not show biorelevant activity toward ds-DNA, thus avoiding possible genotoxicity; however, they did show binding to serum albumin protein, which strongly supported further studies with relevant protein targets. As noted in the Introduction, similar analogs showed intriguing activity against enzymes AChE and BChE; thus, we studied this interaction in all compounds in detail.

### 2.5. ChEs Inhibition

Triazoles **23**–**34** and **44**–**51** were tested for their ability to inhibit acetylcholinesterase and butyrylcholinesterase, applying Ellman’s assay [36] with slight modifications, using galantamine as a standard. From the results shown in Table 2 and considering our previous findings on structurally similar compounds, the following structure–activity relationship can be noted.

Newly synthesized thienobenzo-triazole derivatives **23**–**34** effectively inhibited BChE with very good to moderate IC_50_ values (Table 2). Very good inhibitory activity was observed for **24**, **25**, **26**, and **27**, with IC_50_ values three−four times lower than the IC_50_ of standard galantamine. These thienobenzo-triazoles possess the following substituents at the triazole unit: 3-fluorobenzyl, 3-methoxybenzyl, 3-chlorobenzyl, and 4-dimethylaminobenzyl. Derivative **24**, with a fluorine substituent in the *meta* position, was the only one that reached the IC_50_ value for AChE. In the previously tested series of thienobenzo-triazoles [28], the derivative with a fluorine substituent in the *para* position reached IC_50_ values for both enzymes with the same concentration value. The derivative **25** with the methoxy group in the *meta* position also has a pair of analogs in the previous series [28] with the *para* position of the substituent. Both derivatives, **24** and **25**, inhibit BChE in a similar concentration value, and in comparison with their analogs from [28], this leads to the conclusion that the position of the substituent on the aromatic ring does not significantly change the inhibitory activity. The introduction of a new substituent, 4-dimethylamino benzyl present in **27**, led to a very good inhibition of BChE. A comparison of the derivatives with and without a methyl substituent at the thiophene ring is possible, too. The methyl-substituted thiophene analog of **23** was tested earlier [26], while **27** and **29** tested here represent such analogs too. The introduction of a methyl group at the thiophene reduced affinity toward enzyme BChE.

Another group of triazole derivatives, naphtotriazoles **30**–**34**, showed weak potency toward the inhibition of the cholinesterases. None of the tested compounds within this group achieved significant results. Only derivative **32** with a buthenyl substituent at the triazole ring inhibited both enzymes, with a weak IC_50_ value for AChE, lower by three orders of magnitude than for the standard galantamine, and a good value for BChE, an order of magnitude below the value measured for the standard. Derivatives **31**–**34** with the chlorine substituent on the naphtho moiety have methoxy-substituted analogs with the same substituent at the triazole ring (compound **30** and the compound previously tested in [28]). The replacement of the methoxy group with the chlorine decreased affinity towards enzymes.

**Figure 3:**
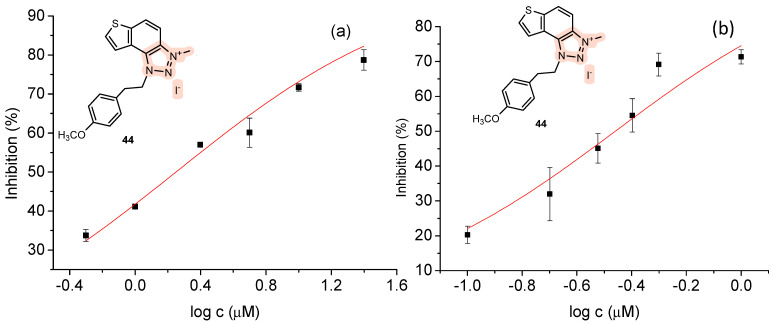
Dose–response curve for the inhibition of AChE (**a**) and BChE (**b**) by **44**.

**Figure 4:**
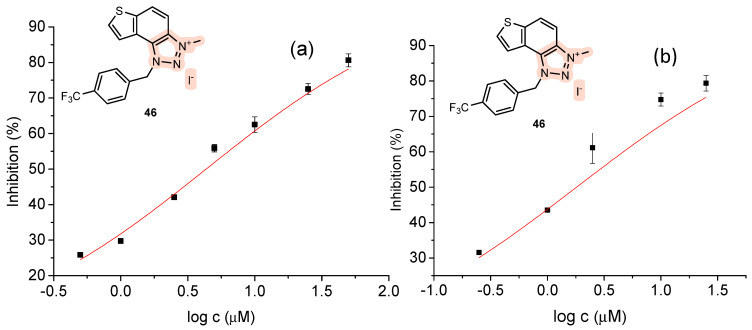
Dose–response curve for the inhibition AChE (**a**) and BChE (**b**) by via **46**.

Both AChE and BChE were effectively inhibited by the last studied group, the 1,2,3-triazolium salts **44**–**51** (Table 2, Figure 3 and Figure 4). The most potent inhibitory effects were observed for **44**, **45**, **46**, **47**, and **49**, with IC_50_ values better or similar to the standard galantamine for BChE and somewhat weaker for AChE. These derivatives can be compared to the uncharged analogs studied earlier [26]—the analog of the most effective salt **44** was highly selective, but an excellent inhibitor of BChE with an IC_50_ value and order of magnitude better than for the standard galantamine. A similar observation also applies to the uncharged analog of **45**: transformation to the salt form retains successful inhibition toward BChE, but activity toward AChE is drastically increased. Salts **46** and **49** increased the activity toward both enzymes compared to their uncharged analogs studied earlier. Regarding the type of the central ring, whether aromatic or non-aromatic, **46** had better IC_50_ values than the non-aromatized analog **50**, while **47** and its non-aromatized analog **51** have almost the same affinities for both enzymes. Somewhat weaker inhibition values were achieved with **48**, so of all the substituents, 4-hydroxybutyl proved to be the one that reduces activity most. Therefore, the type of substituent and charge at the nitrogen of the triazole ring are critical structural features that govern inhibitory potency, albeit with the loss of selectivity.

**Figure 5:**
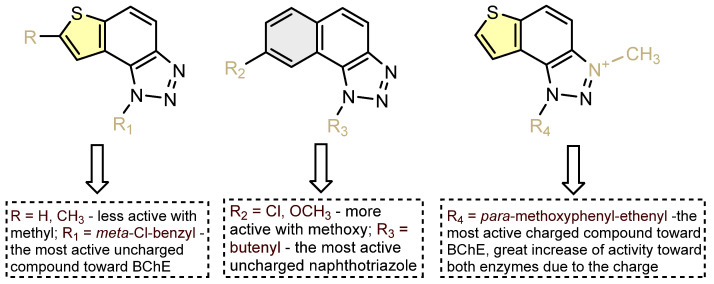
SAR of triazole fragment-based library concerning their cholinesterase-inhibition activity.

The obtained results were summarized in a structure–activity relationship overview (Figure 5). The key determinants in a structure–activity relationship are the type of (hetero)cyclic ring, the molecule’s geometry, the type of substituent, and the charge on the triazole ring.

### 2.6. Molecular Docking into Cholinesterases

**Figure 6:**
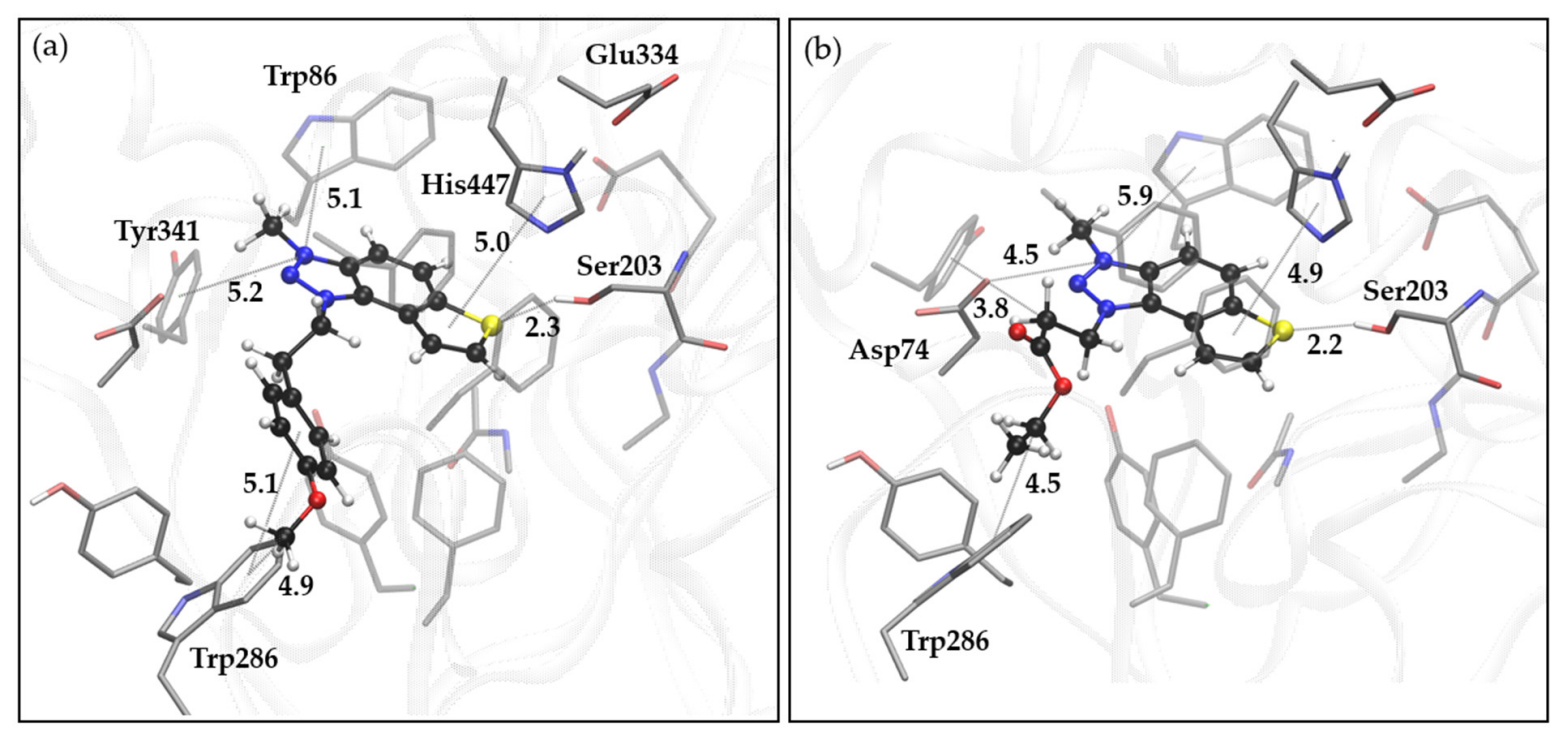
(**a**) The structure of the AChE’s active site docked with **44** and (**b**) docked with **45**. Distances given in angstroms; hydrogen atoms of the residues omitted for clarity.

According to experimental results, two charged triazole derivatives, compounds **44** and **45**, showed excellent inhibitory potential toward both cholinesterases. Molecular docking was performed to obtain insight into the binding modes of these ligands within the active site and identify interactions responsible for the stability of the enzyme–ligand complex. Figure 6 shows the structures of the most stable complexes formed between triazolium ions **44** and **45**, respectively, and the active site of AChE obtained by docking.

Both ligands have similar orientations, with the main molecular framework comprising thiophene, phenyl, and triazole rings positioned within a 5 Å distance from the esteratic active site (Ser203, Glu343, and His438). The sulfur from thiophene is positioned in proximity to the serine hydroxyl group, forming a weak hydrogen bond, and there is a π-π stacking between thiophene and His438. In both complexes, an energetically significant cation–π interaction is observed between the methyl-substituted triazole nitrogen and residue Trp86 of the anionic subsite.

**Figure 7:**
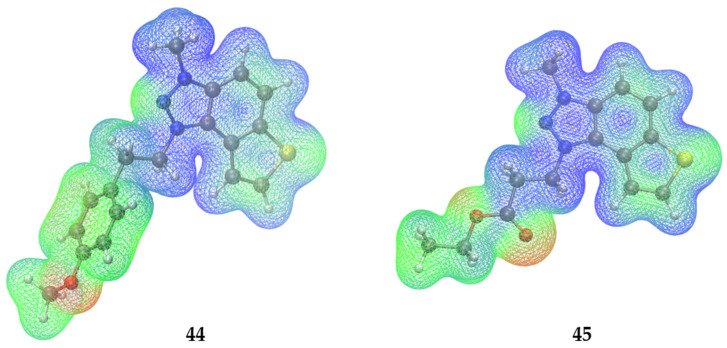
The electrostatic potential maps of triazolium cations of compounds **44** and **45**. Electrostatic surface potentials are colored blue and red for positive and negative charges, respectively, and green represents neutral regions.

The calculated electrostatic potential maps, illustrating the charge distribution within the cations of compounds **44** and **45** (Figure 7), indicate that the triazole ring is the most positively charged region of the scaffold; therefore, the triazole involvement in cation–π interactions is expected. In the AChE complex with **44** (Figure 6a), a cation–π interaction is also observed between the triazolium nitrogen and residue Trp341 belonging to the peripheral anionic site (PAS). However, in the complex involving compound **45** (Figure 6b), the Tyr341 participates in the alkyl–π interaction with the ethyl chain that connects triazole and the *p*-methoxyphenyl, while the positive triazolium nitrogen establishes an electrostatic attraction with Asp74, which is also part of the PAS. Positioning the *p*-methoxyphenyl substituent of compound **44** within the complex enables the alkyl–π interaction with residue Trp286, similar to the situation observed in the complex with **45**, where the terminal ethyl group of the substituent interacts with Trp286.

**Figure 8:**
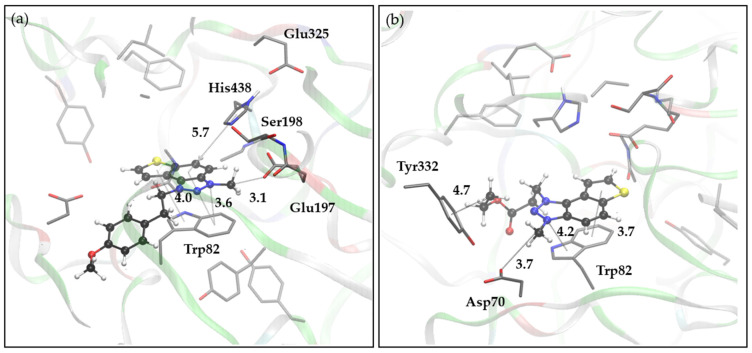
(**a**) The structure of the BChE’s active site docked with **44** and (**b**) docked with **45**. Distances given in angstroms; hydrogen atoms of the residues omitted for clarity.

The docking of cations **44** and **45** into the active site of BChE yielded structures depicted in Figure 8. Notably, the main scaffolds did not adopt mutually similar orientations. In the complex formed between **44** and BChE, a cation–π interaction is evident between the triazolium nitrogen and Trp82 of the anionic site. Simultaneously, there is an electrostatic attraction between the methyl group at triazolium and Glu197, a residue also belonging to the anionic sub-domain (Figure 8a). While the scaffold of **45** is situated between Trp82 and the esteratic site, akin to the complex with **44**, its orientation allows for the electrostatic attraction between the most positively charged part of the triazolium and residue Asp70, belonging to the PAS (Figure 8b). Additional stabilizing factors within these complexes involve the parallel π-π stacking between the thiophene and Trp82 in the complex with **44**, whereas the complex with **45** features an alkyl–π interaction between the terminal ethyl group of the substituent and residue Tyr332.

### 2.7. Genotoxicity

Impurities that can be present in the active pharmaceutical ingredient (API) as well as the related finished drug product cannot be completely avoided and an investigation into these compounds is a pivotal part of drug product development and is of critical importance for drug safety. Impurities that can be present in the active substance, as well as in each intermediate during the manufacturing process, have to be evaluated in regard to their genotoxic potential properties. Compounds with proven or potential genotoxic properties will be more strictly regulated and have to be controlled at very low levels, significantly lower than other impurities (ICH M7 Guideline). These levels that are then allowed to be present in the drug substance/product will be calculated based on their experimental acceptable daily intake (AI) and the maximum daily dose (MDD) of the drug. With new compounds, the AI is usually not yet determined by toxicological studies on animals, and then the most conservative approach has to be taken with the strictest presumed AI as described in the guideline itself. Evaluations are primarily performed consistently by the use of in silico Q(SAR) tools. When developing new active substances and finished drug forms, these impurities will be new compounds, and usually no experimental data will be available. In these cases, the Q(SAR) approach is of vital importance. (Q)SAR models make predictions of biological activity based on structural components [37]. This approach is vital during the early stages of searching for potentially active drug substances. Eliminating all compounds with mutagenic potential saves a lot of money and time. The Lhasa software package (Nexus v.2.5.2 (Build 5, Jul 2022), Derek Nexus v.6.2.1 and Sarah Nexus v.3.2.1) is the most commonly used software because it has two complementary models; it is also very important that all predictions are again reviewed an toxicology expert.

In the case of compounds **44**–**51**, no structural alerts were found by Derek Nexus (Table 3). For Sarah, these compounds are out of scope, as will be the case often with very new synthetic compounds that are only starting to be investigated for their biological activity. This is an excellent example of how a complimentary model is a must, especially in this early development. With compound **10**, Sarah Nexus has found a similar training set, and this compound can be negative. These compounds would be considered the safest ones for the continuation of the early stages of development. As for the compounds **27**, **31**, **34**, and **30** that have the dimethyl-amino-aryl moiety and compounds **33** and **24**–**26** substituted halogen-aryl and methoxy-aryl moieties, the positive hypothesis provided by Sarah cannot be overruled by the negative by Derek, so they would probably be investigated further with utmost scrutiny and only if there were no other better active ingredients as new leads. These compounds would require an experimental AMES test to be conducted to determine their genotoxic potential.

## 3. Materials and Methods

### 3.1. General Procedure

600 and 300 MHz Bruker Avance spectrometers were used to record ^1^H and ^13^C NMR spectra. Compounds analyzed by NMR techniques were dissolved in CDCl_3_. As a standard, tetramethylsilane (TMS) was used. Chemical shifts are expressed in ppm (parts per million) units. The following signals in the ^1^H NMR spectra were specific and did not correspond to compounds: the signal for water in chloroform at about 1.50 ppm, the signal for chloroform at 7.24 ppm, the signal for dichloromethane in chloroform at 5.26 ppm, and the signal for acetone in chloroform at 2.17 ppm. Each ^13^C NMR spectrum contained one specific signal (group of three peak lines) at 77 ppm corresponding to the used solvent—deuterated chloroform. High-resolution mass spectrometry (HRMS) analyses were performed on a MALDI TOF/TOF analyzer mass spectrometer fitted with an Nd:YAG laser at 355 nm (fitting rate of 200 Hz). Photochemical reactions were carried out in a 50.0 mL solution in quartz cuvettes that transmitted light. For this purpose, a Rayonet photochemical reactor equipped with UV lamps (10) with a wavelength of 313 nm was used. All solvents used in this work were purified by distillation and were commercially available. The phosphonium salts and 1-(4-nitrophenyl)-1*H*-1,2,3-triazole-4-carbaldehyde [29] used were previously synthesized in our laboratory. After each Wittig reaction, an extraction was performed thrice, separating the organic and aqueous layers. The organic layer was dried over anhydrous magnesium sulfate, MgSO_4_. Thin-layer chromatography was performed on plates coated with silica gel (0.2 mm, 60/Kieselguhr F_254_) immersed in 10 mL of the dissolution system. Column chromatography was performed in glass columns of different diameters. The columns were filled with silica gel (60 Å, technical grade) of different heights. Abbreviations used in the experimental part of the work are the following: ACN—acetonitrile, DCM—dichloromethane, E—diethyl ether, EtOAc—ethyl acetate, PE—petroleum ether, NMR—nuclear magnetic resonance, UV—ultraviolet spectroscopy, NaOEt—sodium ethoxide, s—singlet, d—doublet, t—triplet, m—multiplet, dd—doublet of doublets, and q—quartet. Spectrophotometric measurements were performed in aqueous buffer solution (sodium cacodylate buffer, pH = 7.0, *I* = 0.05 M). UV/Vis spectra were recorded on a Varian Cary 100 Bio spectrometer, and fluorescence spectra on a Varian Cary Eclipse fluorimeter in appropriate quartz cuvettes (path length = 1 cm). Spectroscopic data were analyzed by Origin 7.0. Polynucleotide ct-DNA (*calf thymus* DNA, Sigma Aldrich, St. Louis, MO, USA) was dissolved in sodium cacodylate buffer (pH = 7.0, *I* = 0.05M), sonicated, and filtered through a 0.45 mm filter to obtain short rod-like B-helical DNA fragments [38]. The concentration of ct-DNA was determined as the concentration of phosphates (corresponding to c(nucleobase)) spectroscopically at *λ*_max_ = 260 nm [39]. Bovine serum albumin (BSA, Sigma Aldrich) and human serum albumin (HSA, Sigma Aldrich) were dissolved in MilliQ water at *c* = 1 mM.

### 3.2. Synthesis of Triazole Aldehydes ***1**–**9***

Triazole aldehydes **1**–**9** were synthesized in small glass vials from 1-(4-nitrophenyl)-1*H*-1,2,3-triazole-4-carbaldehyde dissolved in dry 1,4-dioxane. The appropriate amine was then added to the reaction mixture, and the mixture was briefly purged with argon. Depending on the applied amine, the reaction took place for a certain period of time (most often around 24 h). Care must be taken in the reaction time to avoid amine polymerization. The course of the reaction was monitored by thin-layer chromatography. At the end of the reaction, the solvent was removed by evaporation on a rotary vacuum evaporator. The solid product was purified by column chromatography with an appropriate solvent system.

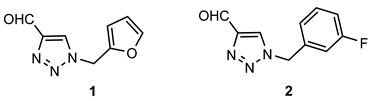



Compound **1** was synthesized according to the general procedure [30] from 2-furfurylamine (106.2 mg or 97.2 μL, 1.10 mmol) and 1-(4-nitrophenyl)-1*H*-1,2,3-triazole-4-carbaldehyde (200.0 mg, 0.92 mmol) dissolved in 2 mL of 1,4-dioxane. The obtained product was purified by column chromatography (*ϕ* = 3 cm; *h* = 13 cm; system PE/DCM and DCM/EtOAc (2%)).

1-(furan-2-ylmethyl)-1*H*-1,2,3-triazol-4-carbaldehyde (**1**): 99.6 mg, 61% of the isolated yield; yellow oil; *R_f_* (DCM) = 0.23; ^1^H NMR (CDCl_3_, 600 MHz) *δ*/ppm: 10.13 (s, 1H), 8.10 (s, 1H), 7.46 (d, *J* = 1.9 Hz, 1H), 6.53 (d, *J* = 3.3 Hz, 1H), 6.42 (dd, *J* = 3.3, 1.9 Hz, 1H), 5.61 (s, 2H).

Aldehyde **2** was synthesized from 3-fluorobenzylamine (137.8 mg or 125.3 μL, 1.10 mmol) and 1-(4-nitrophenyl)-1*H*-1,2,3-triazole-4-carbaldehyde (200.0 mg, 0.92 mmol) dissolved in 2 mL of 1,4-dioxane. The obtained product was purified by column chromatography (*ϕ* = 1.5 cm; *h* = 20 cm; PE/DCM system and pure DCM).

1-(3-fluorobenzyl)-1*H*-1,2,3-triazole-4-carbaldehyde (**2**): 143.6 mg, 76% of the isolated yield; yellow oil; *R_f_* (DCM) = 0.22; ^1^H NMR (CDCl_3_, 600 MHz) *δ*/ppm: 10.14 (s, 1H), 8.03 (s, 1H), 7.41–7.34 (m, 1H), 7.11–7.07 (m, 2H), 7.00 (d, *J* = 9.1 Hz, 1H), 5.59 (s, 2H).

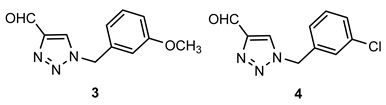

Compound **3** was synthesized from 3-methoxybenzylamine (151.0 mg or 141.1 μL, 1.10 mmol) and 1-(4-nitrophenyl)-1*H*-1,2,3-triazole-4-carbaldehyde (200.0 mg, 0.92 mmol) dissolved in 2 mL of 1,4-dioxane. The obtained product was purified by column chromatography (*ϕ* = 1.5 cm; *h* = 16.5 cm; system PE/DCM and DCM/EtOAc (2%)).

1-(3-methoxybenzyl)-1*H*-1,2,3-triazole-4-carbaldehyde (**3**): 83.0 mg, 42% of the isolated yield; yellow oil; *R_f_* (DCM) = 0.19; ^1^H NMR (CDCl_3_, 600 MHz) *δ*/ppm: 10.13 (s, 1H), 8.00 (s, 1H), 7.32 (t, *J* = 8.1 Hz, 1H), 6.92 (d, *J* = 8.5 Hz, 1H), 6.88 (d, *J* = 7.8 Hz, 1H), 6.82 (s, 1H), 5.55 (s, 2H), 3.80 (s, 1H).

Aldehyde **4** was synthesized from 3-chlorobenzylamine (155.9 mg or 129.9 μL, 1.10 mmol) and 1-(4-nitrophenyl)-1*H*-1,2,3-triazole-4-carbaldehyde (200.0 mg, 0.92 mmol) dissolved in 2 mL of 1,4-dioxane. The obtained product was purified by column chromatography (*ϕ* = 2 cm; *h* = 14 cm; PE/DCM system and pure DCM).

1-(3-chlorobenzyl)-1*H*-1,2,3-triazole-4-carbaldehyde (**4**): 145.1 mg, 71% of the isolated yield; yellow oil; *R_f_* (DCM) = 0.16; 1H NMR (CDCl_3_, 600 MHz) *δ*/ppm: 10.14 (s, 1H), 8.04 (s, 1H), 7.38 (d, *J* = 8.2 Hz, 1H), 7.35 (t, *J* = 7.9 Hz, 1H), 7.30 (s, 1H), 7.19 (d, *J* = 7, 3 Hz, 1H), 5.57 (s, 2H).

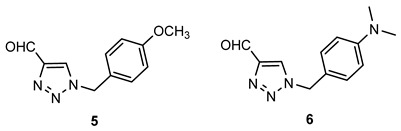

Compound **5** [29] was synthesized according to the general procedure from (4-methoxyphenyl)methanamine (133 mg or 143 μL, 0.99 mmol) and 1-(4-nitrophenyl)-1*H*-1,2, 3-triazole-4-carbaldehyde (180, mg, 0.83 mmol) dissolved in 2 mL of 1,4-dioxane. The obtained product was purified by column chromatography (*ϕ* = 3 cm; *h* = 13 cm; system PE/DCM and DCM/EtOAc (2%)).

1-(4-methoxybenzyl)-1*H*-1,2,3-triazole-4-carbaldehyde (**5**): 70 mg, 36% of isolated yield; yellow oil; *R_f_* (DCM/EtOAc (2%)) = 0.33; ^1^H NMR (CDCl_3_, 300 MHz) *δ*/ppm: 10.10 (s, 1H), 8.08 (s, 1H), 7.26 (d, *J* = 8.6 Hz, 2H), 6.91 (d, *J* = 8.7 Hz, 2H), 5.52 (s, 2H), 3.81 (s, 3H).

Aldehyde **6** was synthesized from 4-(aminomethyl)-*N*,*N*-dimethylaniline (148.8 mg or 114.0 μL, 0.99 mmol) and 1-(4-nitrophenyl)-1*H*-1,2,3-triazole-4-carbaldehyde (180, mg, 0.83 mmol) dissolved in 2 mL of 1,4-dioxane. The obtained product was purified by column chromatography (*ϕ* = 3 cm; *h* = 13 cm; system PE/DCM and DCM/EtOAc (2%)).

1-(4-(dimethylamino)benzyl)-1*H*-1,2,3-triazole-4-carbaldehyde (**6**): 139.4 mg, 73% of the isolated yield; yellow oil; *R_f_* (DCM) = 0.11; ^1^H NMR (CDCl_3_, 600 MHz) *δ*/ppm: 10.10 (s, 1H), 7.91 (s, 1H), 7.19 (d, *J* = 7.2 Hz, 2H), 6.70 (d, *J* = 6.7 Hz, 2H), 5.46 (s, 2H), 2.97 (s, 6H).

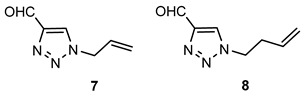

Compound **7** [29] was synthesized from (*E*)-prop-1-en-1-amine (62.82 mg or 82.3 μL, 0.99 mmol) and 1-(4-nitrophenyl)-1*H*-1,2,3-triazol-4-carbaldehyde (180, mg, 0.83 mmol) dissolved in 2 mL of 1,4-dioxane. The obtained product was purified by column chromatography (*ϕ* = 2 cm; *h* = 17 cm; system PE/DCM and DCM/EtOAc (2%)).

1-allyl-1*H*-1,2,3-triazole-4-carbaldehyde (**7**): 80 mg, 70% of the isolated yield; yellow oil; *R_f_* (DCM/EtOAc (2%)) = 0.47; ^1^H NMR (CDCl_3_, 300 MHz) *δ*/ppm: 10.14 (s, 1H), 6.10 –6.01 (m, 1H), 5.45 (d, *J* = 10.3 Hz, 1H), 5.37 (d, *J* = 17.9 Hz, 1H), 5.07 (d, *J* = 6.2 Hz, 2H).

Aldehyde **8** [29] was synthesized from 3-buten-1-amine (78.26 mg or 101 μL, 0.99 mmol) and 1-(4-nitrophenyl)-1*H*-1,2,3-triazol-4-carbaldehyde (180, mg, 0.83 mmol) dissolved in 2 mL of 1,4-dioxane. The obtained product was purified by column chromatography (*ϕ* = 2 cm; *h* = 17 cm; system PE/DCM and DCM/EtOAc (2%)).

1-(but-3-en-1-yl)-1*H*-1,2,3-triazol-4-carbaldehyde (**8**): 72.5 mg, 58% of the isolated yield; yellow oil; *R_f_* (DCM) = 0.35; ^1^H NMR (CDCl_3_, 600 MHz) *δ*/ppm: 10.15 (s, 1H), 8.07 (s, 1H), 6.07–6.02 (m, 1H), 5.45 (d, *J* = 9.4 Hz, 1H), 5.36 (d, *J* = 18.2 Hz, 1H), 5.07 (d, *J* = 7.7 Hz, 2H).

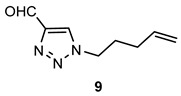

Compound **9** [29] was synthesized from pent-4-en-1-amine (70.5 mg or 90.7 μL, 0.99 mmol) and 1-(4-nitrophenyl)-1*H*-1,2,3-triazol-4-carbaldehyde (180 mg, 0.83 mmol) dissolved in 2 mL of 1,4-dioxane. The obtained product was purified by column chromatography (*ϕ* = 2 cm; *h* = 17 cm; system PE/DCM and DCM/EtOAc (2%)).

1-(pent-4-en-1-yl)-1*H*-1,2,3-triazole-4-carbaldehyde (**9**): 95 mg, 27% of the isolated yield; yellow oil; *R_f_* (DCM/EtOAc (2%)) = 0.47; 1H NMR (CDCl_3_, 300 MHz) δ/ppm: 10.14 (s, 1H), 5.82–5.73 (m, 1H), 5.09–5.06 (m, 2H), 4.44 (t, *J* = 4.4 Hz, 2H), 3.87–3.82 (m, 2H), 3.65–3.60 (m, 2H).

### 3.3. Synthesis of Triazolostilbenes ***10**–**22*** by Wittig Reaction

The apparatus, consisting of a three-necked flask, a dropping funnel, a chlorine-calcium tube, and a balloon filled with nitrogen, was blown with nitrogen for 15 min. A magnet was placed in the flask, the addition funnel was closed, and 30 or 40 mL of absolute ethanol was poured in (depending on the amount of starting reactants). A portion of absolute ethanol (10 or 20 mL) was poured into the flask, and the required amount of triphenylphosphonium salt was added. Sodium previously weighed in PE on an analytical balance with a precision of 0.0001 g was added to the remaining amount of absolute ethanol. After all the sodium had reacted in the ethanol with the evolution of hydrogen, a little portion of NaOEt was added to the flask. The aldehyde was dissolved in ethanol and transferred to a flask, then the rest of the NaOEt from the funnel was added dropwise. The flask was closed with a glass stopper, and the reaction mixture was left to stir in a magnetic stirrer for the next 72 h at room temperature.

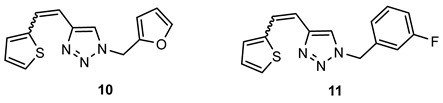

1-(furan-2-ylmethyl)-4-(2-(thiophen-2-yl)vinyl)-1*H*-1,2,3-triazole (**10**). Compound **10** was synthesized by a Wittig reaction from 1-(furan-2-ylmethyl)-1*H*-1,2,3-triazole-4-carbaldehyde (**1**) (99.6 mg, 0.56 mmol) and triphenylphosphonium salt (246.8 mg, 0.56 mmol) with sodium in 10% excess (14.2 mg) and 40 mL of NaOEt. The obtained product was purified by column chromatography (*ϕ* = 3 cm; *h* = 17 cm; PE/E system (50%)). 67.4 mg, 68% of the isolated mixture of isomers; yellow oil; *R_f_* (PE/E (20%)) = 0.51.

1-(3-fluorobenzyl)-4-(2-(thiophen-2-yl)vinyl)-1*H*-1,2,3-triazole (**11**). Compound **11** was synthesized from 1-(3-fluorobenzyl)-1*H*-1,2,3-triazole-4-carbaldehyde (**2**) (143.6 mg, 0.62 mmol) and triphenylphosphonium salt (270.4 mg, 0.62 mmol) with sodium in 10% excess (15.6 mg) and 40 mL of NaOEt. The obtained product was purified by column chromatography (*ϕ* = 3 cm; *h* = 18.5 cm; system PE/E (50%)). 88.3 mg, 50% isolated mixture of isomers; yellow oil; *R_f_* (PE/E (20%)) = 0.53.

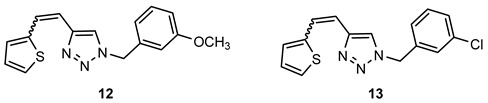

1-(3-methoxybenzyl)-4-(2-(thiophen-2-yl)vinyl)-1*H*-1,2,3-triazole (**12**). Compound **12** was synthesized by a Wittig reaction from 1-(3-methoxybenzyl)-1*H*-1,2,3-triazole-4-carbaldehyde (**3**) (83.0 mg, 0.28 mmol) and triphenylphosphonium salt (122.5 mg, 0.28 mmol) with sodium in 10% excess (7.1 mg) and 30 mL of NaOEt. The obtained product was purified by column chromatography (*ϕ* = 1.5 cm; *h* = 14 cm; system PE/E (50%)). 63.6 mg, 77% isolated mixture of isomers; yellow oil; *R_f_* (PE/E (20%)) = 0.45.

1-(3-chlorobenzyl)-4-(2-(thiophen-2-yl)vinyl)-1*H*-1,2,3-triazole (**13**). Compound **13** was synthesized from 1-(3-chlorobenzyl)-1*H*-1,2,3-triazole-4-carbaldehyde (**4**) (72.6 mg, 0.29 mmol) and triphenylphosphonium salt (127.3 mg, 0.29 mmol) with sodium in 10% excess (7.3 mg) and 30 mL of NaOEt. The obtained product was purified by column chromatography (*ϕ* = 3 cm; *h* = 18.5 cm; system PE/E (50%)). 109.8 mg, 99% of isolated mixture of isomers; yellow oil; *R_f_* (PE/E (20%)) = 0.49.

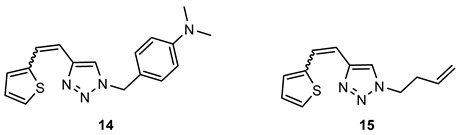

*N*,*N*-dimethyl-4-((4-(2-(thiophen-2-yl)vinyl)-1*H*-1,2,3-triazol-1-yl)methyl)aniline (**14**). Compound **14** was synthesized by a Wittig reaction from 1-(4-(dimethylamino)benzyl)-1*H*-1,2,3-triazole-4-carbaldehyde (**6**) (69.7 mg, 0.30 mmol) and triphenylphosphonium salt (132.9 mg, 0.30 mmol) with sodium in 10% excess (7.6 mg) and 40 mL of NaOEt. The obtained product was purified by column chromatography (*ϕ* = 1.5 cm; *h* = 10 cm; system PE/E (60%)). 19.7 mg, 21% isolated mixture of isomers; yellow oil; *Rf* (PE/E (90%)) = 0.25.

1-(but-3-en-1-yl)-4-(2-(thiophen-2-yl)vinyl)-1*H*-1,2,3-triazole (**15**). Compound **15** was synthesized from 1-(but-3-en-1-yl)-1*H*-1,2,3-triazole-4-carbaldehyde (**8**) (72.5 mg, 0.48 mmol) and triphenylphosphonium salt (210.5 mg, 0.48 mmol) with sodium in 10% excess (12.1 mg) and 40 mL of NaOEt. The obtained product was purified by column chromatography (*ϕ* = 2 cm; *h* = 10 cm; PE/E system (80%)). 80.7 mg, 73% of the isolated mixture of isomers; yellow oil; *R_f_* (PE/E (90%)) = 0.81.

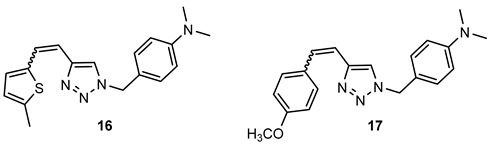

*N*,*N*-dimethyl-4-((4-(2-(5-methylthiophen-2-yl)vinyl)-1*H*-1,2,3-triazol-1-yl)methyl)aniline (**16**). Compound **16** was synthesized from 1-(4-(dimethylamino)benzyl)-1*H*-1,2,3-triazole-4-carbaldehyde (**6**) (69.7 mg, 0.30 mmol) and triphenylphosphonium salt (140.2 mg, 0.30 mmol) with sodium in 10% excess (7.6 mg) and 40 mL of NaOEt. The obtained product was purified by column chromatography (*ϕ* = 1.5 cm; h = 10 cm; system PE/E (70%)). 46.0 mg, 33% of the isolated mixture of isomers; yellow oil; *R_f_* (PE/E (70%)) = 0.12.

4-((4-(3-methoxystyryl)-1*H*-1,2,3-triazol-1-yl)methyl)-*N,N*-dimethylaniline (**17**). Compound **17** was synthesized from 1-(4-(dimethylamino)benzyl)-1*H*-1,2,3-triazole-4-carbaldehyde (**6**) (69.7 mg, 0.30 mmol) and triphenylphosphonium salt (140.2 mg, 0.30 mmol) with sodium in 10% excess (7.6 mg) and 40 mL of NaOEt. The obtained product was purified by column chromatography (*ϕ* = 2 cm; *h* = 10 cm; PE/E system (80%)). 53.6 mg, 53% of the isolated mixture of isomers; yellow oil; *R_f_* (PE/E (90%)) = 0.40.

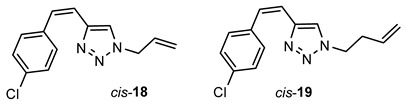

Compound *cis*-**18** was synthesized by a Wittig reaction from 1-allyl-1*H*-1,2,3-triazole-4-carbaldehyde (**7**) (143.8 mg, 0.96 mmol) and triphenylphosphonium salt (140.2 mg, 0.30 mmol) with sodium in 10% excess (7.6 mg) and 40 mL of NaOEt. The obtained product was purified by column chromatography (*ϕ* = 1.5 cm; *h* = 10 cm; system PE/E (70%)). 46.0 mg, 33% of the isolated mixture of isomers; yellow oil; *R_f_* (PE/E (70%)) = 0.65.

(*Z*)-1-allyl-4-(4-chlorostyryl)-1*H*-1,2,3-triazole (*cis*-**18**): 25 mg, 11% isolated yield; yellow oil; *R_f_* (PE/E (50%)) = 0.43; ^1^H NMR (CDCl_3_, 300 MHz) *δ*/ppm: 7.31 (s, 4H), 7.10 (s, 1H), 6.70 (d, *J* = 11.2 Hz, 1H), 6.64 (d, *J* = 11.2 Hz, 1H), 5.96–5.90 (m, 1H), 5.29 (d, *J* = 10.1 Hz, 1H), 5.20 (d, *J* = 16.4 Hz, 1H), 4.87 (d, *J* = 7.9 Hz, 2H); ^13^C NMR (CDCl_3_, 150 MHz) *δ*/ppm: 144.3, 136.0, 133.4, 131.1, 130.0, 129.7, 128.8, 121.5, 120.6, 119.9, 52.4, 30.2.

Compound *cis*-**19** was synthesized according to the general procedure from 1-(but-3-en-1-yl)-1*H*-1,2,3-triazole-4-carbaldehyde (**8**) (139 mg, 1.02 mmol) and triphenyl-phosphonium salt (140.2 mg, 0.30 mmol) with sodium in 10% excess (7.6 mg) and 40 mL of NaOEt. The obtained product was purified by column chromatography (*ϕ* = 2 cm; *h* = 10 cm; PE/E system (80%)). 57.6 mg, 57% of the isolated mixture of isomers; yellow oil; *R_f_* (PE/E (70%)) = 0.68.

4-((4-(3-methoxystyryl)-1*H*-1,2,3-triazol-1-yl)methyl)-*N*,*N*-dimethylaniline (*cis*-**19**): 13 mg, 61% isolated yield; yellow oil; *R_f_* (PE/E (50%)) = 0.51; ^1^H NMR (CDCl_3_, 300 MHz) *δ*/ppm: 7.28 (d, *J* = 10.2 Hz, 4H), 7.07 (s, 1H), 6.71 (d, *J* = 12.4 Hz, 1H) 6.63 (d, *J* = 12.4 Hz, 1H), 5.75–5.62 (m, 1H), 5.09–5.01 (m, 2H), 4.30 (t, *J =* 6.2 Hz, 1H), 2.57 (dd, *J* = 13.2, 6.9 Hz, 2H); ^13^C NMR (CDCl_3_, 150 MHz) *δ*/ppm: 143.9, 136.1, 133.4, 133.1, 129.9, 129.8, 128.8, 121.6, 120.7, 118.5, 49.5, 34.3.

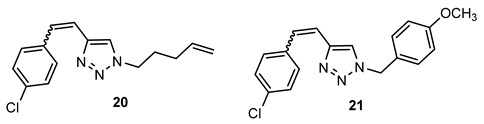

4-(4-chlorostyryl)-1-(pent-4-en-1-yl)-1*H*-1,2,3-triazole (**20**). Compound **20** was synthesized from 1-(pent-4-en-1-yl)-1*H*-1,2,3-triazole-4-carbaldehyde (**9**) (50 mg, 0.30 mmol) and triphenylphosphonium salt (140.2 mg, 0.30 mmol) with sodium in 10% excess (7.6 mg) and 40 mL of NaOEt. The obtained product was purified by column chromatography (*ϕ* = 1.5 cm; *h* = 10 cm; system PE/E (70%)). 46.0 mg, 33% of the isolated mixture of isomers; yellow oil; *R_f_* (PE/E (70%)) = 0.55.

4-(4-chlorostyryl)-1-(4-methoxybenzyl)-1*H*-1,2,3-triazole (**21**). Compound **21** was synthesized according to the general procedure from 1-(4-(dimethylamino)benzyl)-1*H*-1,2,3-triazole-4-carbaldehyde (**6**) (69.7 mg, 0.30 mmol) and triphenylphosphonium salt (140.2 mg, 0.30 mmol) with sodium in 10% excess (7.6 mg) and 40 mL of NaOEt. The obtained product was purified by column chromatography (*ϕ* = 2 cm; *h* = 10 cm; PE/E system (80%)). 62.6 mg, 64% of the isolated mixture of isomers; yellow oil; *R_f_* (PE/E (90%)) = 0.40.

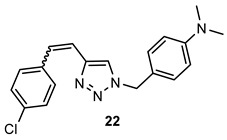

4-((4-(4-chlorostyryl)-1*H*-1,2,3-triazol-1-yl)methyl)-*N*,*N*-dimethylaniline (**22**). Compound **22** was synthesized from 1-(4-(dimethylamino)benzyl)-1*H*-1,2,3-triazole-4-carbaldehyde (**6**) (69.7 mg, 0.30 mmol) and triphenylphosphonium salt (140.2 mg, 0.30 mmol) with sodium in 10% excess (7.6 mg) and 40 mL of NaOEt. The obtained product was purified by column chromatography (*ϕ* = 2 cm; *h* = 10 cm; PE/E system (80%)). 25.2 mg, 25% of the isolated mixture of isomers; yellow oil; *R_f_* (PE/E (90%)) = 0.25.

### 3.4. Photochemical Synthesis of Naphthotriazoles ***23**–**34***

Corresponding 1,2,3-triazoles as mixtures of isomers (except pure isolated *cis*-**18** and *cis*-**19**) were dissolved in 1 to 3 mL of toluene. The mixtures were then transferred to quartz tubes (they transmit light), and the rest of the toluene and a little iodine on the tip of a spatula (oxidizing agent) were added. The solutions (~10^−3^ mol L^−1^) were illuminated for 1–3 h using 10 lamps with a wavelength of 313 nm in a Rayonet photoreactor. The obtained products **23**–**34** were purified by column chromatography. Only compound **20** did not react to give the corresponding photoproduct **35**. The spectroscopic data for the photoproducts **23**–**34** are given below.

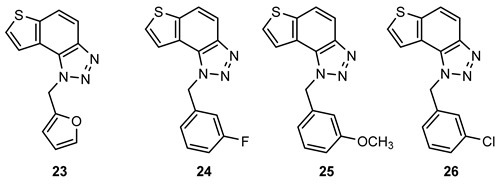

1-(furan-2-ylmethyl)-1*H*-thieno[2′,3′:3,4]benzo[1,2-*d*][1,2,3]triazole (**23**): 10.3 mg, 31% of the isolated compound; yellow oil; *R_f_* (PE/E (30%)) = 0.54; ^1^H NMR (CDCl_3_, 600 MHz) *δ*/ppm: 7.97 (d, *J* = 8.9 Hz, 1H), 7.82–7.80 (m, 2H), 7.66 (d, *J* = 5.5 Hz, 1H), 7.36 (d, *J* = 1.8 Hz, 1H), 6.34 (d, *J* = 3.3 Hz, 1H), 6.32 (dd, *J* = 3.3, 1.8 Hz, 1H), 6.08 (s, 2H); ^13^C NMR (CDCl_3_, 150 MHz) *δ*/ppm: 148.1, 144.5, 143.1, 140.2, 128.6, 127.7, 122.8, 120.3, 119.2, 116.0, 110.9, 109.3, 46.4. MS (ESI) *m*/*z* (%, fragment): 256 (100); HRMS (*m*/*z*) for C_13_H_10_N_3_OS: [M + H]^+^_calcd_ = 255.0466, and [M + H]^+^_measured_ = 255.0463.

1-(3-fluorobenzyl)-1*H*-thieno[2′,3′:3,4]benzo[1,2-*d*][1,2,3]triazole (**24**): 28.2 mg, 64% of the isolated compound; yellow oil; *R_f_* (PE/E (30%)) = 0.51; 1H NMR (CDCl_3_, 600 MHz) *δ*/ppm: 8.00 (d, *J* = 9.0 Hz, 1H), 7.82 (d, *J* = 8.9 Hz, 1H), 7.56 (d, *J* = 5.5 Hz, 1H), 7.46 (d, *J* = 5.5 Hz, 1H), 7.33–7.27 (m, 1H), 7.02–6.93 (m, 2H), 6.86 (d, *J* = 9.3 Hz, 1H), 6.10 (s, 2H); ^13^C NMR (CDCl_3_, 150 MHz) *δ*/ppm: 163.1 (d, *J*_C-F_ = 247 Hz), 144.7, 140.3, 137.7, 130.8, 128.0, 122.5, 119.9, 119.4, 116.1, 115.6, 115.3, 113.7 (d, *J*_C-F_ = 22.5 Hz), 52.5; MS (ESI) *m*/*z* (%, fragment): 284 (50), 122 (100); HRMS (*m*/*z*) for C_15_H_11_FN_3_S: [M + H]^+^_calcd_ = 283.0579, and [M + H]^+^_measured_ = 283.0581.

1-(3-methoxybenzyl)-1*H*-thieno[2′,3′:3,4]benzo[1,2-*d*][1,2,3]triazole (**25**): 9.5 mg, 30% of the isolated compound; yellow oil; *R_f_* (PE/E (30%)) = 0.45; ^1^H NMR (CDCl_3_, 600 MHz) *δ*/ppm: 7.99 (d, *J* = 9.0 Hz, 1H), 7.80 (d, *J* = 8.8 Hz, 1H), 7.54 (d, *J* = 5.5 Hz, 1H), 7.50 (d, *J* = 5.5 Hz, 1H), 7.23 (t, *J* = 2.1 Hz, 1H), 6.81 (d, *J* = 8.3 Hz, 1H), 6.78 (d, *J* = 7.7 Hz, 1H), 6.71 (s, 1H), 6.08 (s, 2H), 3.70 (s, 3H); ^13^C NMR (CDCl_3_, 150 MHz) *δ*/ppm: 160.2, 144.6, 140.1, 136.7, 130.2, 128.7, 127.7, 122.7, 120.3, 119.2, 118.8, 116.1, 113.6, 112.4, 55.2, 53.0.

1-(3-chlorobenzyl)-1*H*-thieno[2′,3′:3,4]benzo[1,2-*d*][1,2,3]triazole (**26**): 21.7 mg, 59% of the isolated compound; yellow oil; *R_f_* (PE/E (30%)) = 0.42; ^1^H NMR (CDCl_3_, 600 MHz) *δ*/ppm: 8.00 (d, *J* = 9.0 Hz, 1H), 7.82 (d, *J* = 8.9 Hz, 1H), 7.58 (d, *J* = 5.5 Hz, 1H), 7.47 (d, *J* = 5.5 Hz, 1H), 7.26–7.25 (m, 1H), 7.24 (t, *J* = 8.0 Hz, 1H), 7.21 (s, 1H), 7.03 (d, *J* = 7.4 Hz, 1H), 6.08 (s, 2H); ^13^C NMR (CDCl_3_, 150 MHz) *δ*/ppm: 144.7, 140.3, 137.1, 135.2, 130.4, 128.7, 128.6, 128.1, 126.7, 124.7, 122.5, 119.8, 119.4, 116.1, 52.4; MS (ESI) *m*/*z* (%, fragment): 300/302 (100); HRMS (*m*/*z*) for C_15_H_11_ClN_3_S: [M + H]^+^_calcd_ = 299.0284, and [M + H]^+^_measured_ = 299.0288.

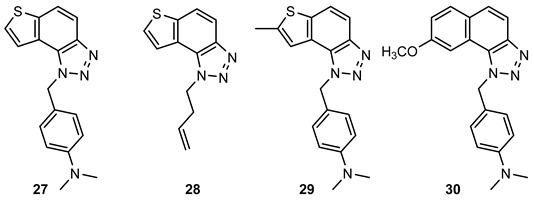

4-((1*H*-thieno[2′,3′:3,4]benzo[1,2-*d*][1,2,3]triazol-1-yl)methyl)-*N*,*N*-dimethylaniline (**27**): 6.4 mg, 33% of the isolated compound; yellow oil; *R_f_* (PE/E (50%)) = 0.24; ^1^H NMR (CDCl_3_, 600 MHz) *δ*/ppm: 7.97 (d, *J* = 8.8 Hz, 1H), 7.78 (d, *J* = 8.8 Hz, 1H), 7.60 (d, *J* = 5.5 Hz, 1H), 7.56 (d, *J* = 5.5 Hz, 1H), 7.11 (d, *J* = 8.8 Hz, 2H), 6.64 (d, *J* = 8.7 Hz, 2H), 6.01 (s, 2H), 2.88 (s, 3H); ^13^C NMR (CDCl_3_, 150 MHz) *δ*/ppm: 150.4, 144.7, 139.9, 129.4, 128.6, 127.8, 127.5, 126.6, 122.9, 122.5, 120.6, 119.0, 116.0, 112.6, 52.9, 40.4; MS (ESI) *m*/*z* (%, fragment): 309 (100); HRMS (*m*/*z*) for C_17_H_17_N_4_S: [M + H]^+^_calcd_ = 308.1096, and [M + H]^+^_measured_ = 308.1100.

1-(but-3-en-1-yl)-1*H*-thieno[2′,3′:3,4]benzo[1,2-*d*][1,2,3]triazole (**28**): 24 mg, 81% isolated compound; yellow oil; *R_f_* (PE/E (30%)) = 0.48; ^1^H NMR (CDCl_3_, 600 MHz) *δ*/ppm: 7.96 (d, *J* = 8.9 Hz, 1H), 7.80 (d, *J* = 8.9 Hz, 1H), 7.74 (d, *J* = 5.5 Hz, 1H), 7.70 (d, *J* = 5.5 Hz, 1H), 5.90–5.83 (m, 1H), 5.15–5.08 (m, 2H), 4.96 (t, *J* = 7.4 Hz, 2H), 2.85–2.80 (q, *J* =14.5, 7.2 Hz, 2H); ^13^C NMR (CDCl_3_, 150 MHz) *δ*/ppm: 144.4, 139.9, 133.1, 128.5, 128.0, 122.6, 119.6, 119.0, 118.4, 116.2, 49.0, 34.3; MS (ESI) *m*/*z* (%, fragment): 230 (40), 122 (100); HRMS (*m*/*z*) for C_12_H_12_N_3_S: [M + H]^+^_calcd_ = 229.0674, and [M + H]^+^_measured_ = 229.0676.

*N,N*-dimethyl-4-((7-methyl-1*H*-thieno[2′,3′:3,4]benzo[1,2-*d*][1,2,3]triazol-1-yl)methyl)aniline (**29**): 12.6 mg, 36% of the isolated compound; yellow oil; *R_f_* (PE/E (50%)) = 0.29; ^1^H NMR (CDCl_3_, 600 MHz) *δ*/ppm: 7.88 (d, *J* = 8.8 Hz, 1H), 7.64 (d, *J* = 8.8 Hz, 1H), 7.27 (s, 1H), 7.11 (d, *J* = 8.8 Hz, 2H), 6.64 (d, *J* = 8.6 Hz, 2H), 5.96 (s, 2H), 2.88 (s, 3H), 2.61 (s, 3H); ^13^C NMR (CDCl_3_, 150 MHz) *δ*/ppm: 150.4, 144.7, 142.4, 139.1, 129.4, 128.2, 127.8, 123.2, 118.7, 118.4, 115.0, 112.6, 52.7, 40.4, 16.3.

4-((8-methoxy-1*H*-naphtho[1,2-*d*][1,2,3]triazol-1-yl)methyl)-*N*,*N*-dimethylaniline (**30**): 7.2 mg, 20% of the isolated compound; yellow oil; *R_f_* (PE/E (50%)) = 0.35; ^1^H NMR (CDCl_3_, 600 MHz) *δ*/ppm: 7.86 (d, *J =* 8.5 Hz, 1H), 8.84 (d, *J* = 8.5 Hz, 1H), 7.62 (d, *J* = 9.0 Hz, 1H), 7.50 (d, *J* = 2.4 Hz, 1H), 7.17 (dd, *J* = 8.9, 2.6 Hz, 1H), 7.06 (d, *J* = 8.7 Hz, 2H), 6.64 (d, *J* = 8.3 Hz, 2H), 6.16 (s, 2H), 3.79 (s, 3H), 2.89 (s, 3H); ^13^C NMR (CDCl_3_, 150 MHz) *δ*/ppm: 158.5, 150.4, 145.3, 130.5, 129.2, 128.9, 127.7, 127.3, 126.0, 121, 0, 117.7, 115.5, 112.8, 103.7, 55.6, 53.9, 40.5; MS (ESI) *m*/*z* (%, fragment): 333 (20), 134 (100); HRMS (*m*/*z*) for C_20_H_21_N_4_O: [M + H]^+^_calcd_ = 332.1637, and [M + H]^+^_measured_ = 332.1635.

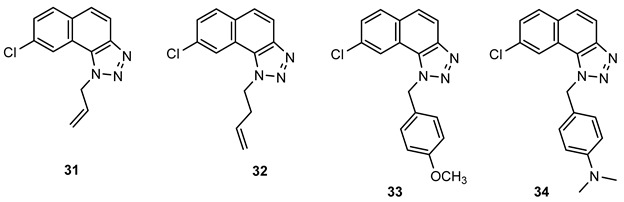

1-allyl-8-chloro-1*H*-naphtho[1,2-*d*][1,2,3]triazole (**31**): 5.1 mg, 17% of the isolated compound; yellow oil; *R_f_* (PE/E (30%)) = 0.60; ^1^H NMR (CDCl_3_, 300 MHz) *δ*/ppm: 8.14 (d, *J* = 1.8 Hz, 1H), 7.94 (d, *J* = 9.3 Hz, 1H), 7.89 (d, *J* = 8.5 Hz, 1H), 7.63 (d, *J* = 8.9 Hz, 1H), 7.53 (dd, *J* = 9.1, 2.5 Hz, 1H), 6.17–6.10 (m, 1H), 5.05–5.01 (dt, *J* = 17.2, 2.5 Hz, 1H), 5.6–5.58 (m, 2H), 5.32 (d, *J* = 11.3 Hz, 1H), 5.03 (d, *J* = 17.3 Hz, 1H); ^13^C NMR (CDCl_3_, 150 MHz) *δ*/ppm: 158.6, 143.9, 132.0, 130.3, 129.7, 127.6, 126.6, 124.9, 120.9, 119.5, 117.9, 117.5, 51.6, 28.7.

1-(but-3-en-1-yl)-8-chloro-1*H*-naphtho[1,2-*d*][1,2,3]triazole (**32**): 5.4 mg, 18% of the isolated compound; *R_f_* (PE/E (30%)) = 0.51; ^1^H NMR (CDCl_3_, 300 MHz) *δ*/ppm: 8.15 (d, *J* = 2.5 Hz, 1H), 7.95 (d, *J* = 8.6 Hz, 1H), 7.90 (d, *J* = 8.6 Hz, 1H), 7.63 (d, *J* = 8.8 Hz, 1H), 7.53 (dd, *J* = 9.3, 2.4 Hz, 1H), 6.20–6.05 (m, 1H), 5.60–5.59 (m, 2H), 5.43 (t, *J* = 15.3 Hz, 1H), 5.33–5.22 (m, 2H), 5.02 (d, *J* = 16.8 Hz, 1H); ^13^C NMR (CDCl_3_, 150 MHz) *δ*/ppm: 132.0, 130.3, 129.8, 129.7, 126.6, 124.9, 120.9, 119.5, 117.9, 117.5, 51.6, 30.9, 28.6, 21.6.

8-chloro-1-(4-methoxybenzyl)-1*H*-naphtho[1,2-*d*][1,2,3]triazole (**33**): 5.7 mg; 19% of the isolated compound; *R_f_* (PE/E(30%)) = 0.49; ^1^H NMR (CDCl_3_, 300 MHz) *δ*/ppm: 8.14 (s, 1H), 8.02 (d, *J* = 8.6 Hz, 1H), 7.90 (d, *J* = 8.6 Hz, 1H), 7.67 (d, *J* = 8.8 Hz, 1H), 7.52 (d, *J* = 8.6 Hz, 1H), 7.15 (d, *J* = 8.6 Hz, 1H), 6.84 (d, *J* = 8.6 Hz, 1H), 6.17 (s, 2H), 3.75 (s, 3H); ^13^C NMR (CDCl_3_, 150 MHz) *δ*/ppm: 158.6, 131.9, 130.2, 129.6, 126.9, 126.8, 126.5, 125.1, 124.8, 121.1, 119.5, 117.4, 113.6, 54.2, 52.7, 28.7.

4-((8-chloro-1*H*-naphtho[1,2-*d*][1,2,3]triazol-1-yl)methyl)-*N*,*N*-dimethylaniline (**34**): 12.2 mg, 40% of the isolated compound; *R_f_* (PE/E (50%)) = 0.75; ^1^H NMR (CDCl_3_, 300 MHz) *δ*/ppm: 8.24 (s, 1H), 8.01 (d, *J* = 9.4 Hz, 1H), 7.88 (d, *J* = 8.6 Hz, 1H), 7.65 (d, *J* = 8.8 Hz, 1H), 7.51 (d, *J* = 8.6 Hz, 1H), 7.12 (d, *J* = 8.6 Hz, 2H), 6.66 (d, *J* = 8.6 Hz, 2H), 6.12 (s, 2H), 2.90 (s, 6H); ^13^C NMR (CDCl_3_, 150 MHz) *δ*/ppm: 150.4, 145.2, 132.9, 131.2, 130.5, 128.6, 127.8, 127.4, 125.7, 122.3; 120.7, 118.4, 112.7, 53.9, 40.4.

### 3.5. General Methylation Procedure for the Synthesis of ***44**–**51***

In a glass reaction tube equipped with a magnetic stirring bar, the corresponding thienobenzo-triazoles **36**–**43** (0.2 mmol, 1equiv), dry dichloromethane (p.a., 0.4 mL), and iodomethane (2 mmol, 10 equiv.) were added. Firstly, the reaction mixtures were purged with nitrogen, N_2_, and stirred at 60 °C for 24 h. After 24 h, the reaction mixtures were cooled to 0 °C and diluted with 5 mL of diethyl ether. After precipitation, the reaction tubes were centrifuged three times for 10 min at 3000 rpm. The solvent was decanted, and the precipitate was washed with diethyl ether five times. The crude powders were dried under a high vacuum to afford the pure triazolium salts **44**–**51**.

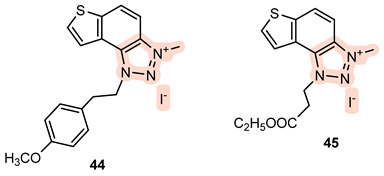

1-(4-methoxyphenethyl)-3-methyl-1*H*-thieno[3′,2′:3,4]benzo[1,2-*d*][1,2,3]triazol-3-ium iodide **44**: 30 mg, 50% isolated yield; yellow powder; *R_f_* (DCM (100%)) = 0.13; ^1^H NMR (CDCl_3_, 600 MHz) *δ*/ppm: 8.32 (d, *J* = 9.3 Hz, 1H), 8.23 (d, *J* = 9.1 Hz, 1H), 8.03 (d, *J* = 5.6 Hz, 1H), 7.96 (d, *J* = 5.2 Hz, 1H), 7.06 (d, *J* = 8.4, 2H), 6.79 (d, *J* = 8.4 Hz, 2H), 5.37 (t, *J* = 7.4 Hz, 2H), 4.81 (s, 3H), 3.76 (s, 3H), 3.50 (t, *J* = 7.4 Hz, 2H).; ^13^C NMR (CDCl_3_, 150 MHz) *δ*/ppm: 159.2, 142.9, 134.7, 133.0, 130.8, 129.8, 127.1, 126.8, 122.7, 120.1, 114.6, 109.2, 53.3, 53.2, 40.1, 34.2; MS (ESI) *m*/*z* (%, fragment): 324 (100); HRMS (*m*/*z*) for C_18_H_18_N_3_OS: [M + H]^+^_calcd_ = 323.1092, and [M + H]^+^_measured_ = 323.1089.

1-(3-ethoxy-3-oxopropyl)-3-methyl-1*H*-thieno[3′,2′:3,4]benzo[1,2-*d*][1,2,3]triazol-3-ium iodide **45**: 30 mg, 48% isolated yield; yellow powder; *R_f_* (DCM (100%)) = 0.15; ^1^H NMR (CDCl_3_, 600 MHz) *δ*/ppm: 8.31 (d, *J* = 9.2 Hz, 1H), 8.19 (d, *J* = 5.3 Hz, 1H), 8.11 (d, *J* = 9.2 Hz, 1H), 8.05 (d, *J* = 5.8 Hz, 1H), 5.52 (t, *J* = 6.2 Hz, 1H), 4.78 (s, 3H), 4.15 (q, 2H), 3.48 (t, *J* = 5.8 Hz, 2H); ^13^C NMR (CDCl_3_, 150 MHz) *δ*/ppm: 169.8, 142.9, 134.7, 133.0, 131.4, 127.1, 123.1, 120.8, 108.6, 61.7, 49.2, 39.9, 32.7, 14.1; MS (ESI) *m*/*z* (%, fragment): 290 (100), 214 (30); HRMS (*m*/*z*) for C_14_H_16_N_3_O_2_S: [M + H]^+^_calcd_ = 289.0885, and [M + H]^+^_measured_ = 289.0889.

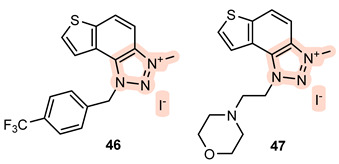

3-methyl-1-(4-(trifluoromethyl)benzyl)-1*H*-thieno[3′,2′:3,4]benzo[1,2-*d*][1,2,3]triazol-3-ium iodide **46**: 11 mg, 53% isolated yield; yellow powder; *R_f_* (DCM (100%)) = 0.18; ^1^H NMR (CDCl_3_, 600 MHz) *δ*/ppm: 8.34 (d, *J* = 9.4 Hz, 1H), 8.06 (d, *J* = 9.4 Hz, 1H), 7.97 (d, *J* = 5.9 Hz, 1H), 7.84 (d, *J* = 5.1 Hz, 1H), 7.69 (d, *J* = 8.1 Hz, 2H), 6.64 (d, *J* = 8.1 Hz, 2H), 6.57 (s, 2H), 4.84 (s, 3H); ^13^C NMR (CDCl_3_, 150 MHz) *δ*/ppm: 143.3, 135.1, 134.6, 133.3, 132.2, 131.2, 130.4, 128.5, 127.3, 126.7, 124.1, 122.8, 120.5, 108.4, 56.7, 39.9 (the characteristic CF_3_ coupling is not detected due to an insufficient number of scans); MS (ESI) *m*/*z* (%, fragment): 348 (100); HRMS (*m*/*z*) for C_17_H_13_F_3_N_3_S: [M + H]^+^_calcd_ = 347.0704, and [M + H]^+^_measured_ = 347.0708.

3-methyl-1-(2-morpholinoethyl)-1*H*-thieno[3′,2′:3,4]benzo[1,2-*d*][1,2,3]triazol-3-ium iodide **47**: 36 mg, 99% isolated yield; yellow powder; *R_f_* (DCM (100%)) = 0.11; ^1^H NMR (CDCl_3_, 600 MHz) *δ*/ppm: 8.35 (d, *J* = 8.8 Hz, 1H), 8.17 (d, *J* = 9.1 Hz, 1H), 8.11 (d, *J* = 5.9 Hz, 1H), 8.08 (d, *J* = 5.4 Hz, 1H), 5.36 (t, *J* = 6.3 Hz, 2H), 4.85 (s, 3H), 3.59 (t, *J* = 4.8 Hz, 4H), 3.21 (t, *J* = 6.5 Hz, 2H), 2.57 (t, *J* = 5.2 Hz, 4H); ^13^C NMR (CDCl_3_, 150 MHz) *δ*/ppm: 143.0, 134.7, 133.2, 131.0, 127.0, 122.9, 120.3, 108.5, 66.7, 56.1, 53.5, 51.5, 39.9; MS (ESI) *m*/*z* (%, fragment): 303 (100); HRMS (*m*/*z*) for C_15_H_19_N_4_OS: [M + H]^+^_calcd_ = 302.1201, and [M + H]^+^_measured_ = 302.1205.

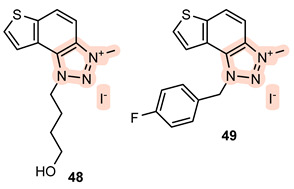

1-(4-hydroxybutyl)-3-methyl-1*H*-thieno[3′,2′:3,4]benzo[1,2-*d*][1,2,3]triazol-3-ium iodide **48**: 3 mg, 10% isolated yield; yellow powder; *R_f_* (DCM (100%)) = 0.07; ^1^H NMR (CDCl_3_, 600 MHz) *δ*/ppm: 8.33 (d, *J* = 8.9 Hz, 1H), 8.13 (d, *J* = 5.2 Hz, 1H), 8.08 (d, *J* = 6.2 Hz, 1H), 8.01 (d, *J* = 8.9 Hz, 1H), 5.30 (t, *J* = 8.0 Hz, 2H), 4.83 (s, 3H), 3.78 (q, 2H), 2.44 (t, *J* = 7.9 Hz, 2H), 2.36–2.33 (m, 2H), 1.92–1.88 (m, 2H); MS (ESI) *m*/*z* (%, fragment): 262 (100), 216 (30); HRMS (*m*/*z*) for C_13_H_16_N_3_OS: [M + H]^+^_calcd_ = 261.0936, and [M + H]^+^_measured_ = 261.0937.

1-(4-fluorobenzyl)-3-methyl-1*H*-thieno[3′,2′:3,4]benzo[1,2-*d*][1,2,3]triazol-3-ium iodide **49**: 16 mg, 25% isolated yield; yellow powder; *R_f_* (DCM (100%)) = 0.22; ^1^H NMR (CDCl_3_, 600 MHz) *δ*/ppm: 8.34 (d, *J* = 9.1 Hz, 1H), 8.11 (d, *J* = 9.1 Hz, 1H), 7.99 (d, *J* = 5.7 Hz, 1H), 7.86 (d, *J* = 5.7 Hz, 1H), 7.49 (dd, *J* = 8.9, 4.5 Hz, 2H), 7.13 (t, *J* = 8.6 Hz, 2H), 6.42 (s, 2H), 4.83 (s, 3H); ^13^C NMR (CDCl_3_, 150 MHz) *δ*/ppm: 135.1, 133.0, 131.2, 130.1, 130.0, 127.2, 122.9, 120.5, 116.9, 116.8, 109.6, 108.6, 56.7, 29.7 (the characteristic CF coupling is not detected); MS (ESI) *m*/*z* (%, fragment): 298 (100); HRMS (*m*/*z*) for C_16_H_13_FN_3_S: [M + H]^+^_calcd_ = 297.0736, and [M + H]^+^_measured_ = 297.0742.

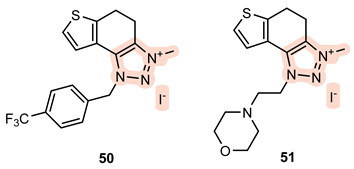

3-methyl-1-(4-(trifluoromethyl)benzyl)-4,5-dihydro-1*H*-thieno[3′,2′:3,4]benzo[1,2-*d*][1,2,3]triazol-3-ium iodide **50**: 9 mg, 98% isolated yield; yellow powder; *R_f_* (DCM (100%)) = 0.19; ^1^H NMR (CDCl_3_, 600 MHz) *δ*/ppm: 7.71 (d, *J* = 8.4 Hz, 2H), 7.55 (d, *J* = 8.1 Hz, 2H), 7.29 (d, *J* = 5.2 Hz, 1H), 7.03 (d, *J* = 5.7 Hz, 1H), 5.92 (s, 2H), 4.49 (s, 3H), 3.35 (s, 4H); ^13^C NMR (CDCl_3_, 150 MHz) *δ*/ppm: 145.4, 144.4, 139.7, 136.6, 134.6, 127.9, 126.8, 126.7, 126.7, 120.9, 55.0, 39.8, 23.4, 20.9 (the characteristic CF_3_ coupling is not detected due to an insufficient number of scans); MS (ESI) *m*/*z* (%, fragment): 350 (100); HRMS (*m*/*z*) for C_17_H_15_F_3_N_3_S: [M + H]^+^_calcd_ = 349.0860, and [M + H]^+^_measured_ = 349.0864.

3-methyl-1-(2-morpholinoethyl)-4,5-dihydro-1*H*-thieno[3′,2′:3,4]benzo[1,2-*d*][1,2,3]triazol-3-ium iodide **51**: 4 mg, 30% isolated yield; yellow powder; *R_f_* (DCM (100%)) = 0.10; ^1^H NMR (CDCl_3_, 600 MHz) *δ*/ppm: 7.39 (d, *J* = 5.8 Hz, 1H), 7.34 (d, *J* = 5.5 Hz, 1H), 4.76 (t, *J* = 6.6 Hz, 2H), 4.45 (s, 3H), 3.61 (t, *J* = 4.5 Hz, 4H), 3.56 (d, *J* = 7.3 Hz, 2H), 3.52 (d, *J* = 7.7 Hz, 2H), 3.01 (t, *J* = 6.6 Hz, 2H), 2.54 (t, *J* = 4.1 Hz, 4H); ^13^C NMR (CDCl_3_, 150 MHz) *δ*/ppm: 143.9, 138.9, 136.3, 126.5, 121.2, 120.9, 66.7, 56.5, 53.7, 50.1, 39.8, 23.5, 20.9; MS (ESI) *m*/*z* (%, fragment): 305 (100), 214 (45); HRMS (*m*/*z*) for C_15_H_21_N_4_OS: [M + H]^+^_calcd_ = 304.1358, and [M + H]^+^_measured_ = 304.1364.

### 3.6. ChEs Inhibition

Ellman’s modified spectrometric method [36] was used to evaluate the inhibition of AChE and BchE. AchE (EC 3.1.1.7, *Electrophorus electricus*, Type: V-S), BchE (EC 3.1.1.8, equine serum), and 2-amino-2-(hydroxymethyl)-1,3-propanediol (Trisma base) were purchased from Sigma-Aldrich (St. Louis, MO, USA) as well as substrates acetylthiocholine iodide (ATChI) and *S*-butyrylthiocholine iodide (BTChI). Ellman’s reagent 5,50-dithiobis-(2-nitrobenzoic acid) (DTNB) was purchased from Zwijndrecht (Antwerpen, Belgium). Each tested compound was dissolved in ethanol, and aliquots of 10 µL were added to 180 µL Tris buffer (50 mM, pH 8.0) and 10 µL of the enzyme (final concentration 0.03 U/mL). Then, 10 µL of DTNB (final concentration 0.3 mM) was added, and finally, the reaction was initiated by adding 10 µL of ATChI/BTChI (final concentration of 0.5 mM). Non-enzymatic hydrolysis was measured as a blank for each measurement, where enzyme volume was replaced by 10 µL of buffer solution. The absorbance of the reaction mixture was measured at 405 nm over 5 min at room temperature using a 96-well microplate reader (Bio Tek 800TSUV Absorbance Reader, Agilent, Santa Clara, CA, USA). In control measurement, the tested compound (inhibitor) was replaced by a buffer solution to determine 100% enzyme activity. The experiments were run at least in triplicate. The percentage of cholinesterase inhibition was calculated due to the following equation: inhibition (%) = [(*A*_c_ − *A*_T_)/*A*_C_]∙100. *A*_*C*_ represents the enzyme activity without the test sample, and *A*_T_ is the enzyme activity with the test sample, calculated as mean values ± standard deviations. The IC_50_ values were obtained graphically by a nonlinear fit of inhibitor concentration (log) values vs. response.

### 3.7. Computational Details

The geometries of ligands were optimized at the B3LYP/6-31G(d) level of theory using the Gaussian16 program package [40]. The molecular electrostatic potential maps were calculated from the total SCF density obtained at the same level of theory. Molecular docking was conducted using the Autodock program suite [41]. The crystallographic data of acetylcholinesterase (AChE) and butyrylcholinesterase (BChE), identified by their respective Protein Data Bank (PDB) codes 4EY7 and 3DJY, were obtained from the Protein Data Bank archives [42,43]. The molecular docking was performed employing the Lamarckian Genetic Algorithm, which yielded a set of 25 genetic algorithm dockings, i.e., 25 binding poses for each of the investigated ligands. The residues of the enzymes were intentionally kept rigid during the docking process, simplifying the procedure and shortening computational time, although this may have resulted in slightly less accurate predictions compared to simulations allowing for residue flexibility.

## 4. Conclusions

In this study, new naphtho- and thienobenzo-triazoles **23**–**34** and triazolium salts **44**–**51** were synthesized, characterized, and spectroscopically and biologically evaluated. Triazolium salts **44**–**46** exhibited excellent inhibition, and salts **47** and **49** demonstrated very good inhibition of both BChE and AChE enzymes. In contrast, neutral photoproducts were shown to be selective towards BChE, even with very good inhibition potential (molecules **24**–**27**). The representatives of newly prepared salts, **45** and **50**, remained stable in aqueous solution and displayed intriguing fluorimetric properties, characterized by strong Stokes shifts exceeding 160 nm. Despite their condensed polycyclic structure, the studied compounds did not interact with ds-DNA, likely due to the unfavorable steric hindrance of substituents. However, these dyes exhibited the capability to bind to proteins, displaying diverse inhibition properties towards AChE and BChE. The molecular docking of the best-performing candidates to cholinesterases’ active site identified cation–π and π-π interactions as being responsible for the stability of the enzyme–ligand complexes. Regarding the safety of triazolium salts **44**–**51**, no structural alerts were found by the Derek Nexus. They can be considered the safest choices for further development into drug formulations, particularly given their high inhibitory potential against AChE and BChE.

## Figures and Tables

**Table 1:** Spectrophotometric properties of studied compounds.

Compound	*λ*_max_/nm	*ε*/mol dm^−3^ cm^−1^	^1^ *λ*_em_/nm	Apparent Stokes Shift/nm
**45**	310	12,851.5	480	170
**50**	296 265	12,529.3 10,693.2	456	160

^1^ excitation at the longest absorption wavelength.

**Table 2:** Inhibition of AChE and BChE and calculated IC_50_ values by compounds **23**–**34** and **44**–**51**.

Compound	AChE		BChE	
	% Inhibition *	IC_50_/μM	% Inhibition *	IC_50_/μM
**23**	30.1 ± 0.3 (250)	-	75.4 ± 3.0 (250)	70.0
**24**	70.8 ± 4.2 (200)	113.0	68.9 ± 2.7 (100)	31.0
**25**	32.6 ± 6.9 (100)	-	70.0 ± 2.5 (75)	29.3
**26**	26.5 ± 1.0 (100)	-	69.7 ± 1.7 (75)	20.6
**27**	24.3 ± 1.2 (100)	-	64.0 ± 3.4 (100)	31.1
**28**	44.7 ± 1.6 (500)	-	71.9 ± 4.4 (150)	79.4
**29**	53.2 ± 0.8 (100)	-	57.3 ± 0.8 (200)	92.2
**30**	63.3 ± 0.8 (250)	171.0	33.2 ± 1.7 (250)	-
**31**	50.1 ± 2.4 (241)	-	53.4 ± 1.99 (241)	232.5
**32**	58.1 ± 2.0 (250)	195.4	71.6 ± 2.7 (250)	71.0
**33**	33.9 ± 1.8(250)	-	52.1 ± 1.7 (250)	195.4
**34**	50.3 ± 1.9 (200)	-	39.7 ± 0.8 (150)	-
**44**	78.7 ± 2.6 (25)	1.8	71.3 ± 2.0 (1)	0.3
**45**	80.6 ± 1.8 (50)	4.3	74.0 ± 7.0 (50)	9.2
**46**	82.6 ± 0.6 (250)	6.9	79.4 ± 2.2 (25)	1.8
**47**	74.6 ± 0.6 (250)	15.3	86.5 ± 5.1 (150)	9.8
**48**	57.2 ± 0.7 (250)	167.6	78.6 ± 2.0 (250)	37.2
**49**	82.7 ± 4.2 (250)	6.7	82.7 ± 4.2 (250)	9.4
**50**	76.3 ± 2.1 (250)	29.3	81.0 ± 0.3 (250)	29.7
**51**	74.1 ± 5.0 (250)	20.3	74.1 ± 5.0 (250)	17.5
**Galantamine**	90.0 ± 1.5 (60)	0.15	90.1 ± 3.4 (4.5)	7.9

* Numbers given in parentheses represent maximal concentrations tested in μM.

**Table 3:** Mutagenic potential of naphtho- and thienobenzo-triazoles **23**–**34** and triazolium salts **44**–**51** by Lhasa M7 evaluation (green square—negative, red square—positive, white square—no data available); grey highlight—negative, orange highlight—positive, white—strongly negative).

Structure	ICH M7 Class	Derek Prediction	Sarah Prediction	Similarity to API	Overall In Silico
**23**	Inconclusive	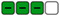	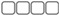	No Derek Alerts found	Negative
**24**	Class 3	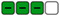	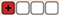	No Derek Alerts found	Positive
**25**	Class 3	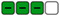	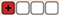	No Derek Alerts found	Positive
**26**	Class 3	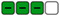	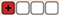	No Derek Alerts found	Positive
**27**	Class 3	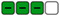	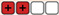	No Derek Alerts found	Positive
**28**	Class 5	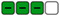	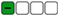	No Derek Alerts found	Negative
**29**	Class 3	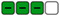	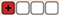	No Derek Alerts found	Positive
**30**	Class 3	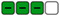	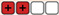	No Derek Alerts found	Positive
**31**	Class 3	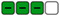	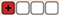	No Derek Alerts found	Positive
**32**	Class 5	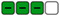	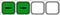	No Derek Alerts found	Negative
**33**	Class 3	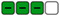	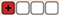	No Derek Alerts found	Positive
**34**	Class 3	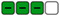	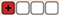	No Derek Alerts found	Positive
**44**	Inconclusive	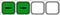	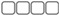	No Derek Alerts found	Negative
**45**	Inconclusive	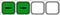	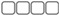	No Derek Alerts found	Negative
**46**	Inconclusive	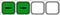	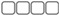	No Derek Alerts found	Negative
**47**	Inconclusive	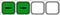	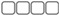	No Derek Alerts found	Negative
**48**	Inconclusive	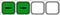	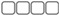	No Derek Alerts found	Negative
**49**	Inconclusive	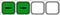	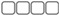	No Derek Alerts found	Negative
**50**	Inconclusive	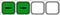	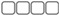	No Derek Alerts found	Negative
**51**	Inconclusive	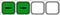	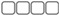	No Derek Alerts found	Negative

## Data Availability

The data presented in this study are available on request from the corresponding author. The data are not publicly available due to privacy.

## Supplementary Materials

The following supporting information can be downloaded at https://www.mdpi.com/article/10.3390/molecules29071622/s1, NMR spectra (Figures S1–S95), mass spectra and HRMS analyses (Figures S96–S109), spectrophotometric data and DNA binding (Figures S110–S121), and Cartesian coordinates of ligands docked into AChE and BChE (Tables S1–S3).
