# Supplementary material for: New Charged Cholinesterase Inhibitors: Design, Synthesis, and Characterization
Source: Molecules. 2024 Apr 4;29(7):1622. doi: 10.3390/molecules29071622 (PMC11013433; doi:10.3390/molecules29071622)
Supplement: Supplementary file 1 [file molecules-29-01622-s001.zip › molecules-2917887-supplementary.pdf]

Electronic Supporting Information

Article

New charged cholinesterase inhibitors; Design, synthesis and characterization

Milena Mlakić [1], Danijela Barić [2], Ana Ratković [3], Ivana Šagud [4], Ivona Čipor [5], Ivo Piantanida [5], Ilijana Odak [6,*] and Irena Škorić[1,*]

[1] Department of Organic Chemistry, Faculty of Chemical Engineering and Technology, University of Zagreb, Trg Marka Marulića 19, HR-10 000 Zagreb, Croatia
[2] Group for Computational Life Sciences, Division of Physical Chemistry, Ruđer Bošković Institute, Bijenička cesta 54, HR-10 000 Zagreb, Croatia
[3] Chemistry, Selvita Ltd., Prilaz Baruna Filipovića 29, HR-10 000 Zagreb, Croatia
[4] Croatian Agency for Medicinal Products and Medical Devices, Ksaverska Cesta 4, HR-10 000 Zagreb, Croatia
[5] Division of Organic Chemistry and Biochemistry, Ruđer Bošković Institute
[6] Department of Chemistry, Faculty of Science and Education, University of Mostar, Matice hrvatske bb, 88000 Mostar, Bosnia and Herzegovina
* Correspondence: ilijana.odak@fpmoz.sum.ba (I.O.); iskoric@fkit.unizg.hr (I.Š.)

Content:

1. NMR spektra……………………………………………………………………………………………………..2

2. Mass spectra and HRMS analyses……………………………………………………………………………97

3. Spectrophotometric data and DNA binding…………………………………………………………………...111

4. Cartesian coordinates of ligands docked into AChE and BChE……………………………………………..117

1. NMR spectra

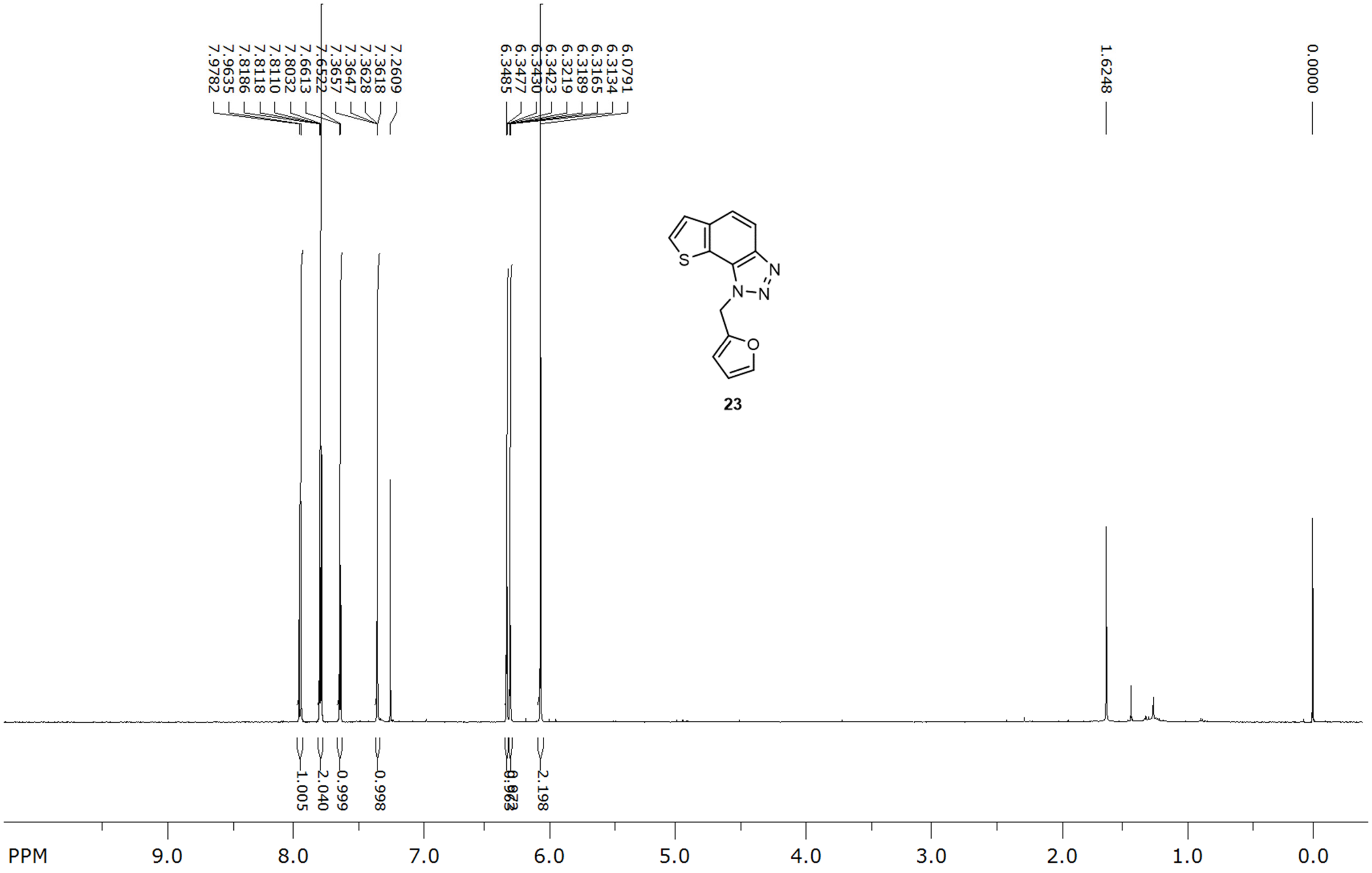

Figure S1. ^{1}H NMR ($CDCl_3$) spectrum of **23**.

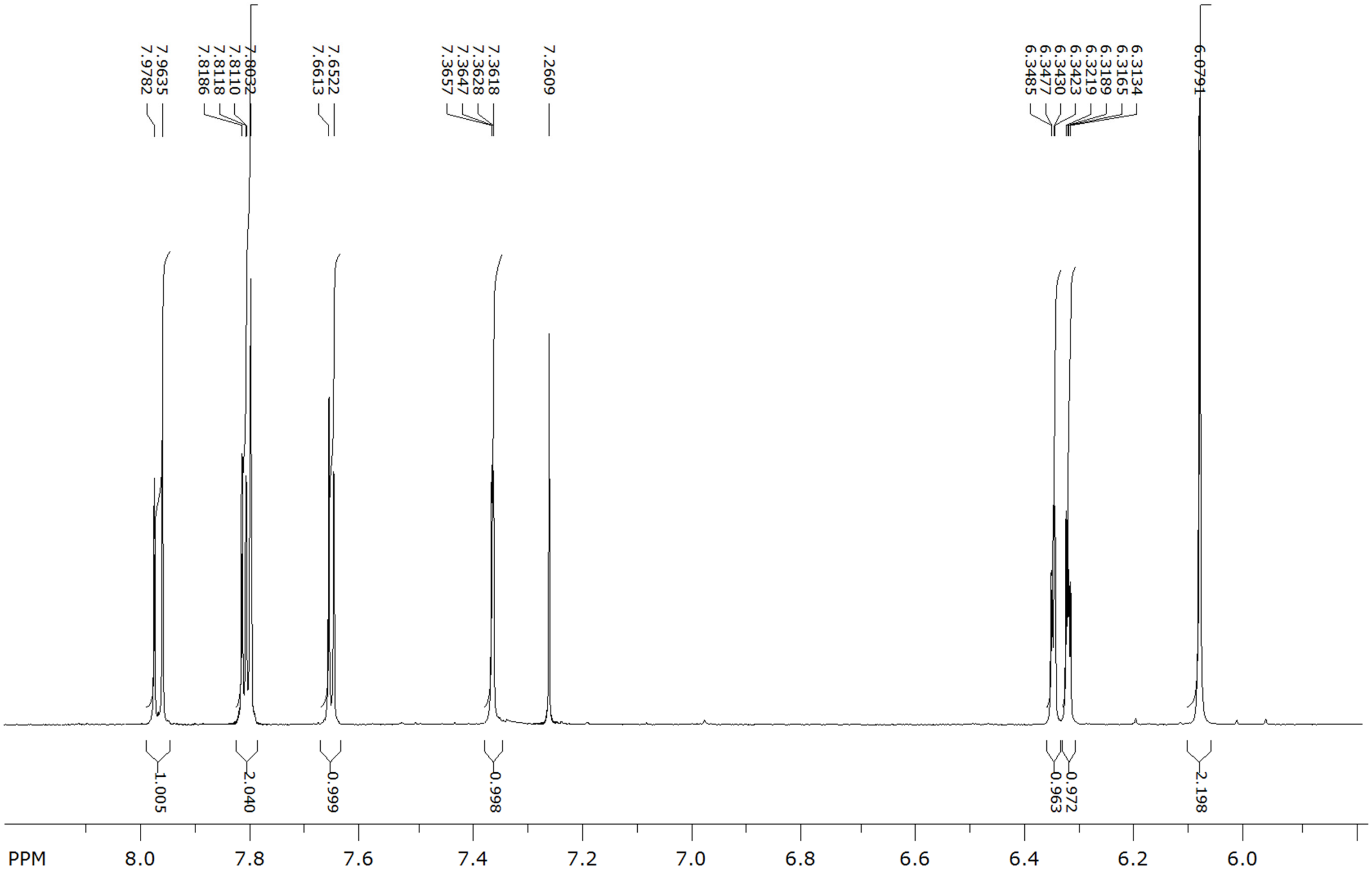

Figure S2. ^{1}H NMR ($CDCl_3$) spectrum of aromatic part of **23**.

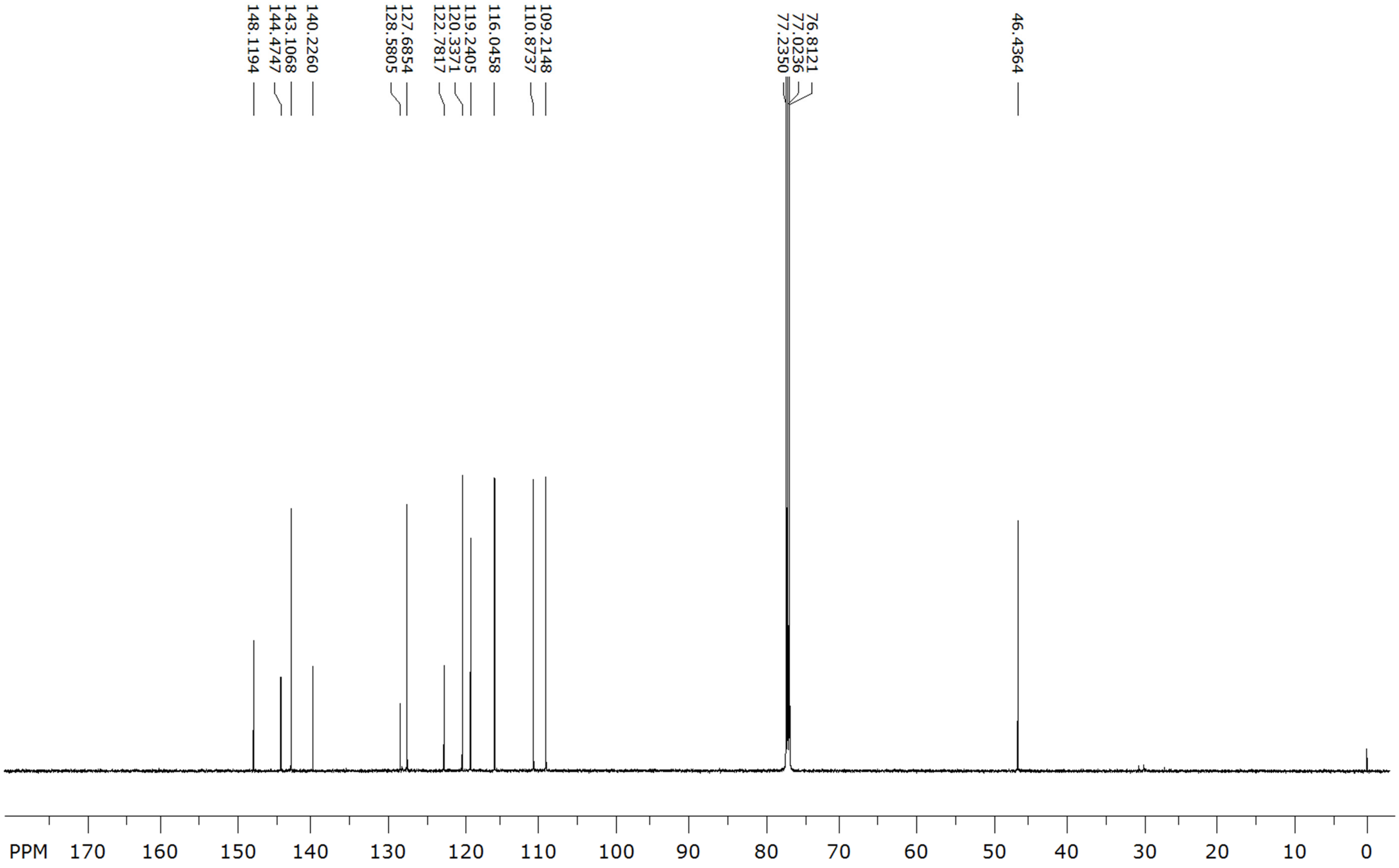

Figure S3. ^{13}C NMR ($CDCl_3$) spectrum of **23**.

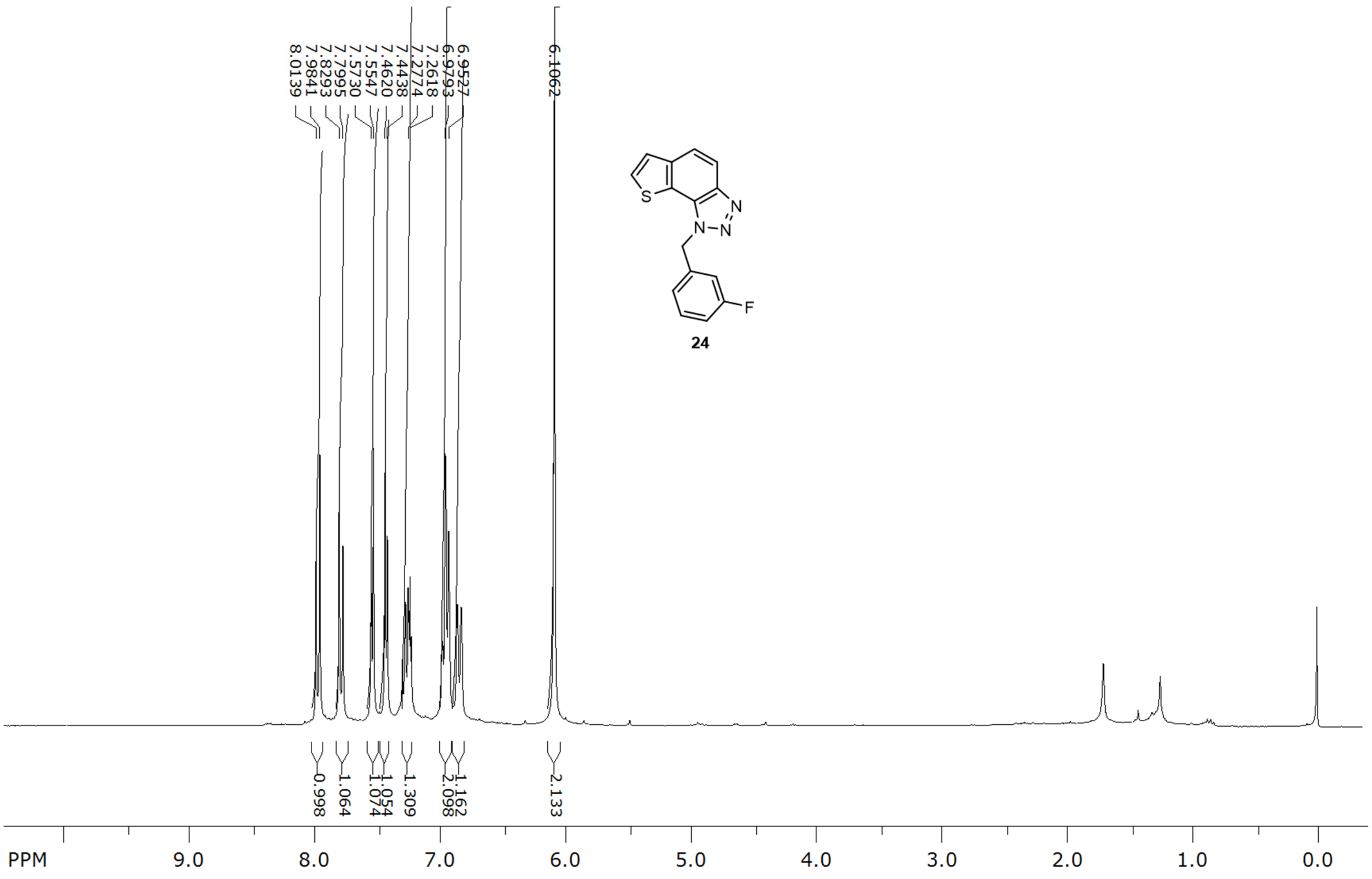

Figure S4. ^{1}H NMR ($CDCl_3$) spectrum of **24**.

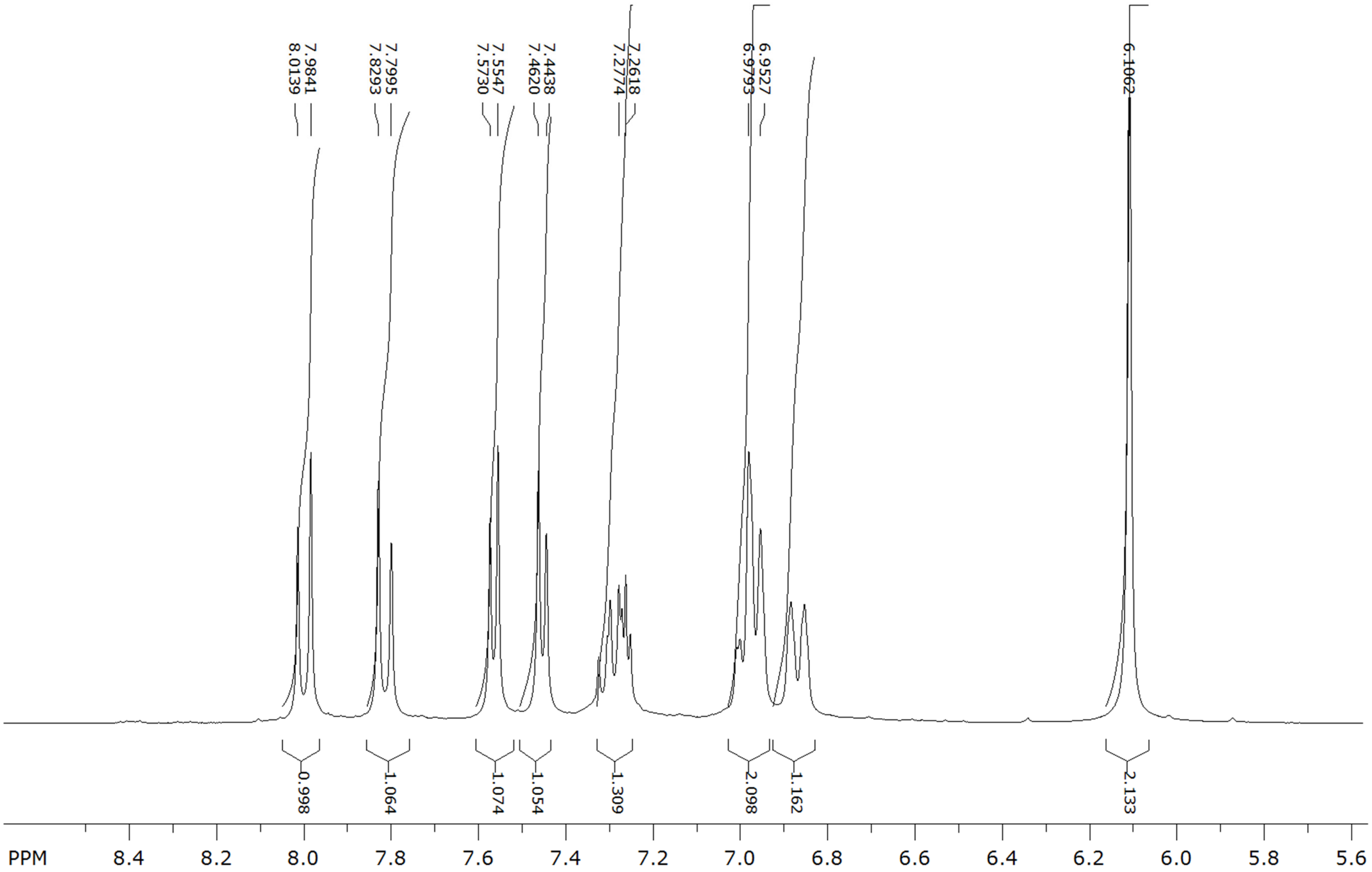

Figure S5. ^{1}H NMR ($CDCl_3$) spectrum of aromatic part of **24**.

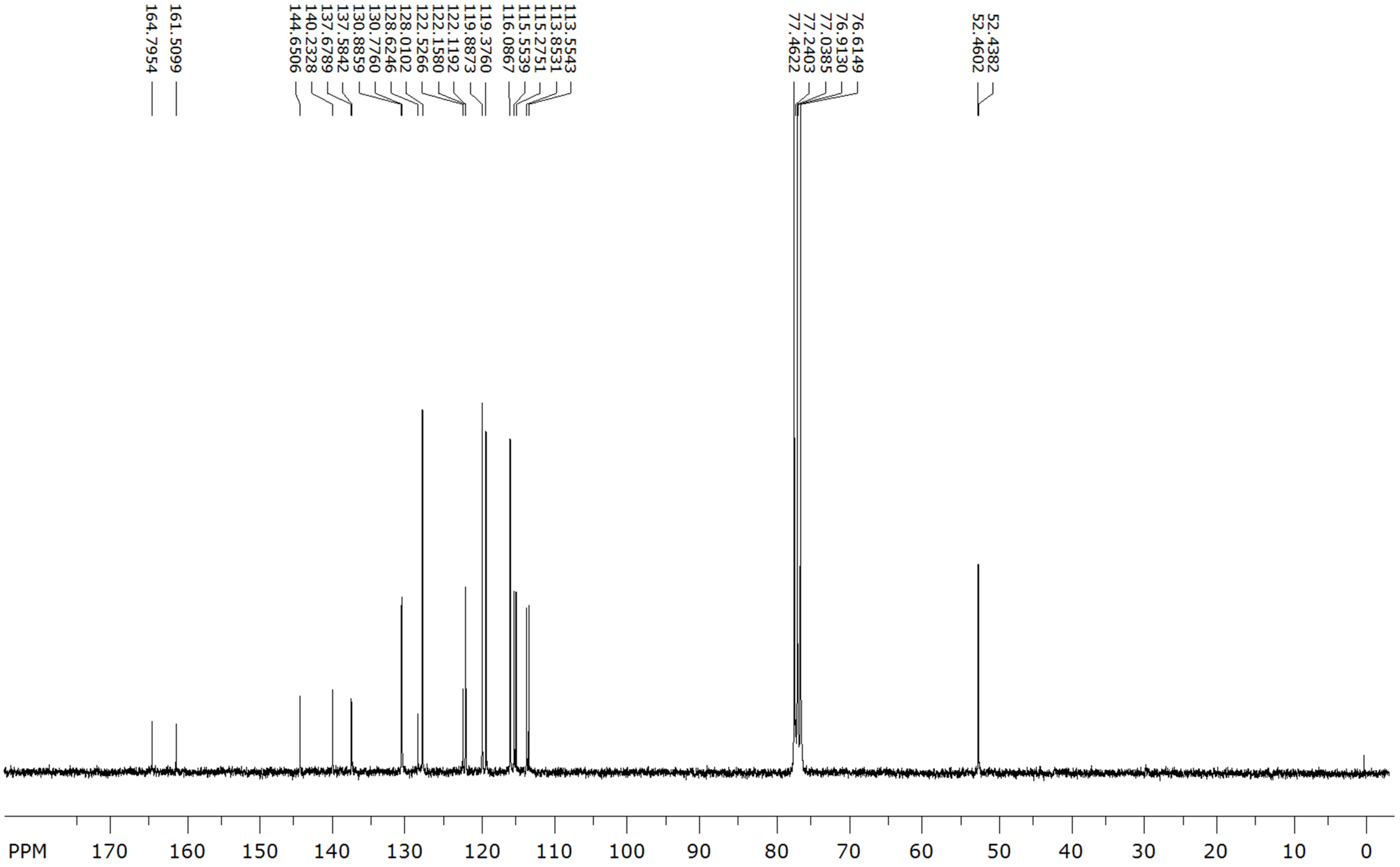

Figure S6. ^{13}C NMR ($CDCl_3$) spectrum of **24**.

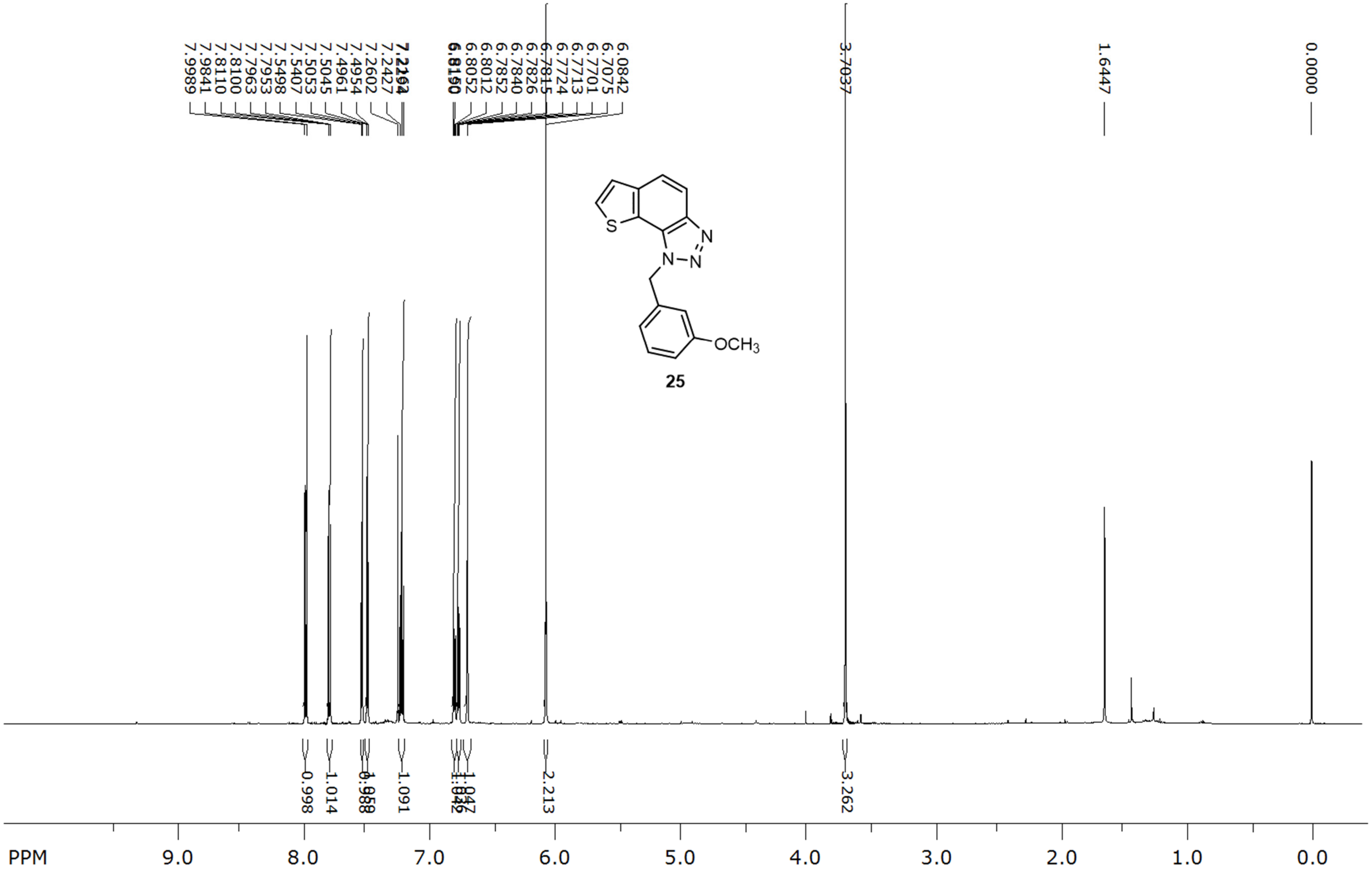

Figure S7. ^{1}H NMR ($CDCl_3$) spectrum of **25**.

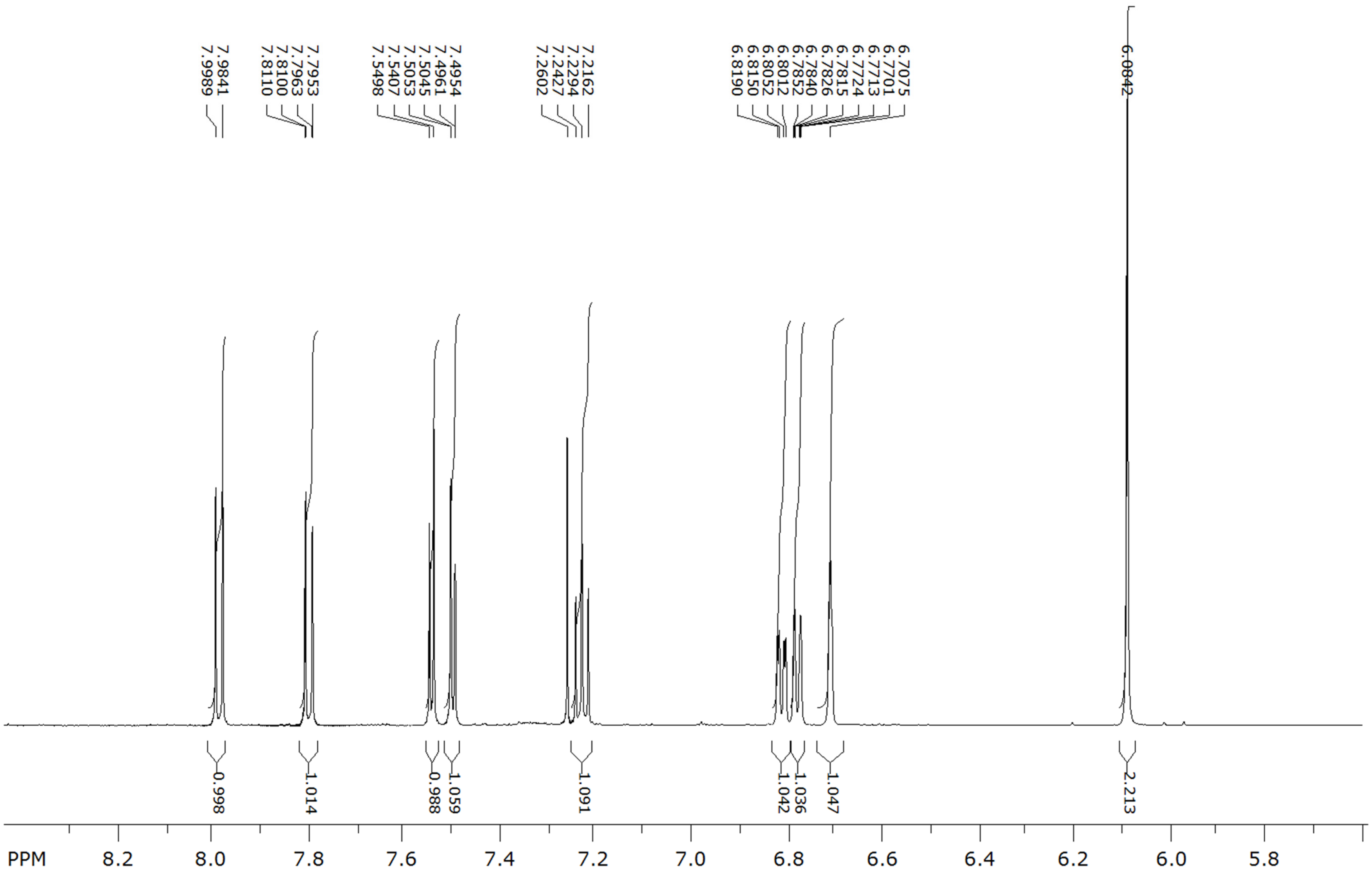

Figure S8. ^{1}H NMR ($CDCl_3$) spectrum of aromatic part of **25**.

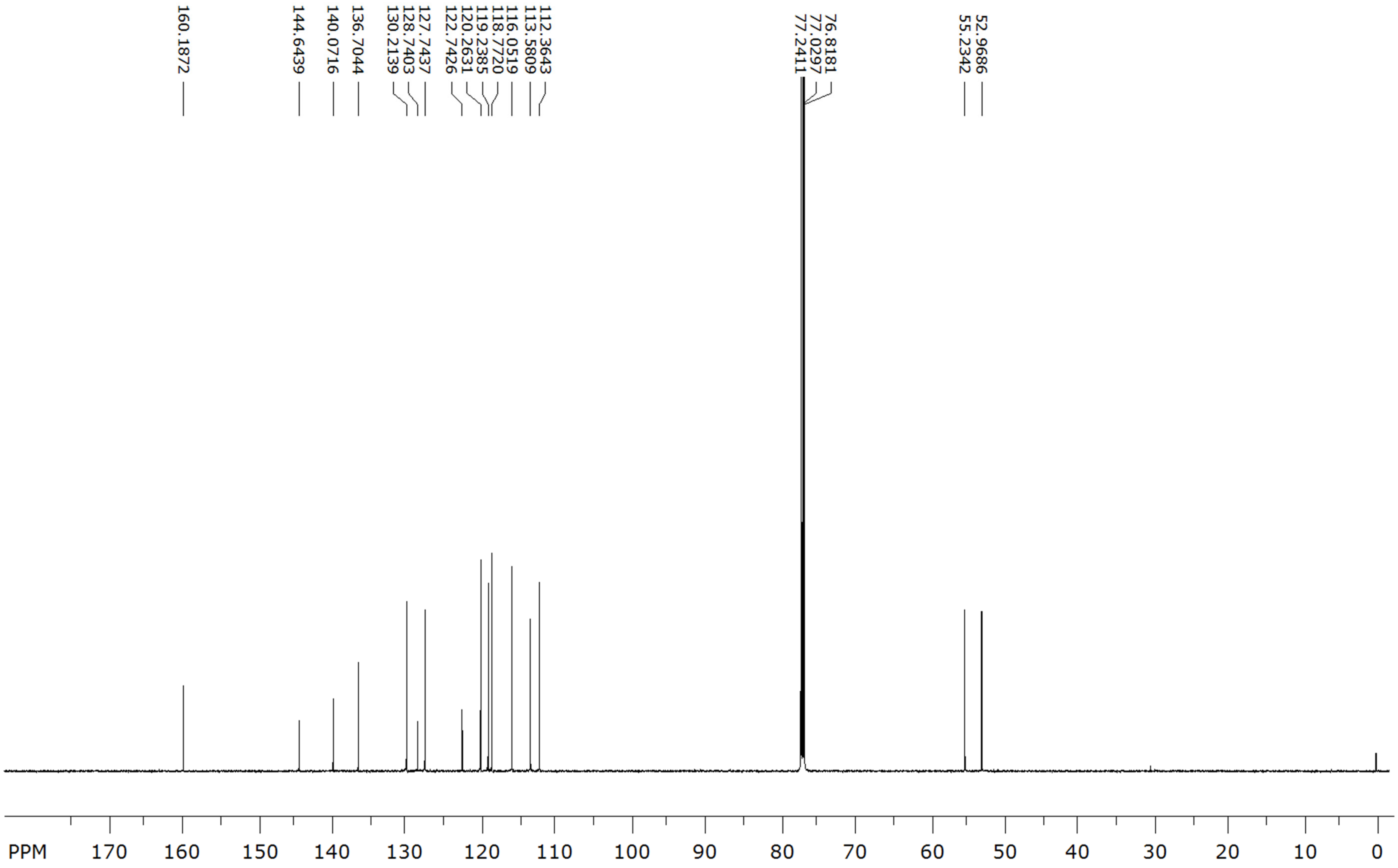

Figure S9. ^{13}C NMR ($CDCl_3$) spectrum of **25**.

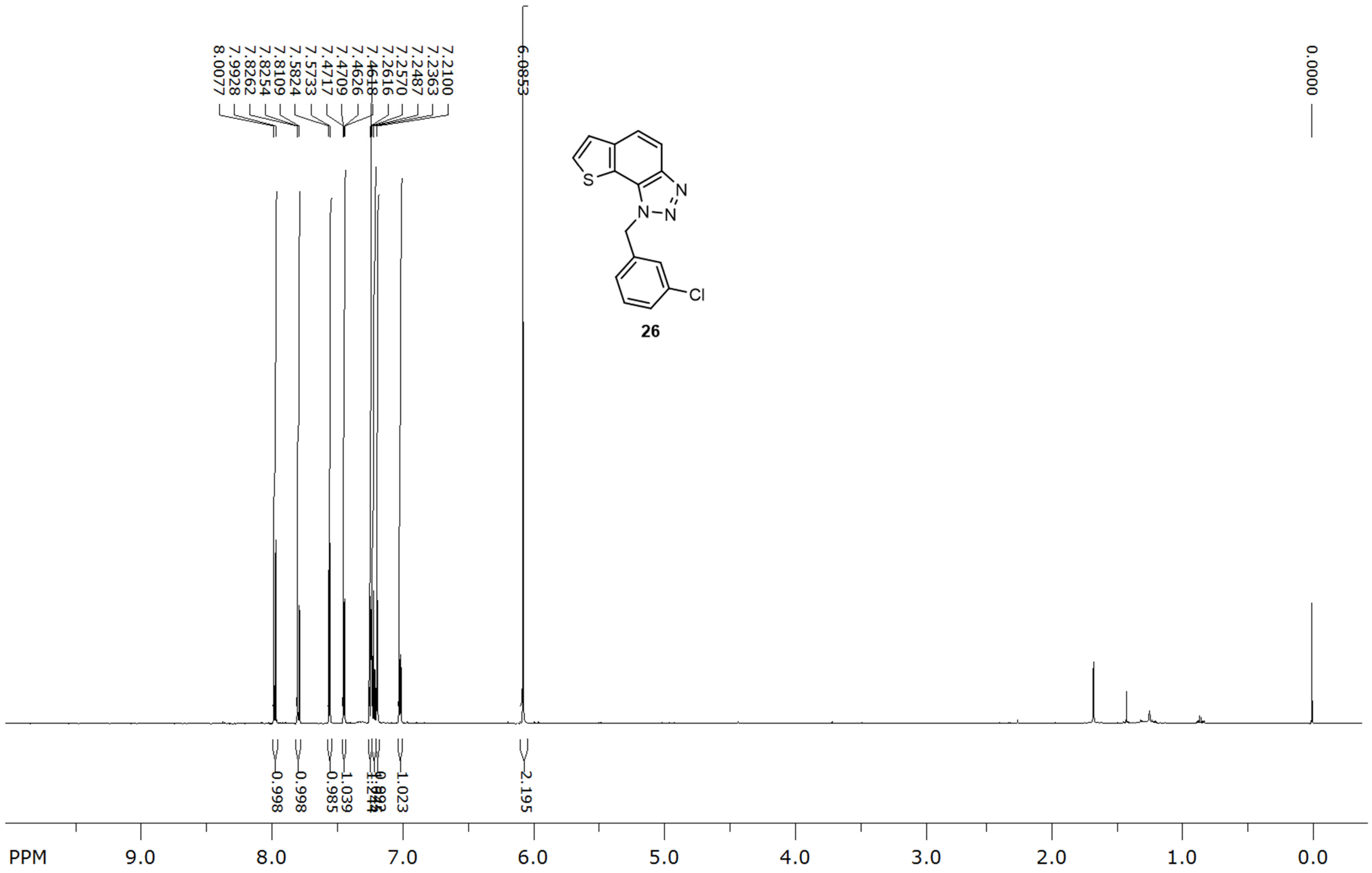

Figure S10. ^{1}H NMR ($CDCl_3$) spectrum of **26**.

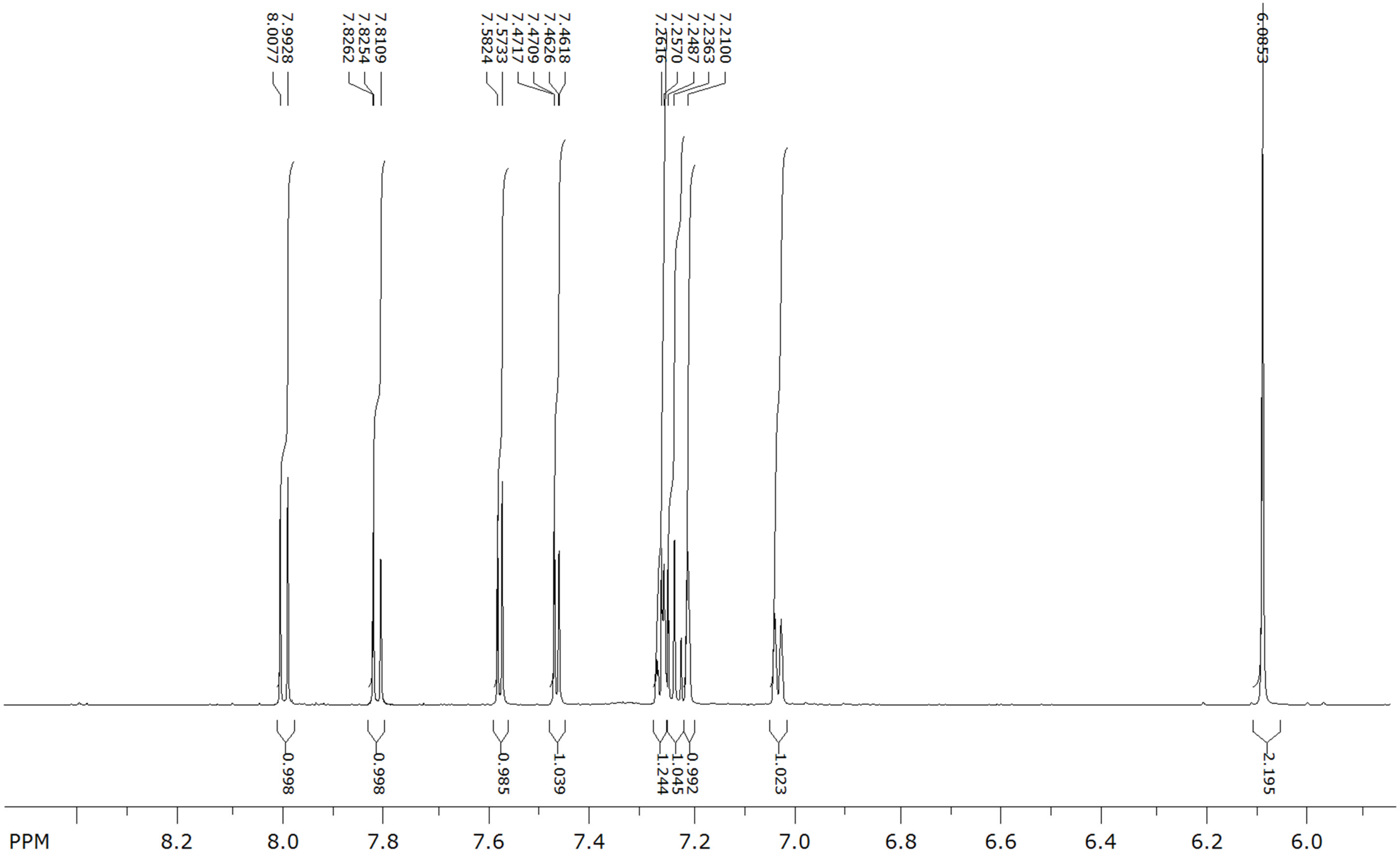

Figure S11. ^{1}H NMR ($CDCl_3$) spectrum of aromatic part of **26**.

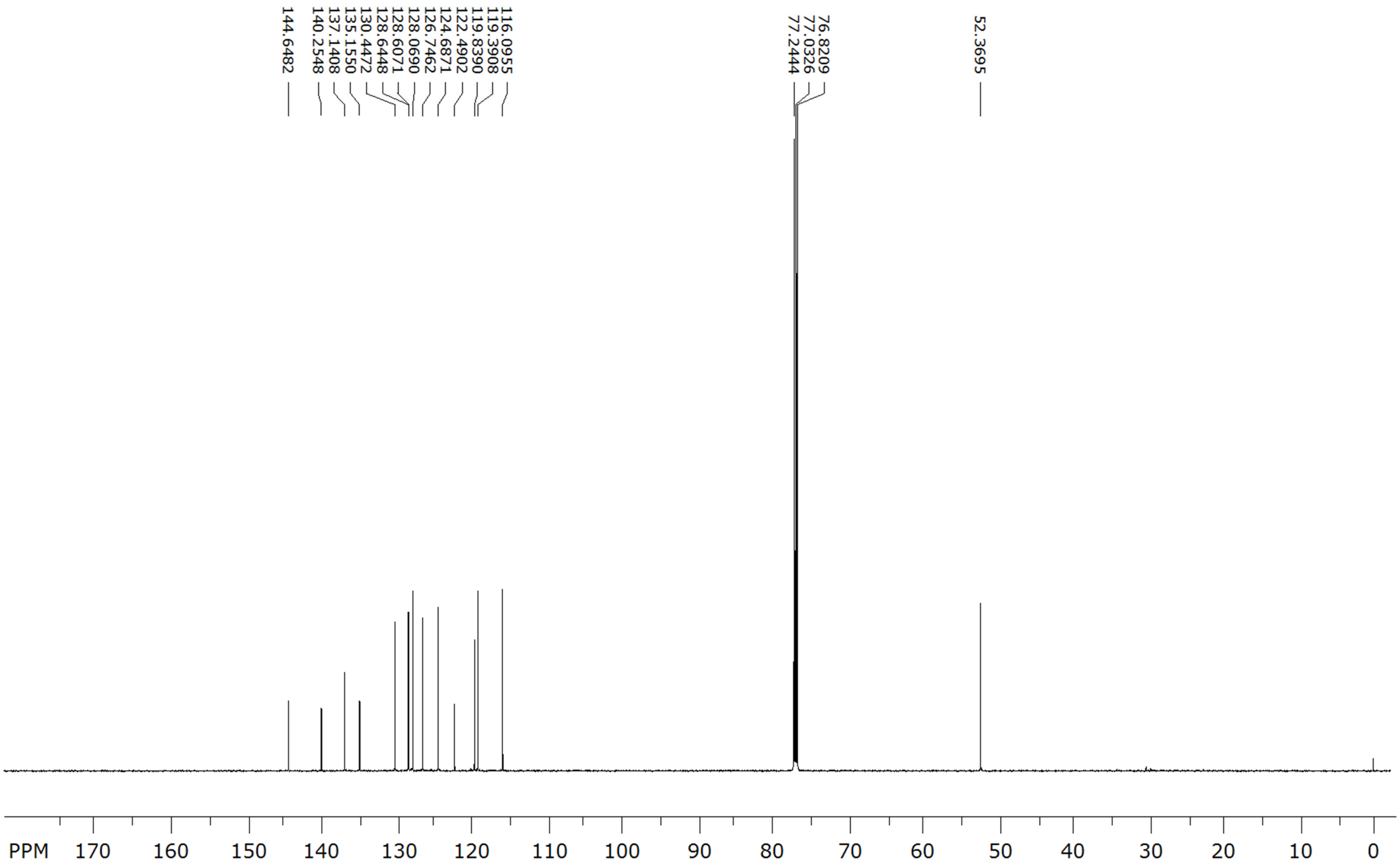

Figure S12. ^{13}C NMR ($CDCl_3$) spectrum of **26**.

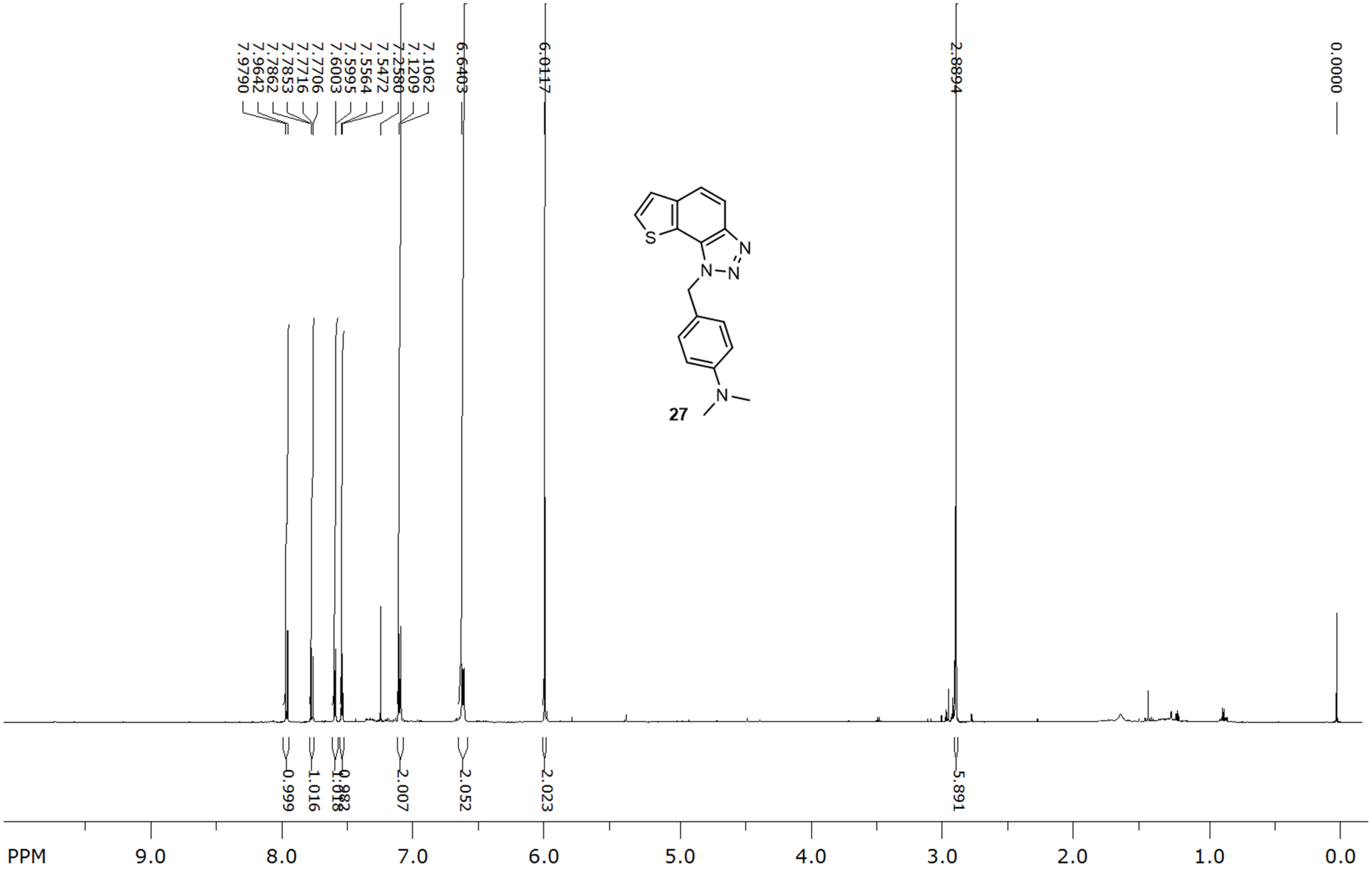

Figure S13. ^{1}H NMR ($CDCl_3$) spectrum of **27**.

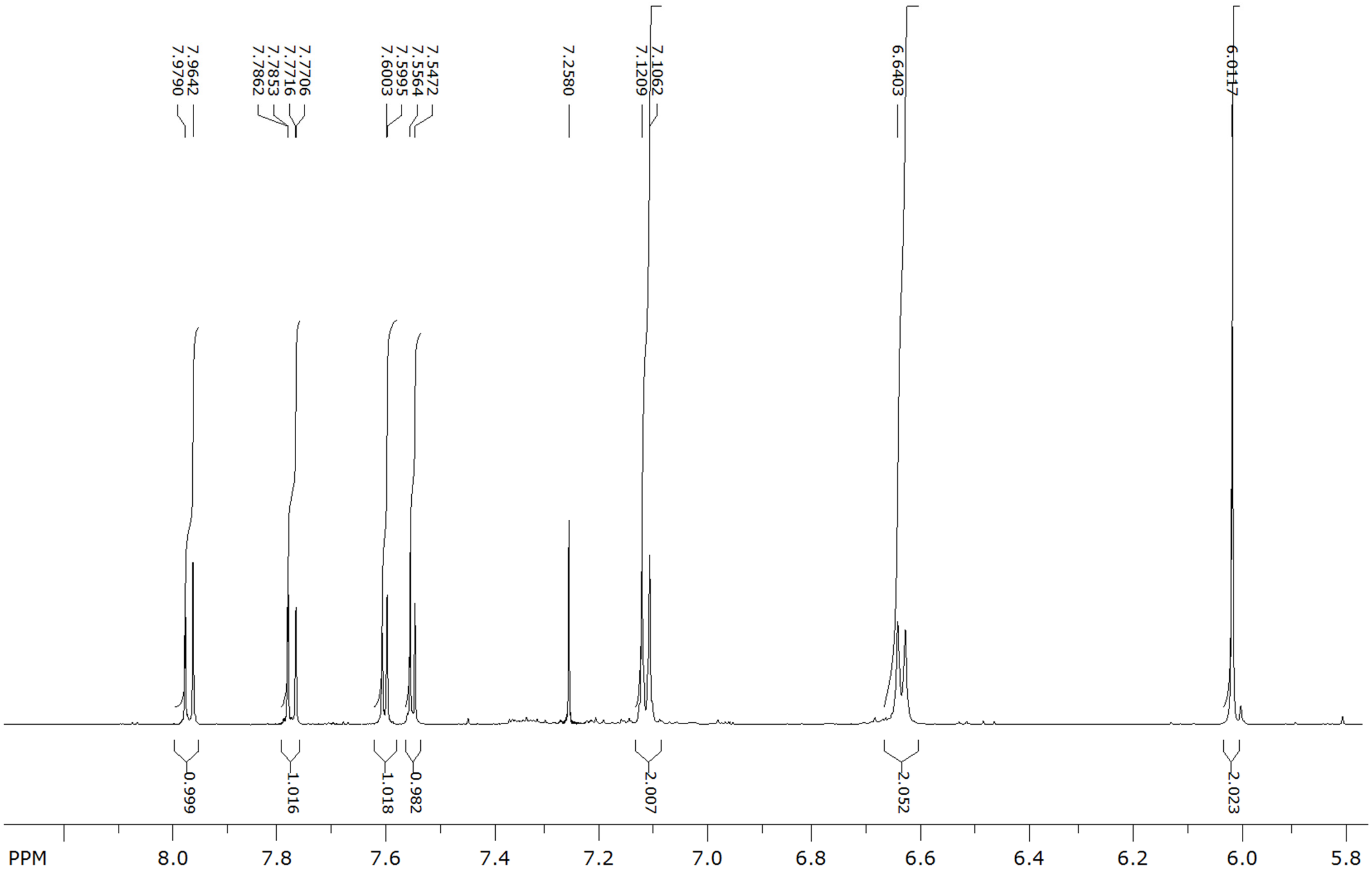

Figure S14. ^{1}H NMR ($CDCl_3$) spectrum of aromatic part of **27**.

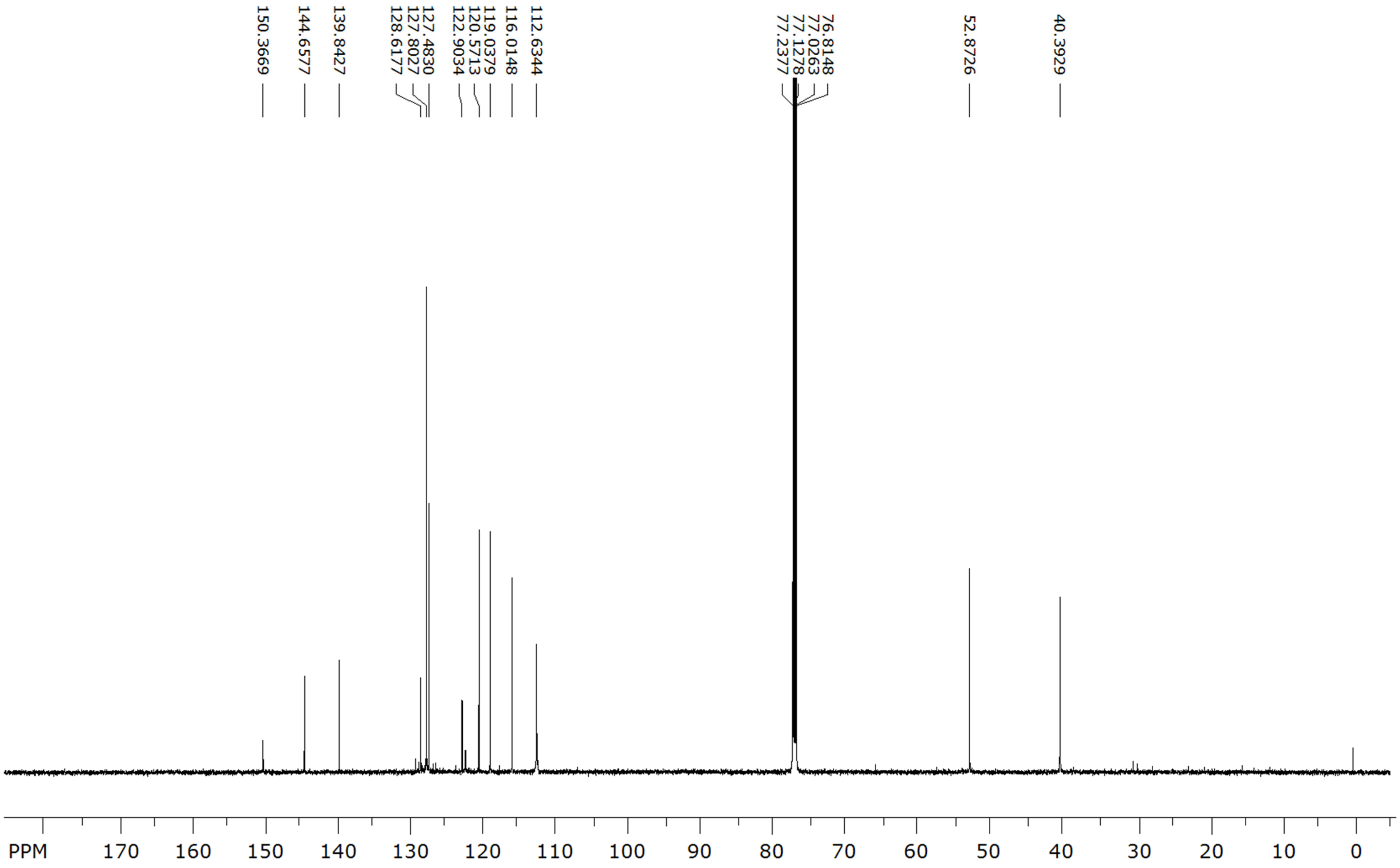

Figure S15. ^{13}C NMR ($CDCl_3$) spectrum of **27**.

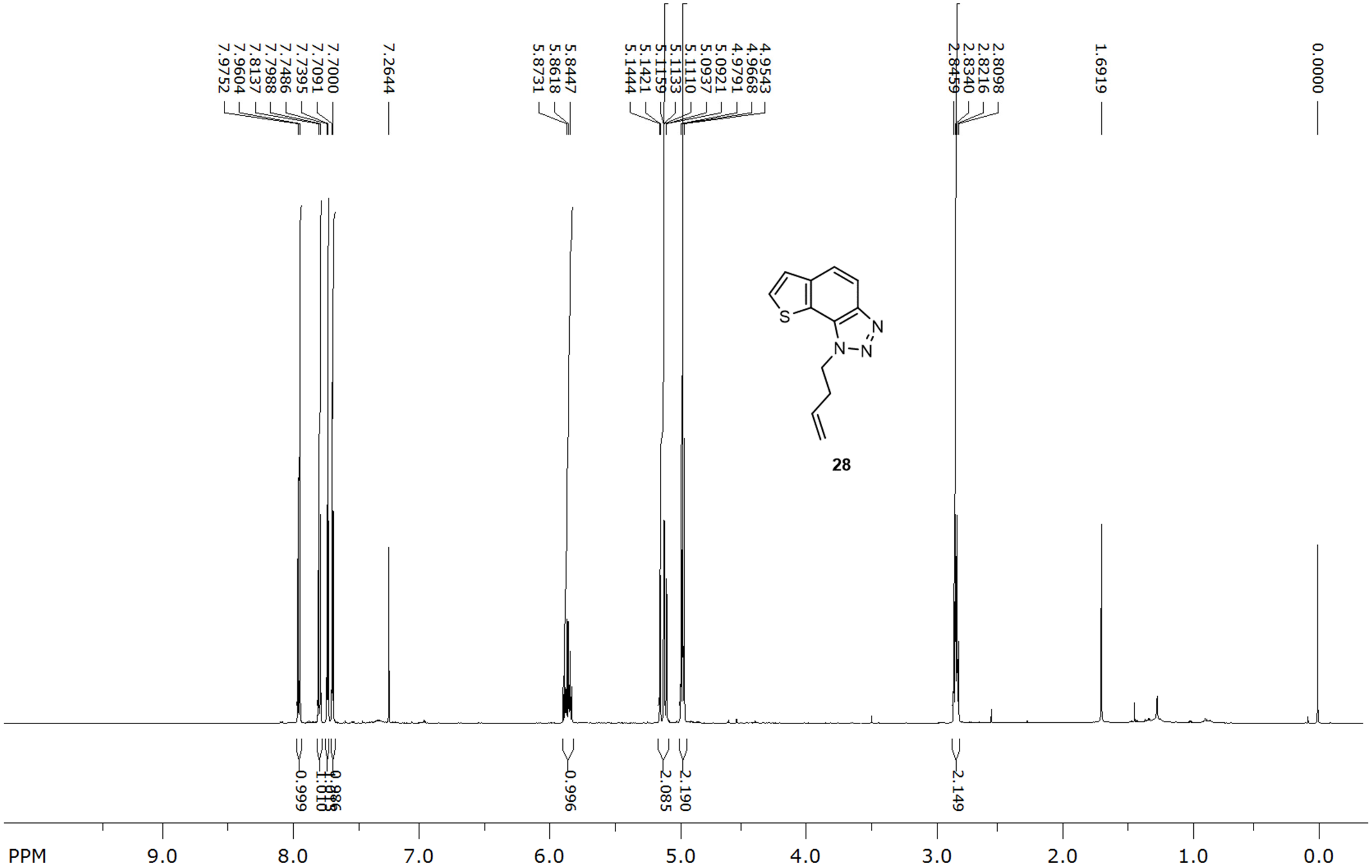

Figure S16. ^{1}H NMR ($CDCl_3$) spectrum of **28**.

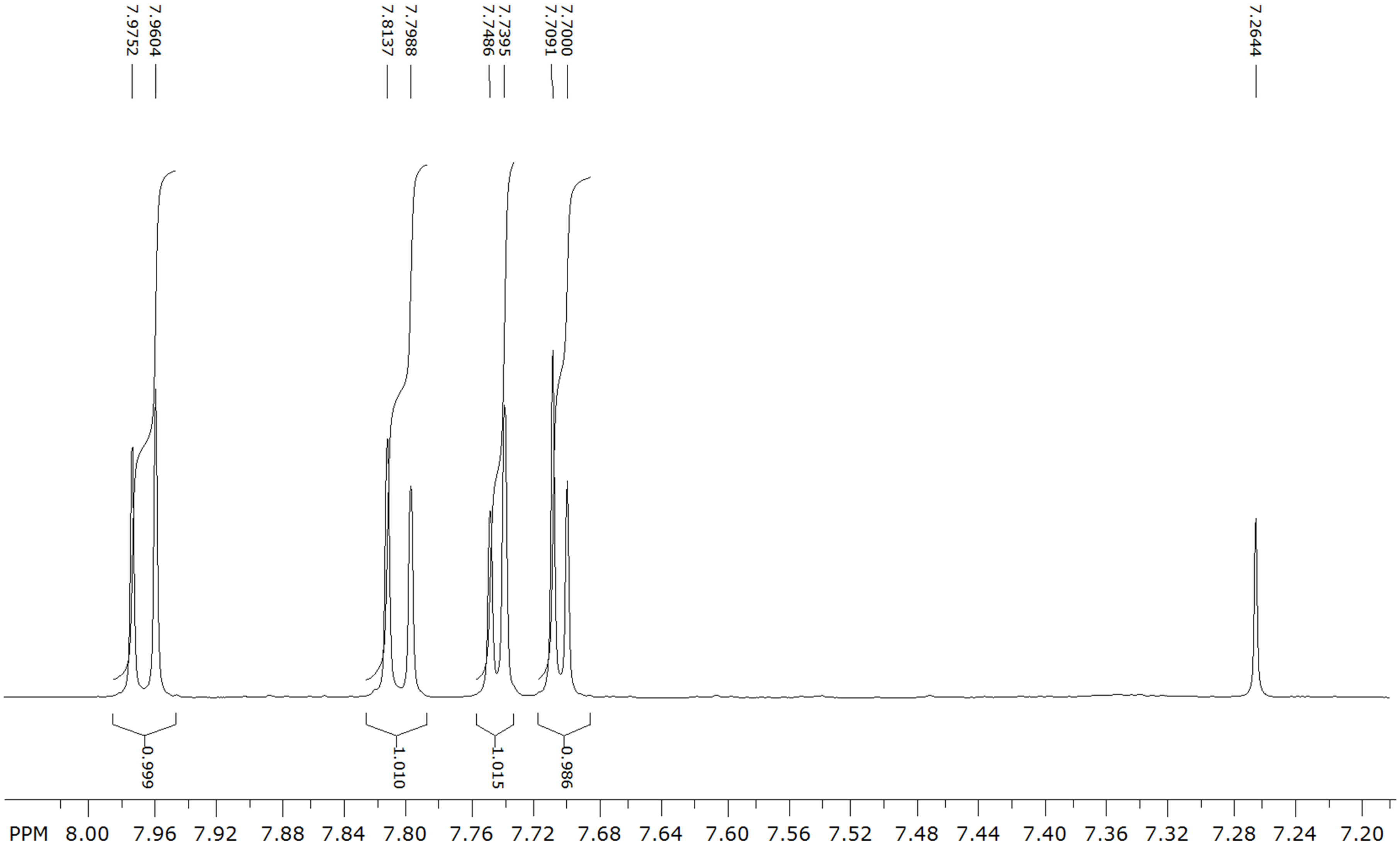

Figure S17. ^{1}H NMR ($CDCl_3$) spectrum of aromatic part of **28**.

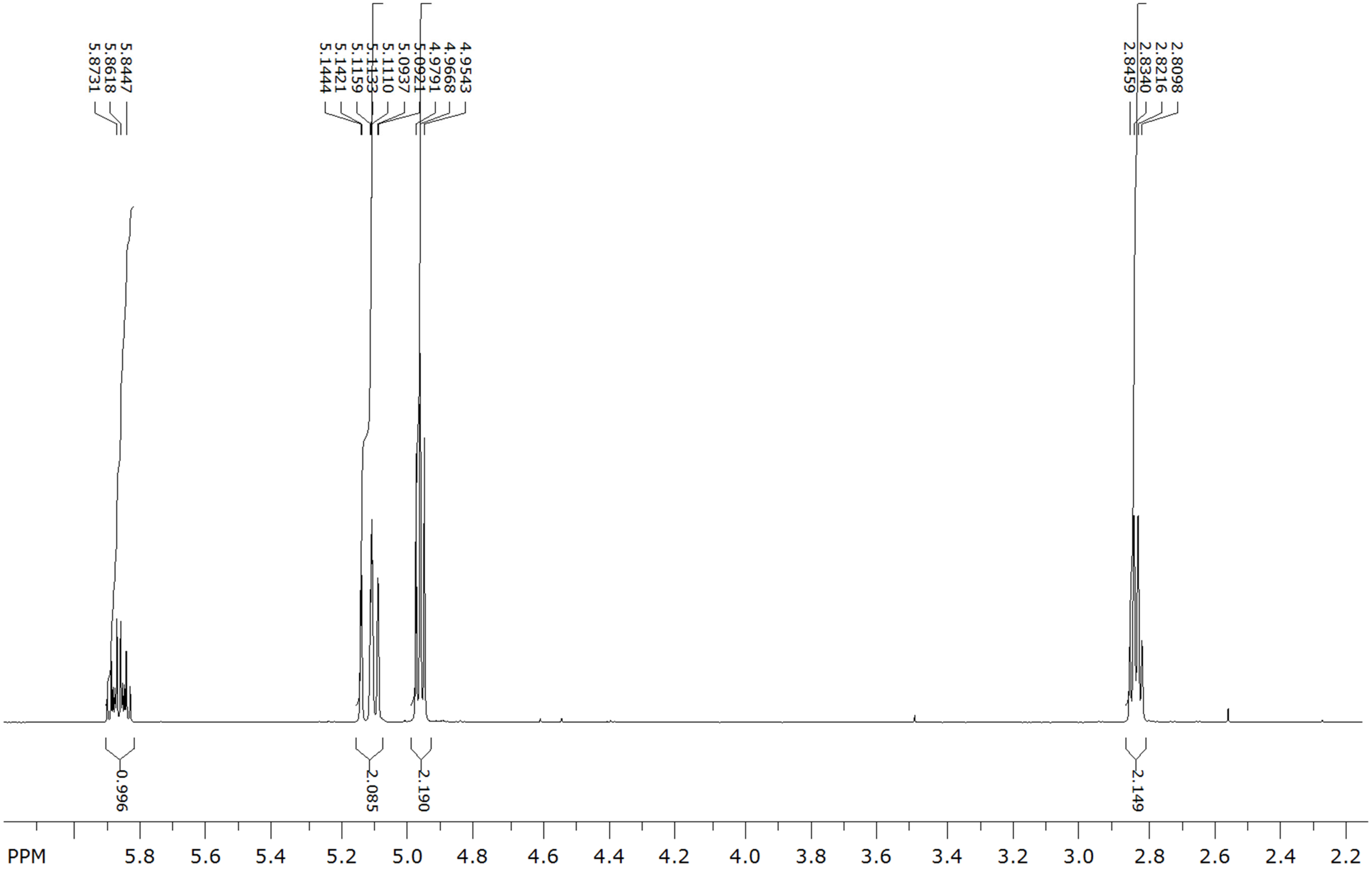

Figure S18. ^{1}H NMR ($CDCl_3$) spectrum of aliphatic part of **28**.

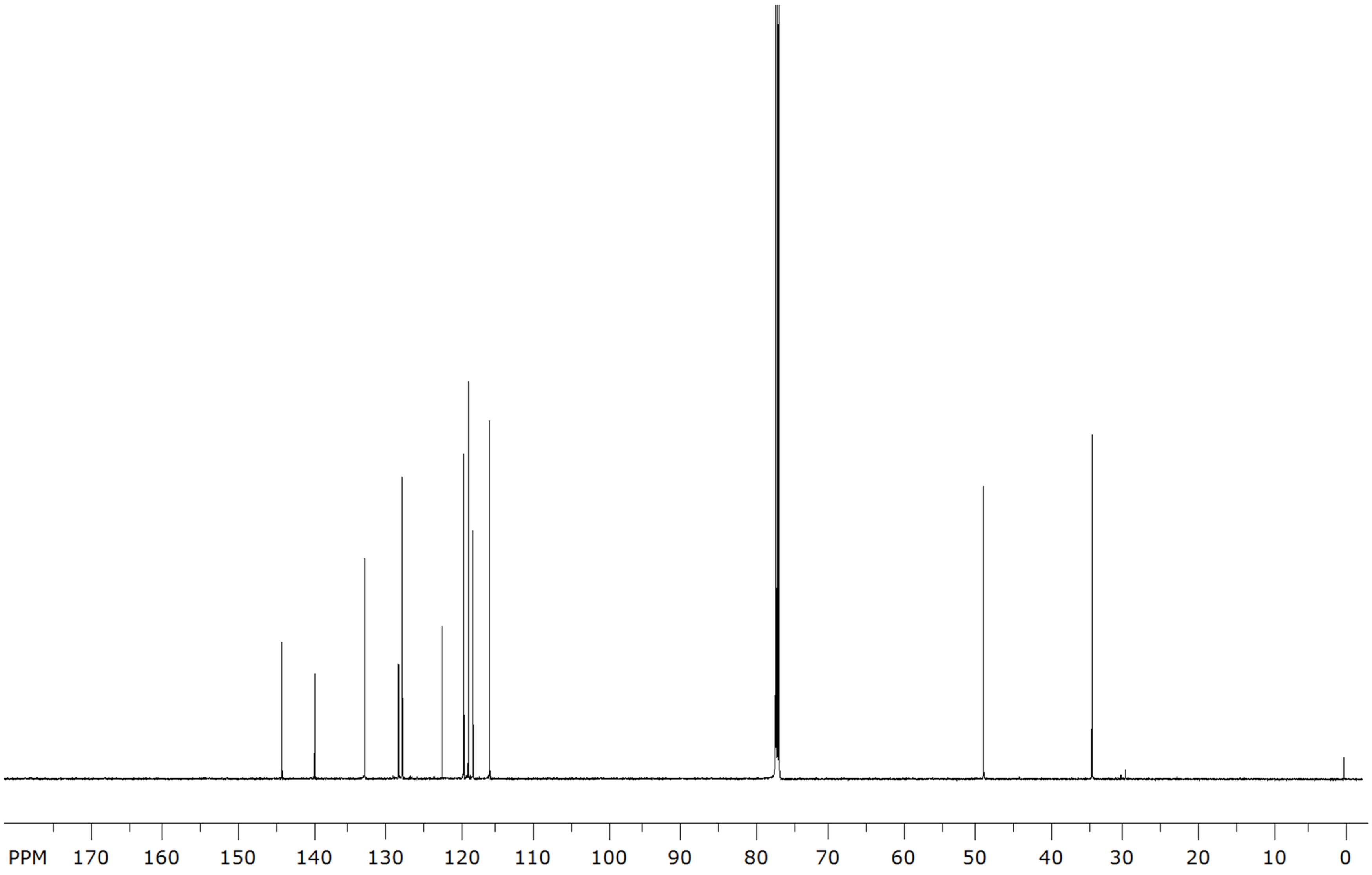

Figure S19. ^{13}C NMR ($CDCl_3$) spectrum of **28** (*Spin Works 3.0*).

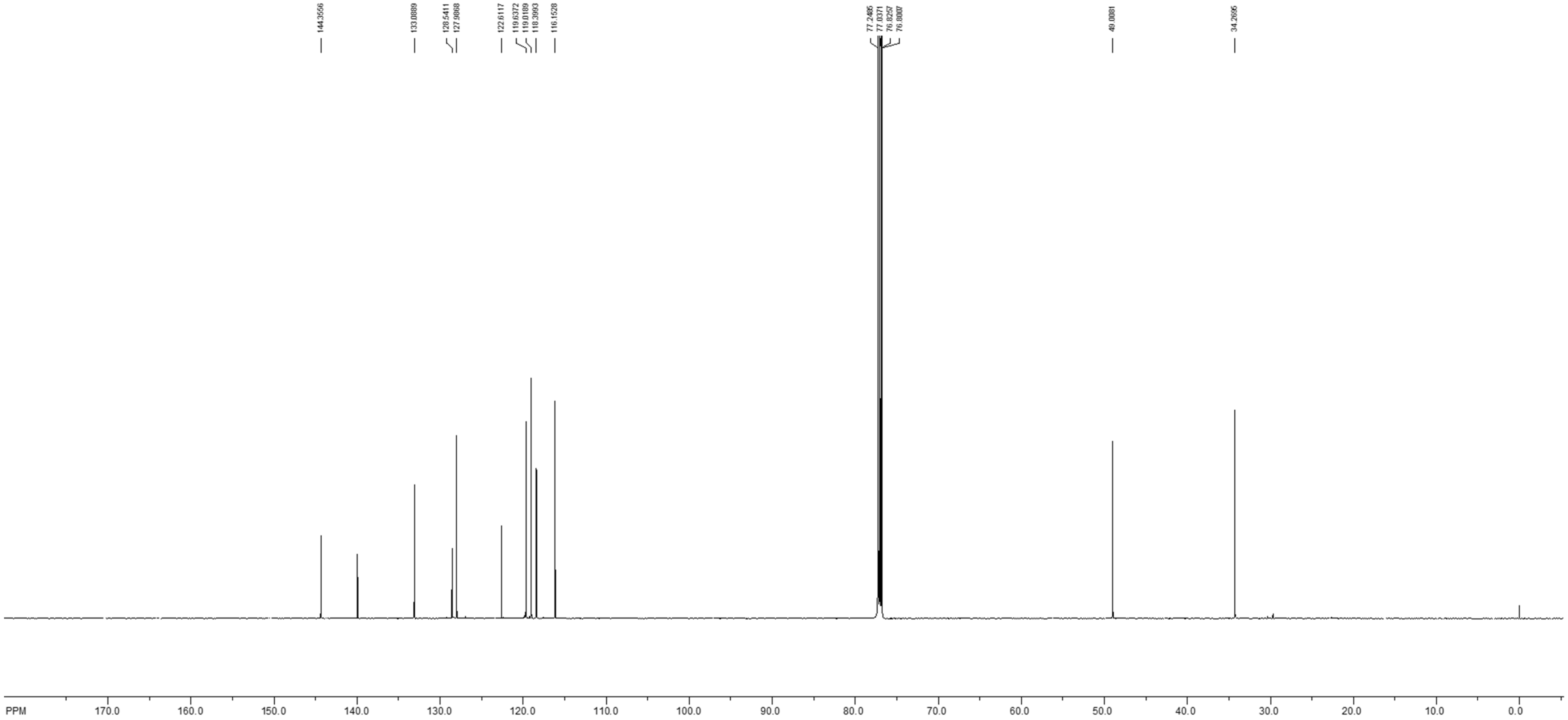

Figure S20. ^{13}C NMR ($CDCl_3$) spectrum of **28** (*Spin Works 2.5.5.*).

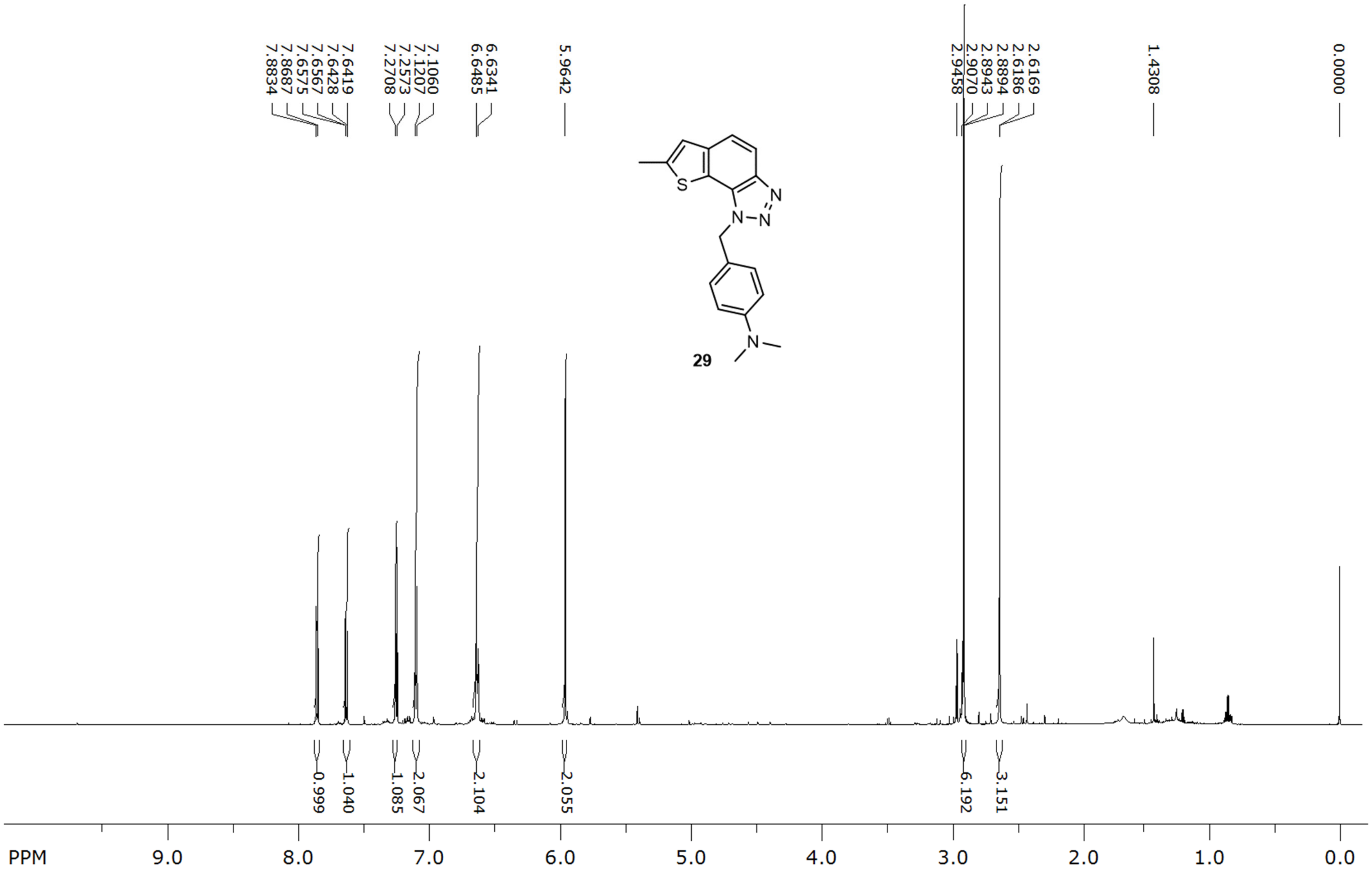

Figure S21. ^{1}H NMR ($CDCl_3$) spectrum of **29**.

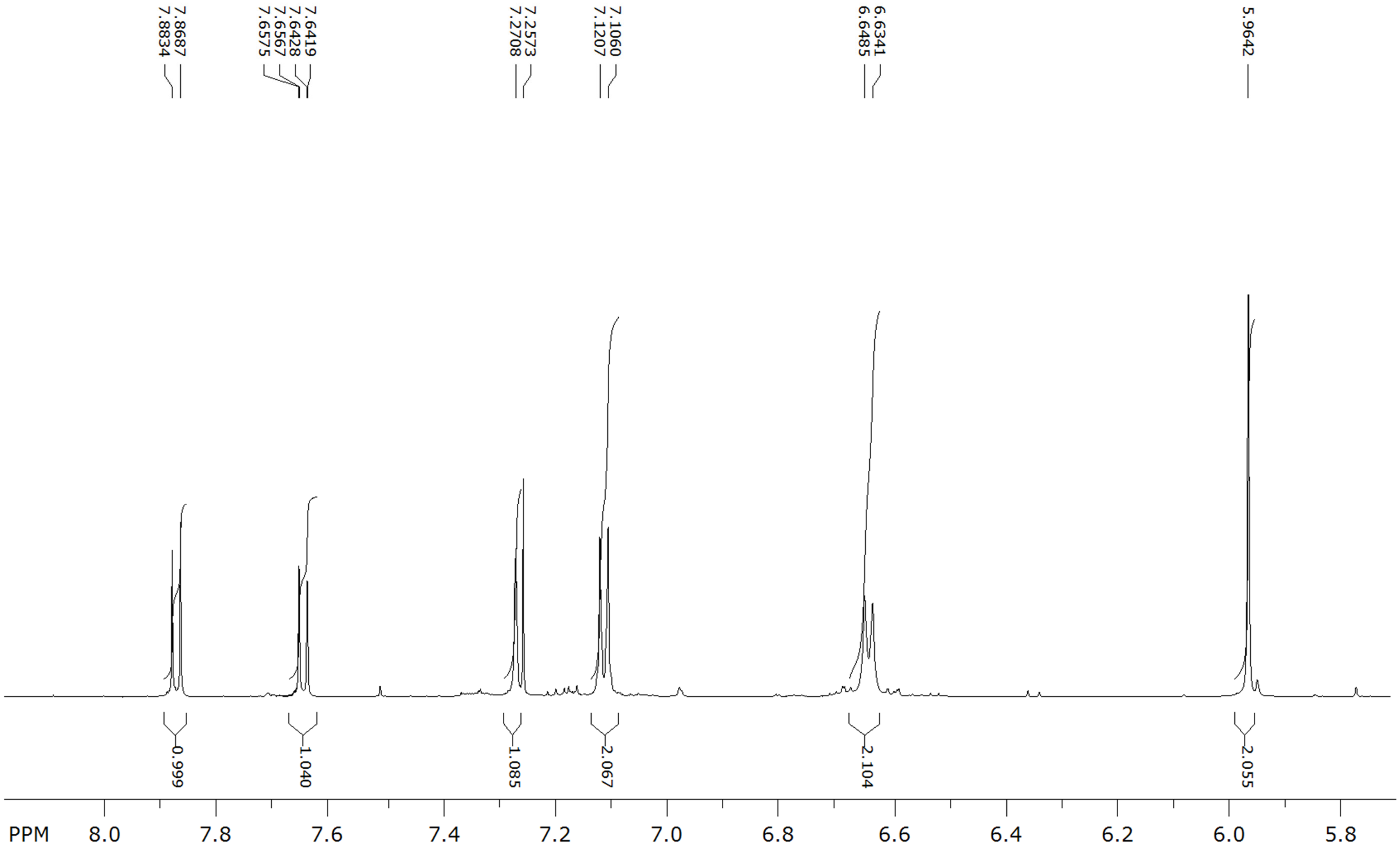

Figure S22. ^{1}H NMR ($CDCl_3$) spectrum of aromatic part of **29**.

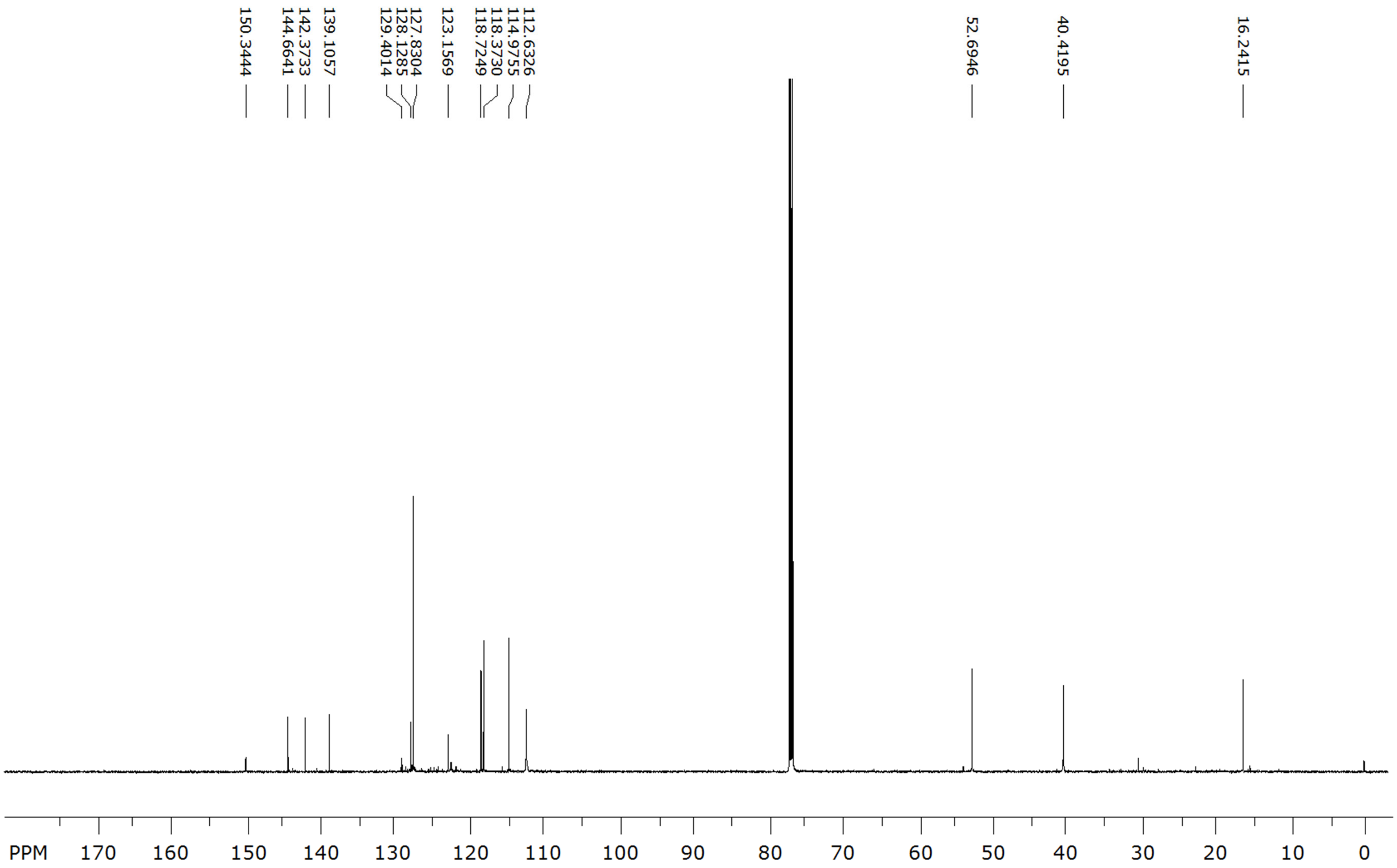

Figure S23. ^{13}C NMR ($CDCl_3$) spectrum of **29**.

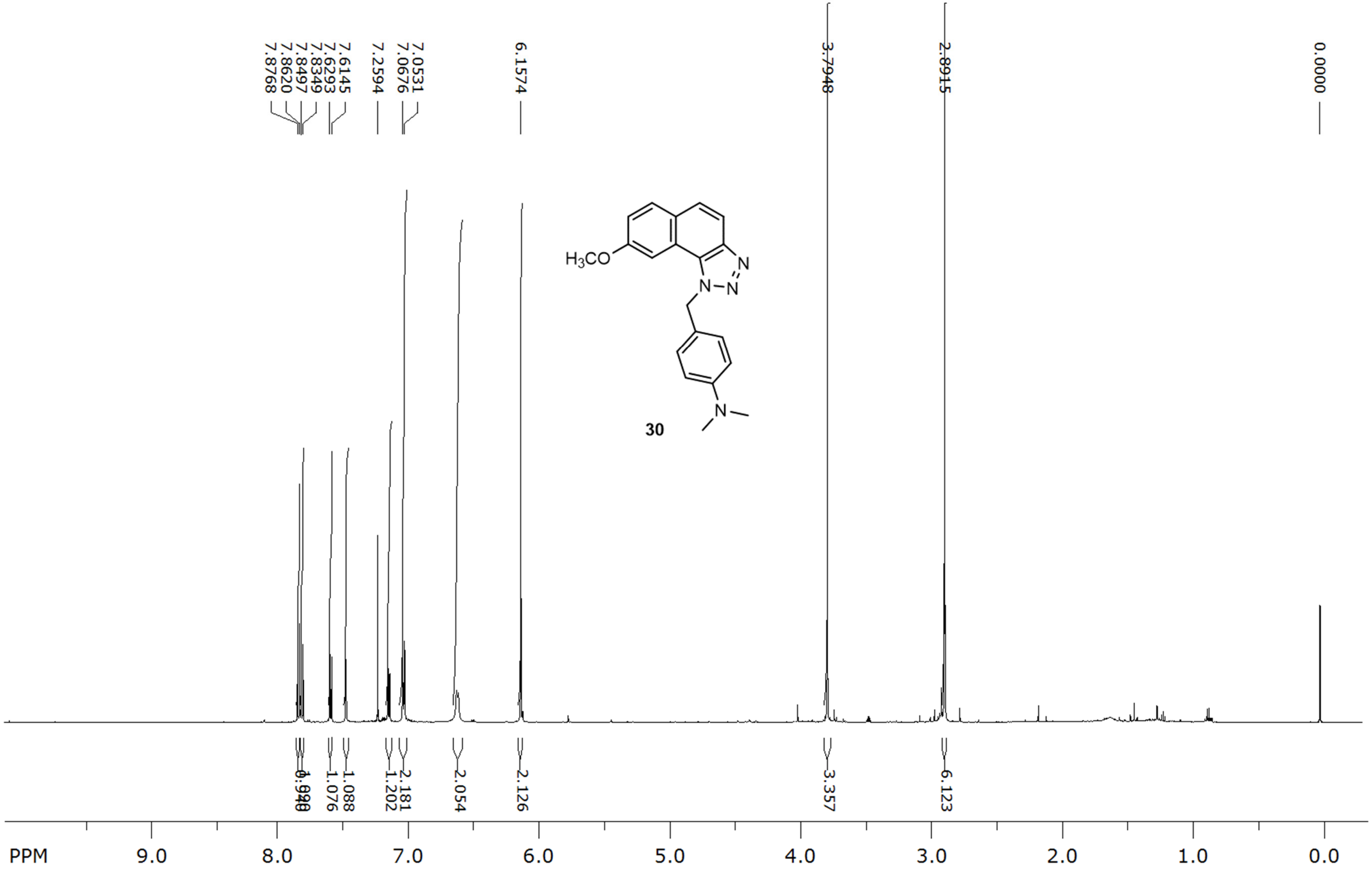

Figure S24. ^{1}H NMR ($CDCl_3$) spectrum of **30**.

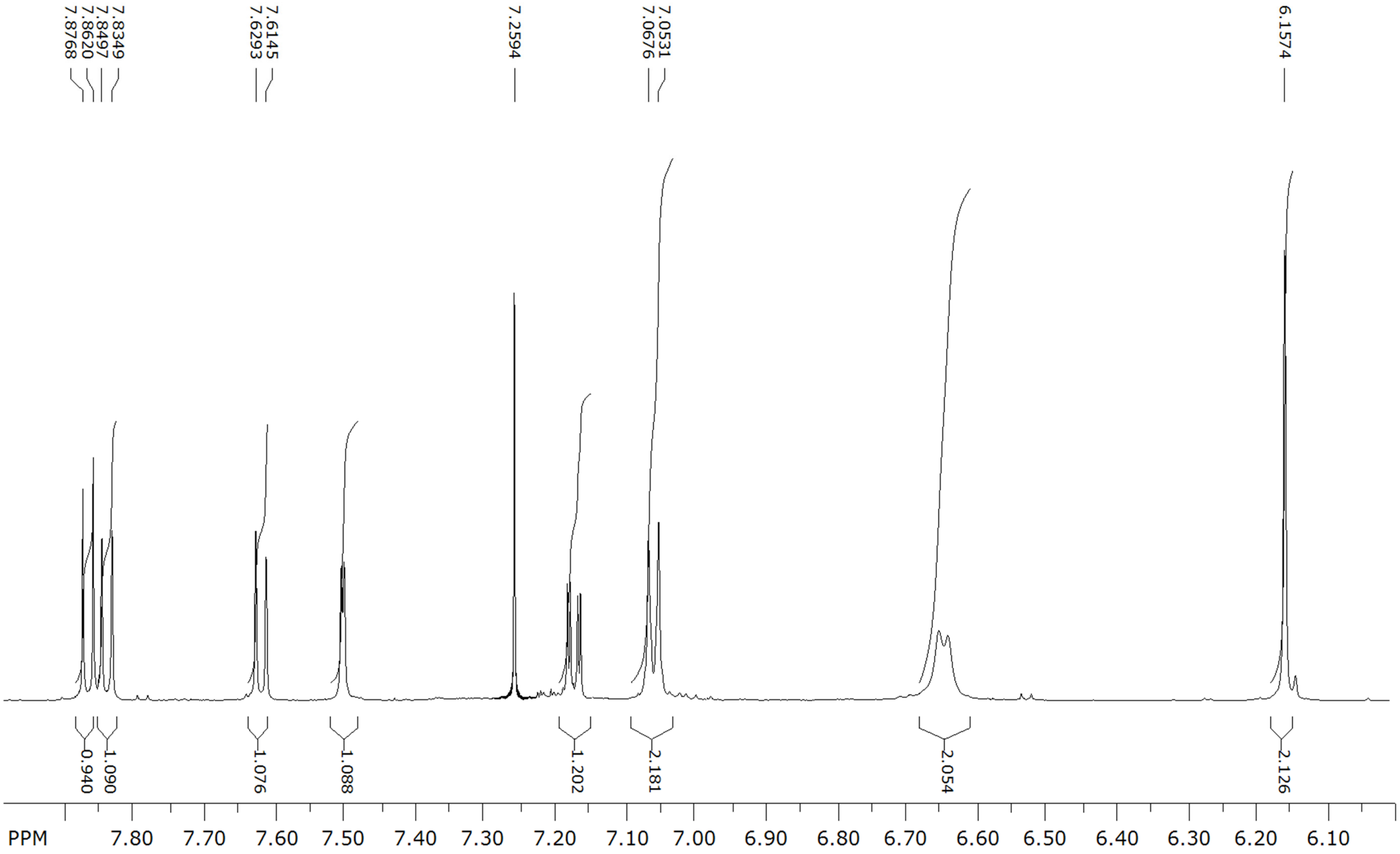

Figure S25. ^{1}H NMR ($CDCl_3$) spectrum of aromatic part of **30**.

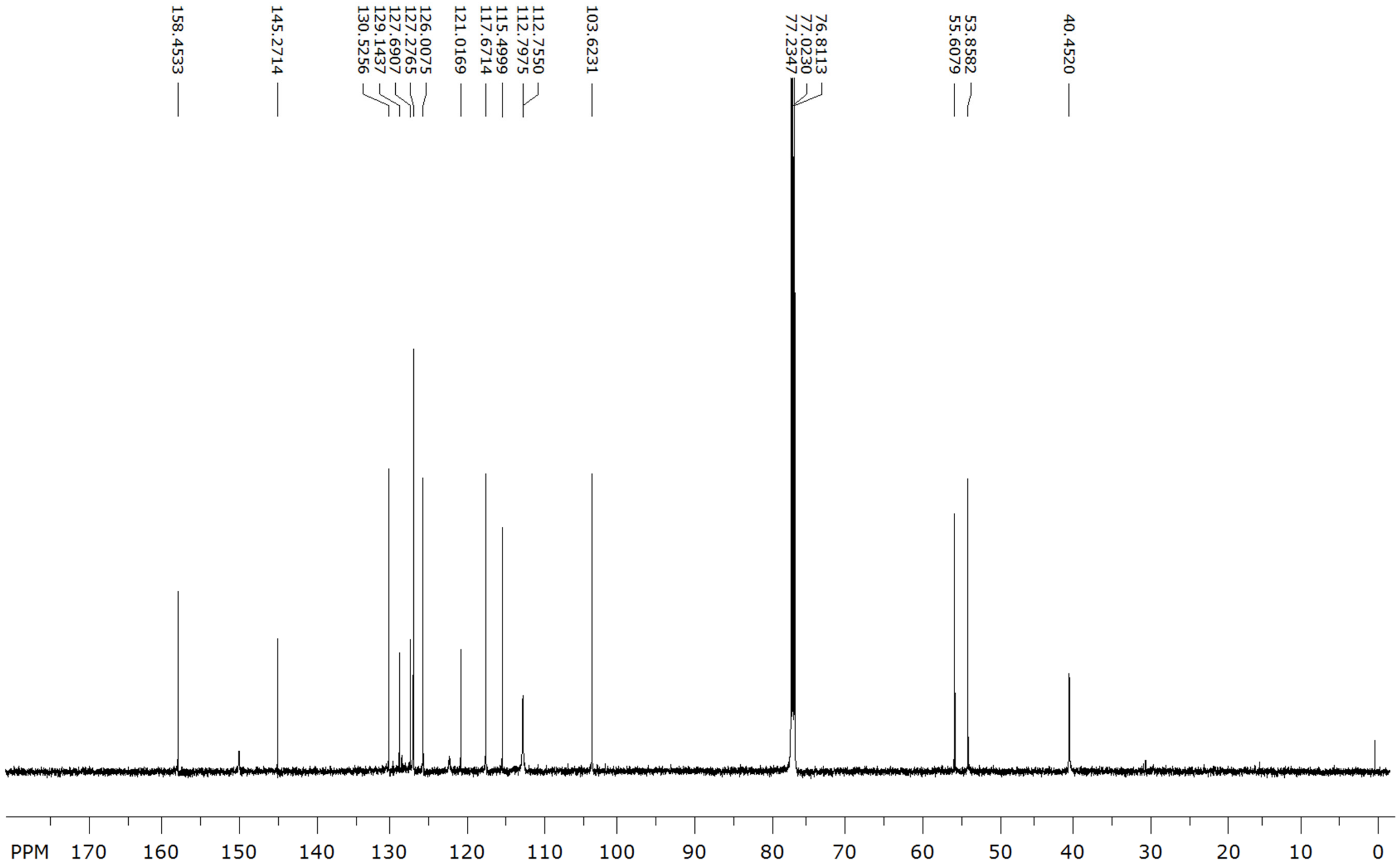

Figure S26. ^{13}C NMR ($CDCl_3$) spectrum of **30**.

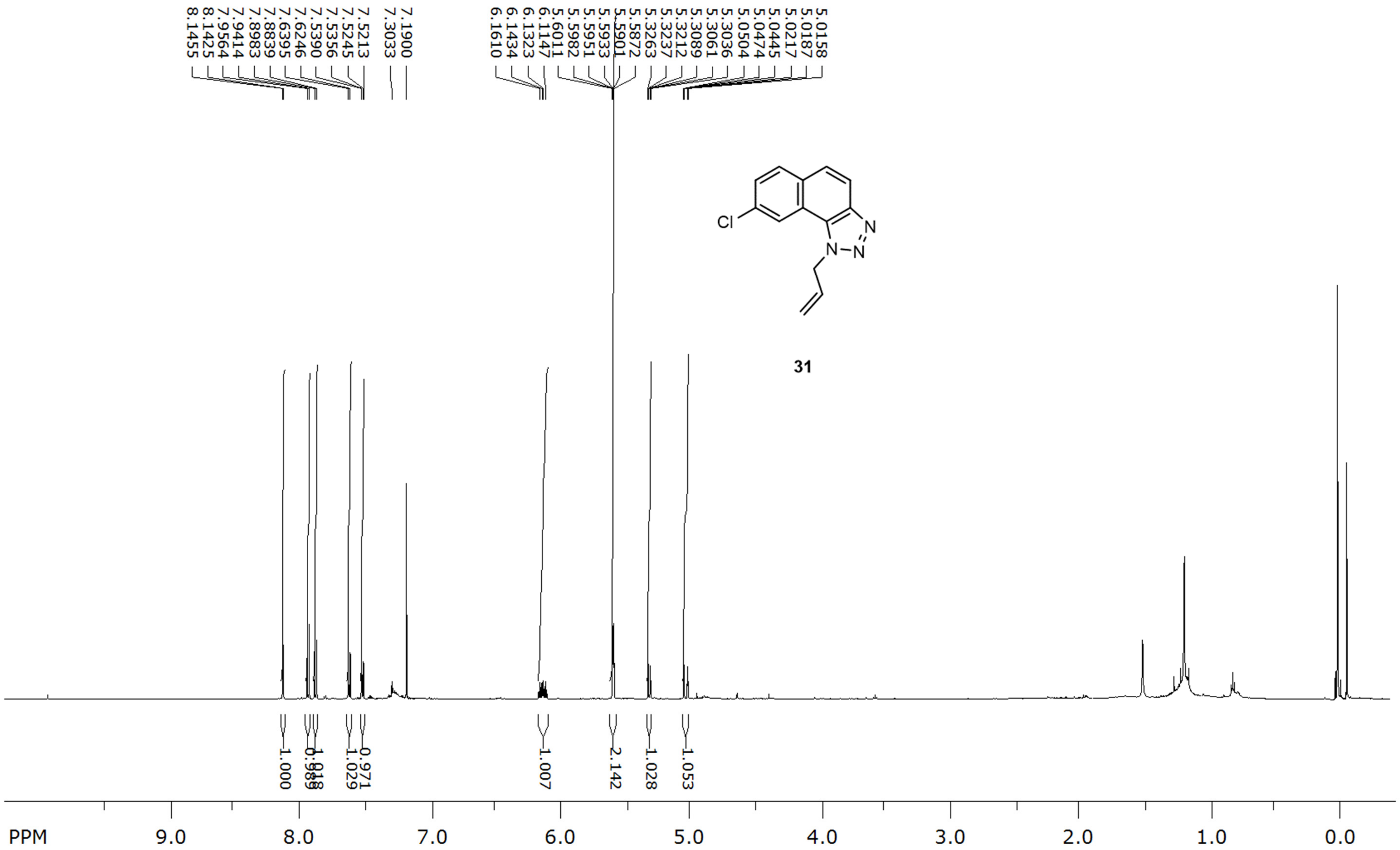

Figure S27. ^{1}H NMR ($CDCl_3$) spectrum of **31**.

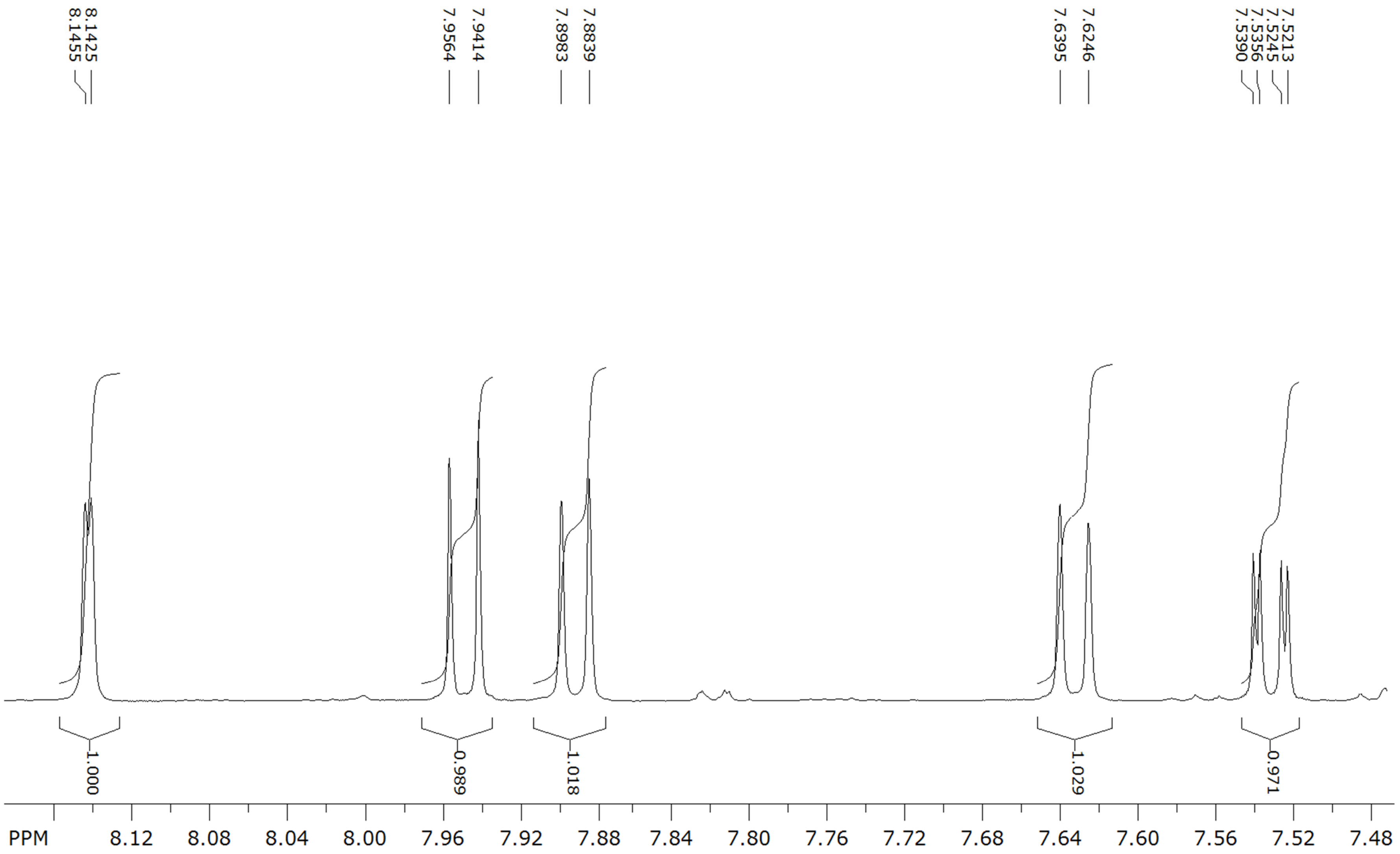

Figure S28. ^{1}H NMR ($CDCl_3$) spectrum of aromatic part of **31**.

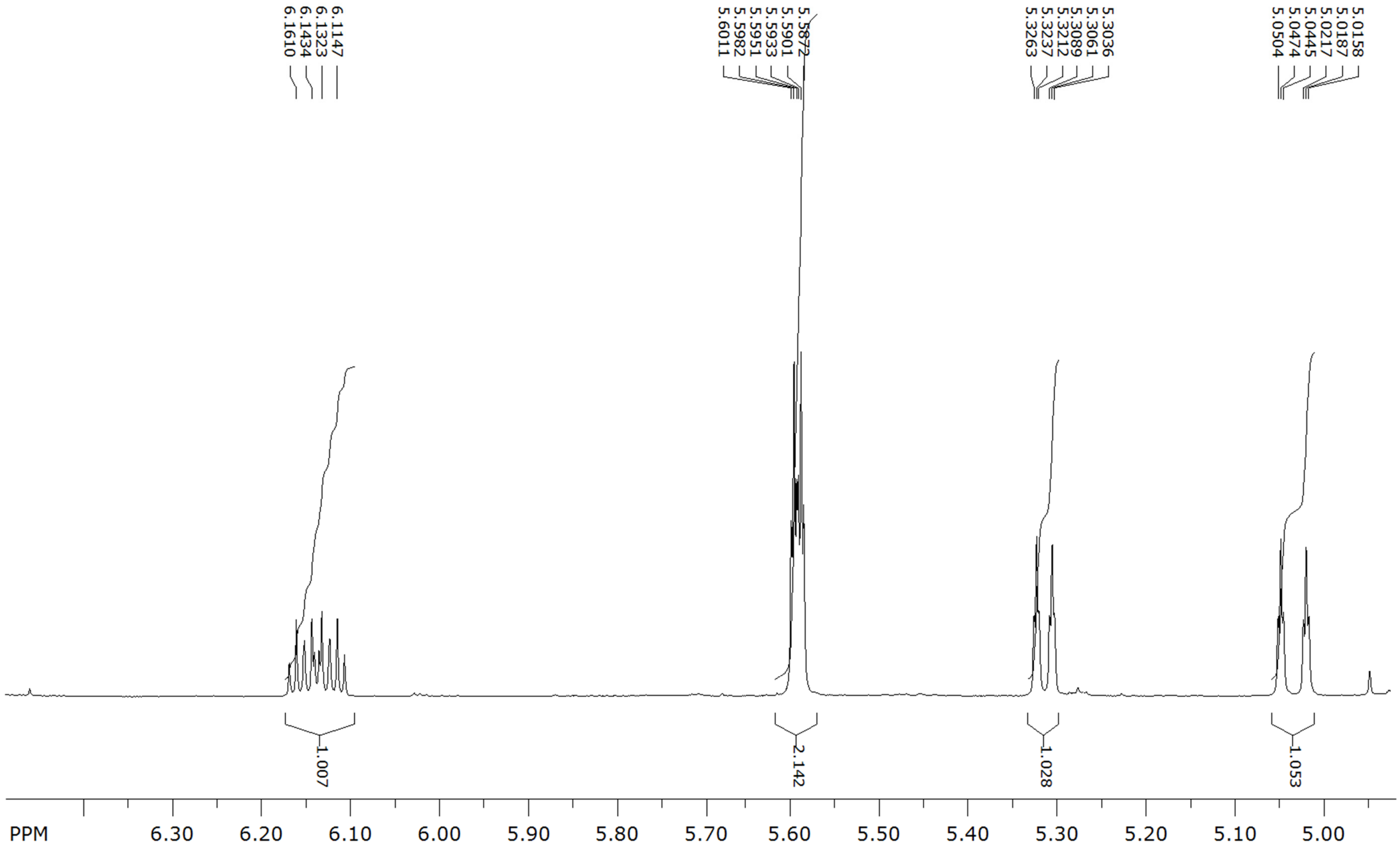

Figure S29. ^{1}H NMR ($CDCl_3$) spectrum of aliphatic part of **31**.

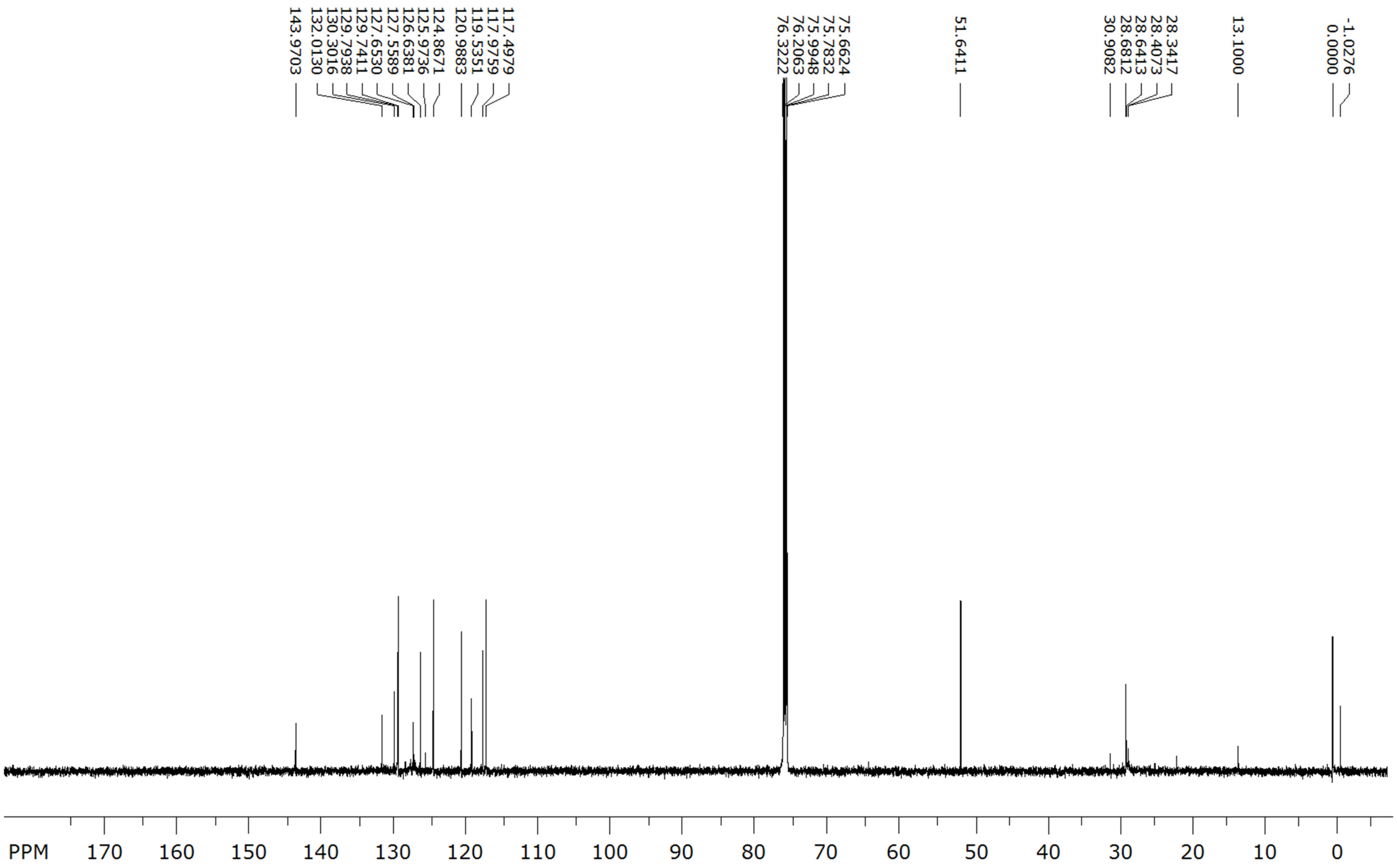

Figure S30. ^{13}C NMR ($CDCl_3$) spectrum of **31**.

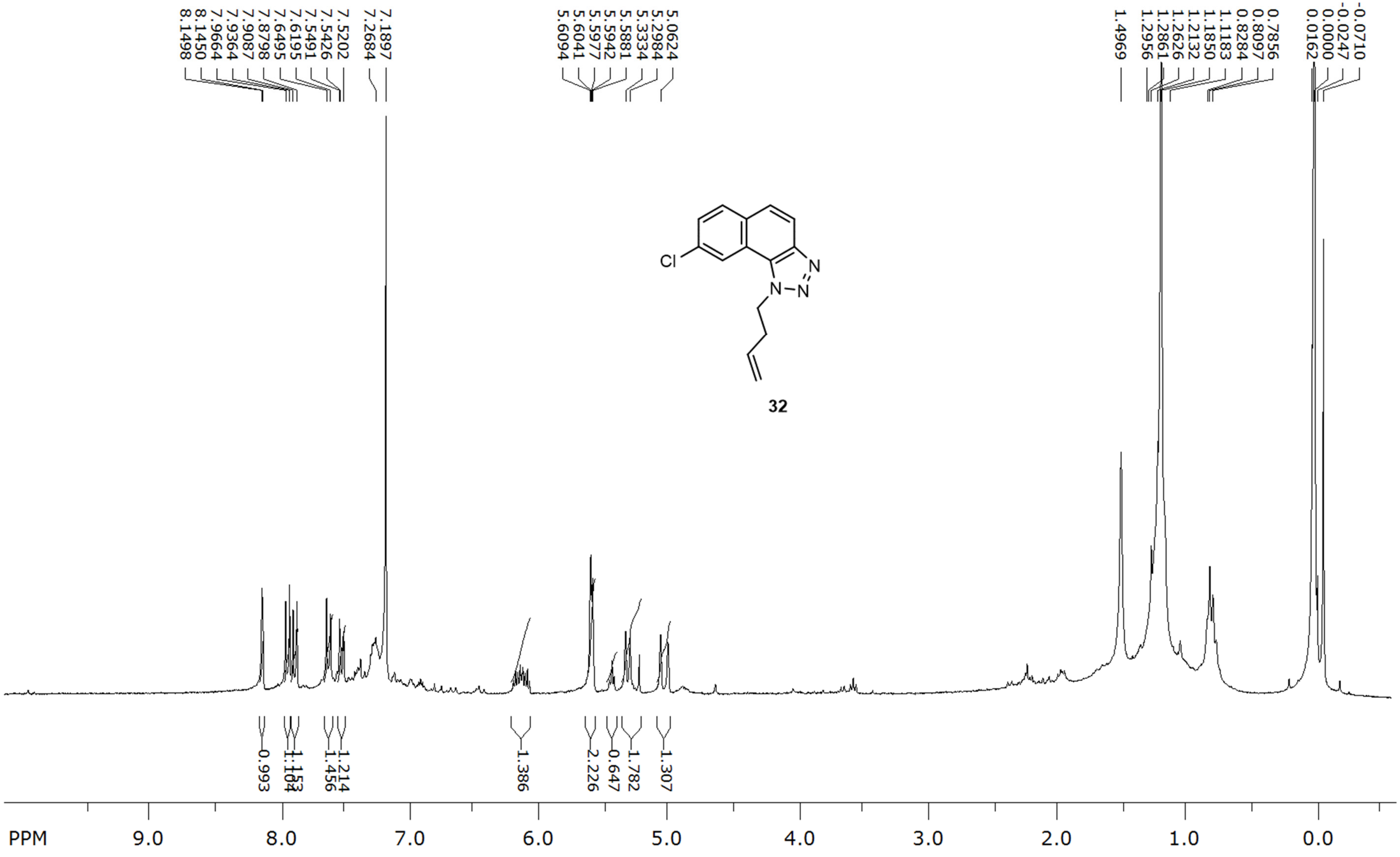

Figure S31. ^{1}H NMR ($CDCl_3$) spectrum of **32**.

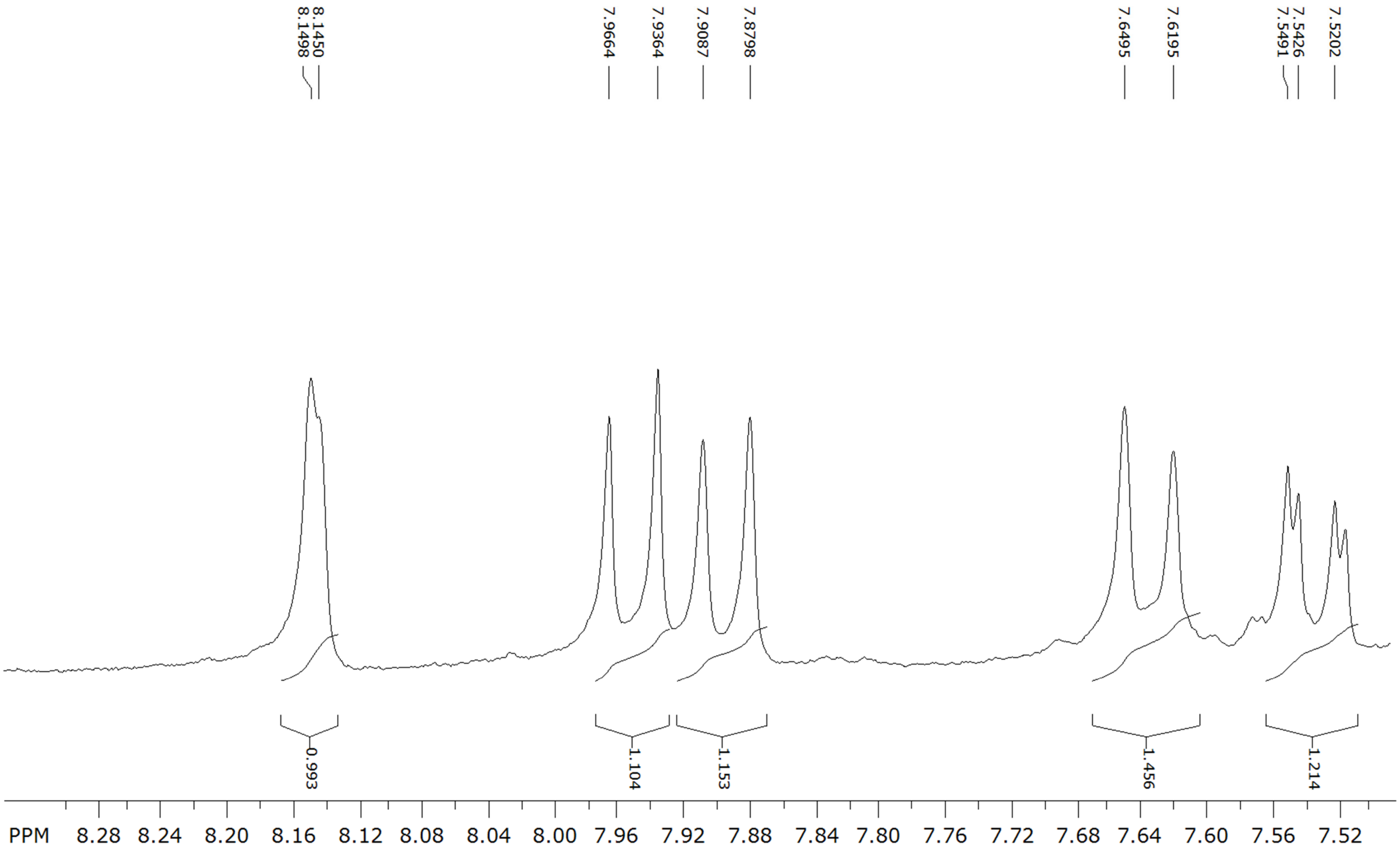

Figure S32. ^{1}H NMR ($CDCl_3$) spectrum of aromatic part of **32**.

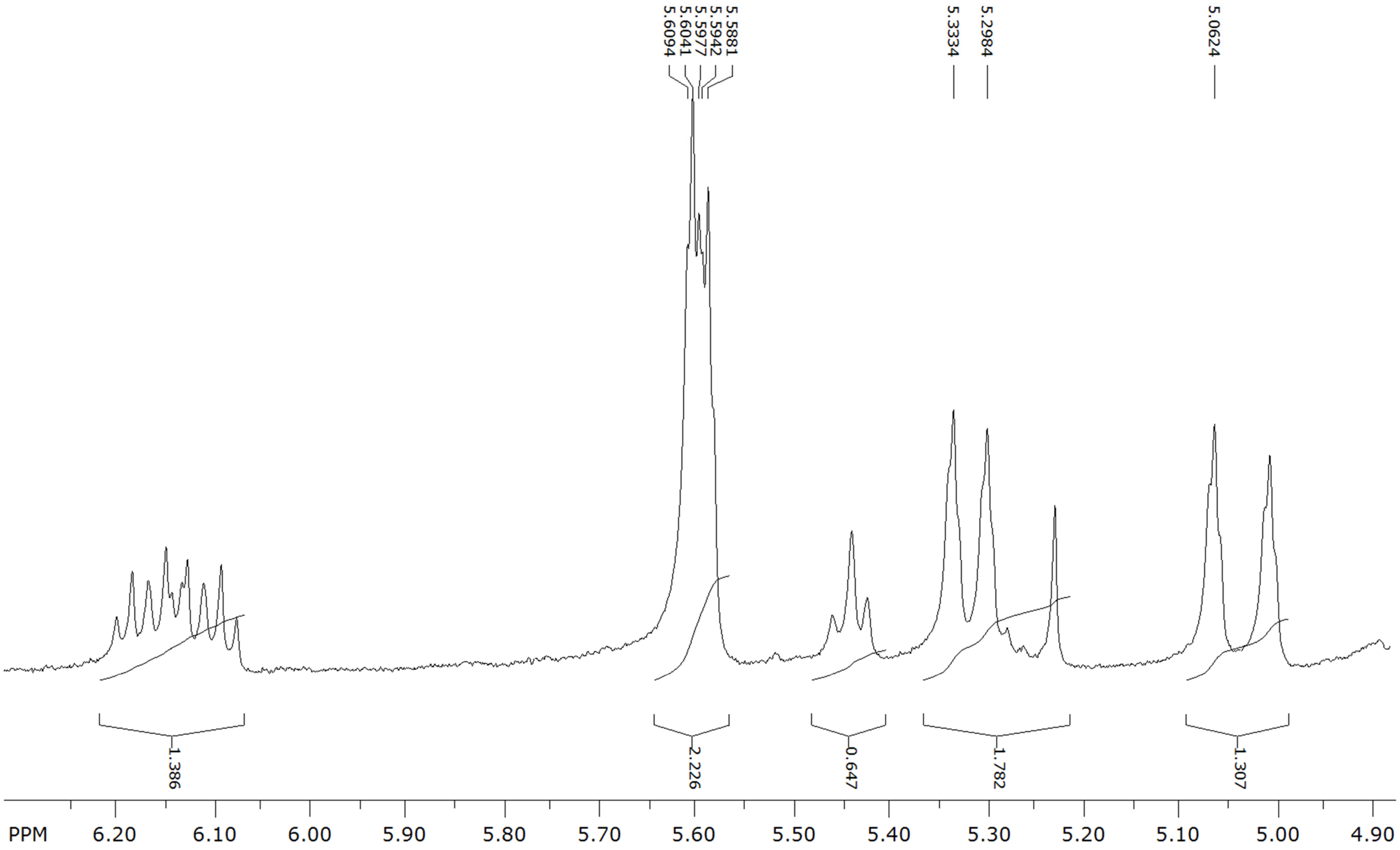

Figure S33. ^{1}H NMR ($CDCl_3$) spectrum of aliphatic part of **32**.

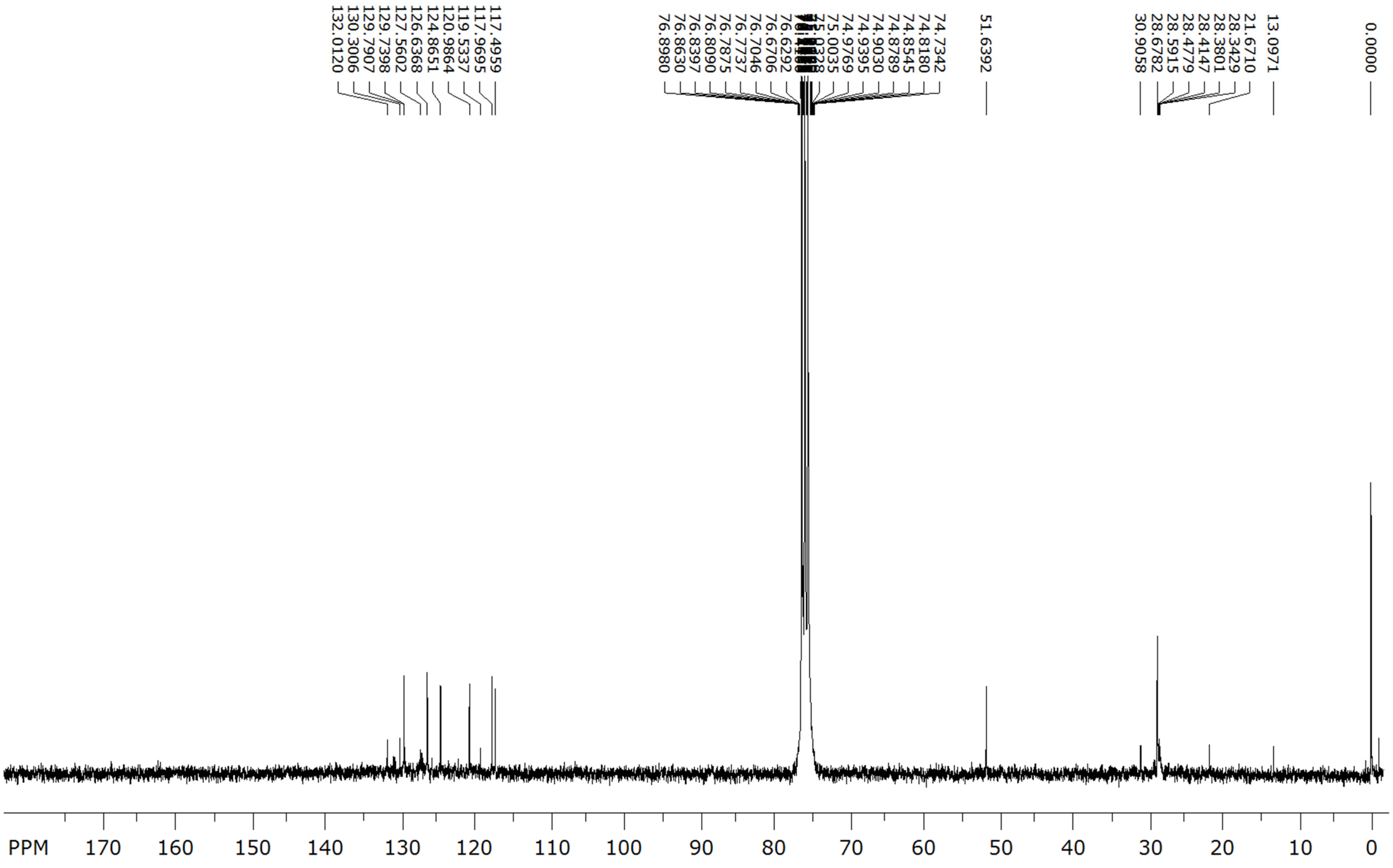

Figure S34. ^{13}C NMR ($CDCl_3$) spectrum of **32**.

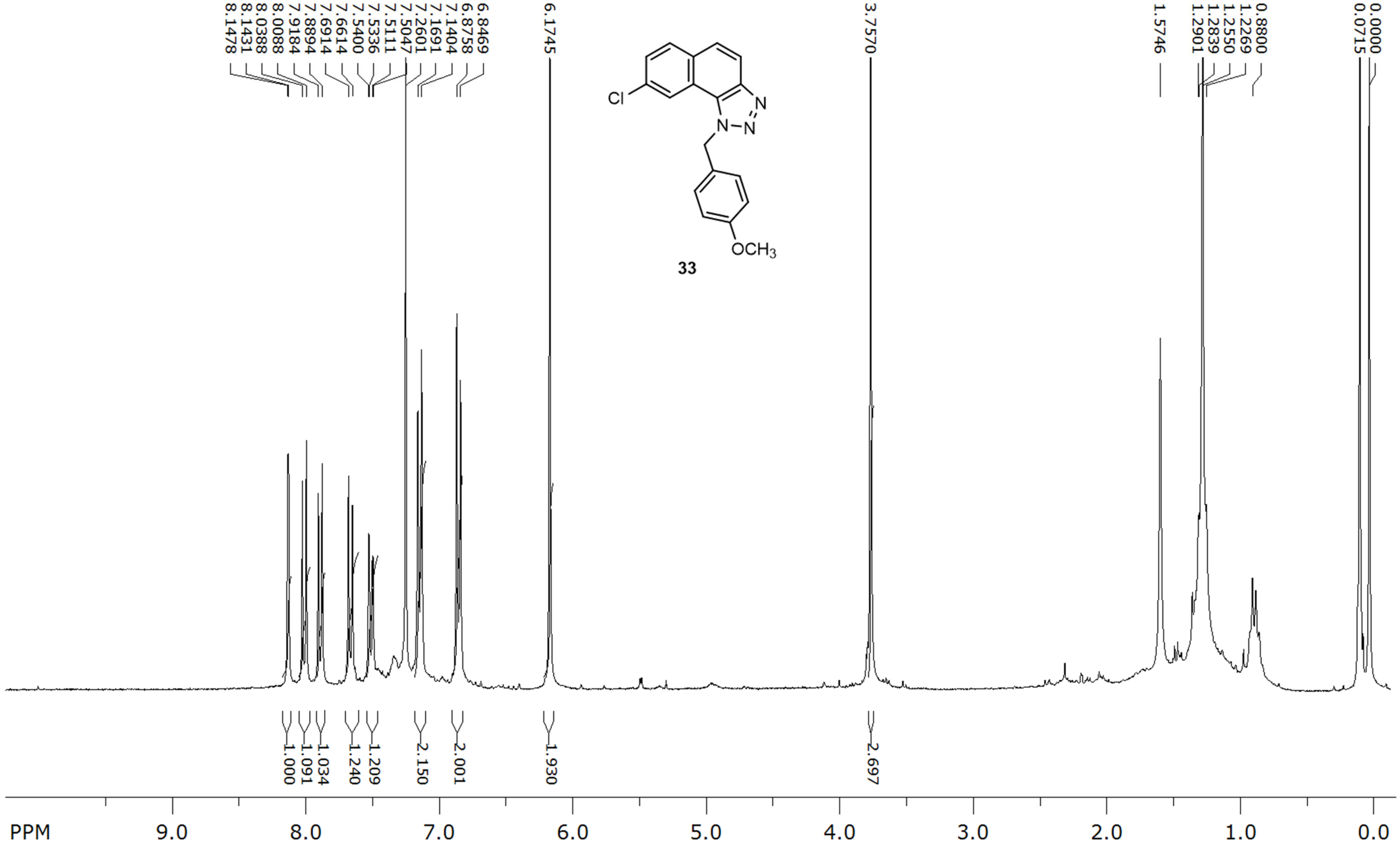

Figure S35. ^{1}H NMR ($CDCl_3$) spectrum of **33**.

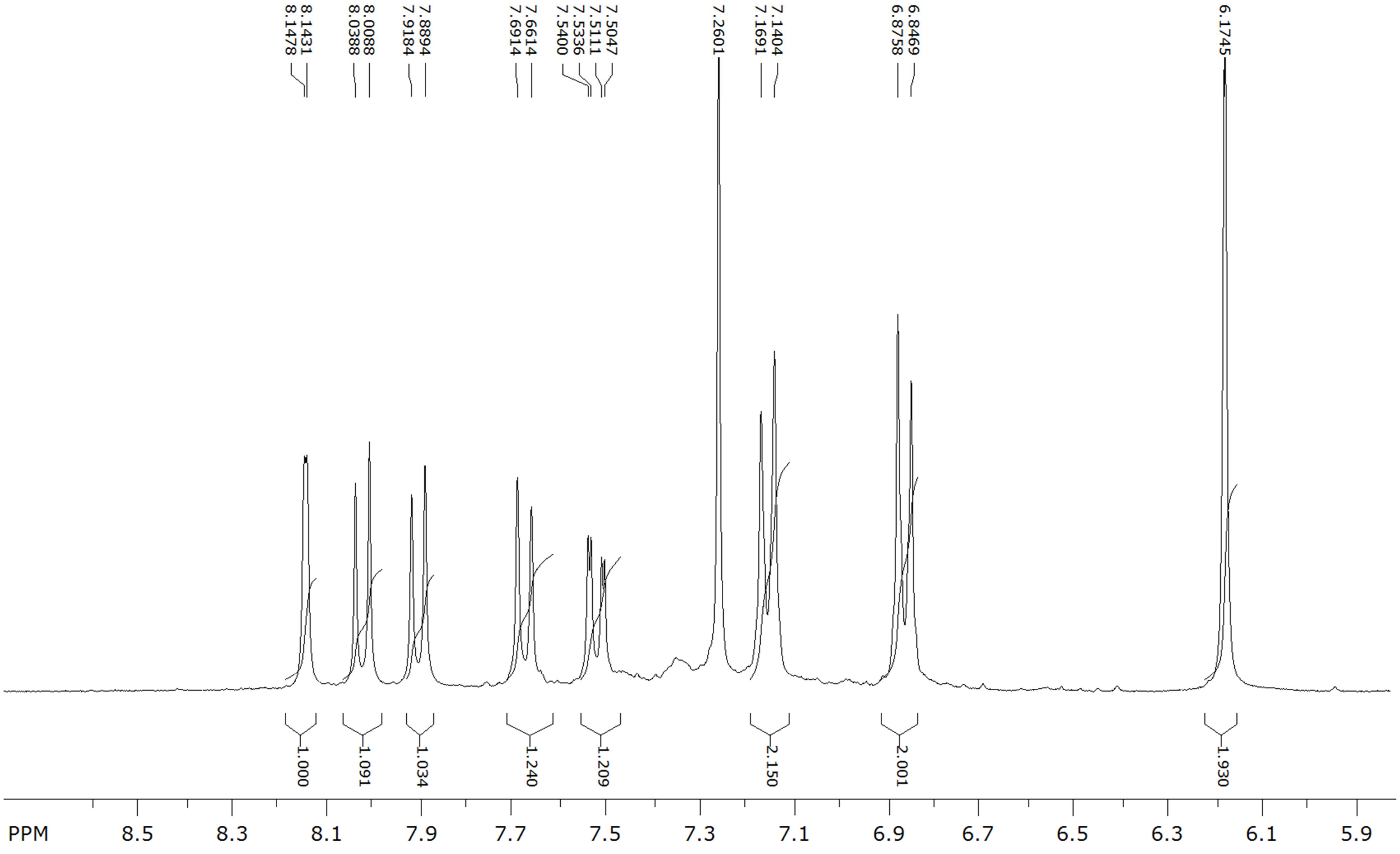

Figure S36. ^{1}H NMR ($CDCl_3$) spectrum of aromatic part of **33**.

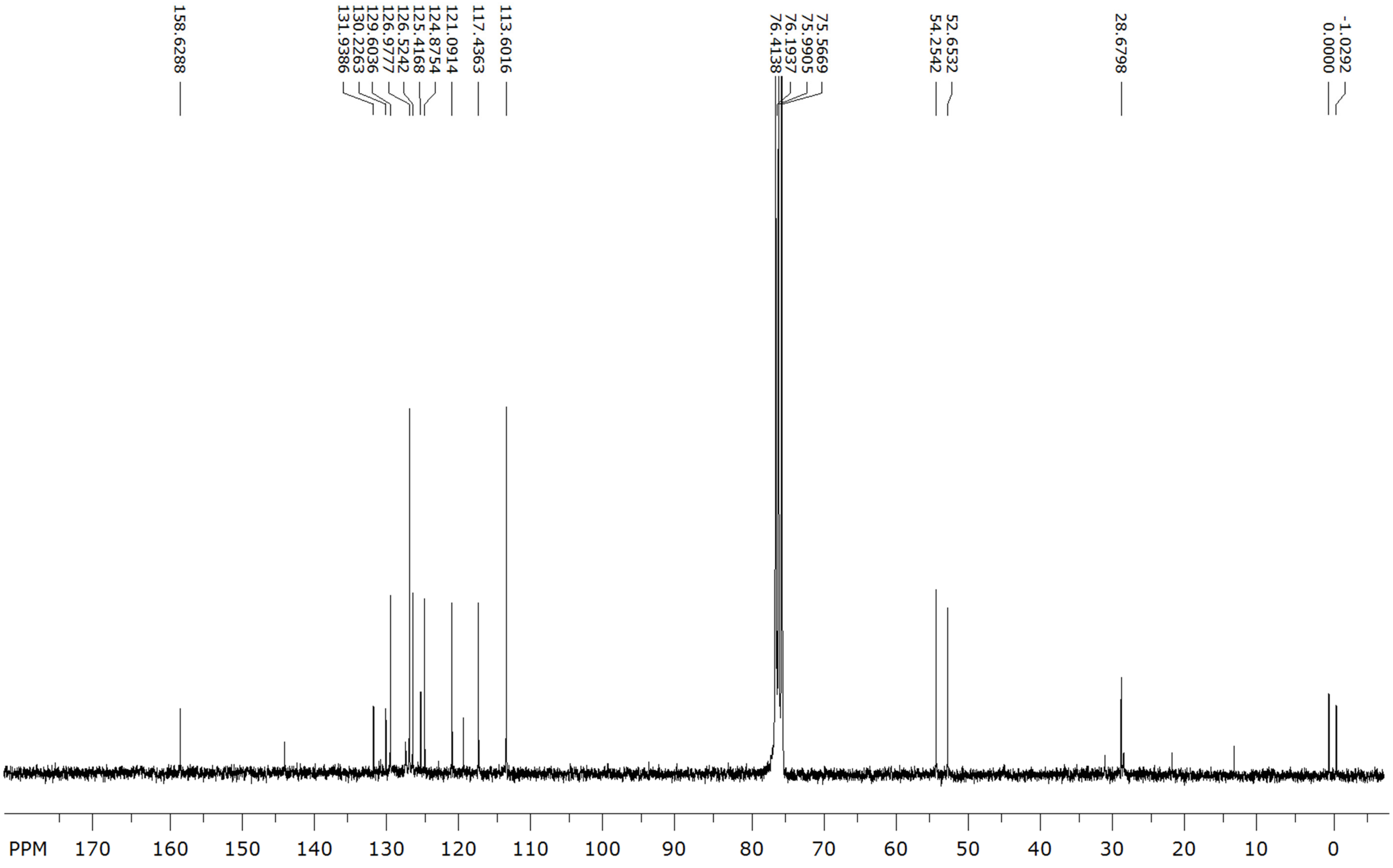

Figure S37. ^{13}C NMR ($CDCl_3$) spectrum of **33**.

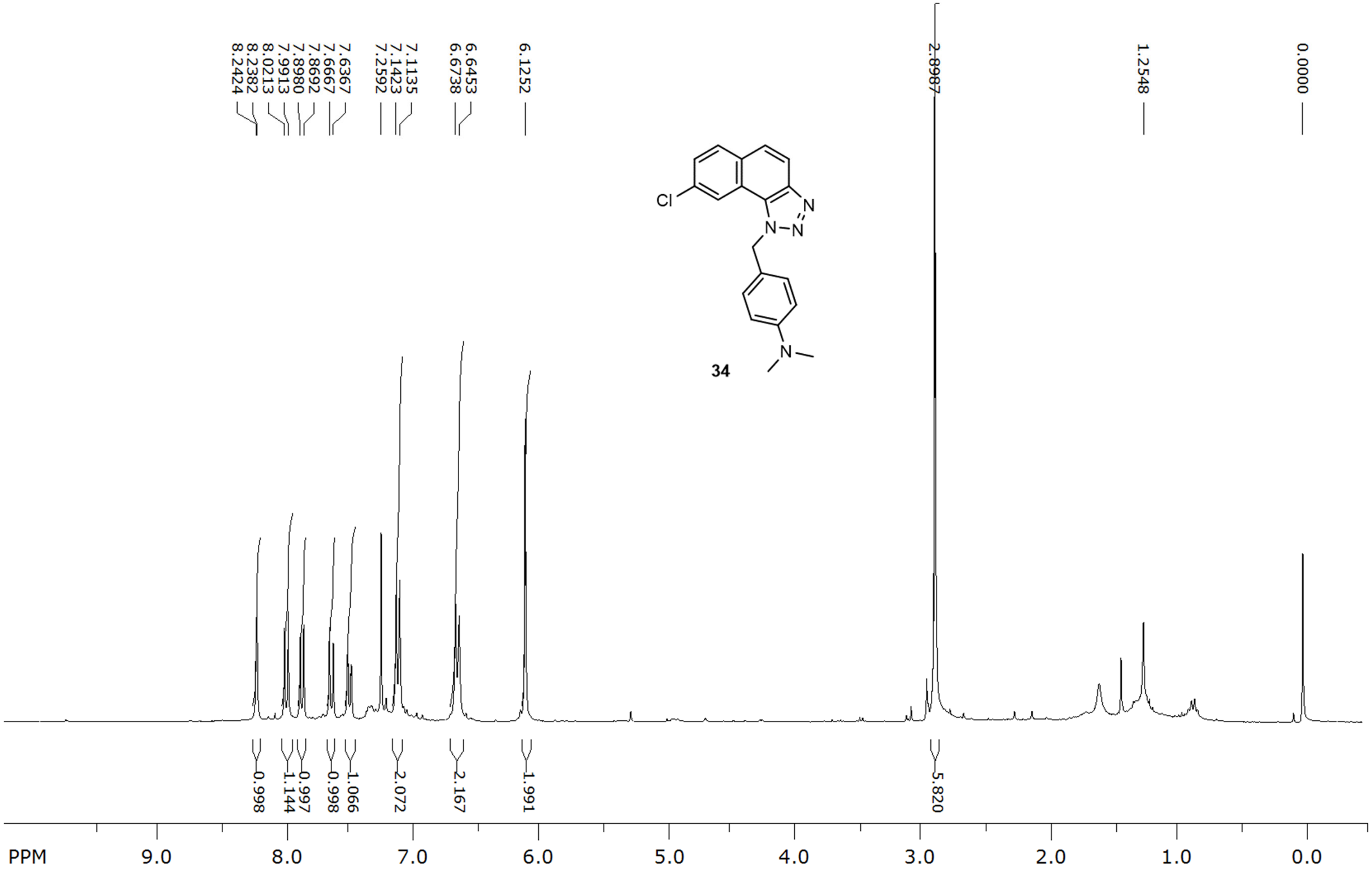

Figure S38. ^{1}H NMR ($CDCl_3$) spectrum of **34**.

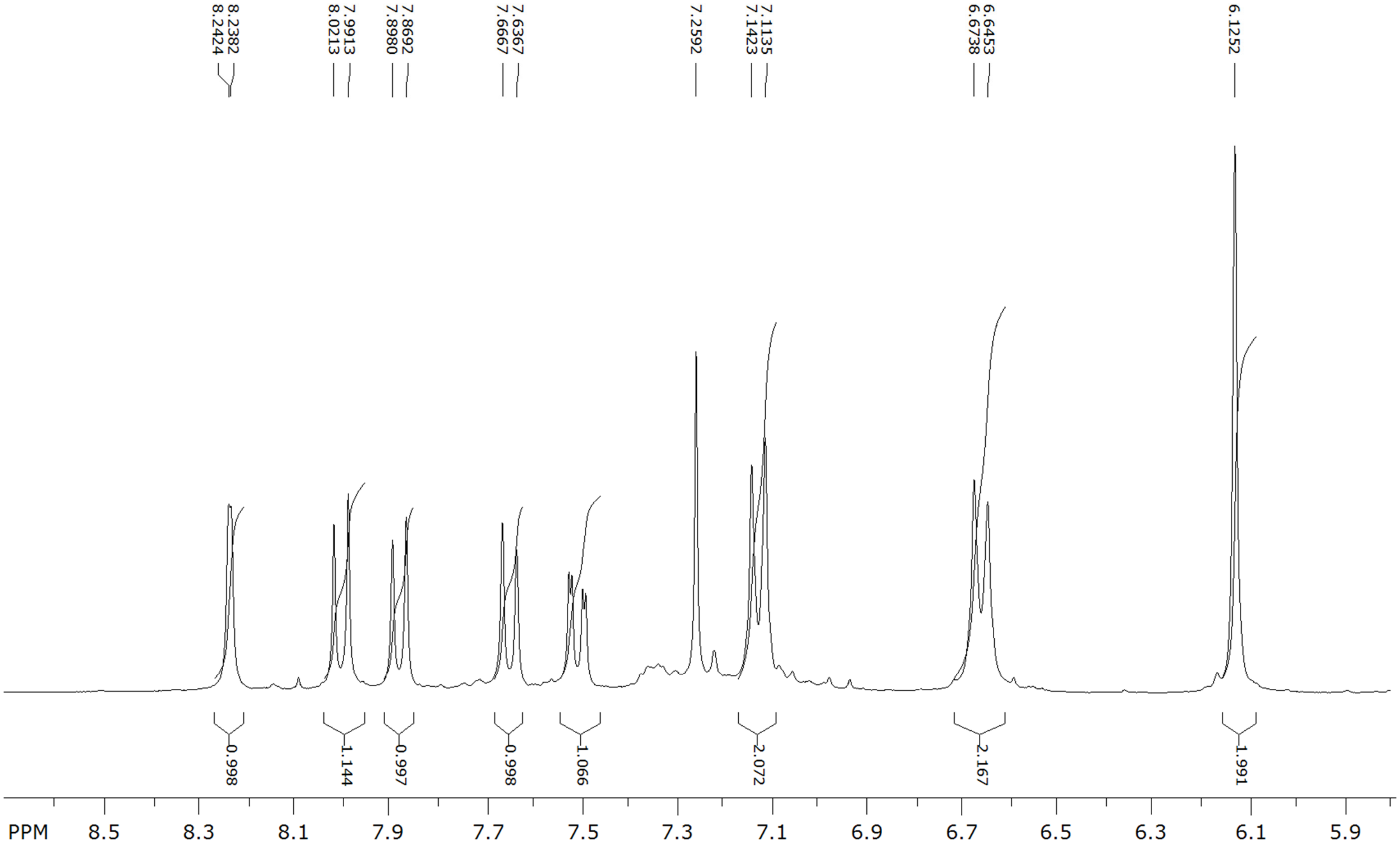

Figure S39. ^{1}H NMR ($CDCl_3$) spectrum of aromatic part of **34**.

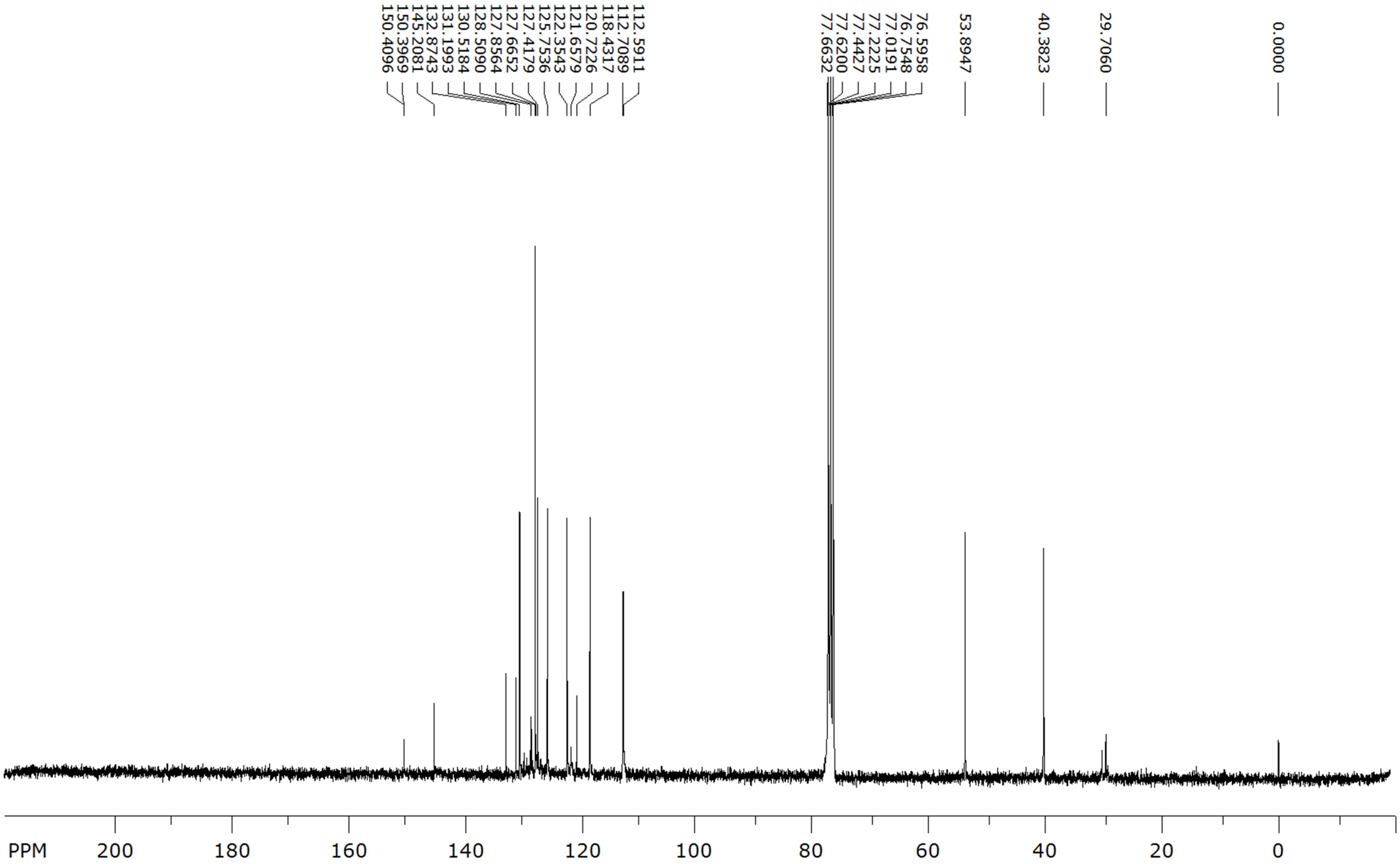

Figure S40. ^{13}C NMR ($CDCl_3$) spectrum of **34**.

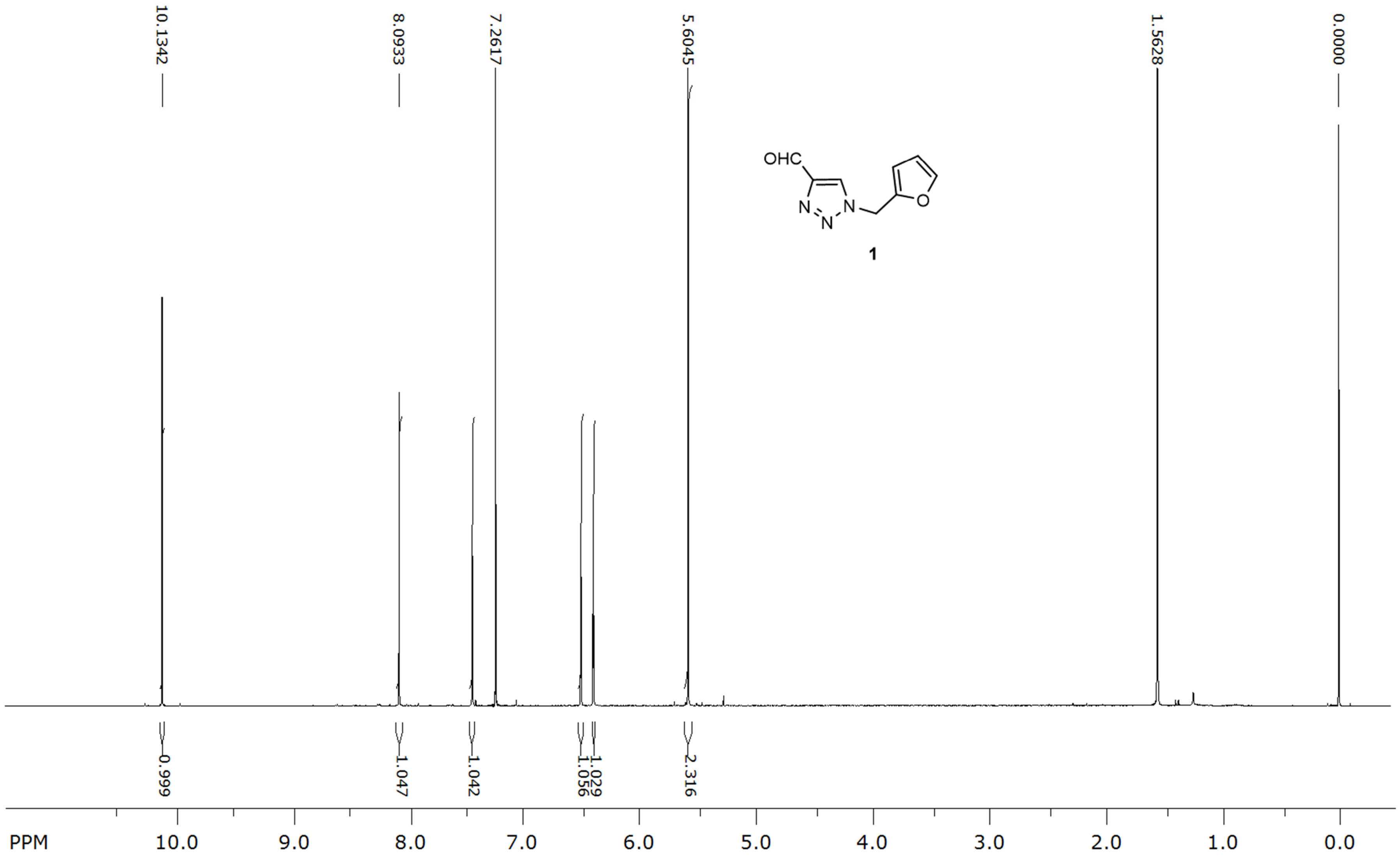

Figure S41. ^{1}H NMR ($CDCl_3$) spectrum of **1**.

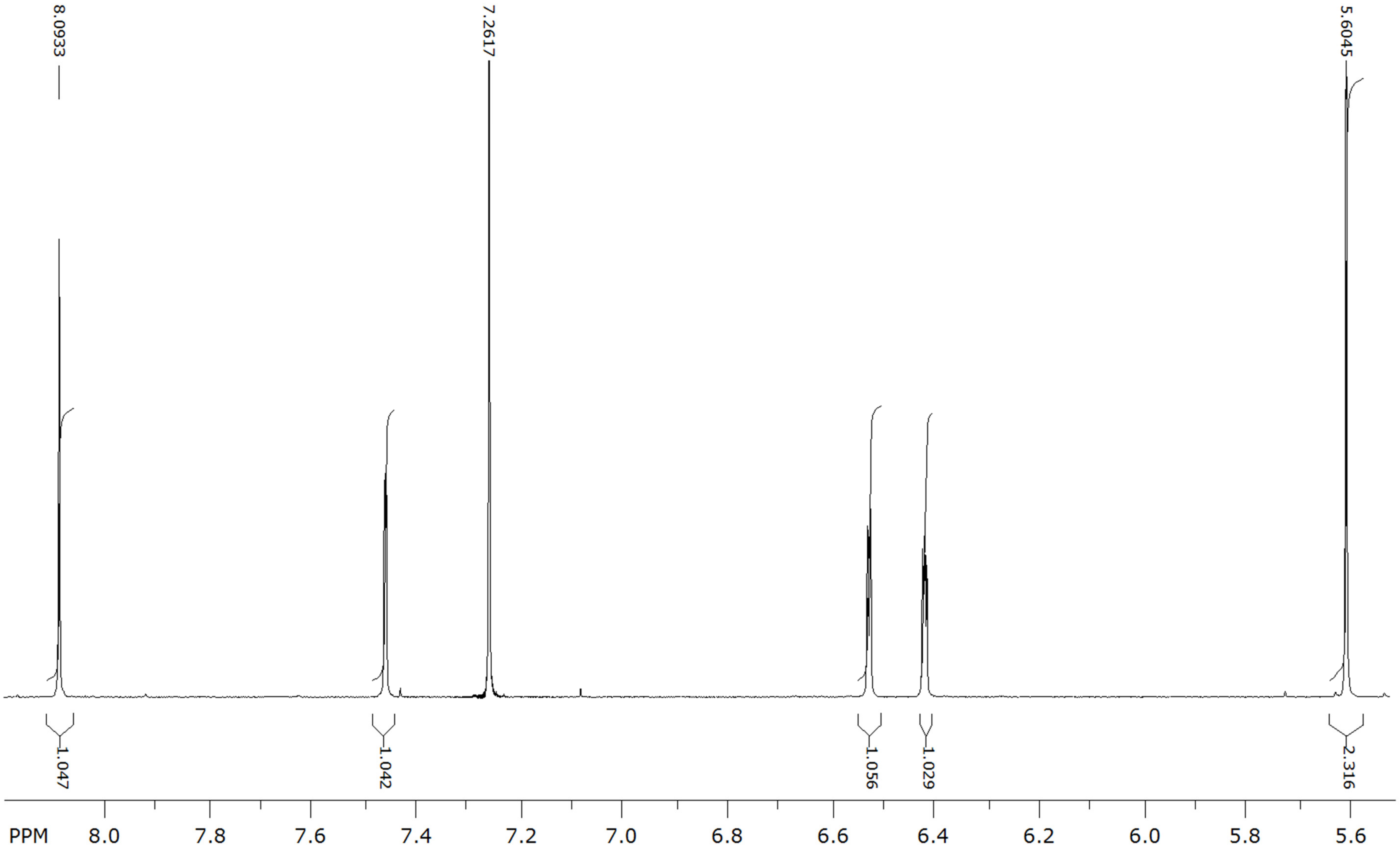

Figure S42. ^{1}H NMR ($CDCl_3$) spectrum of aromatic part of **1**.

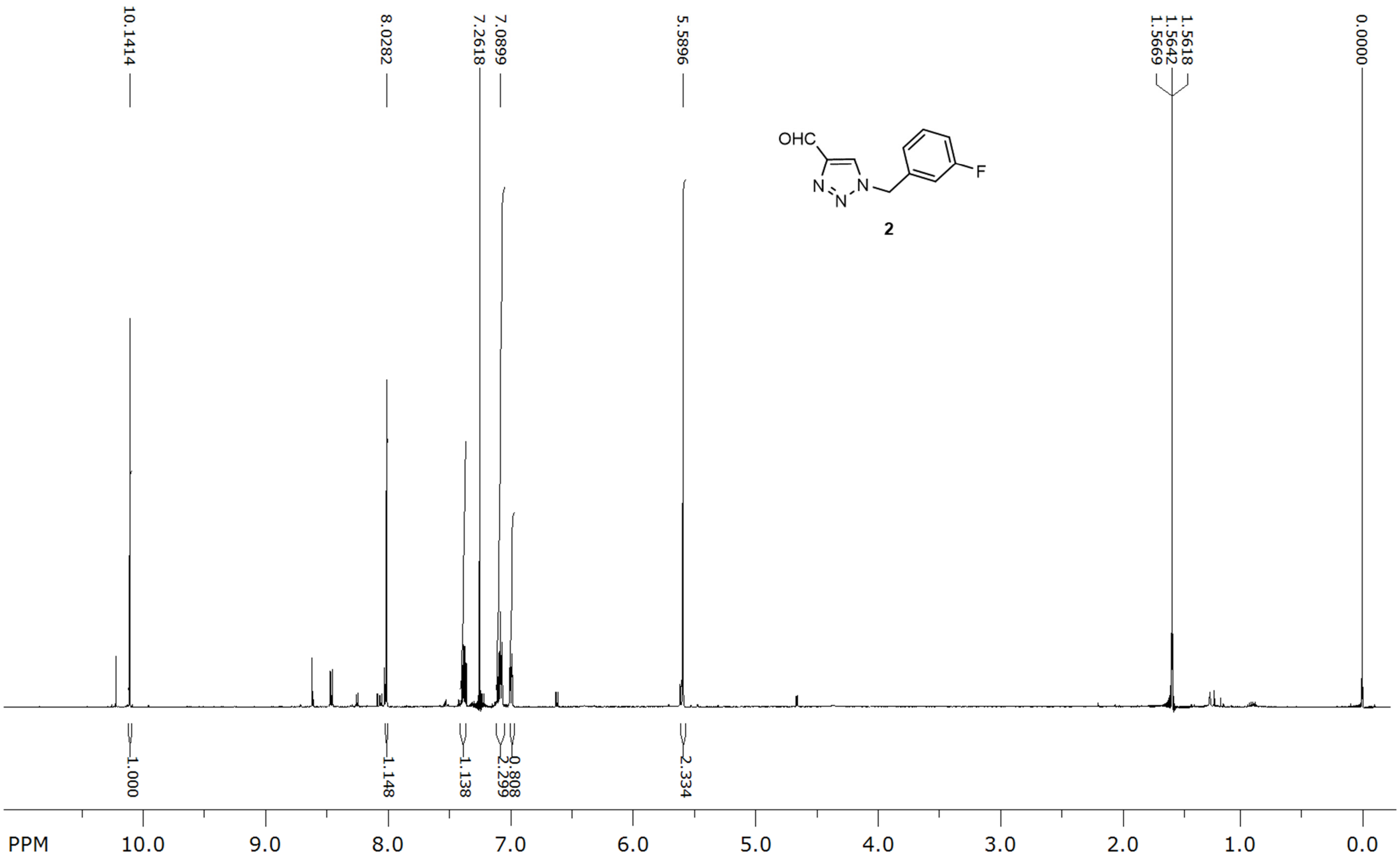

Figure S43. ^{1}H NMR ($CDCl_3$) spectrum of **2**.

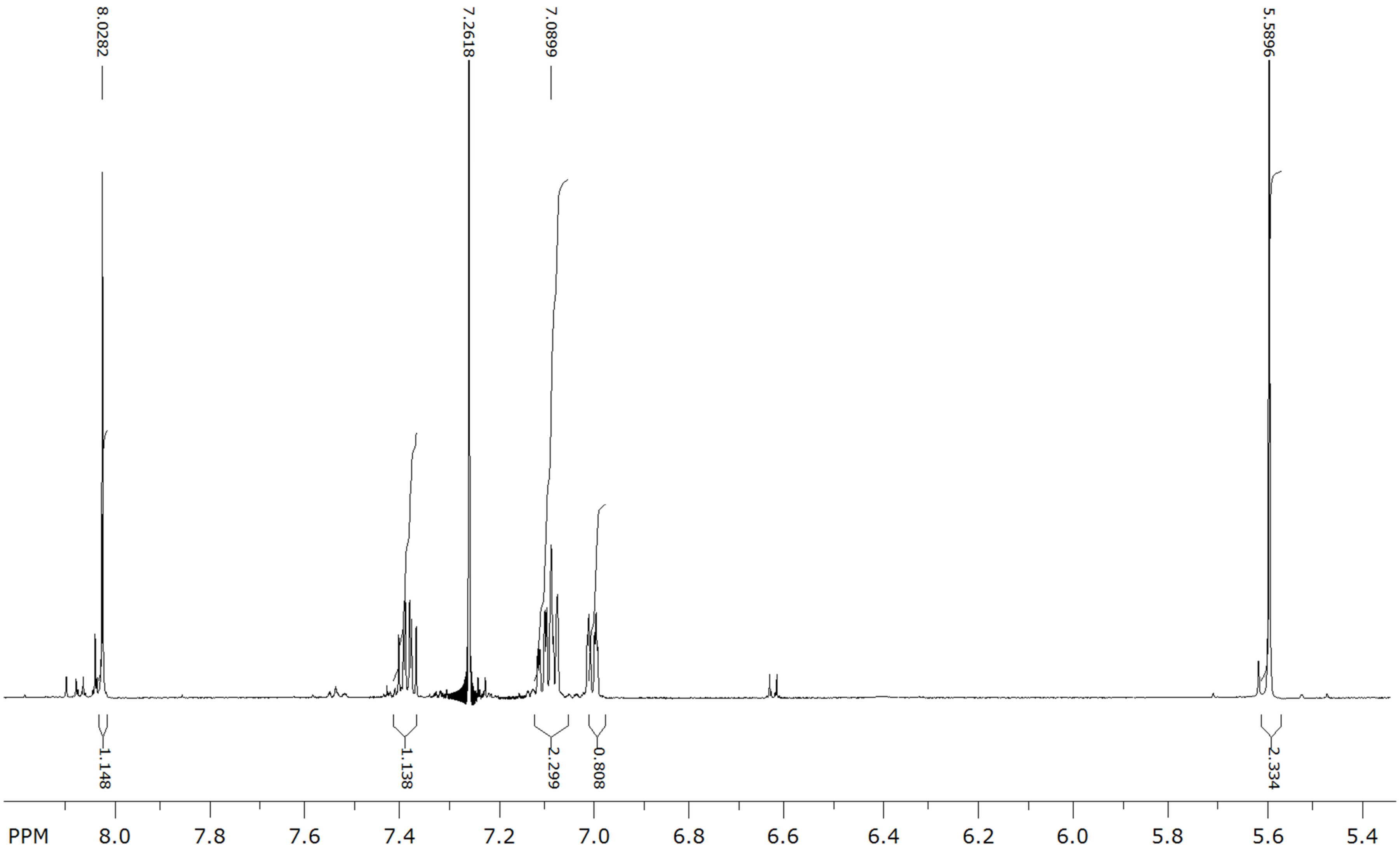

Figure S44. ^{1}H NMR ($CDCl_3$) spectrum of aromatic part of **2**.

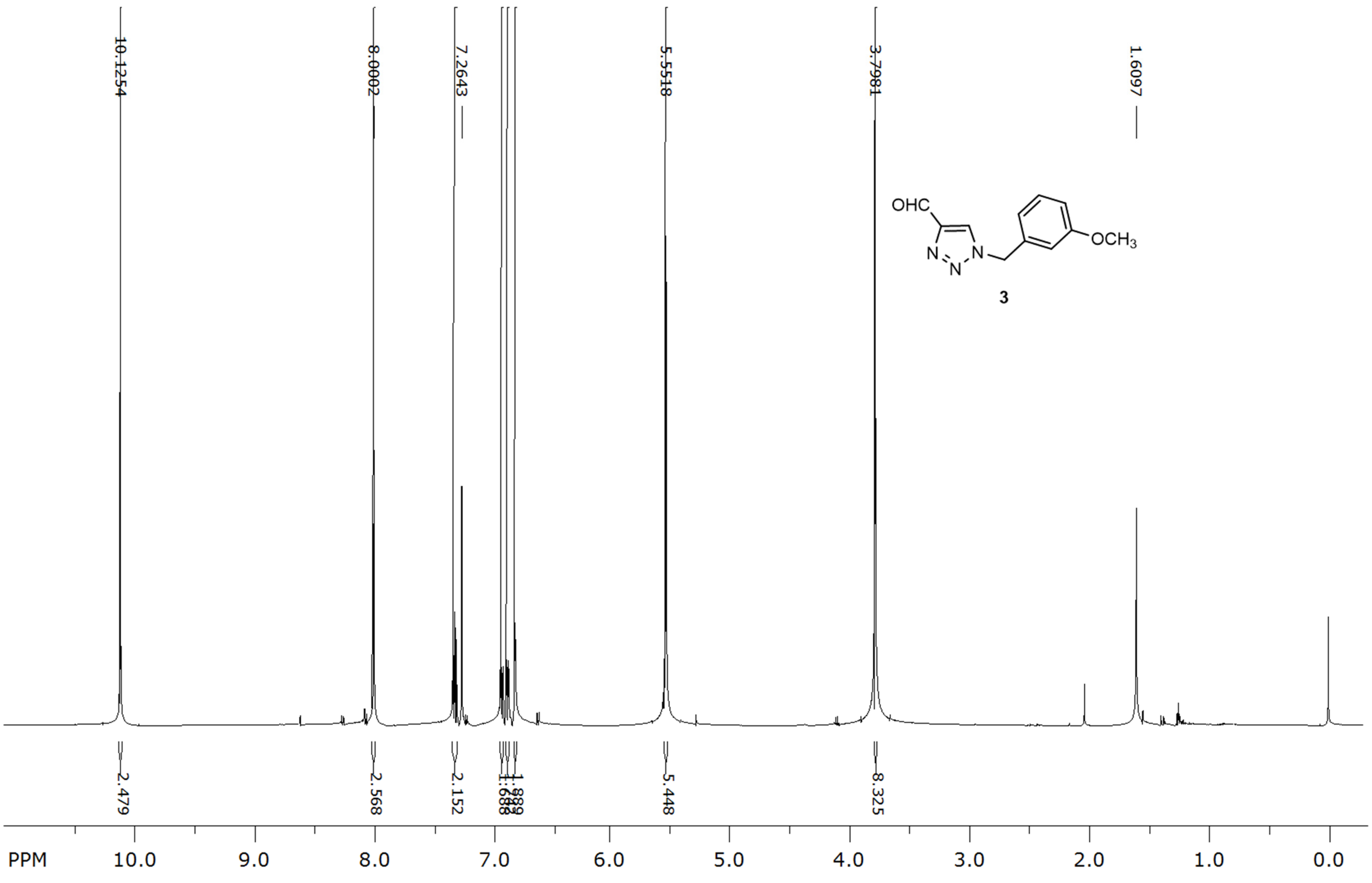

Figure S45. ^{1}H NMR ($CDCl_3$) spectrum of **3**.

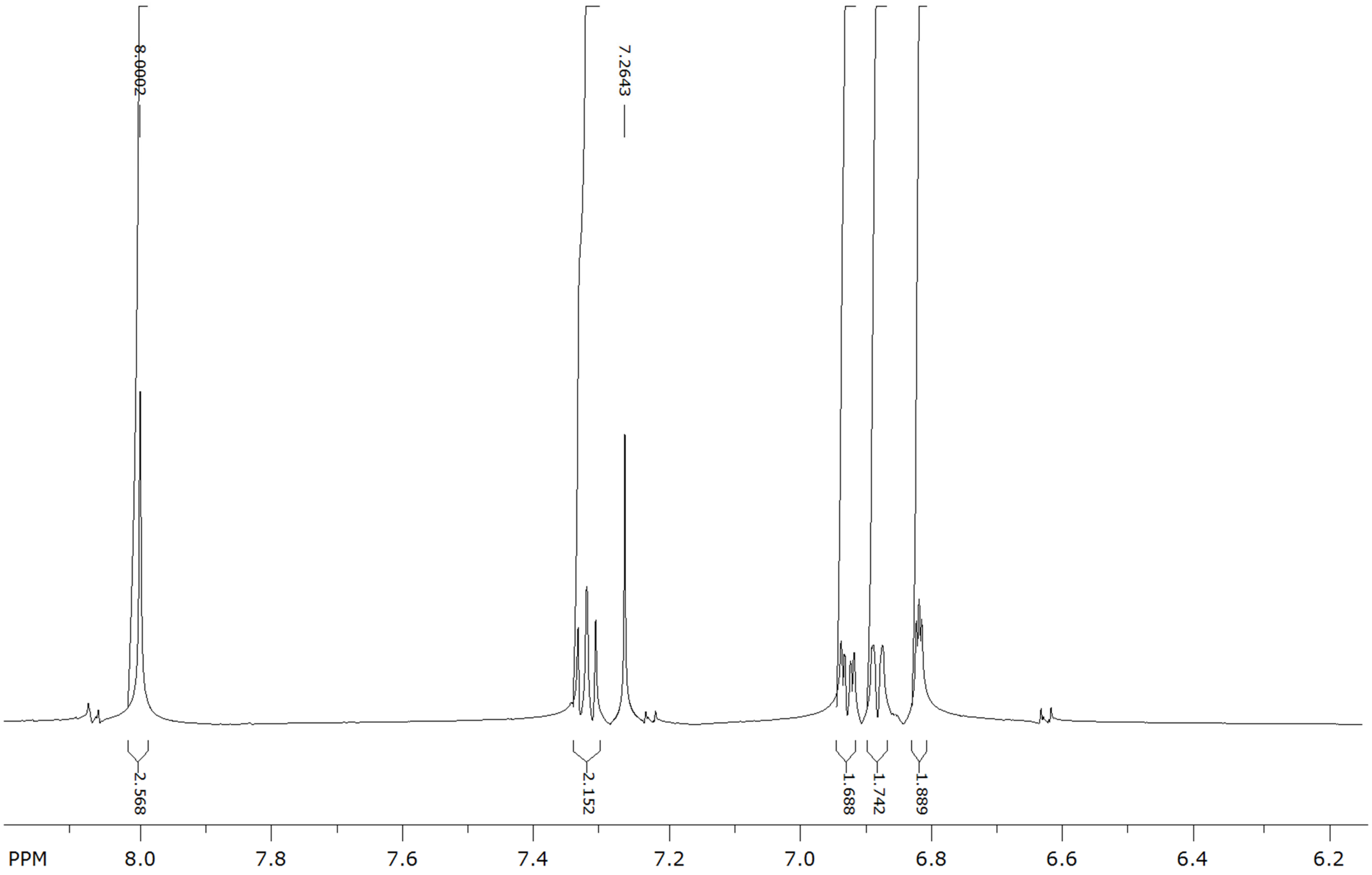

Figure S46. ^{1}H NMR ($CDCl_3$) spectrum of aromatic part of **3**.

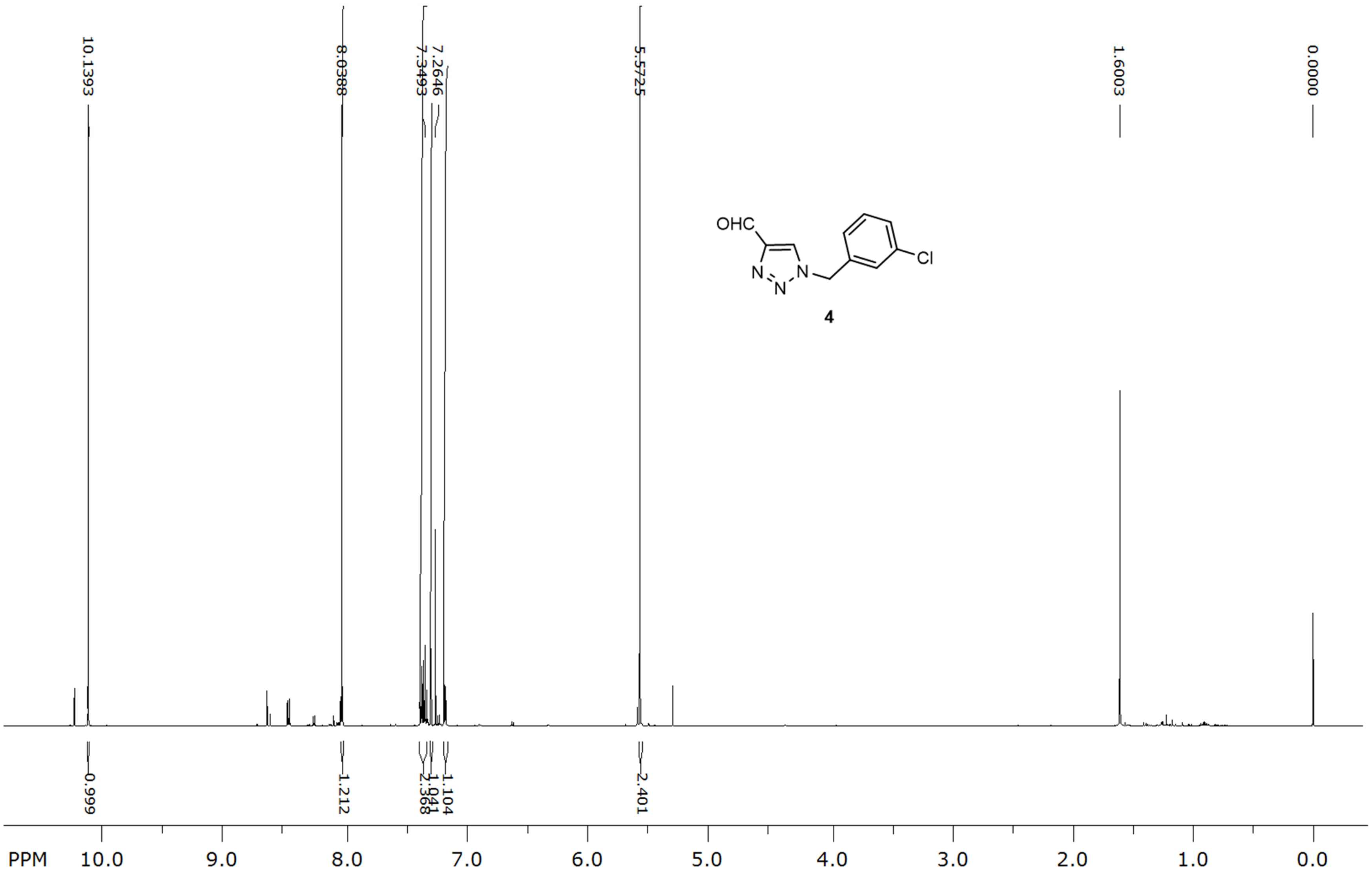

Figure S47. ^{1}H NMR ($CDCl_3$) spectrum of **4**.

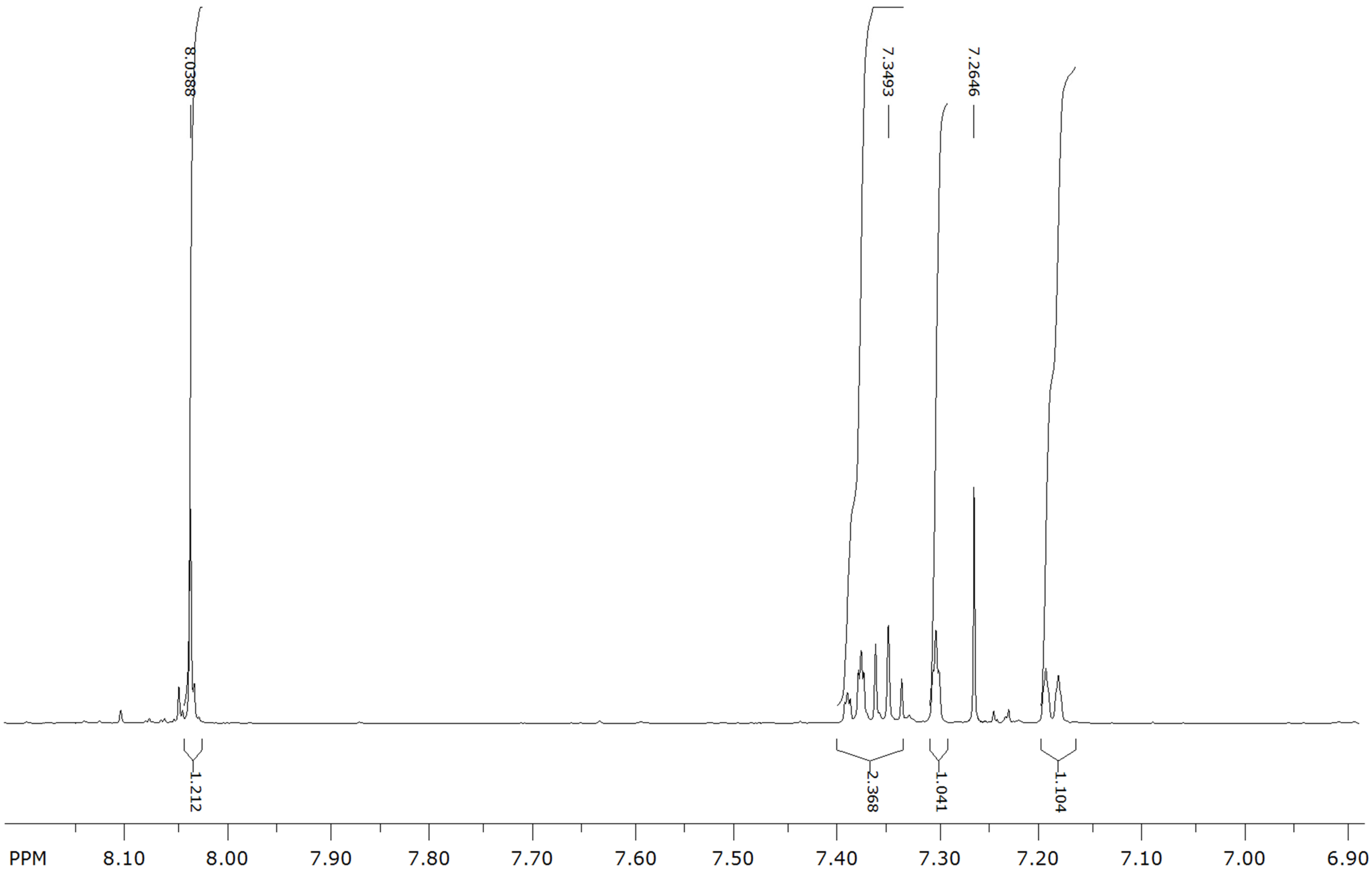

Figure S48. ^{1}H NMR ($CDCl_3$) spectrum of aromatic part of **4**.

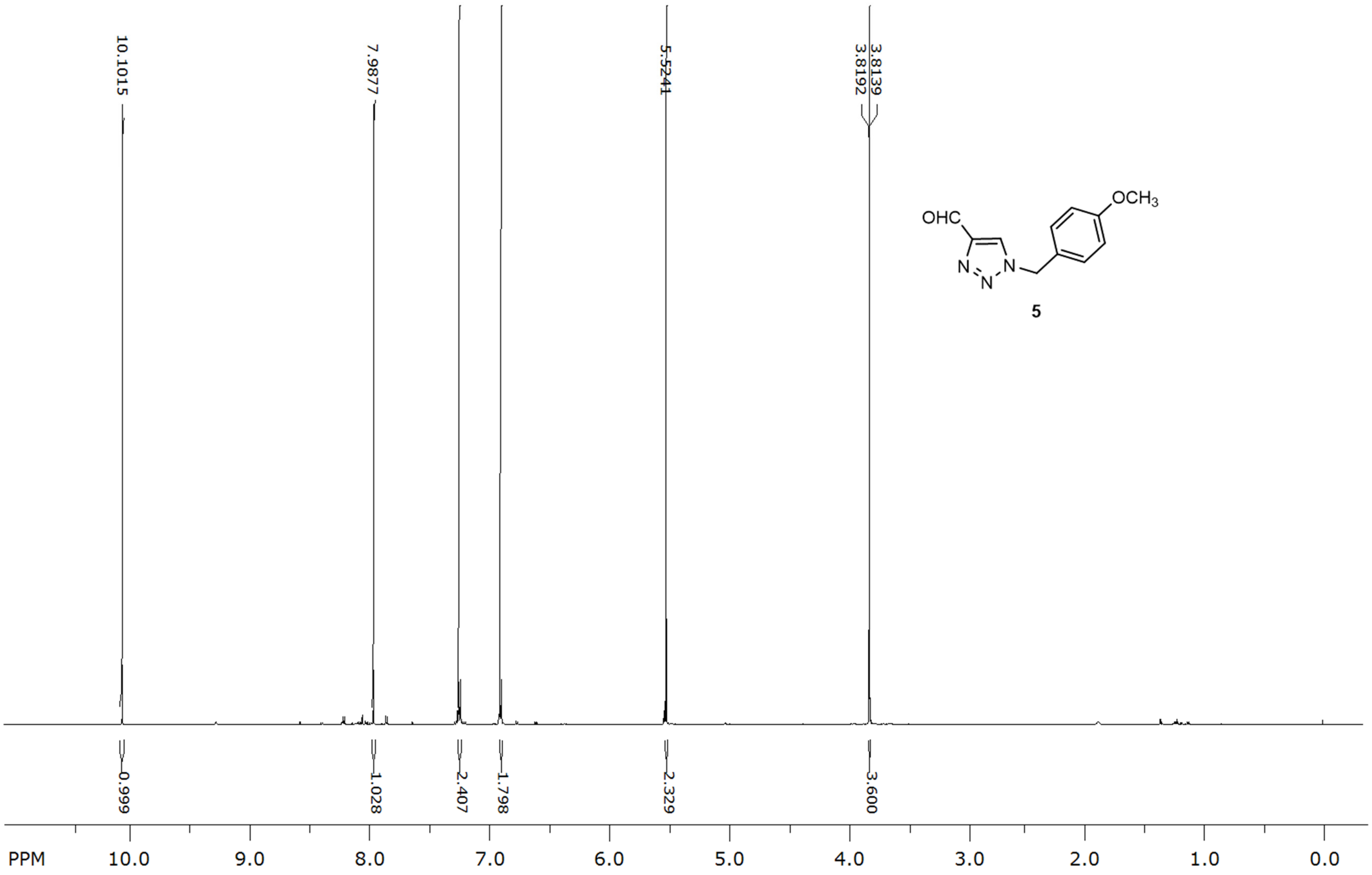

Figure S49. ^{1}H NMR ($CDCl_3$) spectrum of **5**.

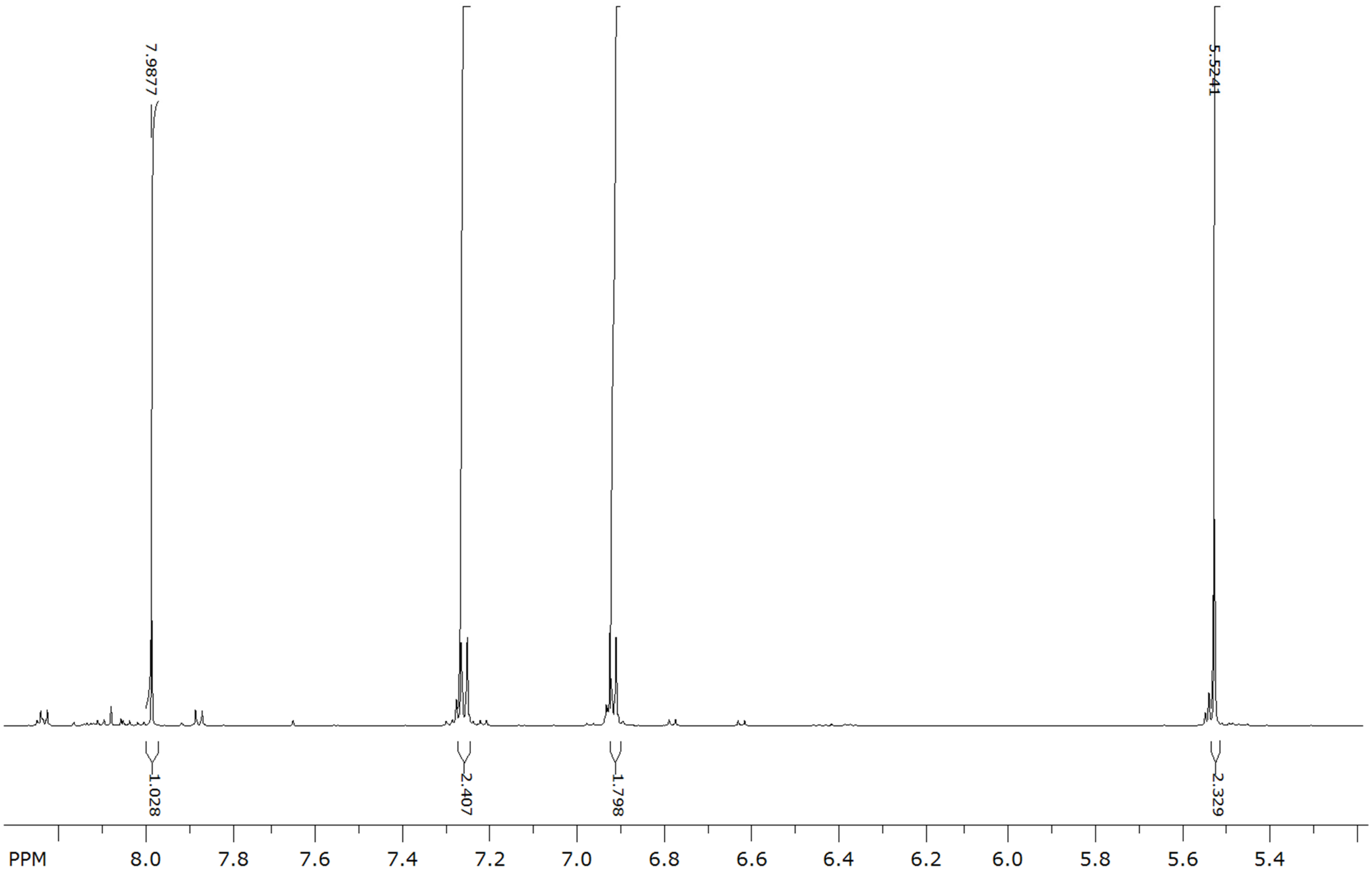

Figure S50. ^{1}H NMR ($CDCl_3$) spectrum of aromatic part of **5**.

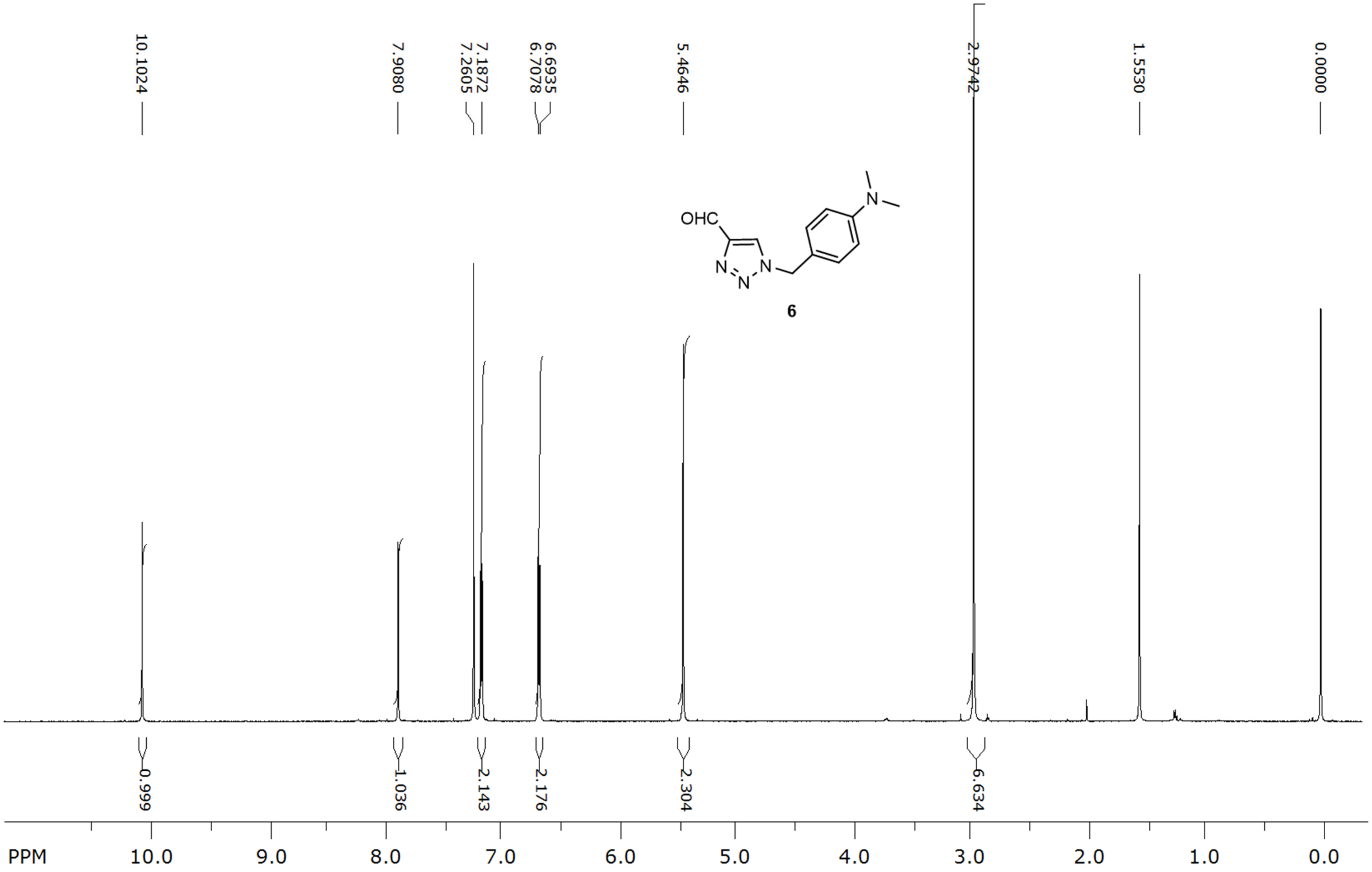

Figure S51. ^{1}H NMR ($CDCl_3$) spectrum of **6**.

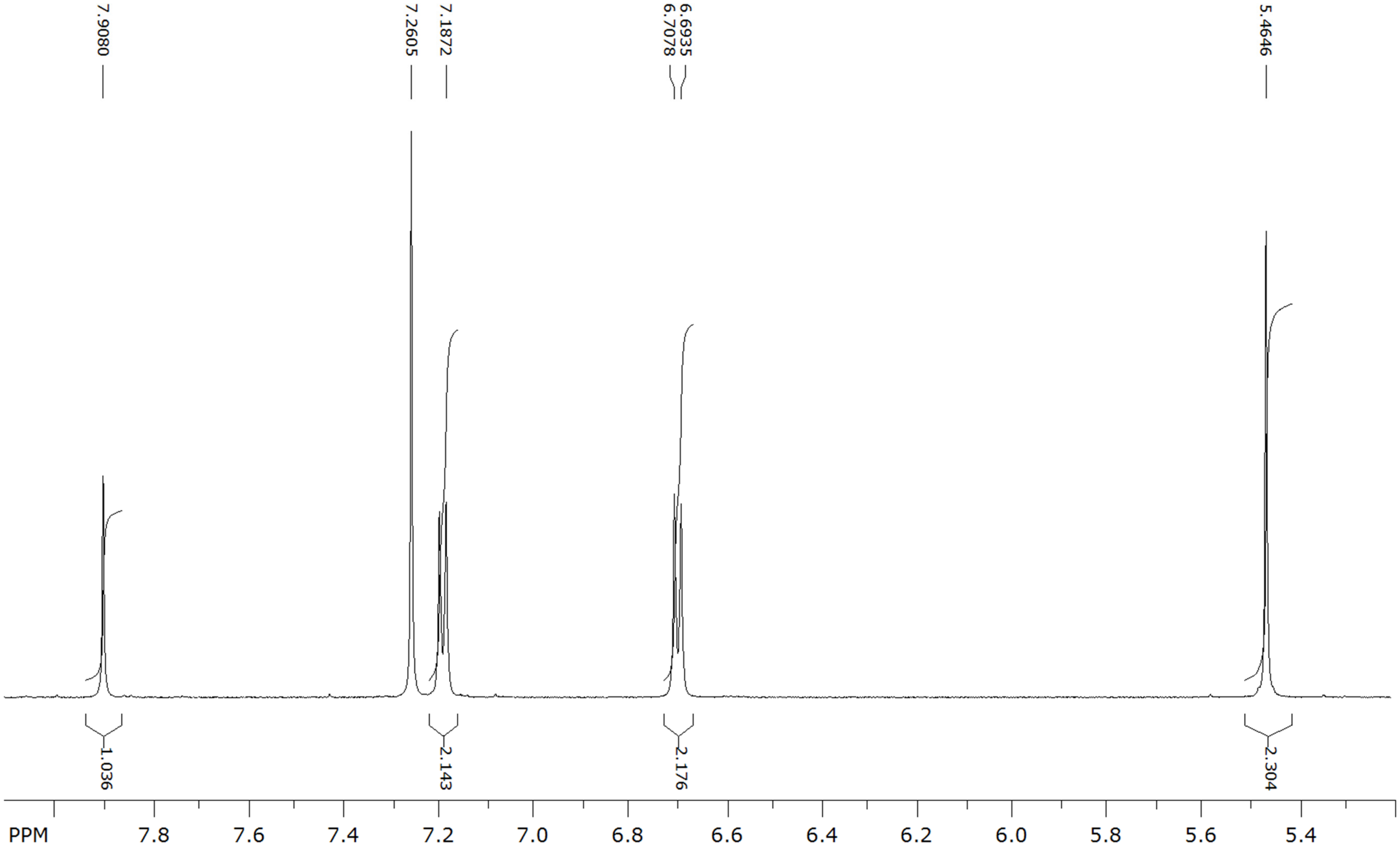

Figure S52. ^{1}H NMR ($CDCl_3$) spectrum of aromatic part of **6**.

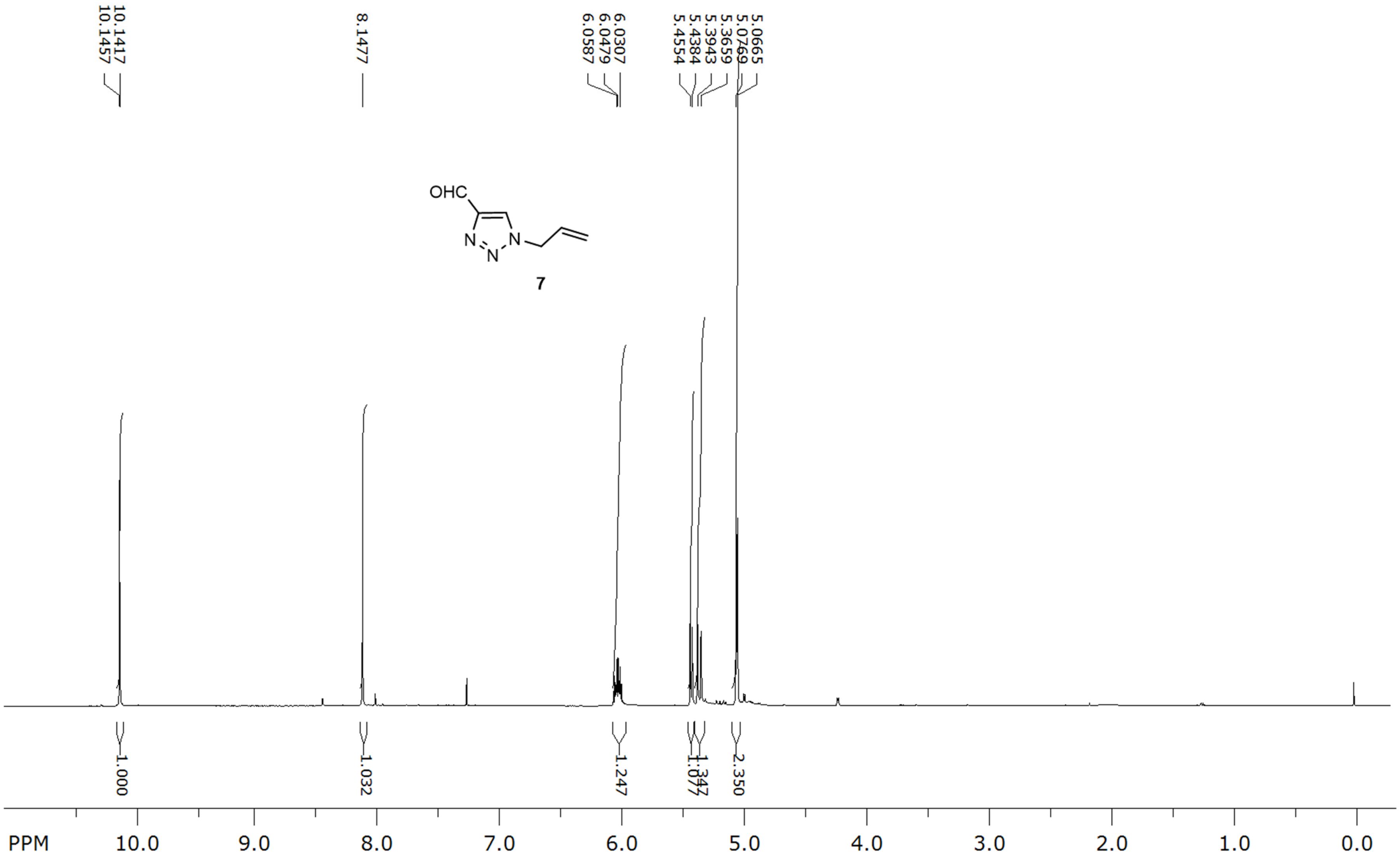

Figure S53. ^{1}H NMR ($CDCl_3$) spectrum of **7**.

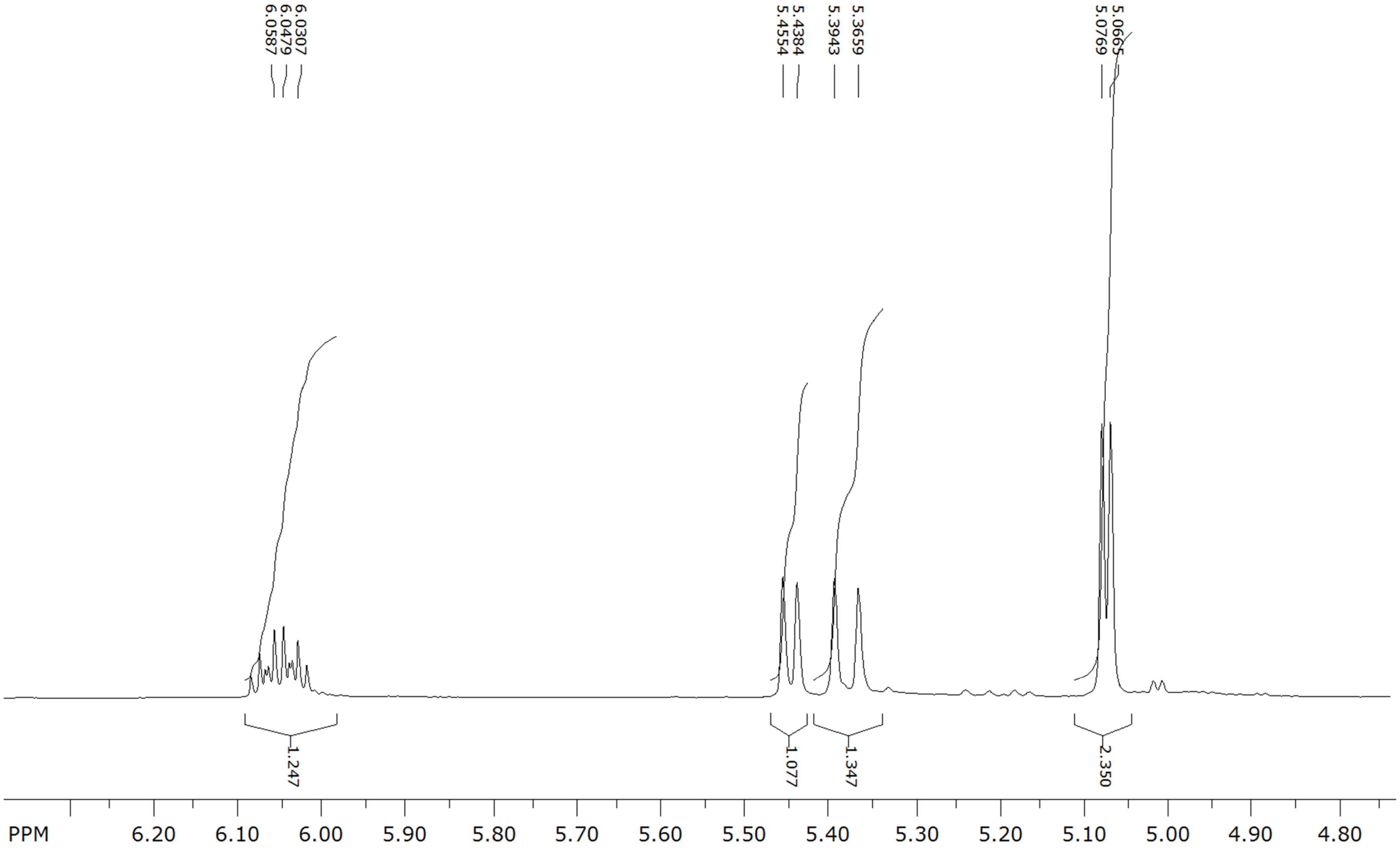

Figure S54. ^{1}H NMR ($CDCl_3$) spectrum of aliphatic part of **7**.

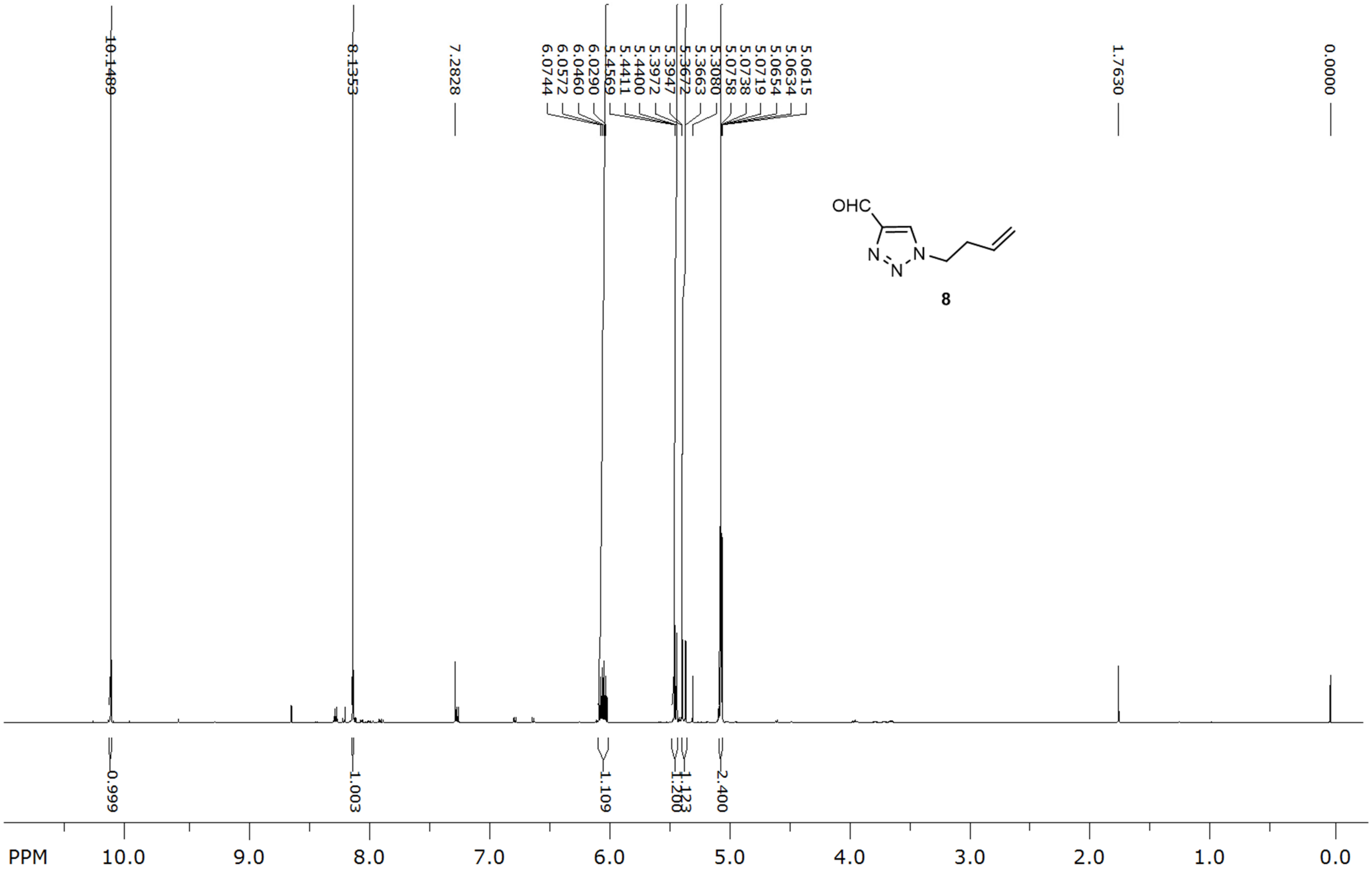

Figure S55. ^{1}H NMR ($CDCl_3$) spectrum of **8**.

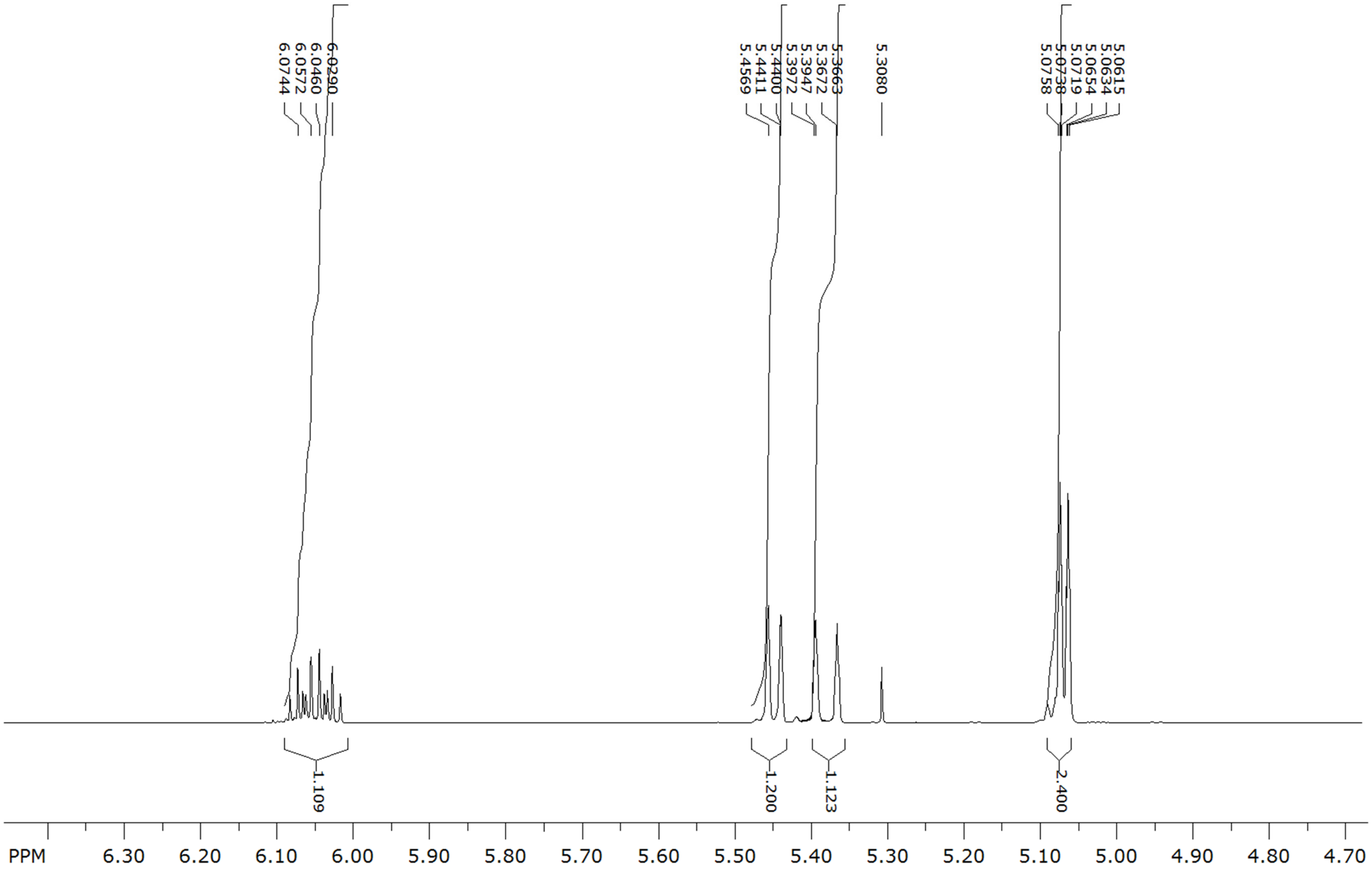

Figure S56. ^{1}H NMR ($CDCl_3$) spectrum of aliphatic part of **8**.

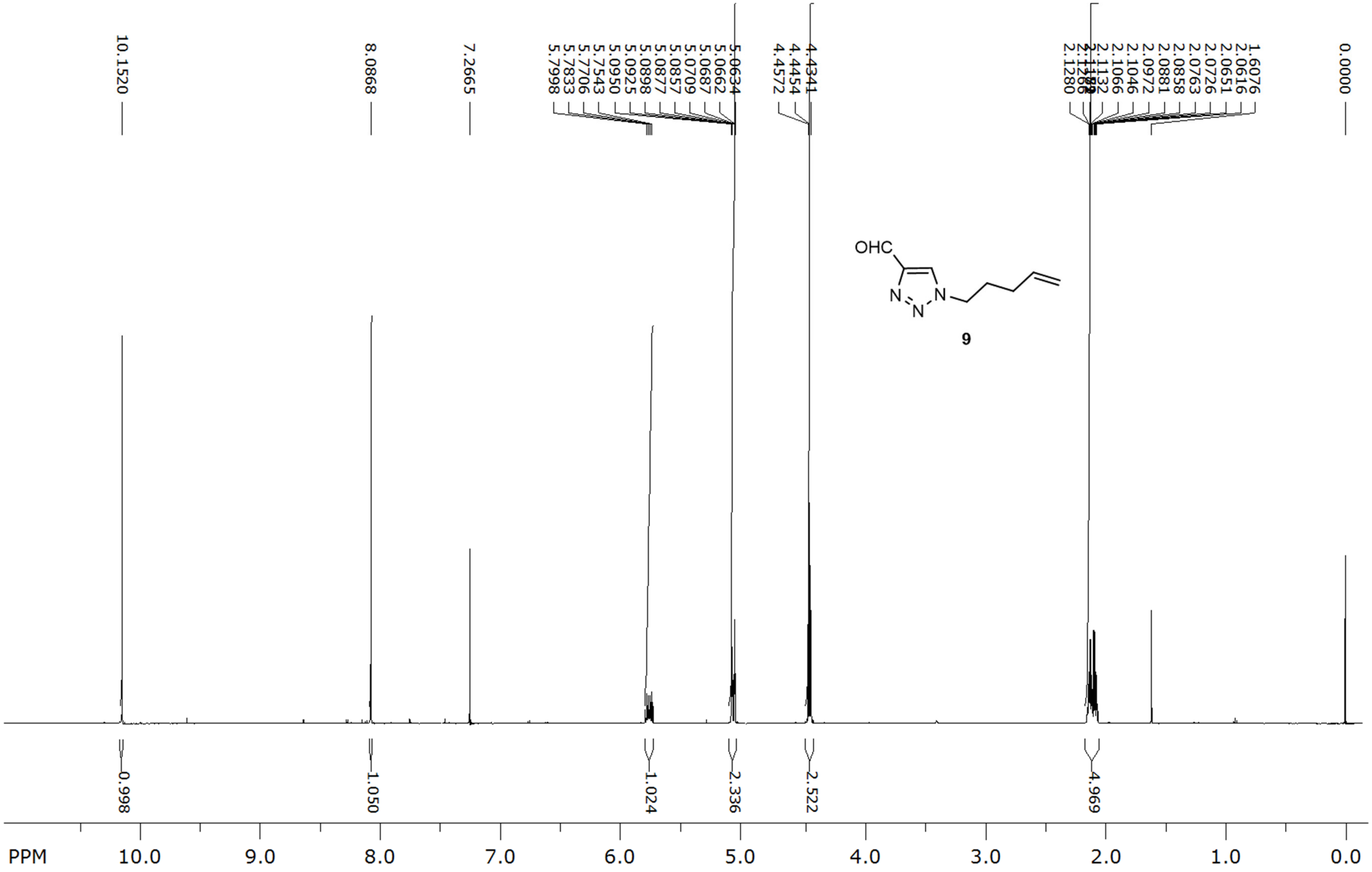

Figure S57. ^{1}H NMR ($CDCl_3$) spectrum of **9**.

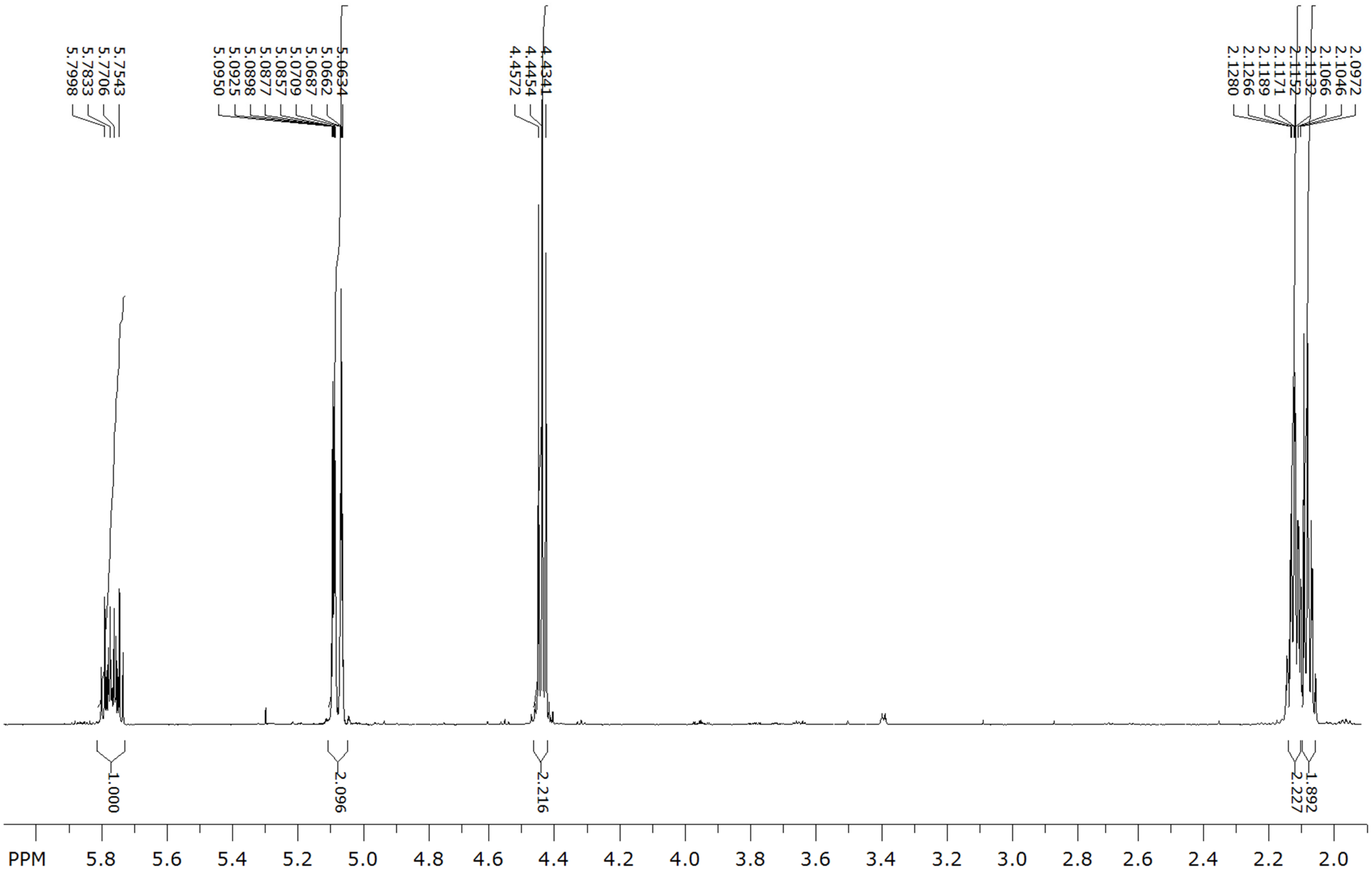

Figure S58. ^{1}H NMR ($CDCl_3$) spectrum of aliphatic part of **9**.

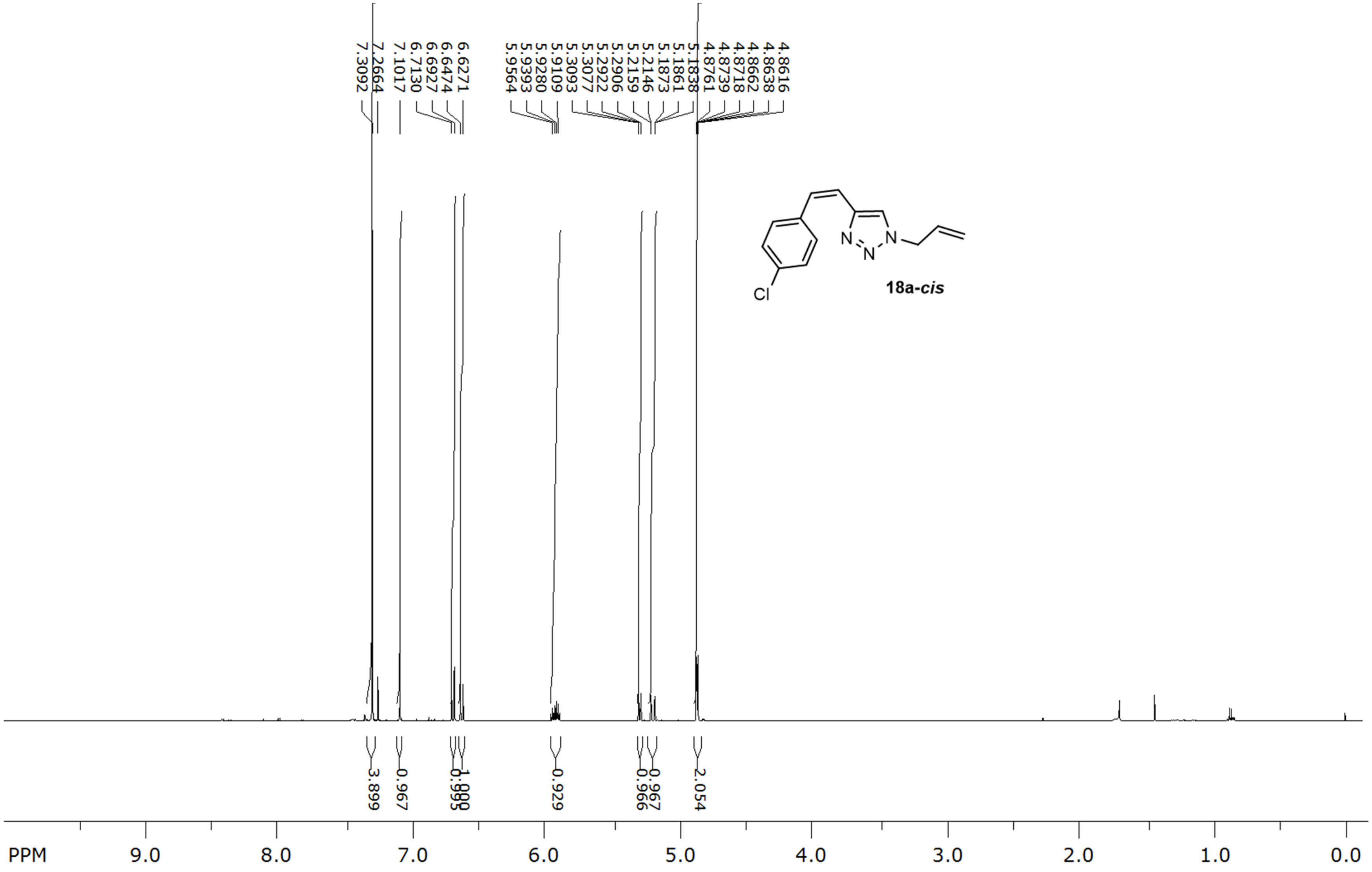

Figure S59. ^{1}H NMR ($CDCl_3$) spectrum of ***cis*-18**.

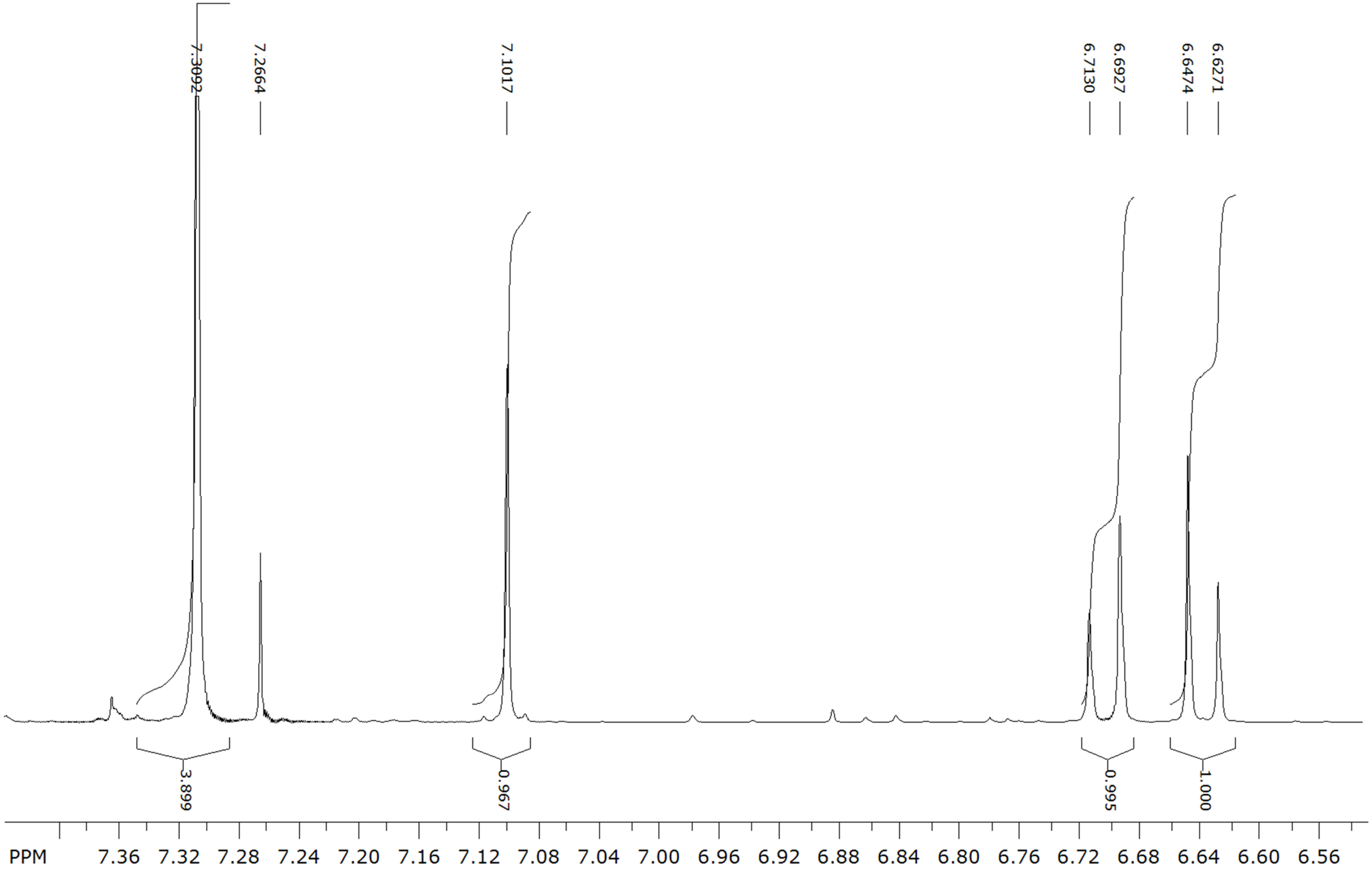

Figure S60. ^{1}H NMR ($CDCl_3$) spectrum of aromatic part of ***cis*-18**.

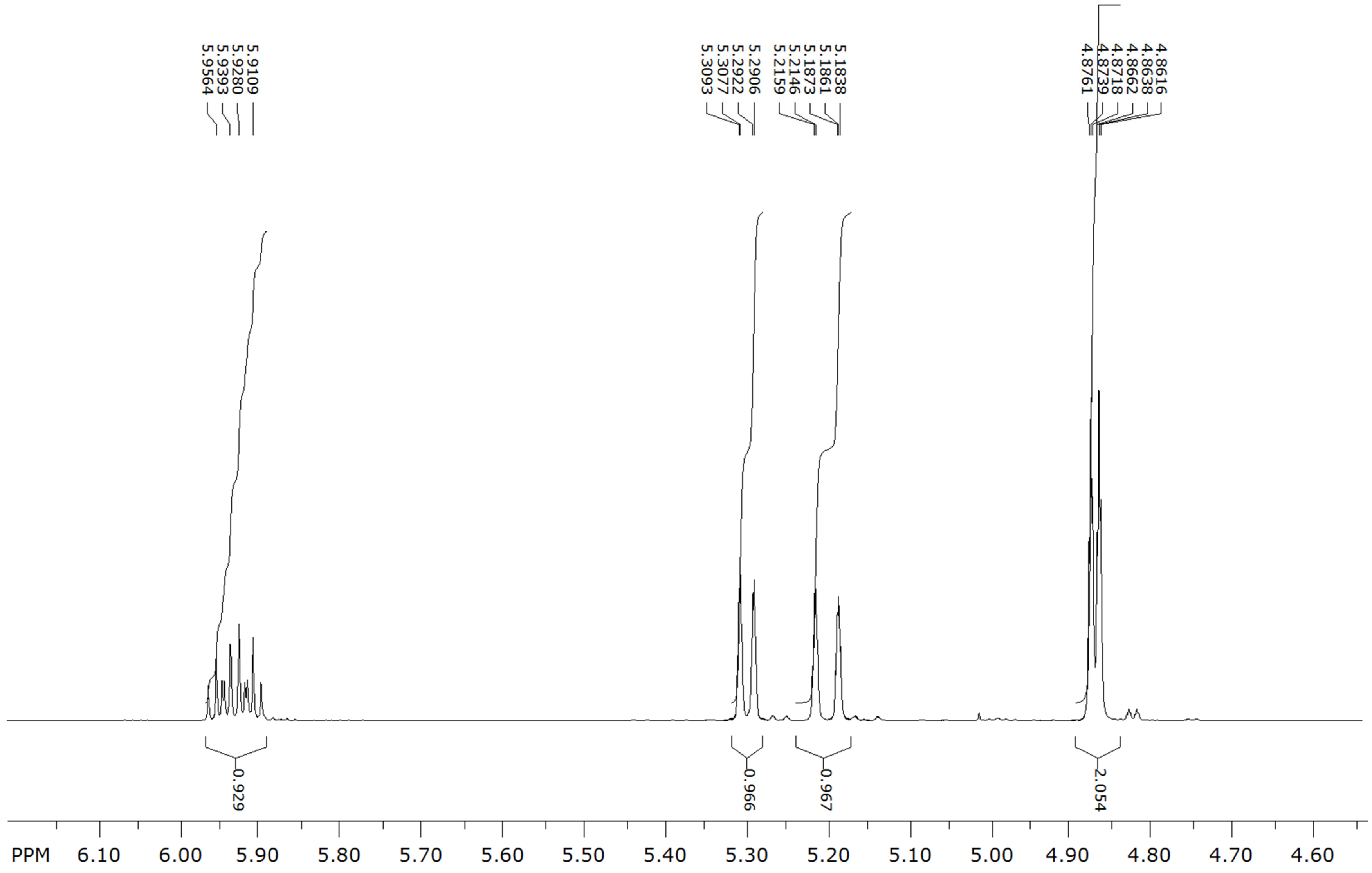

Figure S61. ^{1}H NMR ($CDCl_3$) spectrum of aliphatic part of ***cis*-18**.

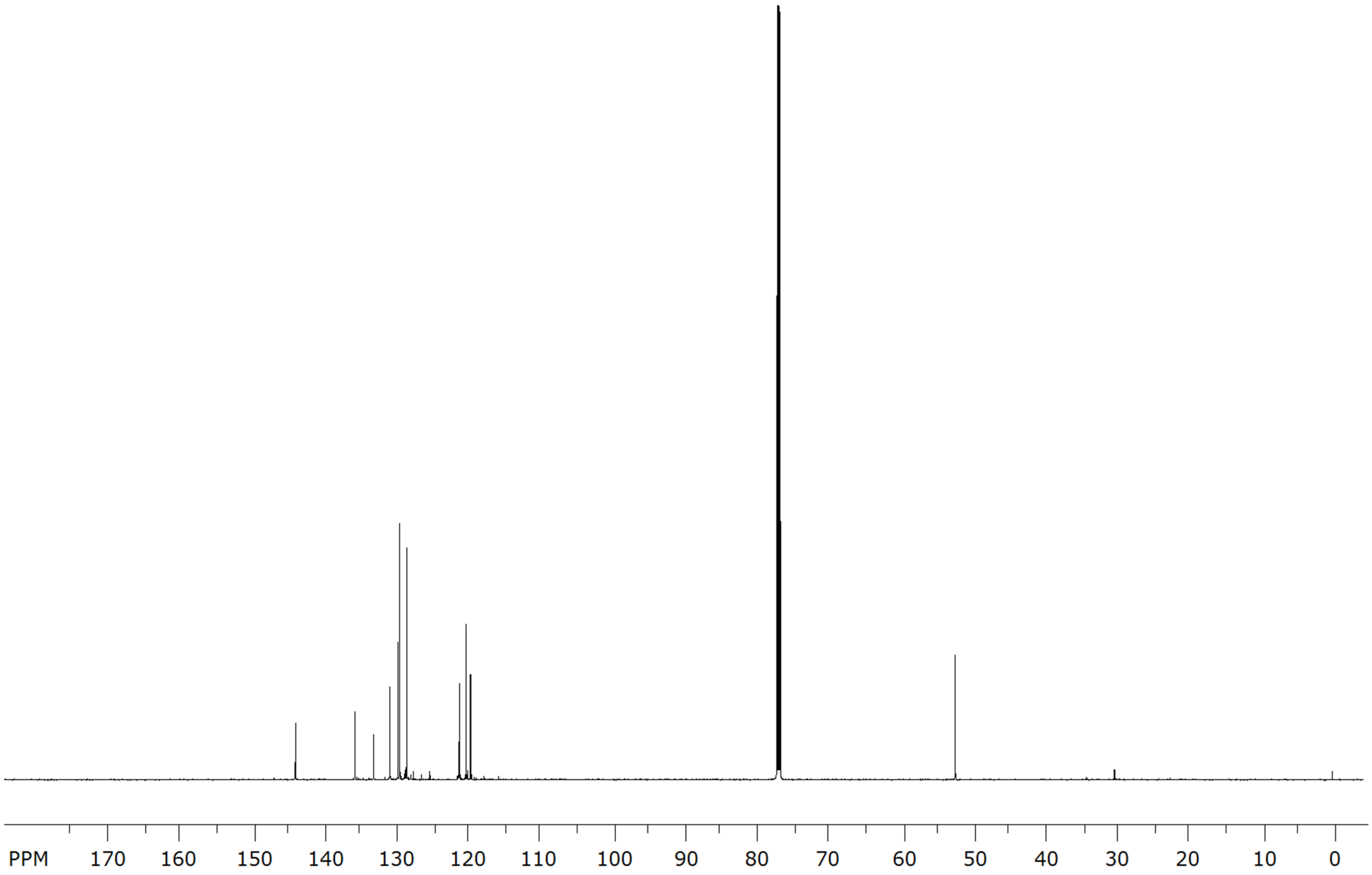

Figure S62. ^{13}C NMR ($CDCl_3$) spectrum of ***cis*-18** (*Spin Works 3.0*).

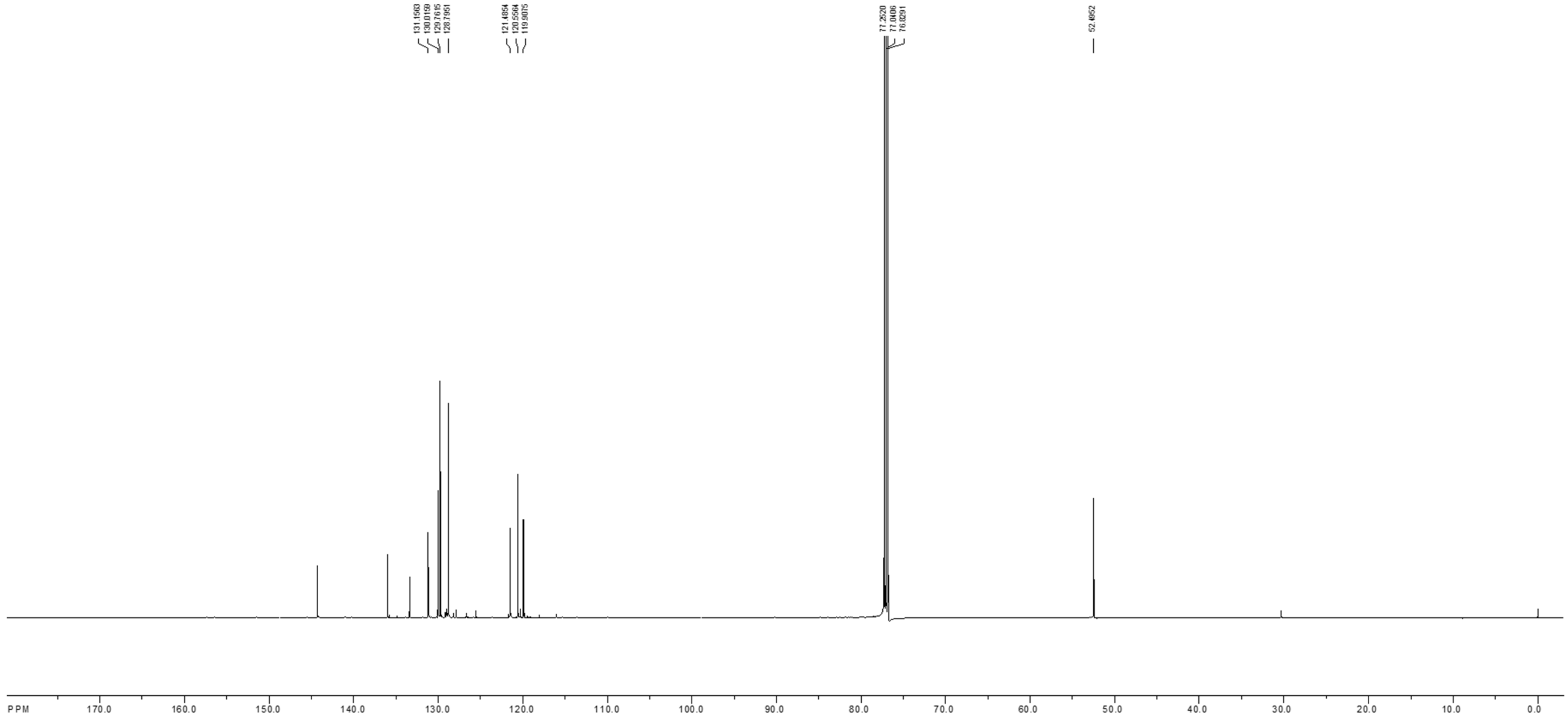

Figure S63. ^{13}C NMR ($CDCl_3$) spectrum of ***cis*-18** (*Spin Works 2.5.5.*).

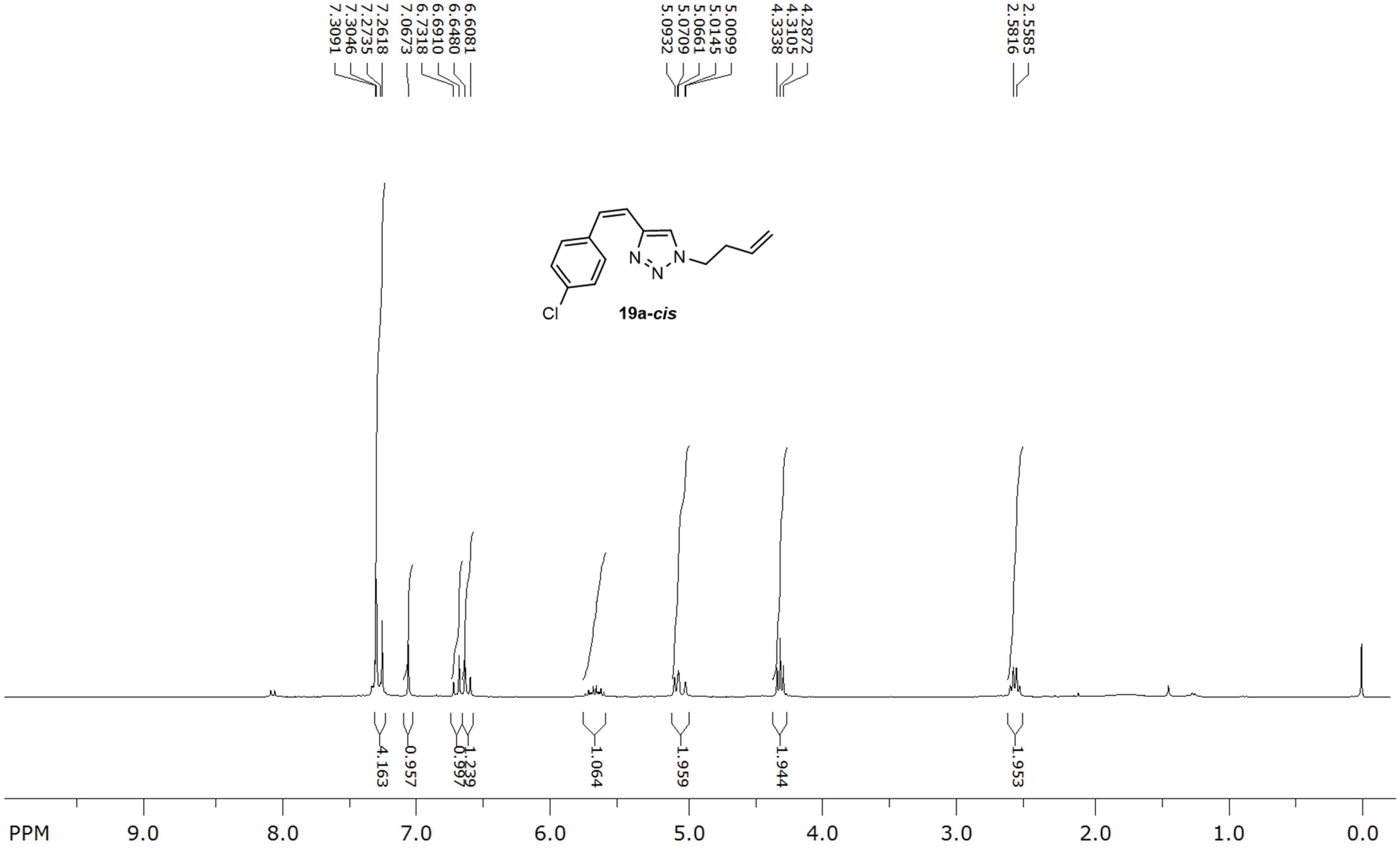

Figure S64. ^{1}H NMR ($CDCl_3$) spectrum of *cis*-**19**.

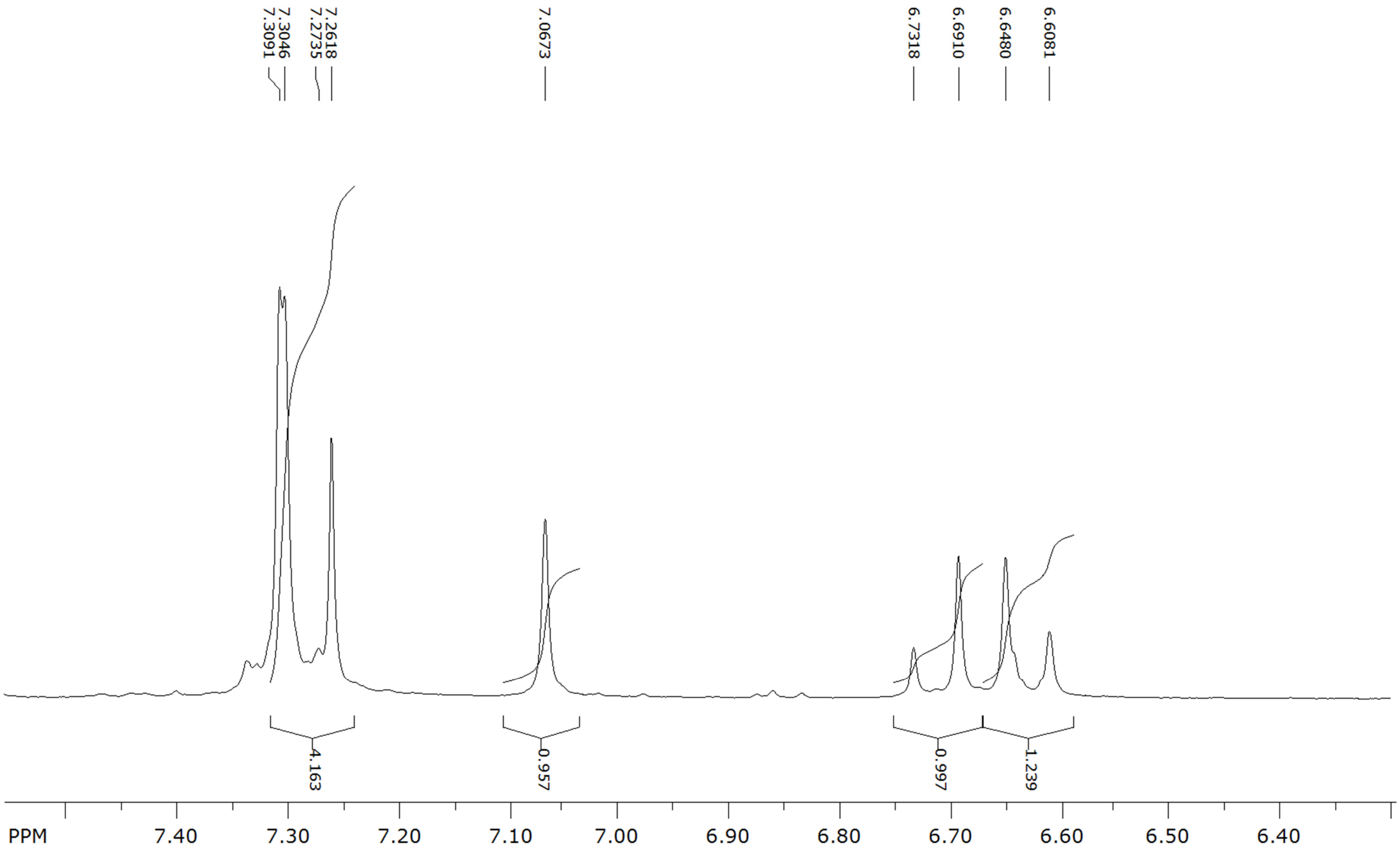

Figure S65. ^{1}H NMR ($CDCl_3$) spectrum of aromatic part of *cis*-**19**.

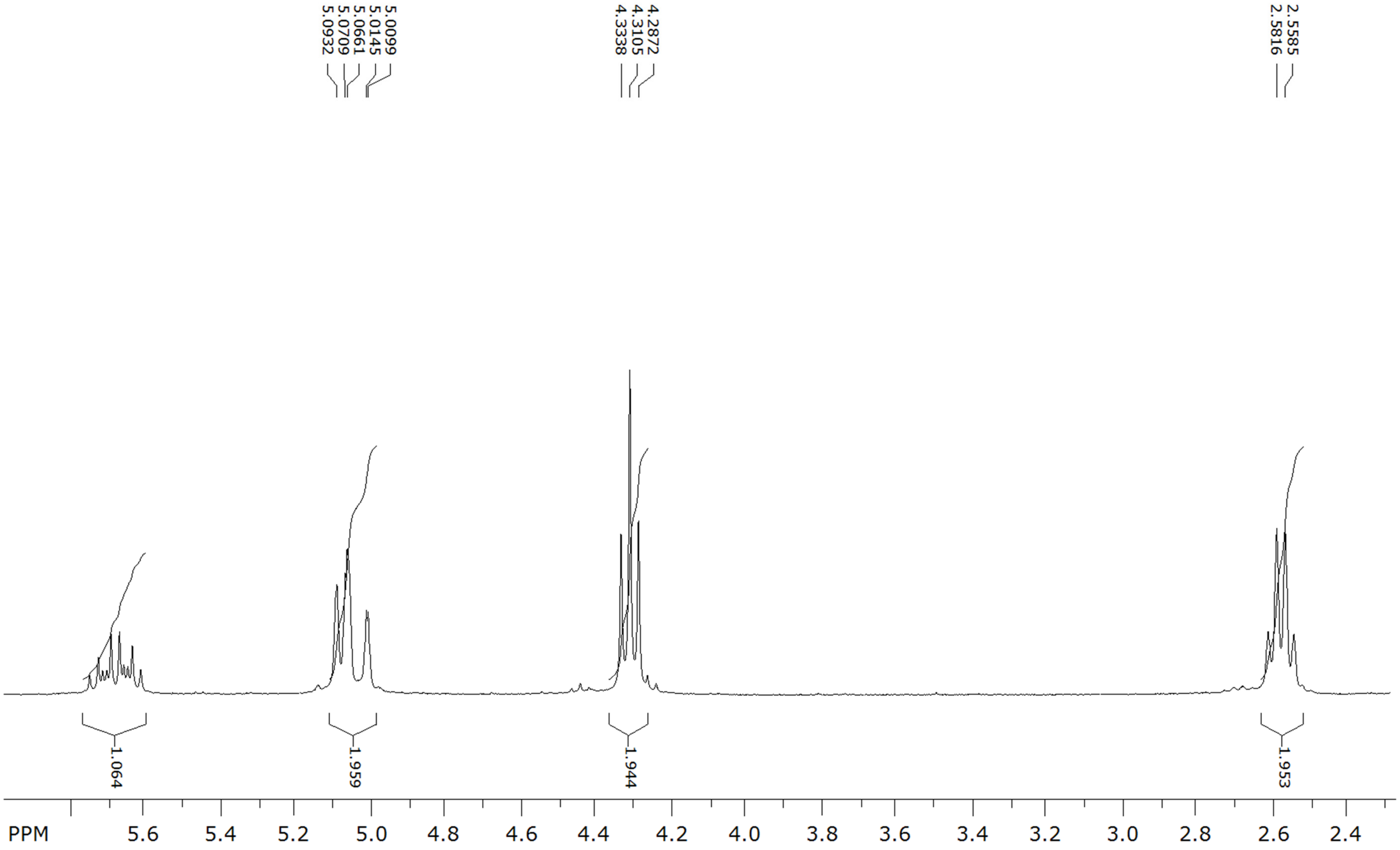

Figure S66. ^{1}H NMR ($CDCl_3$) spectrum of aliphatic part of *cis*-**19**.

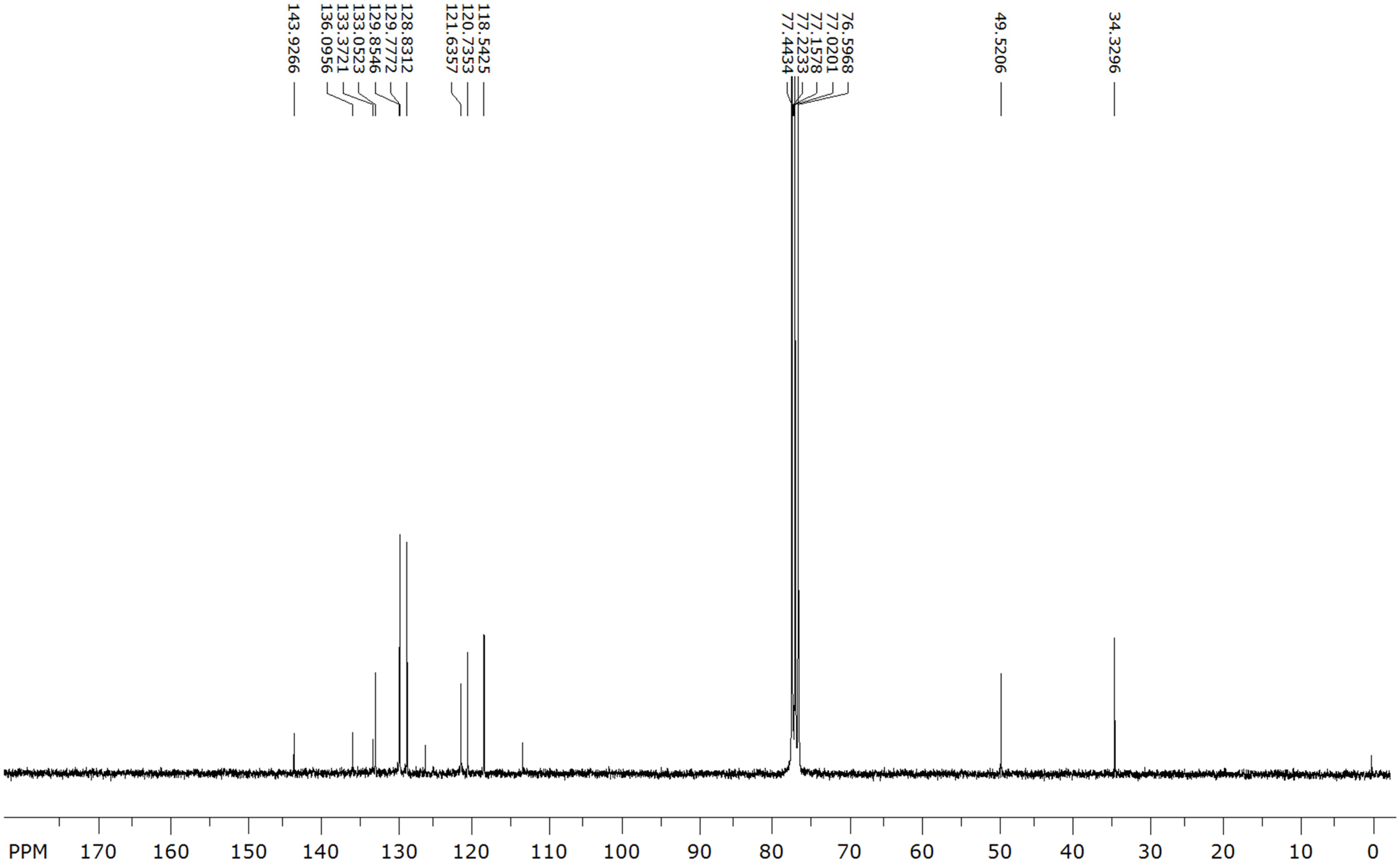

Figure S67. ^{13}C NMR ($CDCl_3$) spectrum of *cis*-**19**.

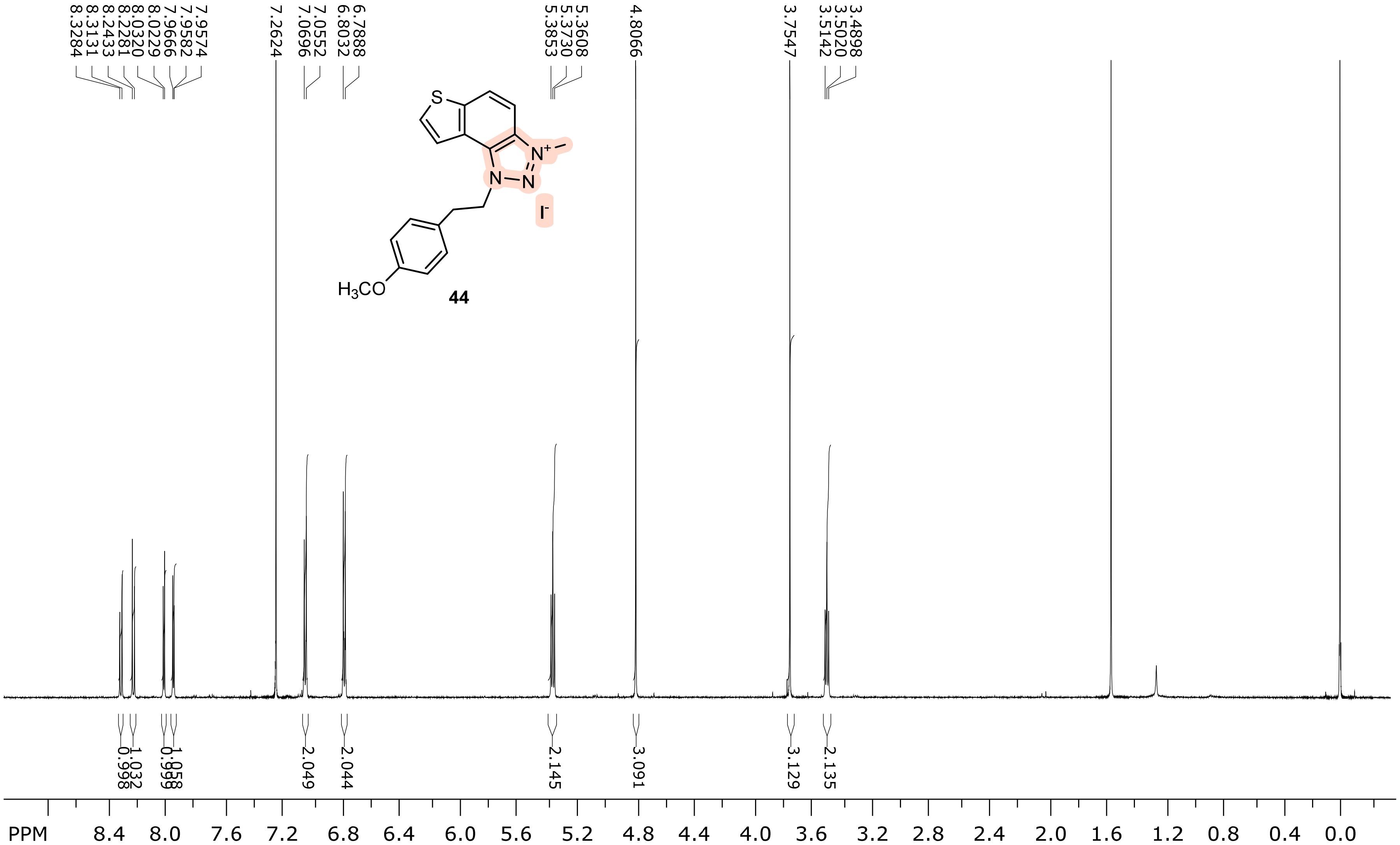

Figure S68. ^{1}H NMR ($CDCl_3$) spectrum of **44**.

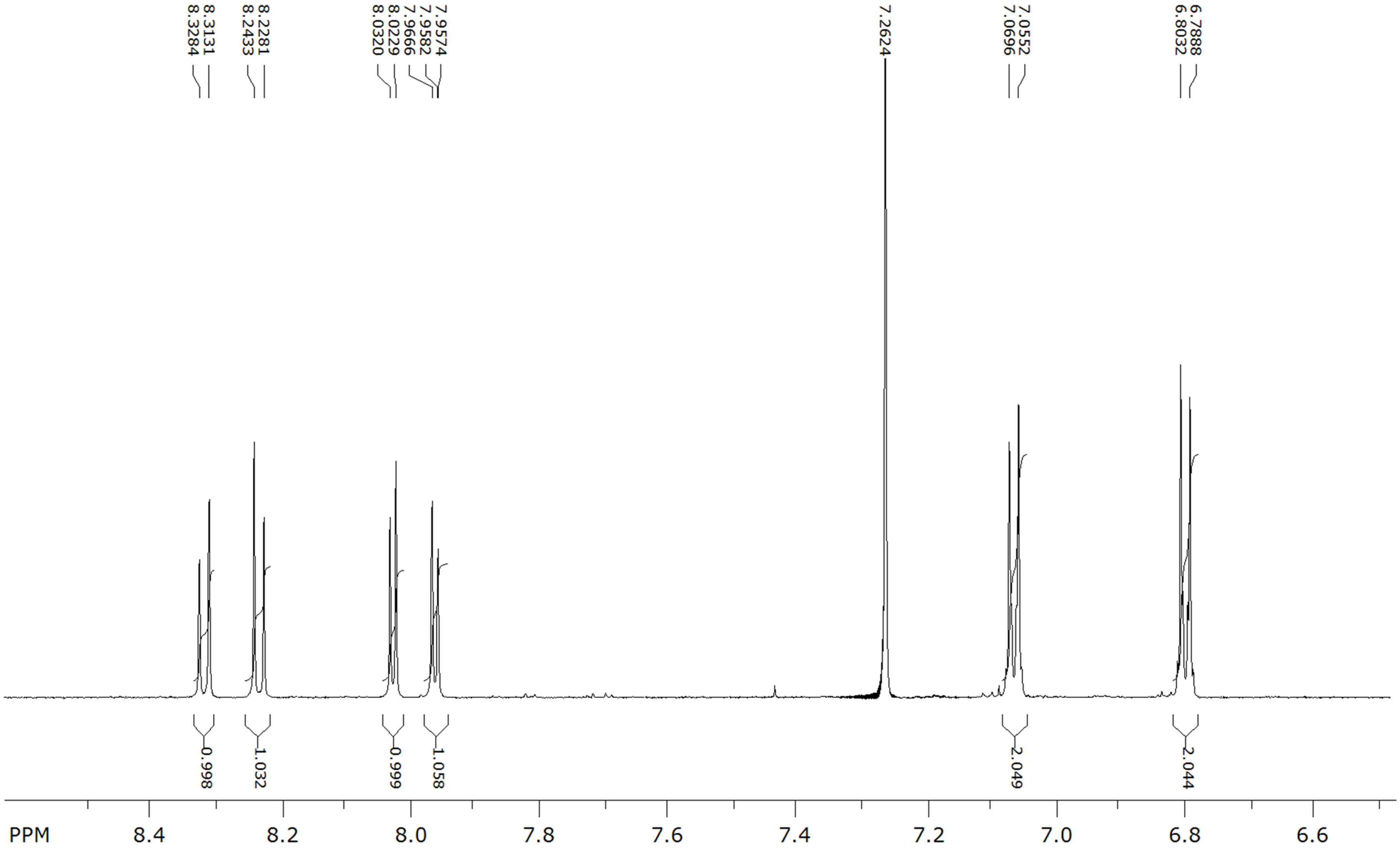

Figure S69. ^{1}H NMR ($CDCl_3$) spectrum of aromatic part of **44**.

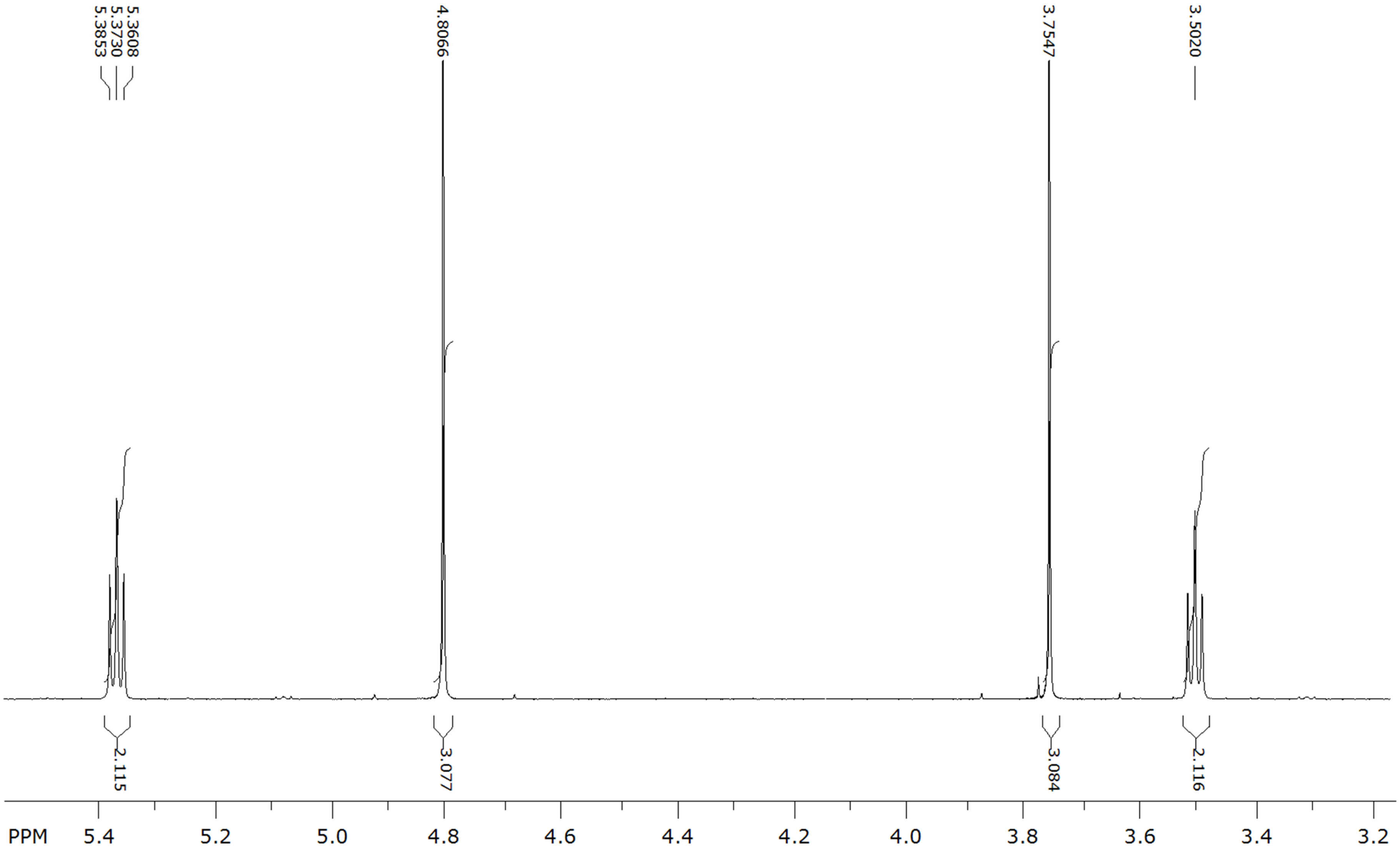

Figure S70. ^{1}H NMR ($CDCl_3$) spectrum of aliphatic part of **44**.

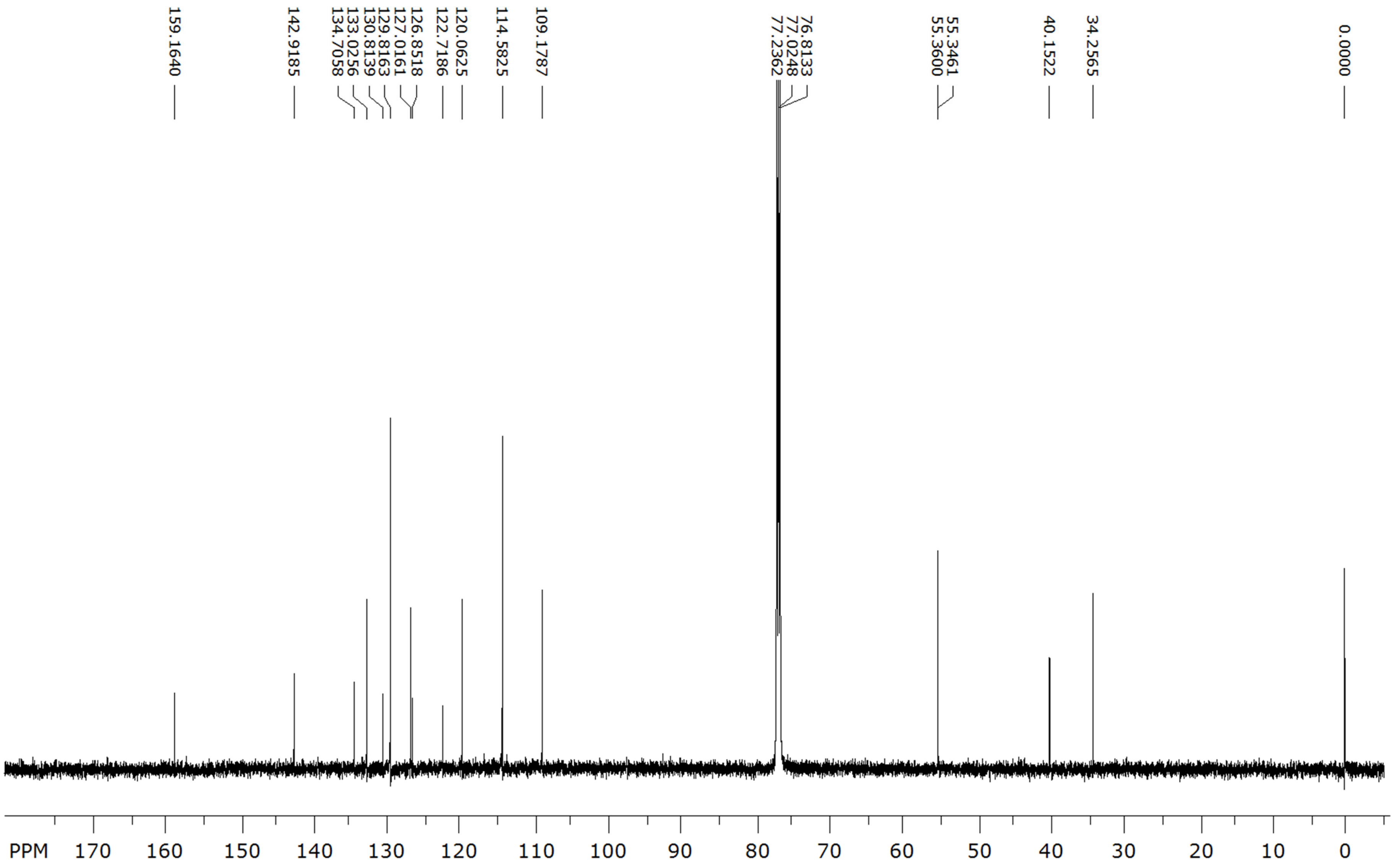

Figure S71. ^{13}C NMR ($CDCl_3$) spectrum of **44**.

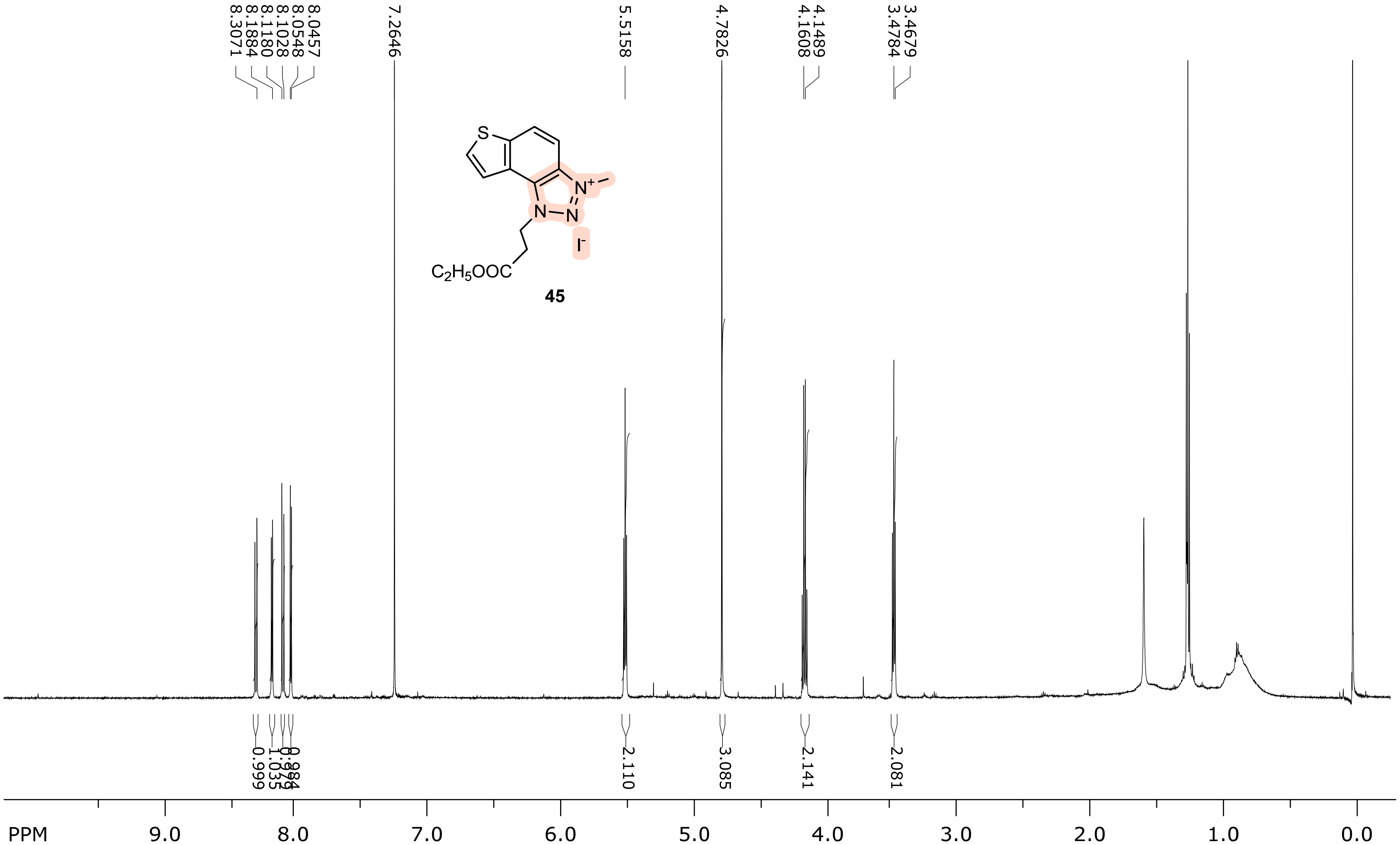

Figure S72. ^{1}H NMR ($CDCl_3$) spectrum of **45**.

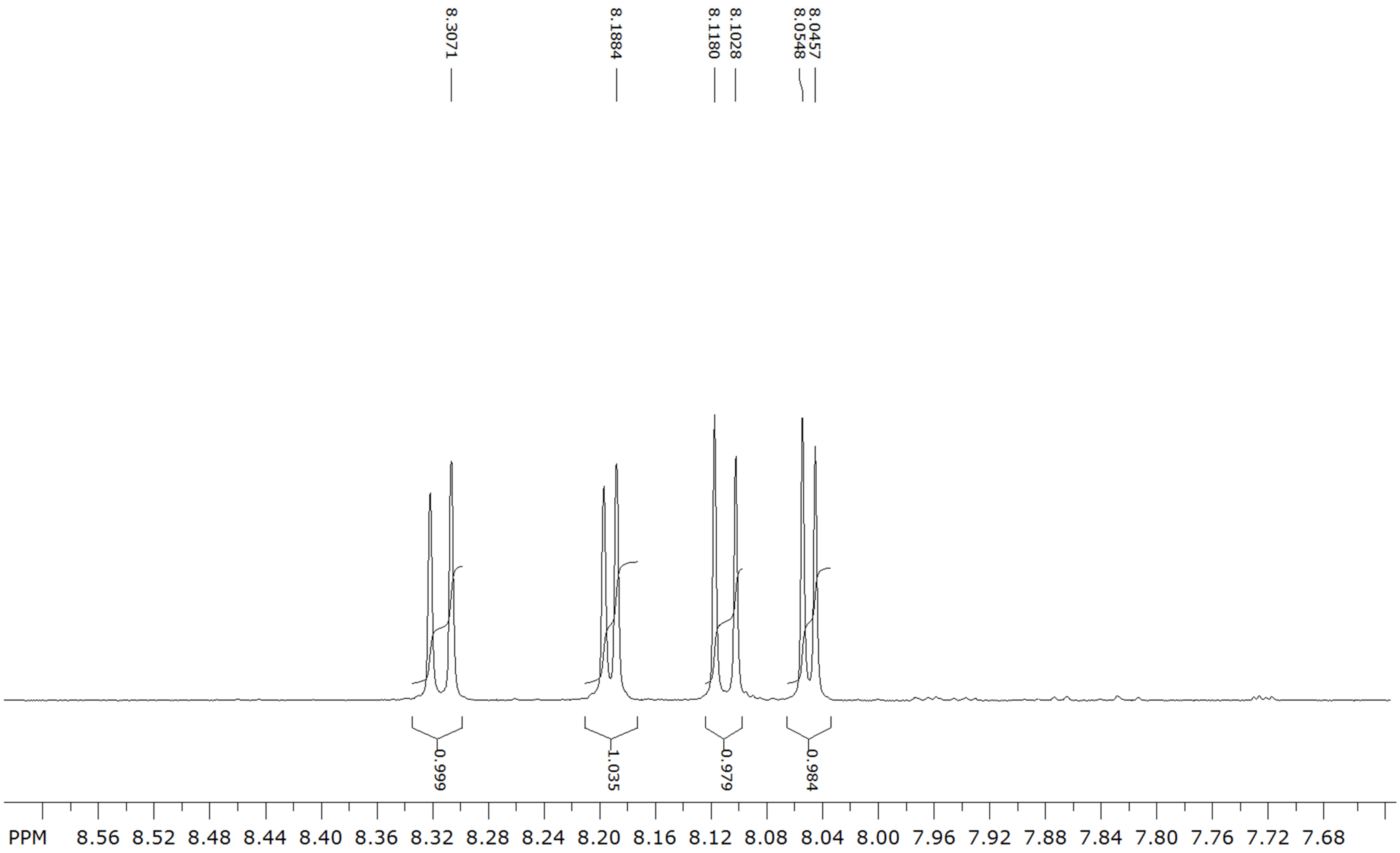

Figure S73. ^{1}H NMR ($CDCl_3$) spectrum of aromatic part of **45**.

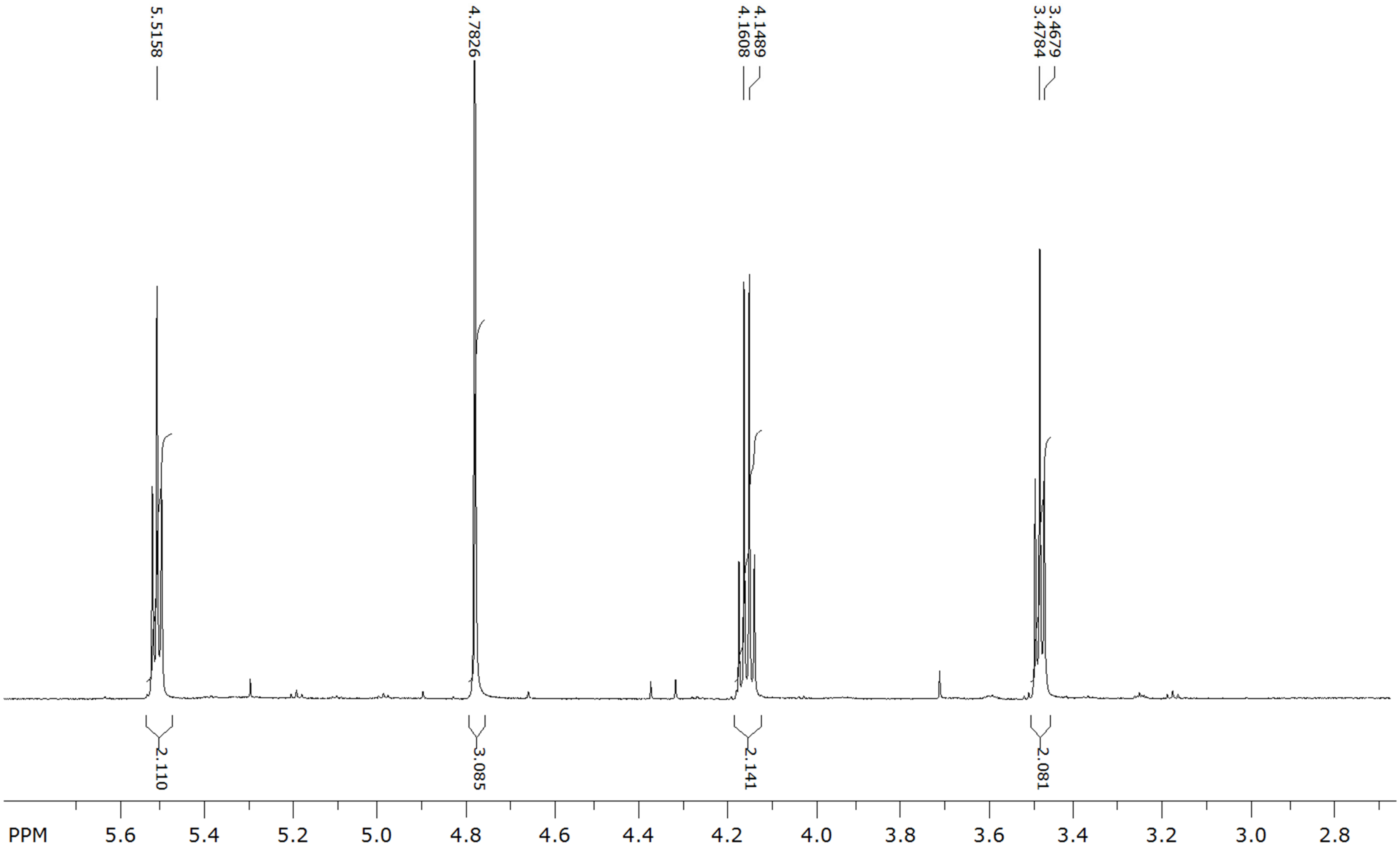

Figure S74. ^{1}H NMR ($CDCl_3$) spectrum of aliphatic part of **45**.

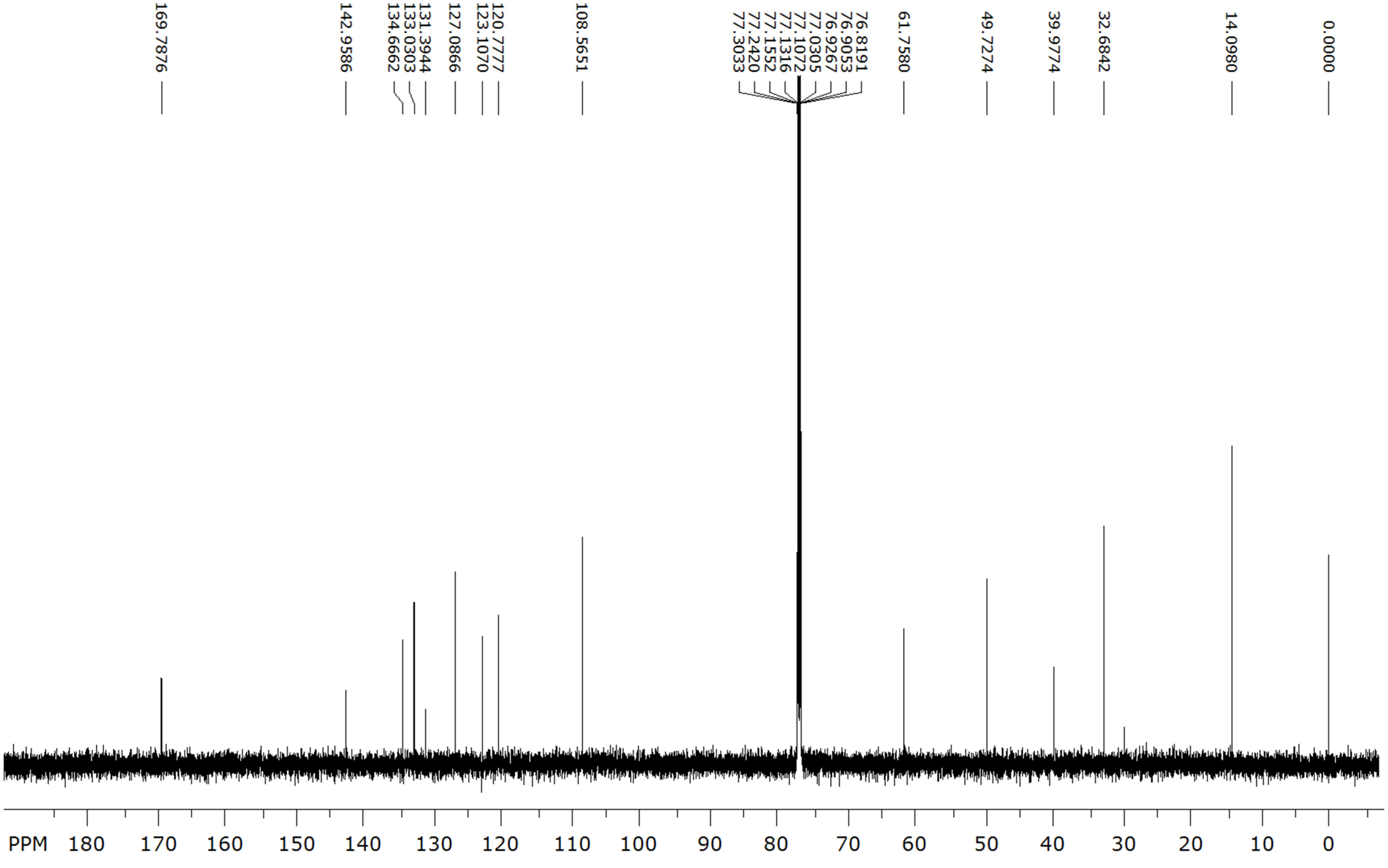

Figure S75. ^{13}C NMR ($CDCl_3$) spectrum of **45**.

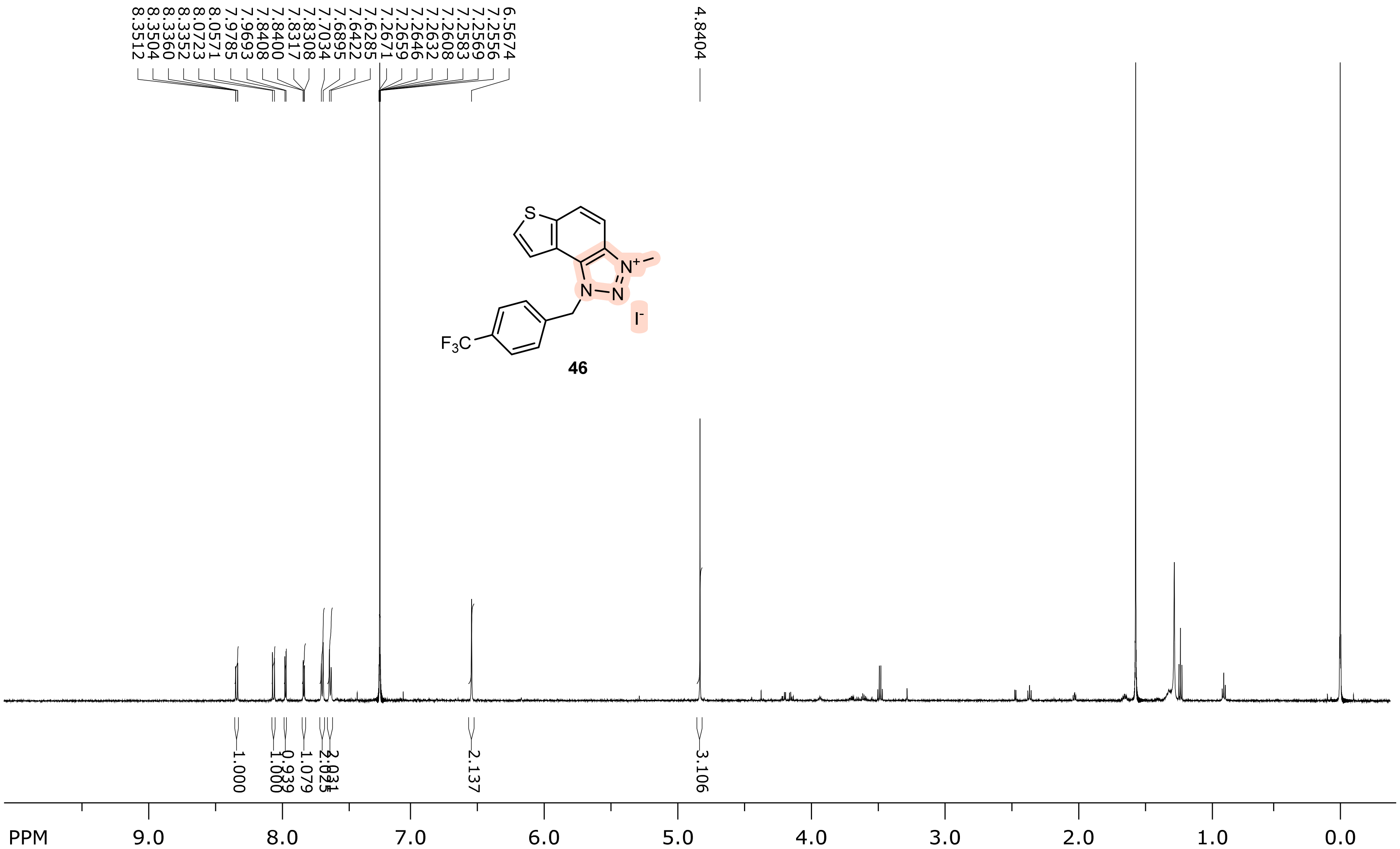

Figure S76. ^{1}H NMR ($CDCl_3$) spectrum of **46**.

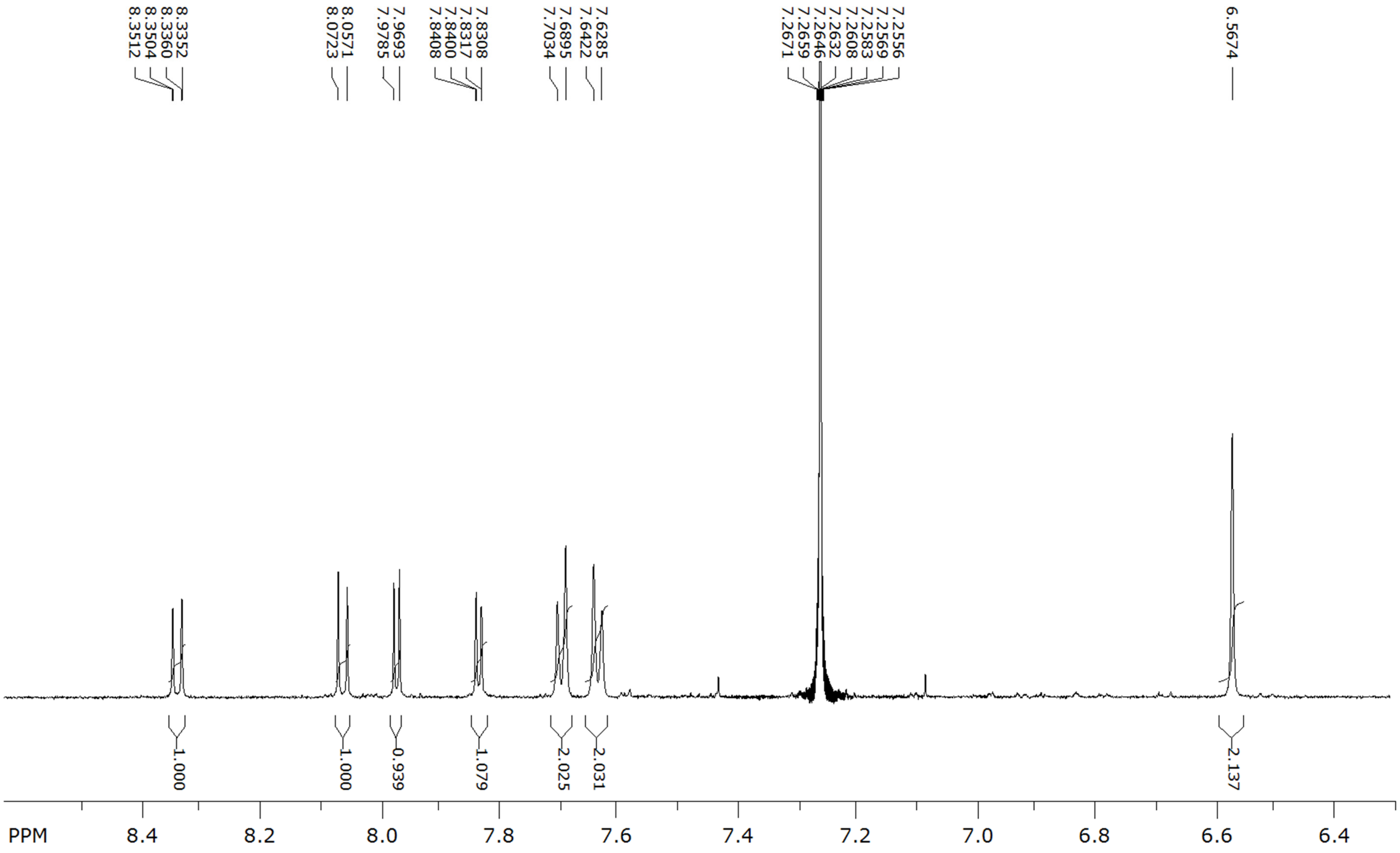

Figure S77. ^{1}H NMR ($CDCl_3$) spectrum of aromatic part of **46**.

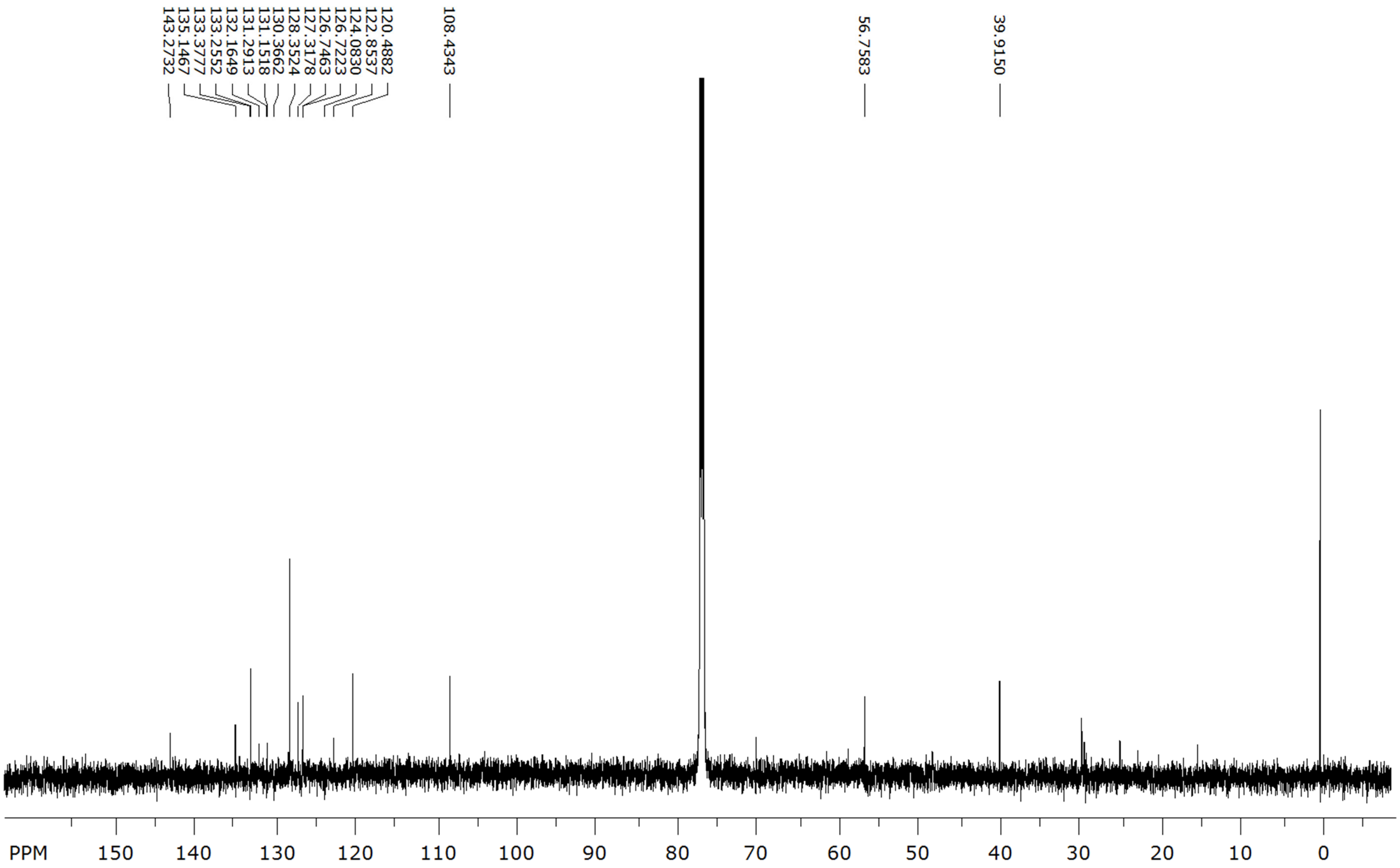

Figure S78. ^{13}C NMR ($CDCl_3$) spectrum of **46**.

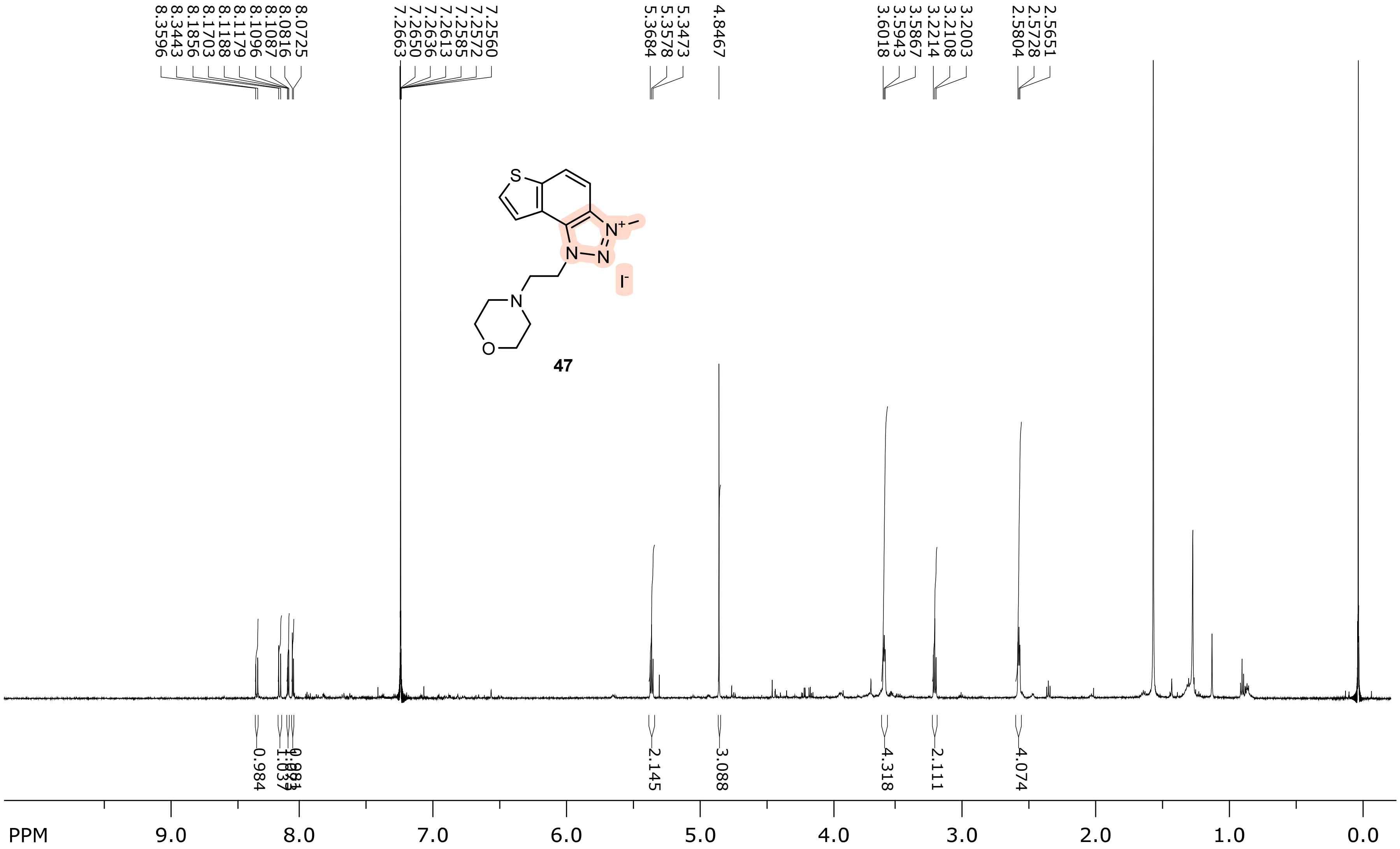

Figure S79. ^{1}H NMR ($CDCl_3$) spectrum of **47**.

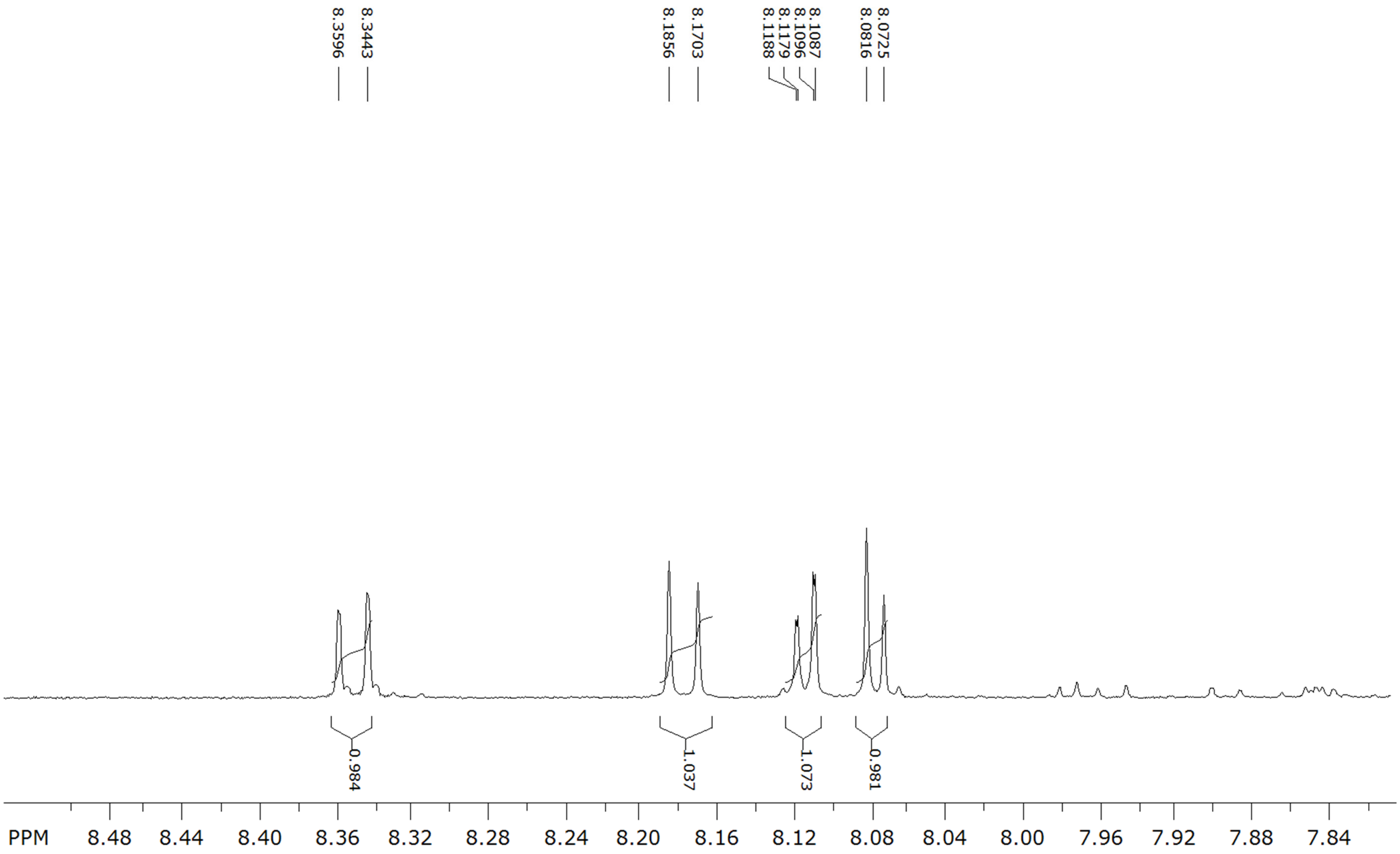

Figure S80. ^{1}H NMR ($CDCl_3$) spectrum of aromatic part of **47**.

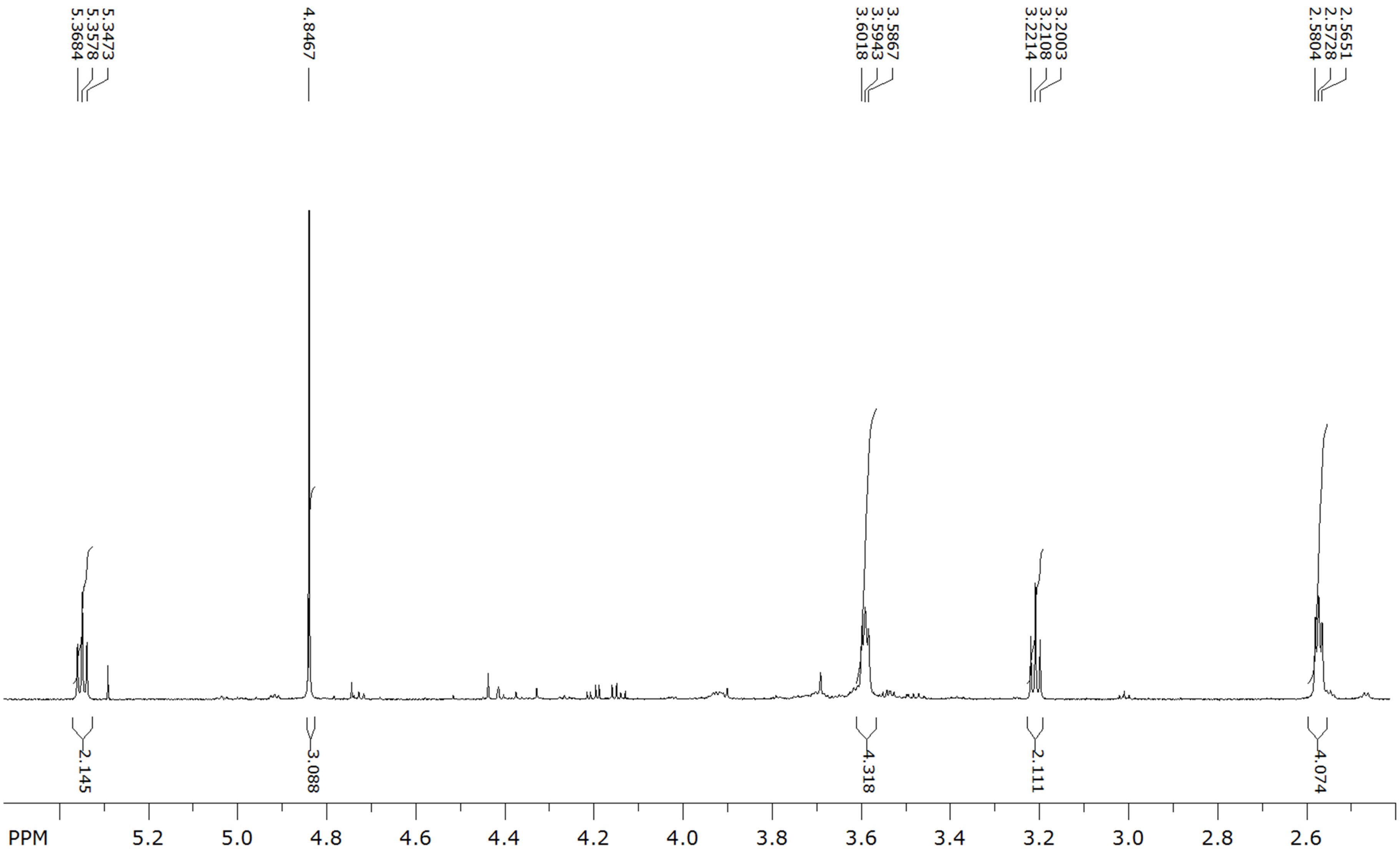

Figure S81. ^{1}H NMR ($CDCl_3$) spectrum of aliphatic part of **47**.

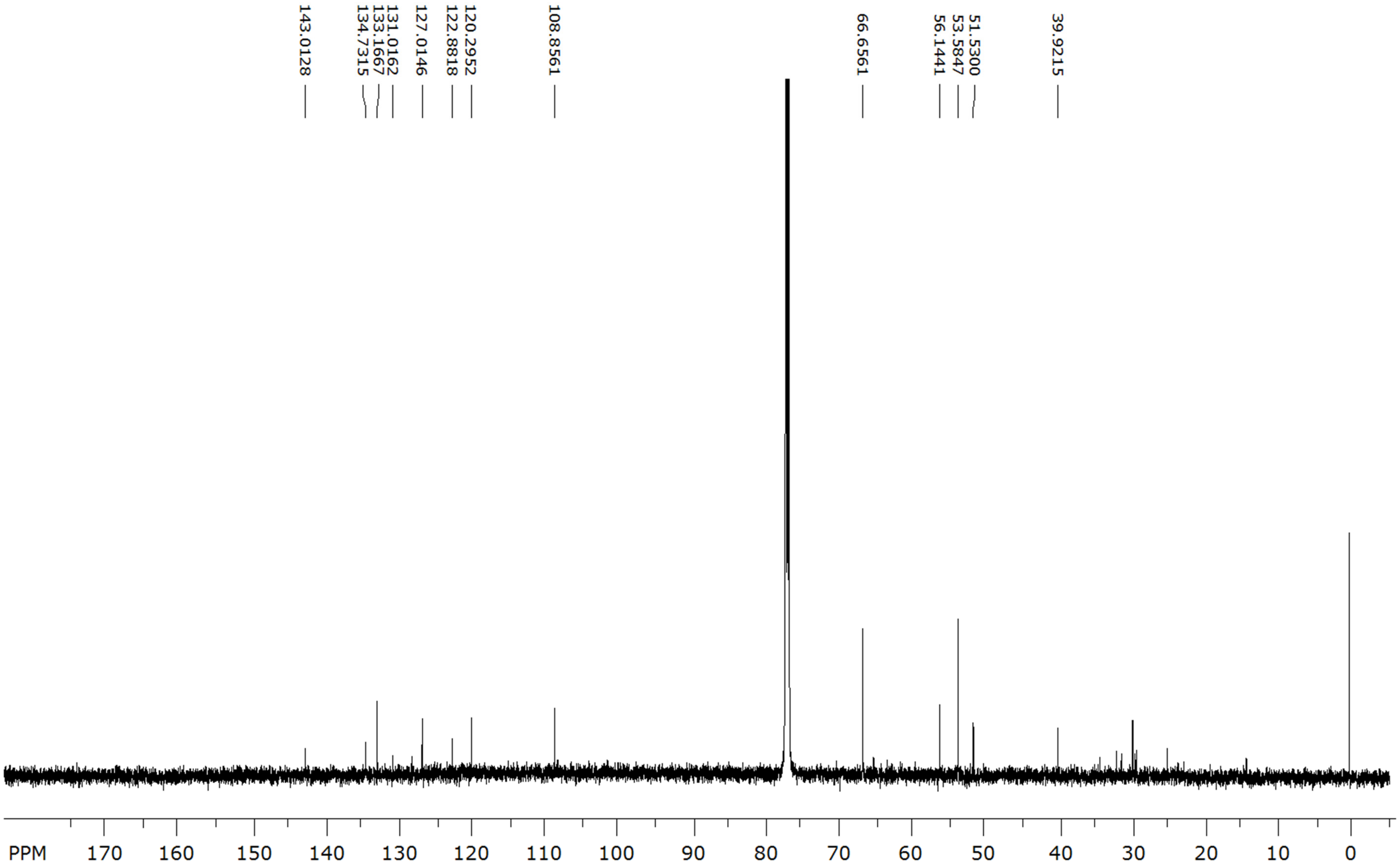

Figure S82. ^{13}C NMR ($CDCl_3$) spectrum of **47**.

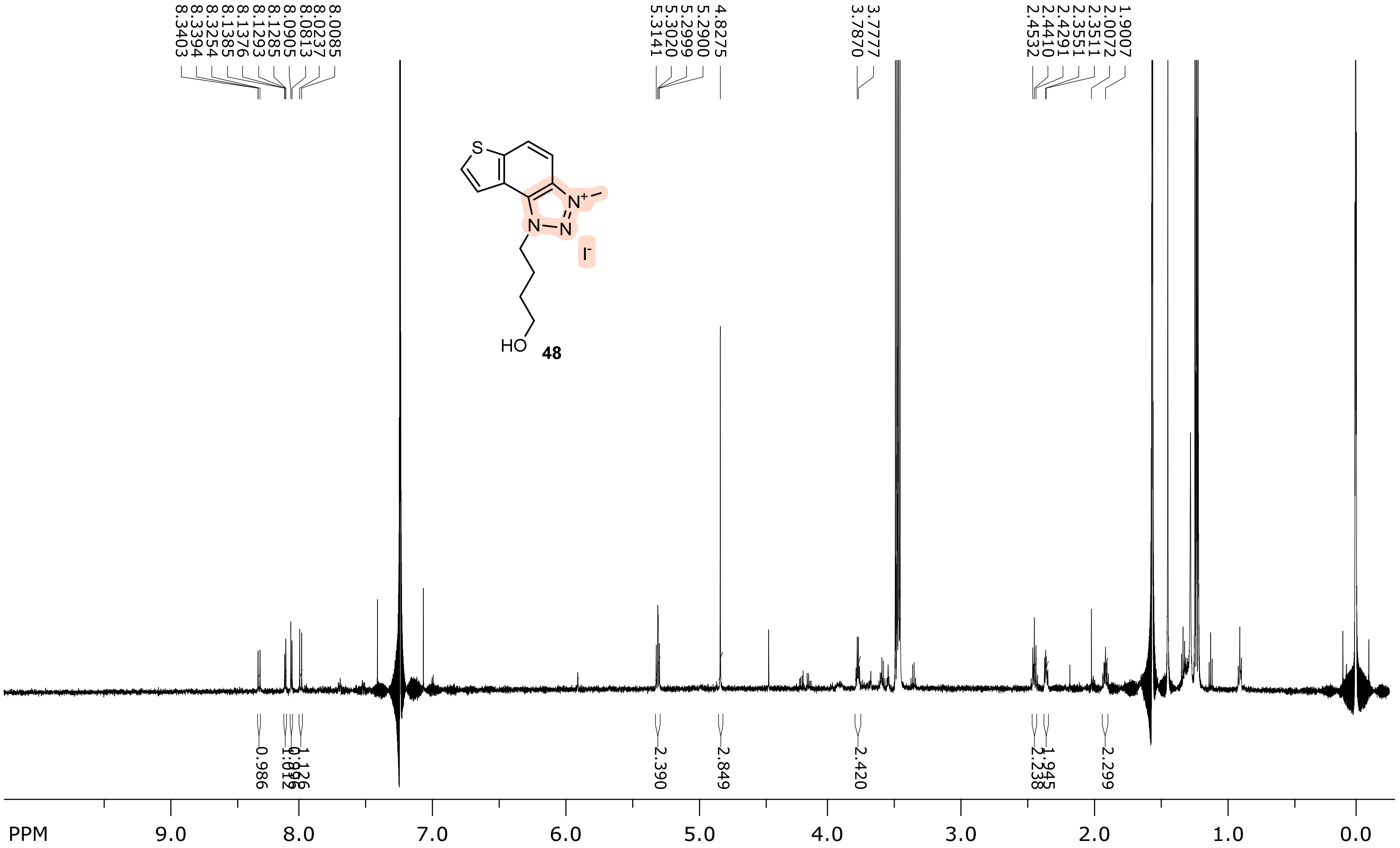

Figure S83. ^{1}H NMR (CDCl$_3$) spectrum of **48**.

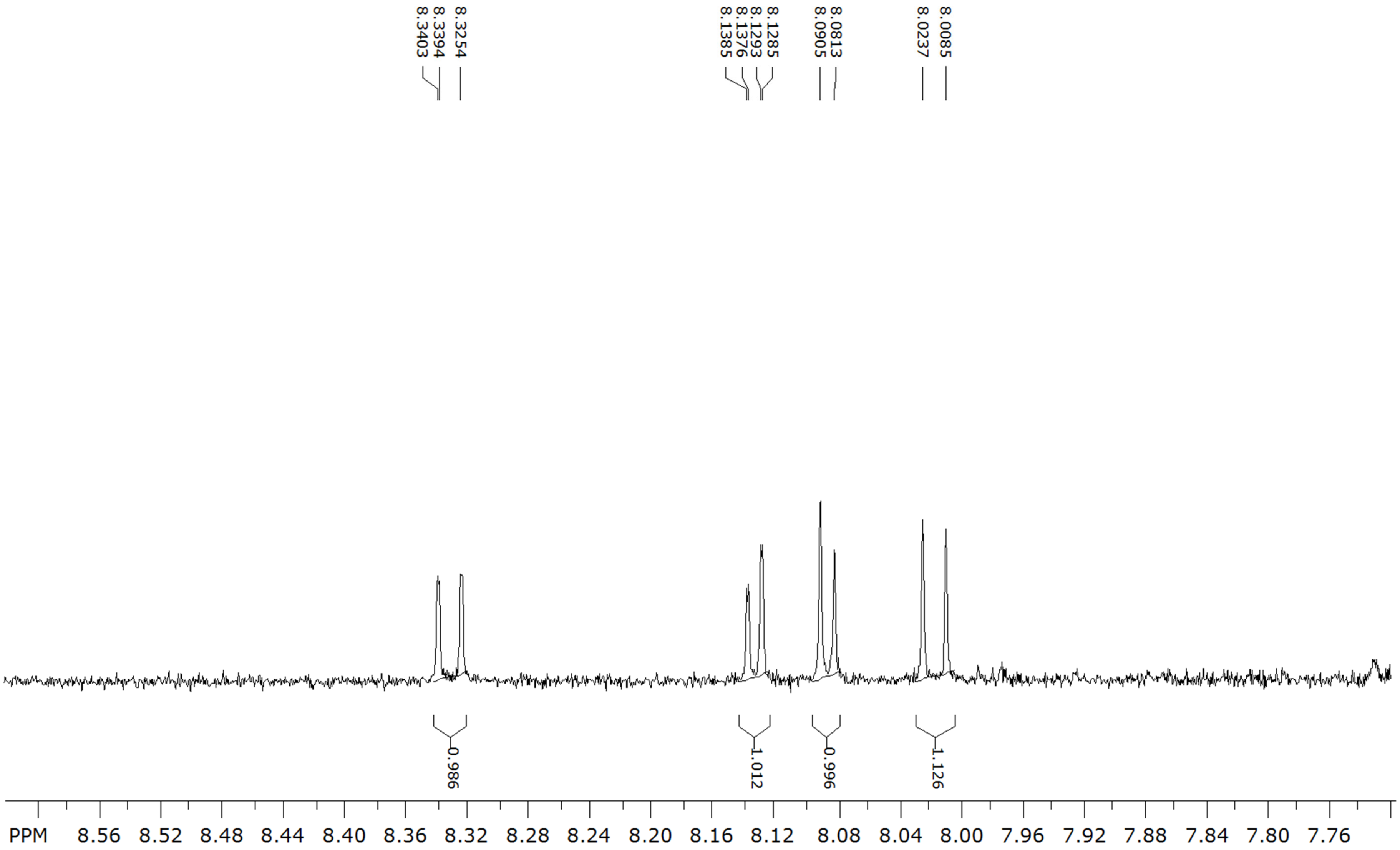

Figure S84. ^{1}H NMR ($CDCl_3$) spectrum of aromatic part of **48**.

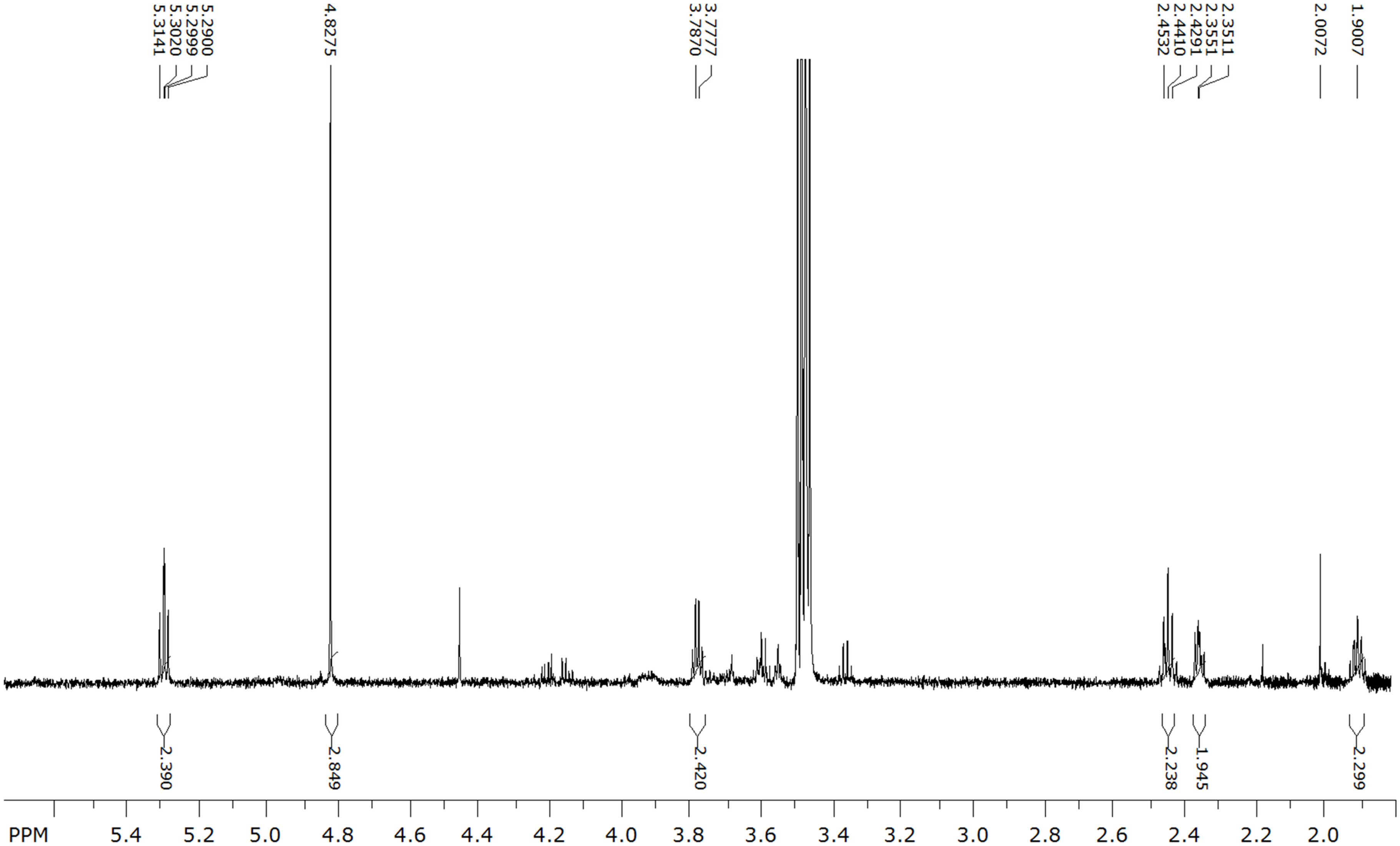

Figure S85. ^{1}H NMR ($CDCl_3$) spectrum of aliphatic part of **48**.

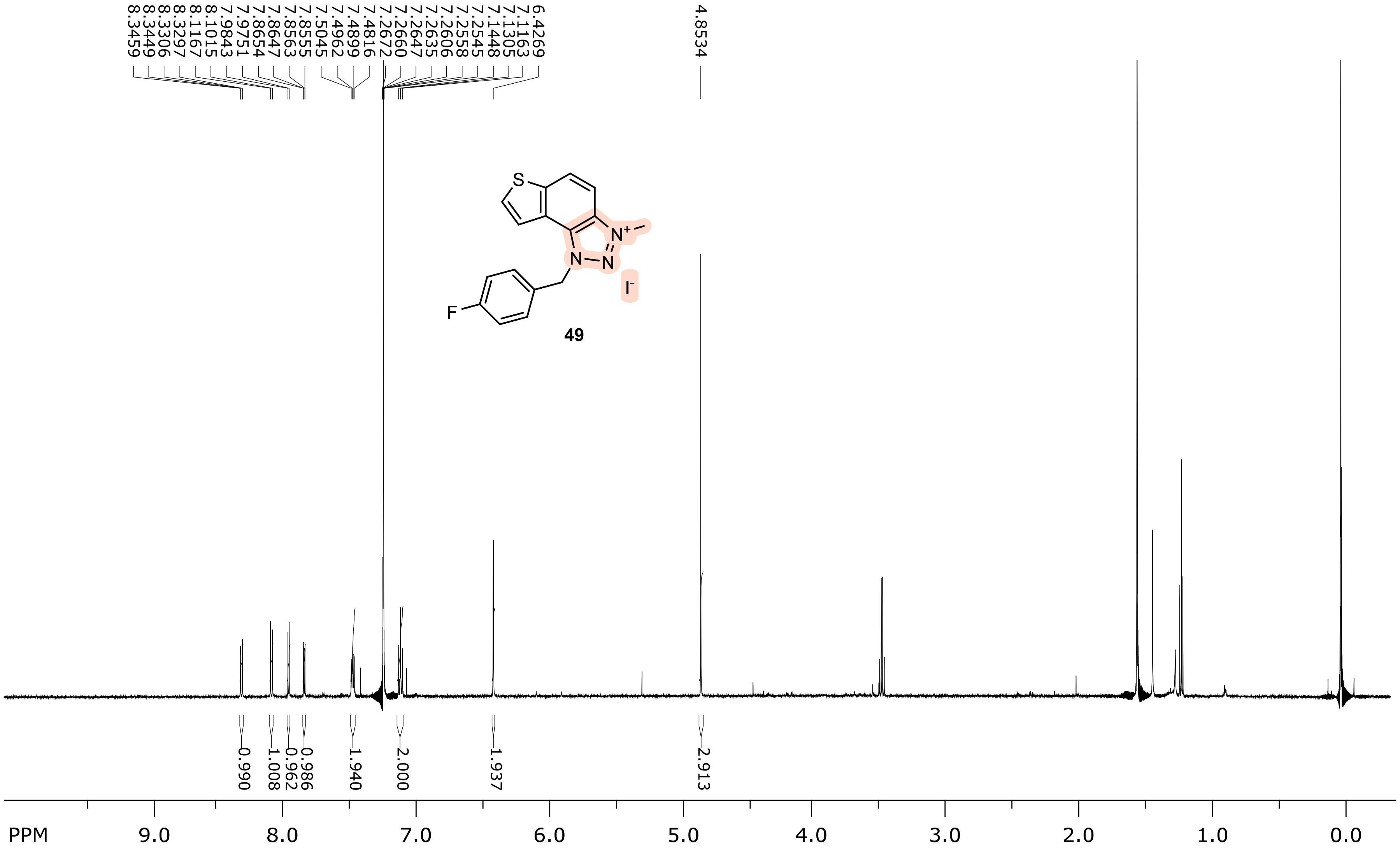

Figure S86. ^{1}H NMR ($CDCl_3$) spectrum of **49**.

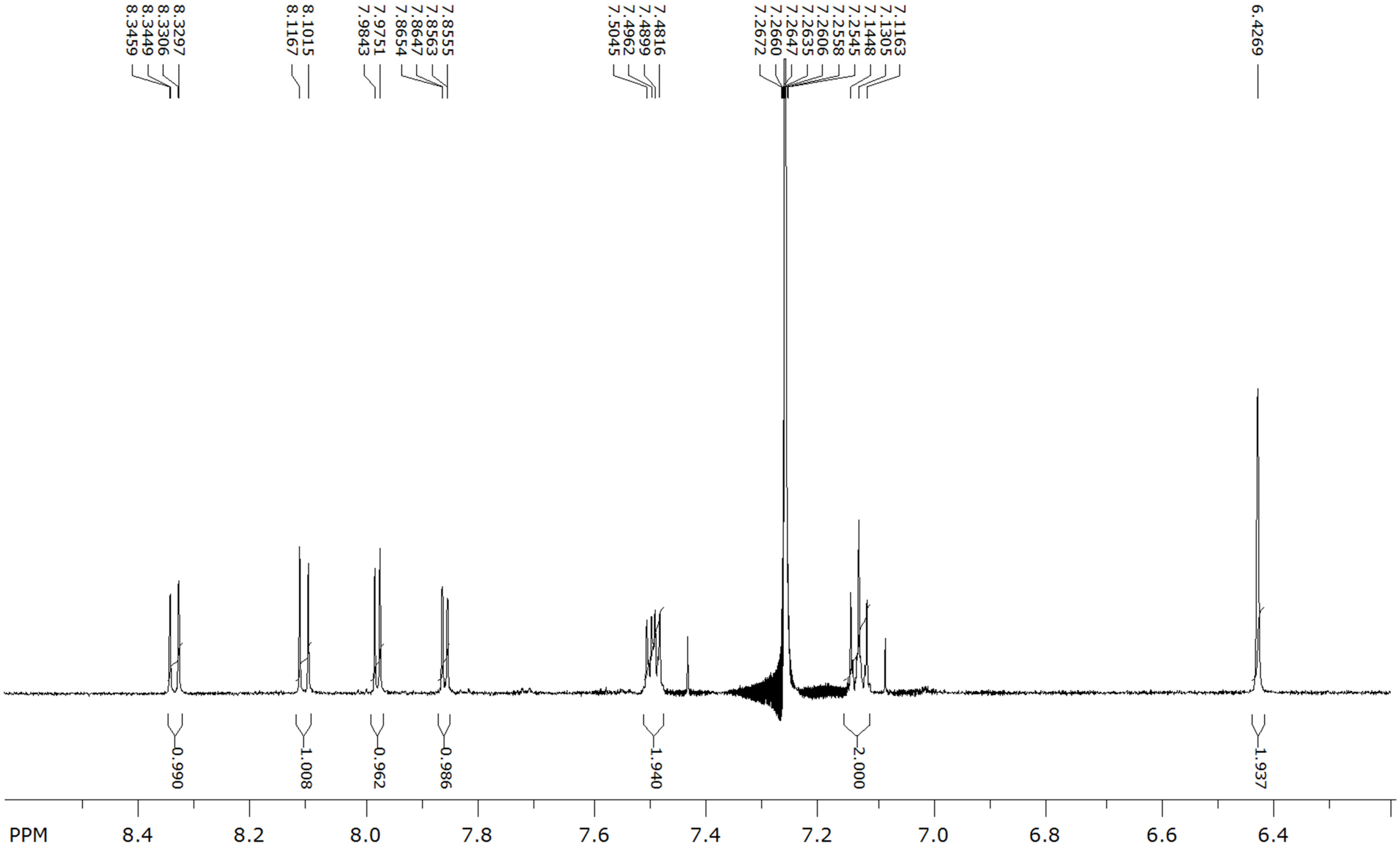

Figure S87. ^{1}H NMR ($CDCl_3$) spectrum of aromatic part of **49**.

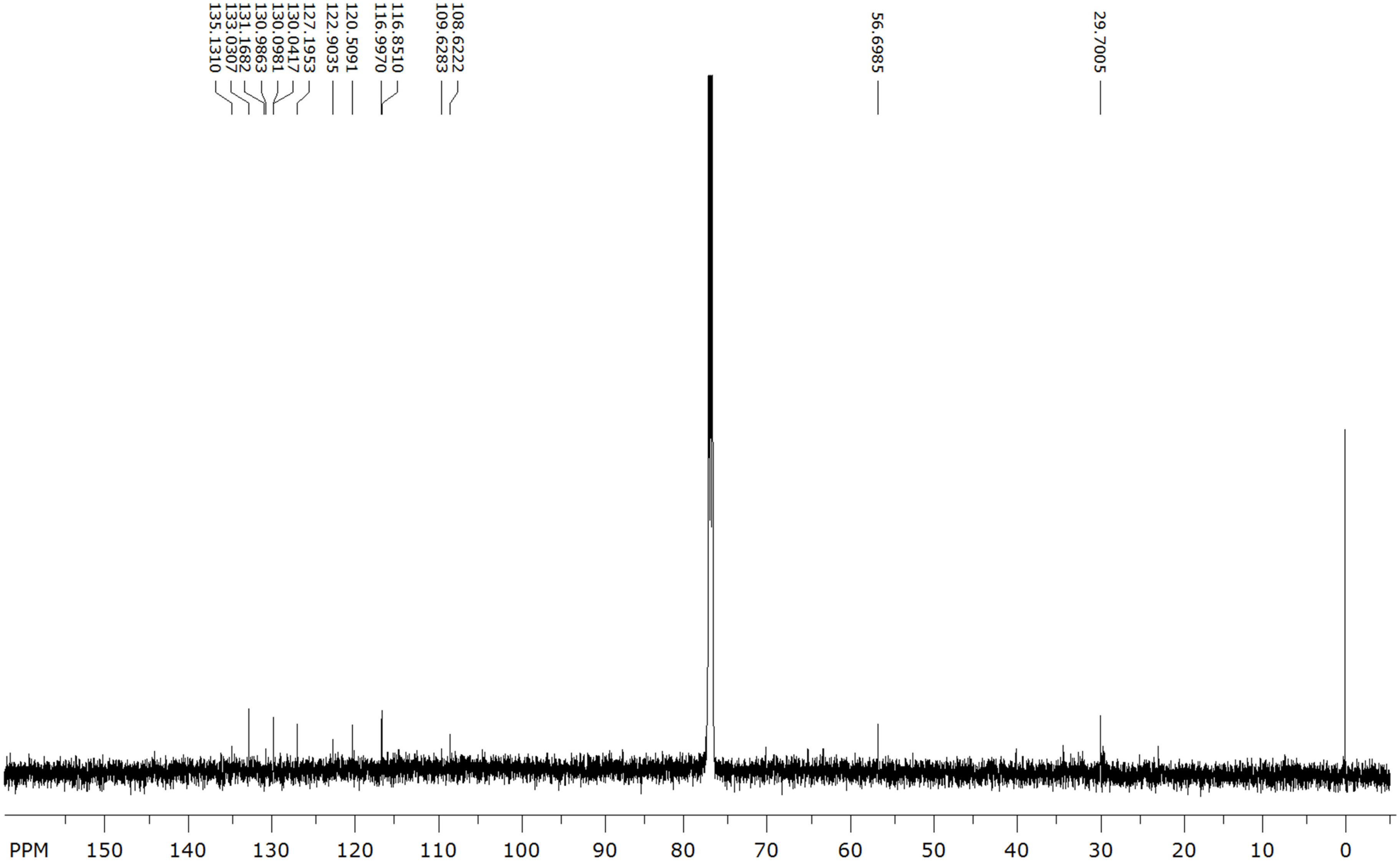

Figure S88. ^{13}C NMR ($CDCl_3$) spectrum of **49**.

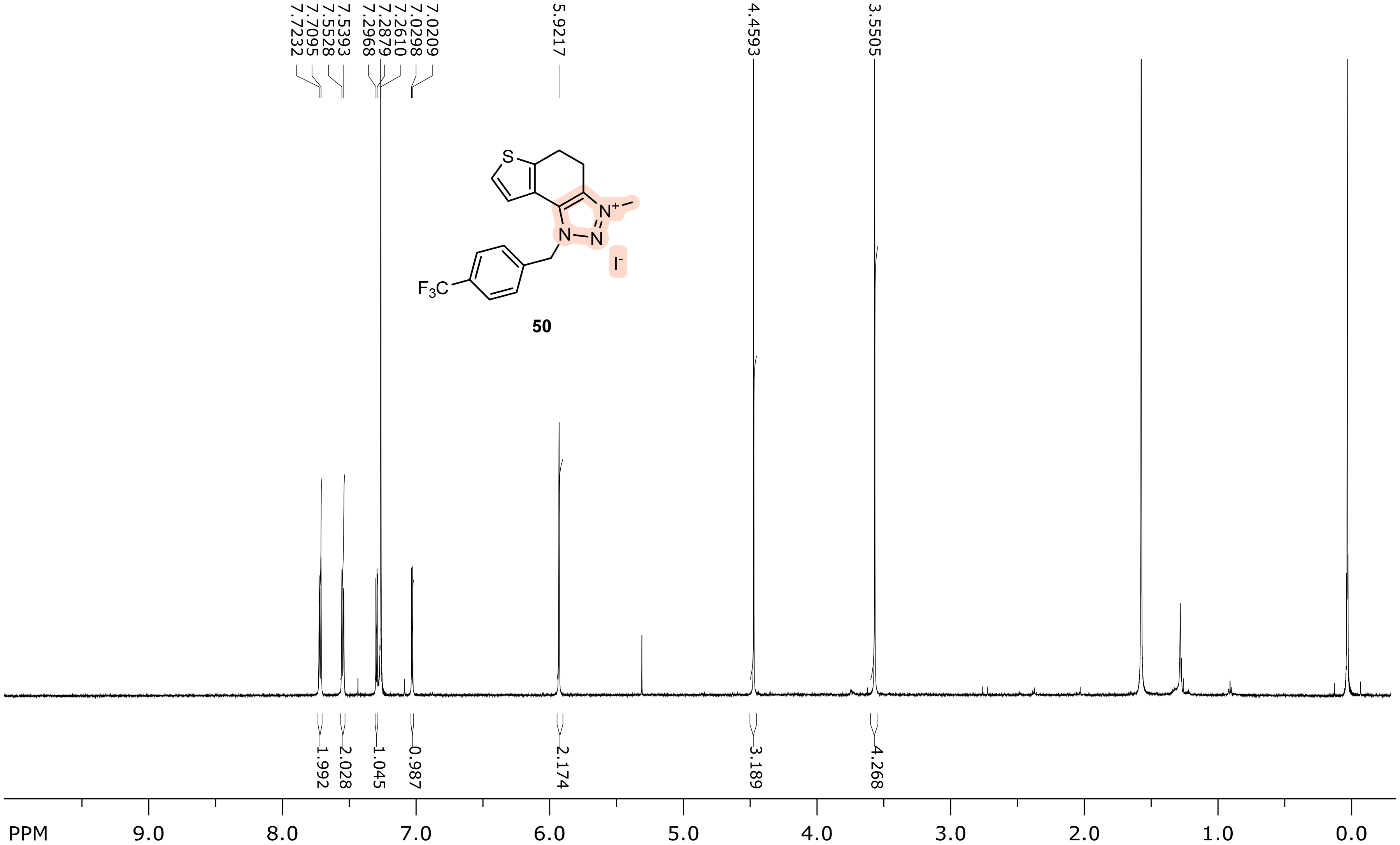

Figure S89. ^{1}H NMR ($CDCl_3$) spectrum of **50**.

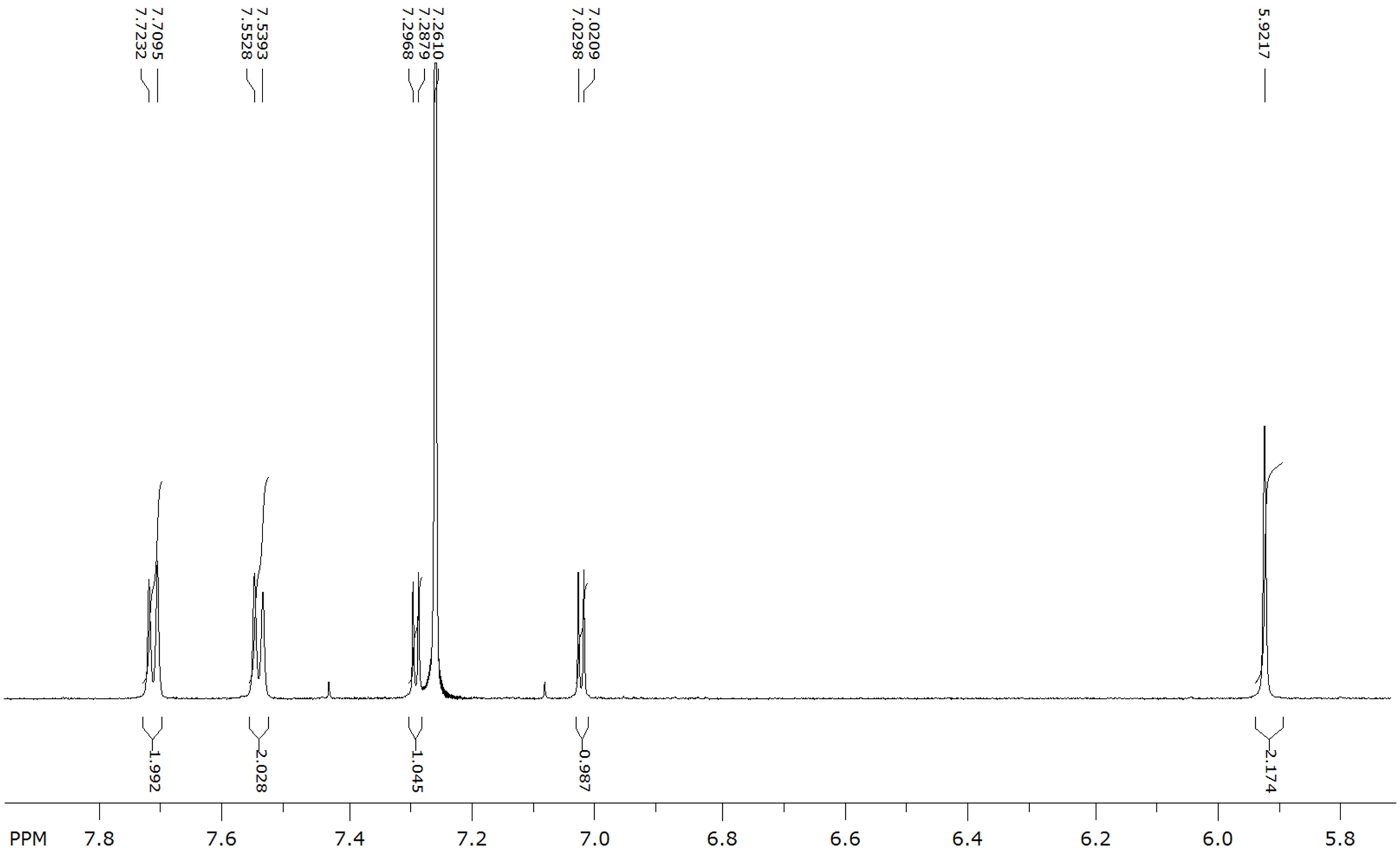

Figure S90. ^{1}H NMR ($CDCl_3$) spectrum of aromatic part of **50**.

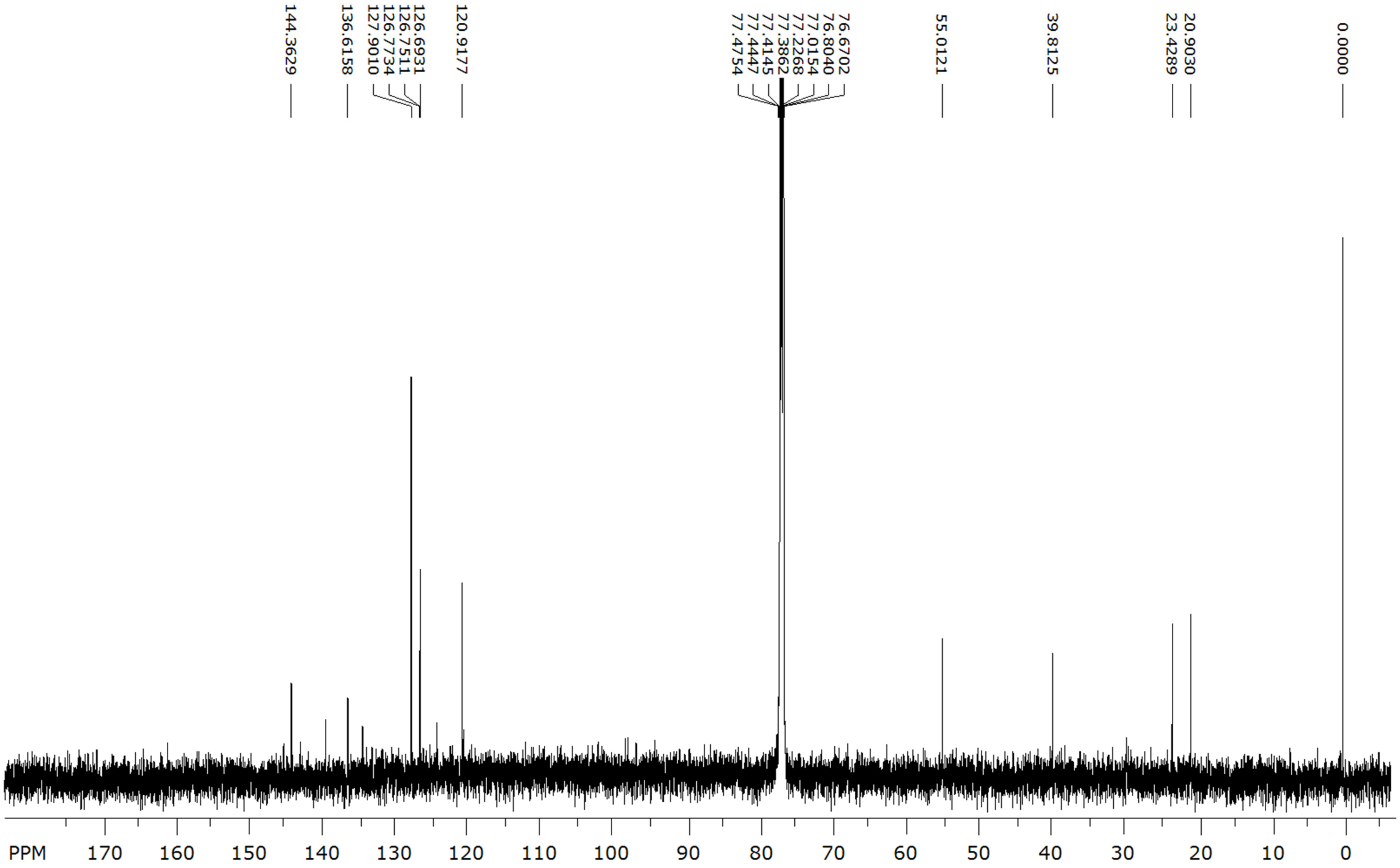

Figure S91. ^{13}C NMR ($CDCl_3$) spectrum of **50**.

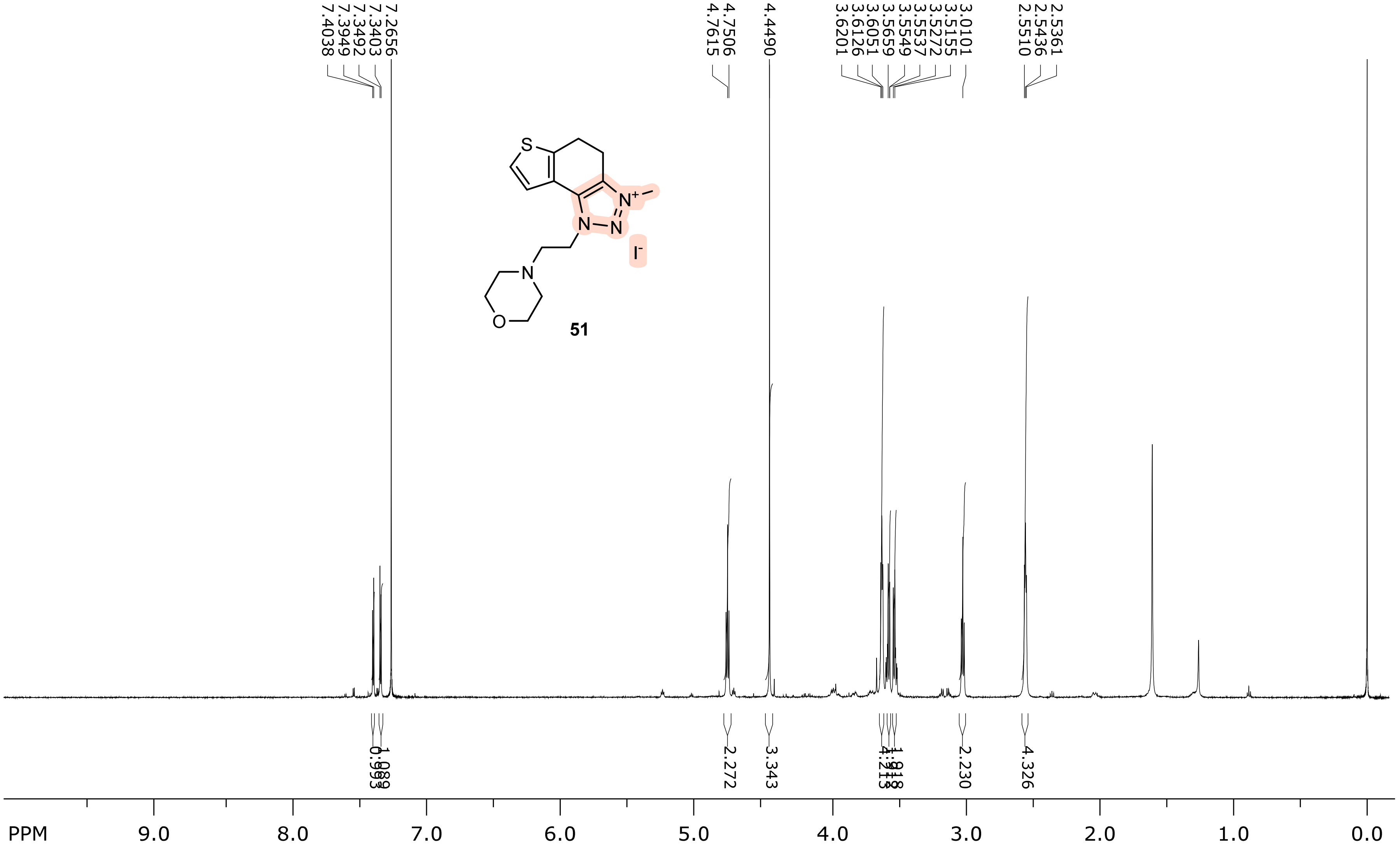

Figure S92. ^{1}H NMR ($CDCl_3$) spectrum of **51**.

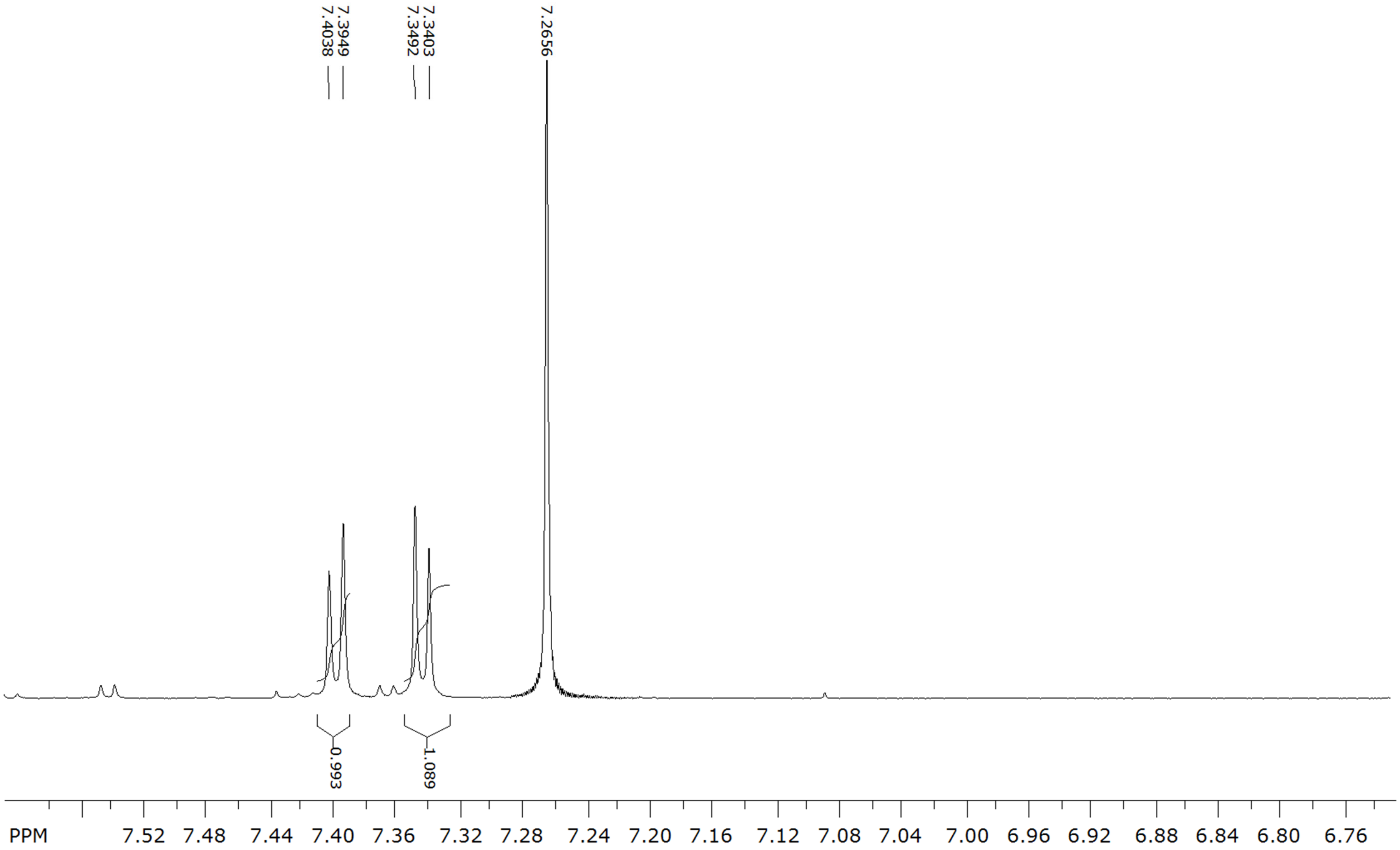

Figure S93. ^{1}H NMR ($CDCl_3$) spectrum of aromatic part of **51**.

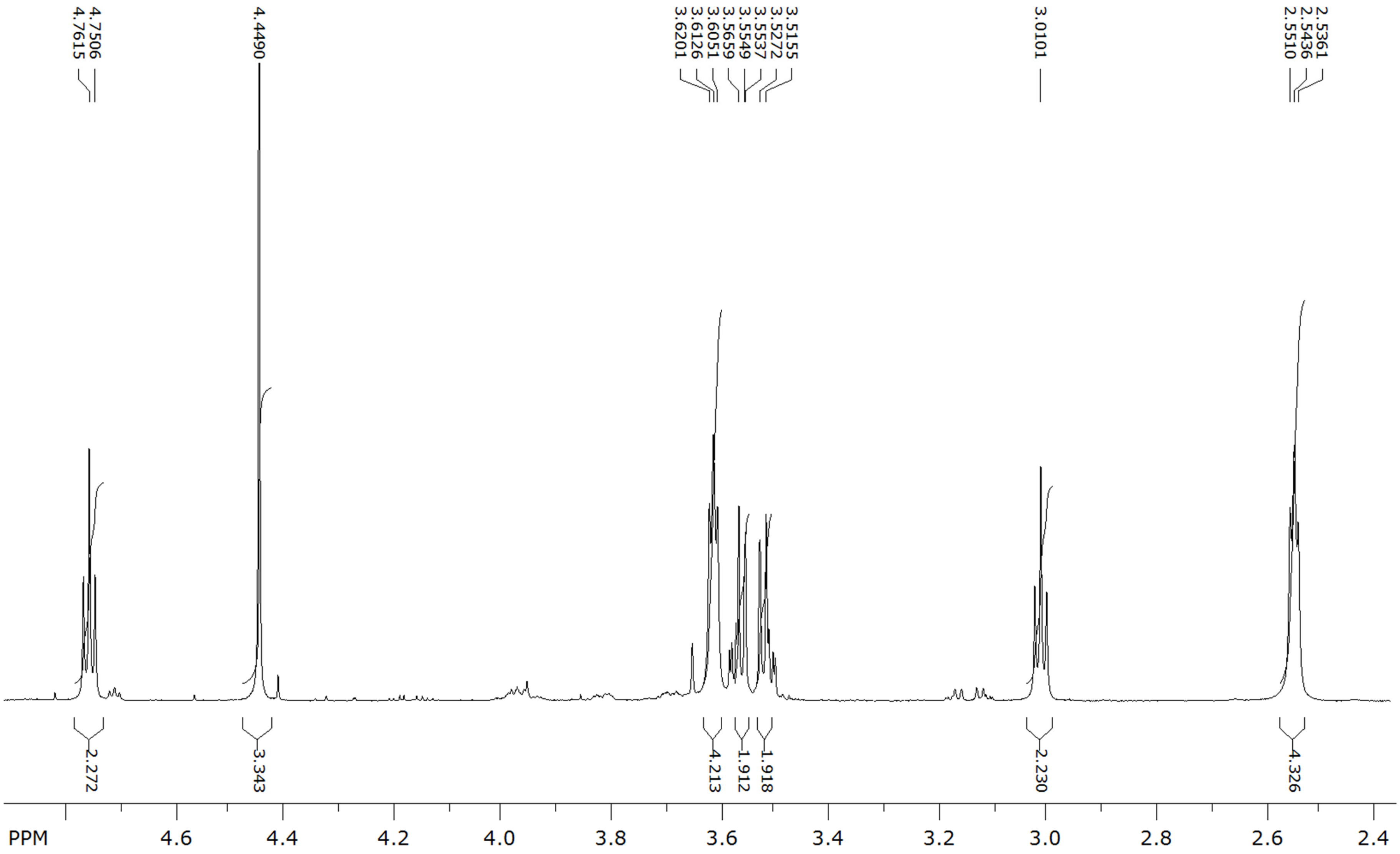

Figure S94. ^{1}H NMR ($CDCl_3$) spectrum of aliphatic part of **51**.

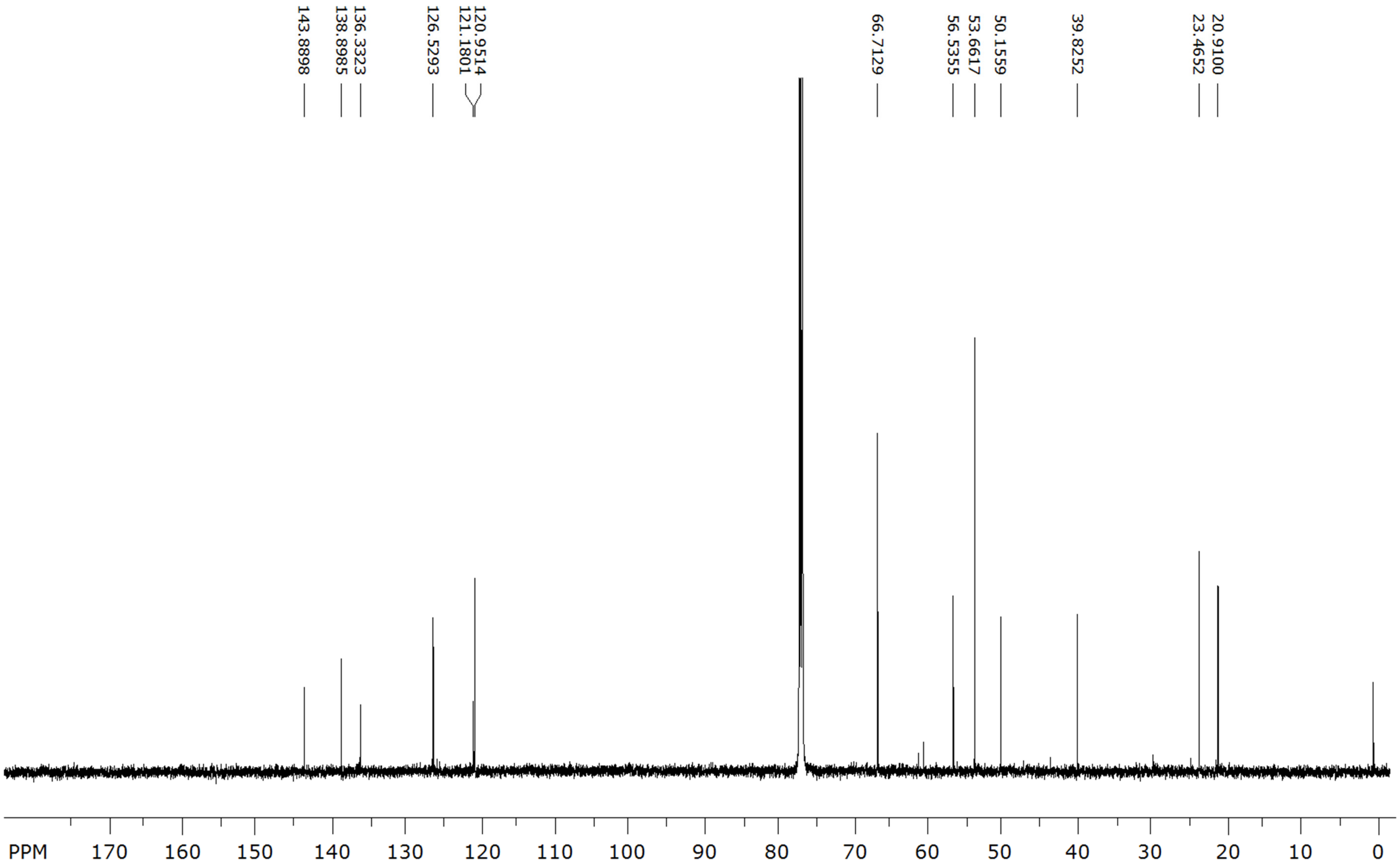

Figure S95. ^{13}C NMR ($CDCl_3$) spectrum of **51**.

2. Mass spectra and HRMS analyses

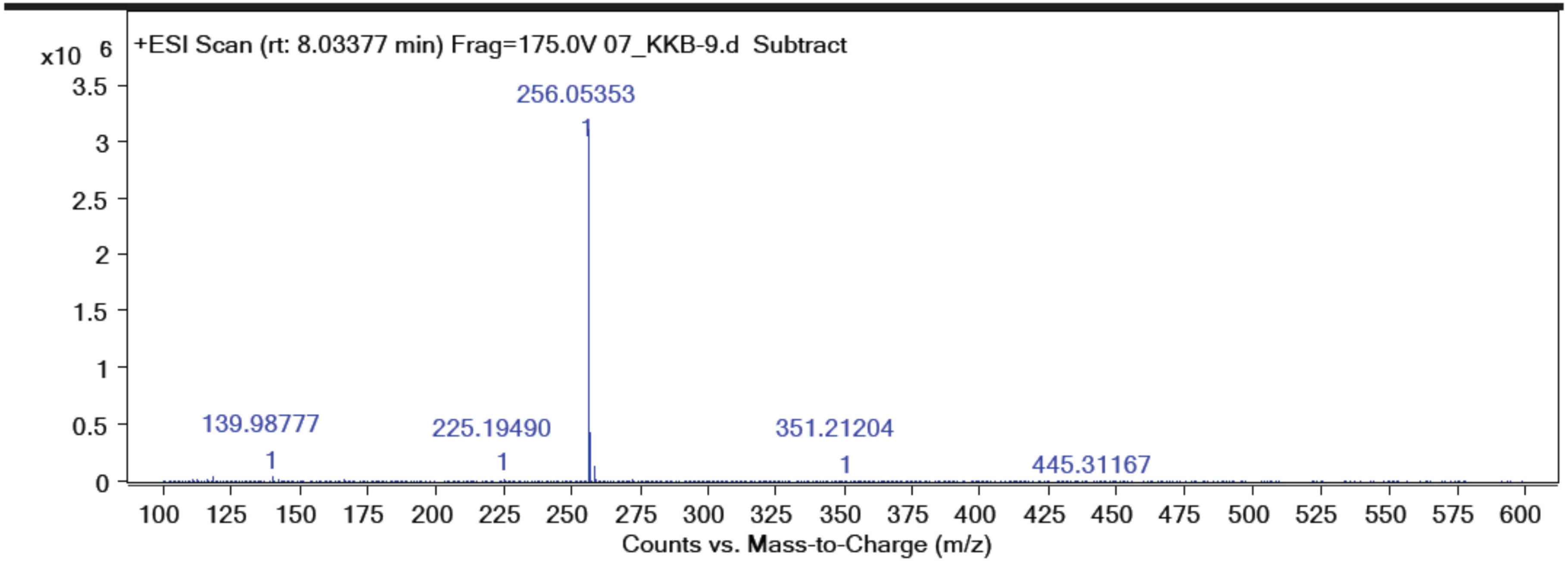

Formula Calculator Results

Formula	Best	Mass	Tgt Mass	Diff (ppm)	Ion Species	Score
C13 H9 N3 O S	True	255.04633	255.04663	1.2	C13 H10 N3 O S	96.98

Figure S96. Mass spectrum and HRMS analysis of compound **23**.

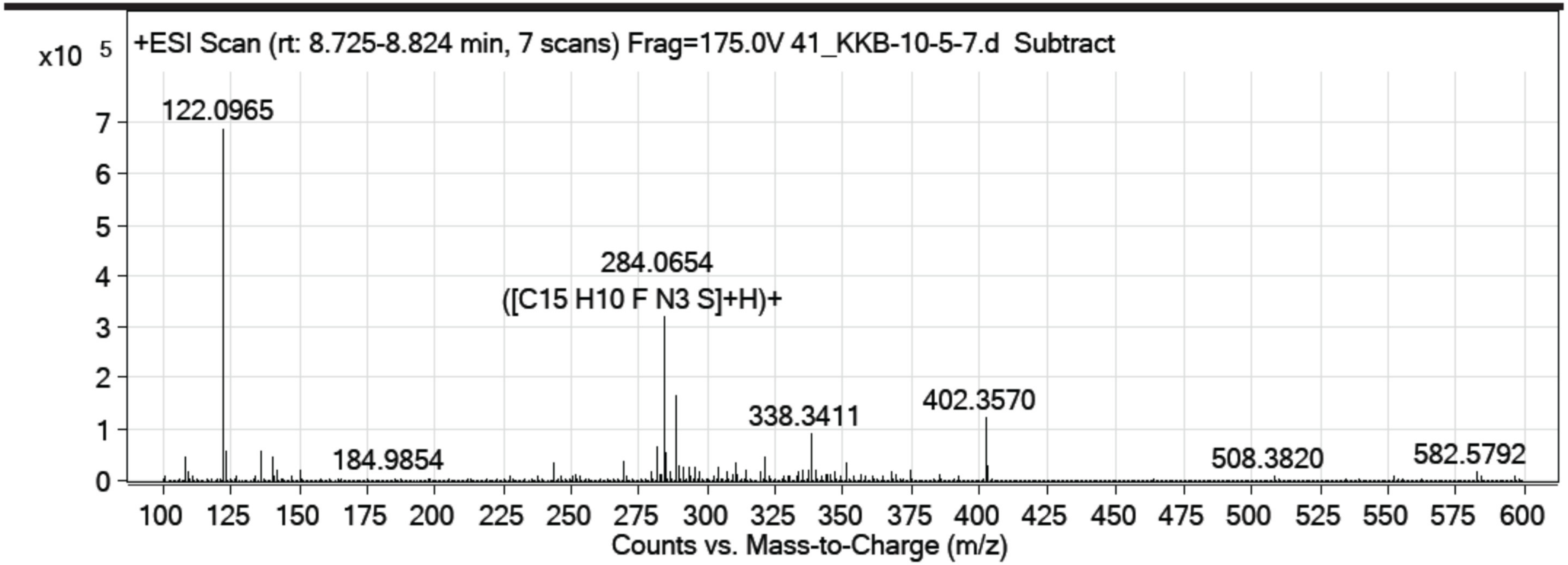

Formula Calculator Results

Formula	Best	Mass	Tgt Mass	Diff (ppm)	Ion Species	Score
C15 H10 F N3 S	True	283.0581	283.0579	-0.53	C15 H11 F N3 S	98.47

Figure S97. Mass spectrum and HRMS analysis of compound **24**.

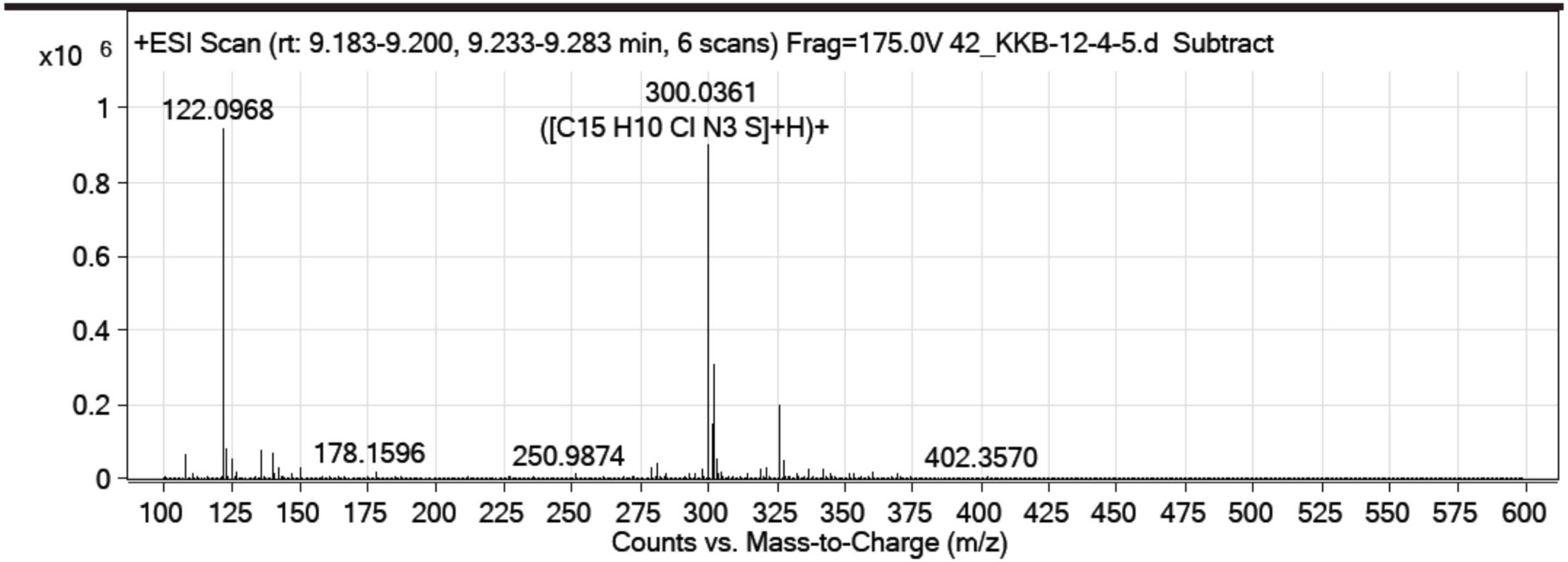

Formula Calculator Results

Formula	Best	Mass	Tgt Mass	Diff (ppm)	Ion Species	Score
C15 H10 Cl N3 S	True	299.0288	299.0284	-1.5	C15 H11 Cl N3 S	97.66

Figure S98. Mass spectrum and HRMS analysis of compound **26**.

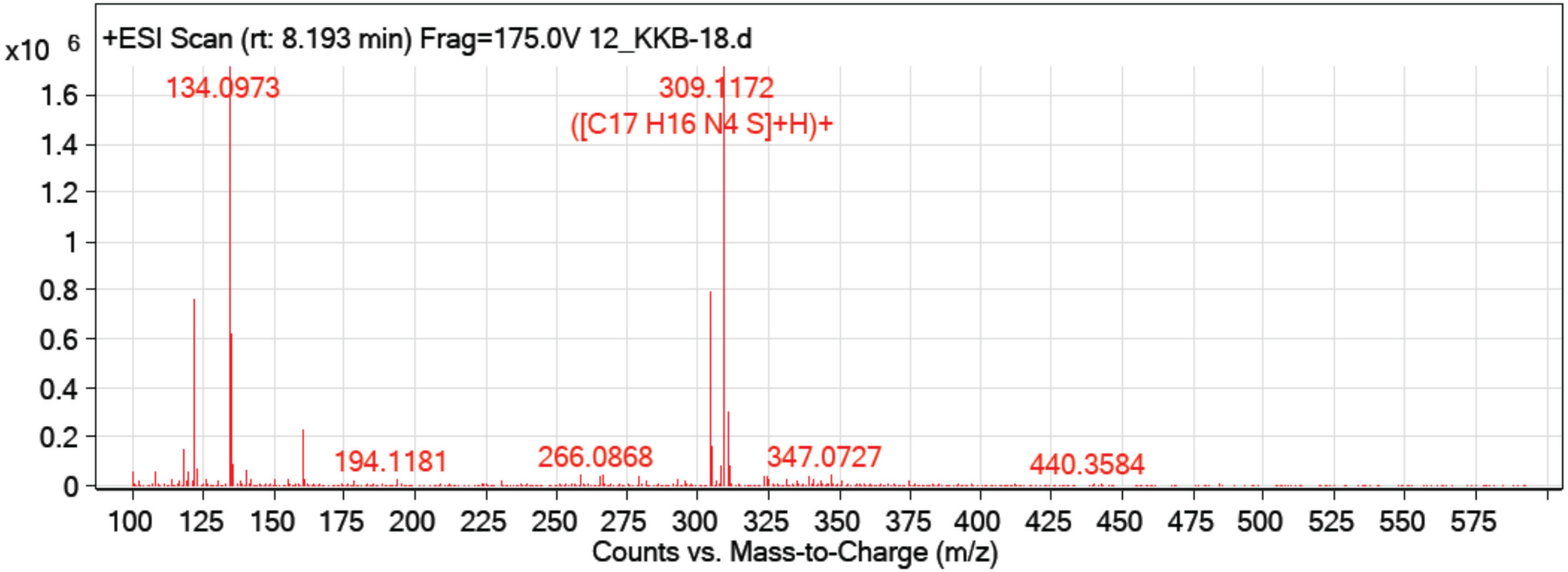

Formula Calculator Results

Formula	Best	Mass	Tgt Mass	Diff (ppm)	Ion Species	Score
C17 H16 N4 S	True	308.11	308.1096	-1.44	C17 H17 N4 S	96.35

Figure S99. Mass spectrum and HRMS analysis of compound **27**.

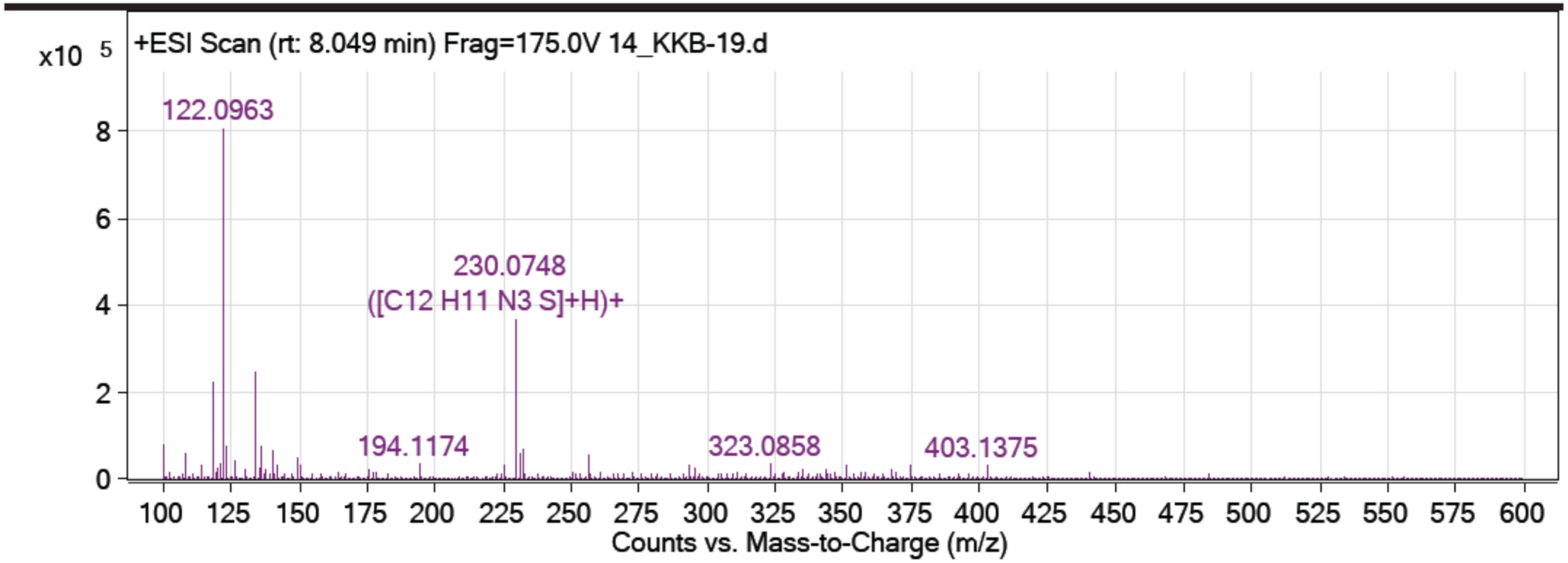

Formula Calculator Results

Formula	Best	Mass	Tgt Mass	Diff (ppm)	Ion Species	Score
C12 H11 N3 S	True	229.0676	229.0674	-0.87	C12 H12 N3 S	47.44

Figure S100. Mass spectrum and HRMS analysis of compound **28**.

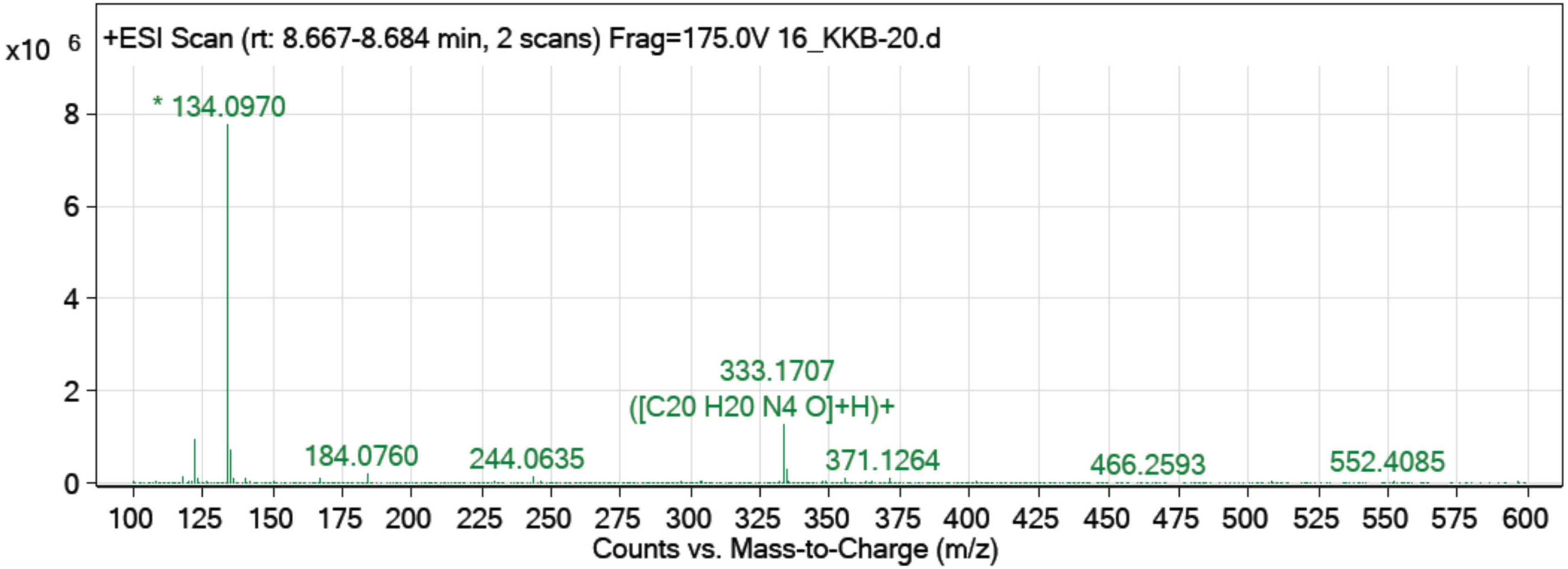

Formula Calculator Results

Formula	Best	Mass	Tgt Mass	Diff (ppm)	Ion Species	Score
C20 H20 N4 O	True	332.1635	332.1637	0.64	C20 H21 N4 O	98.1

Figure S101. Mass spectrum and HRMS analysis of compound **30**.

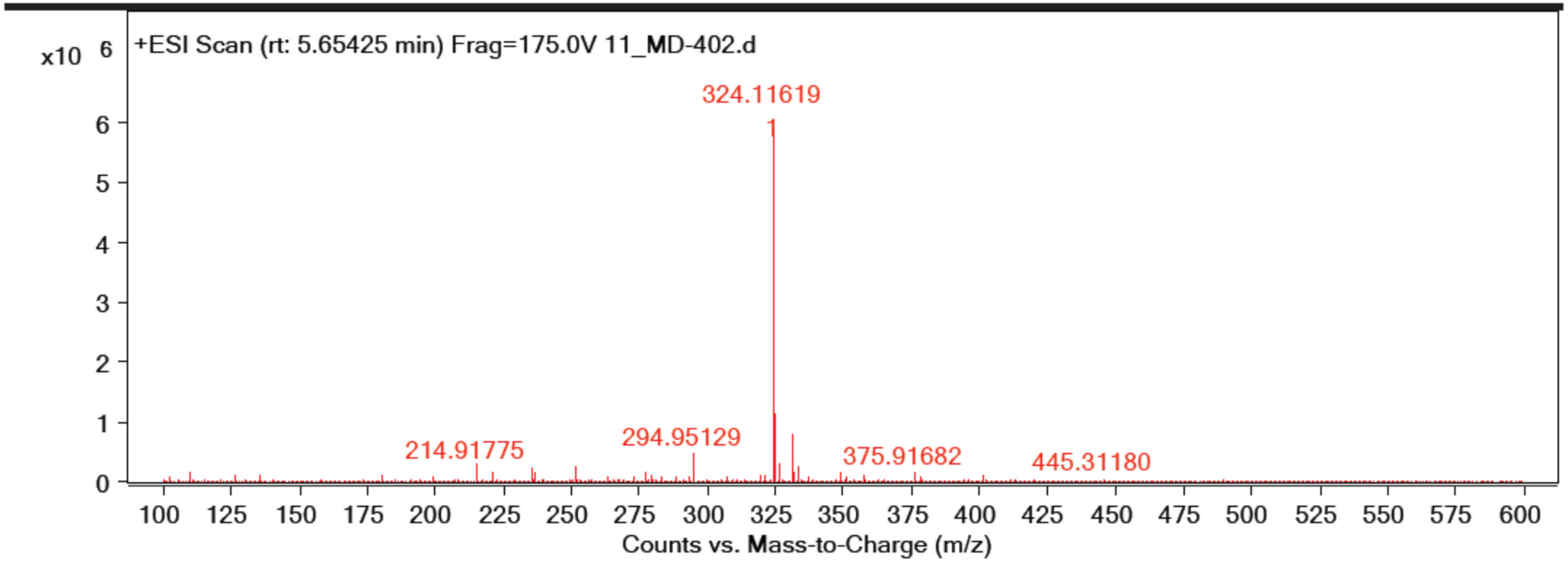

Formula Calculator Results

Formula	Best	Mass	Tgt Mass	Diff (ppm)	Ion Species	Score
C18 H17 N3 O S	True	323.10892	323.10923	0.97	C18 H18 N3 O S	97.78

Figure S102. Mass spectrum and HRMS analysis of compound **44**.

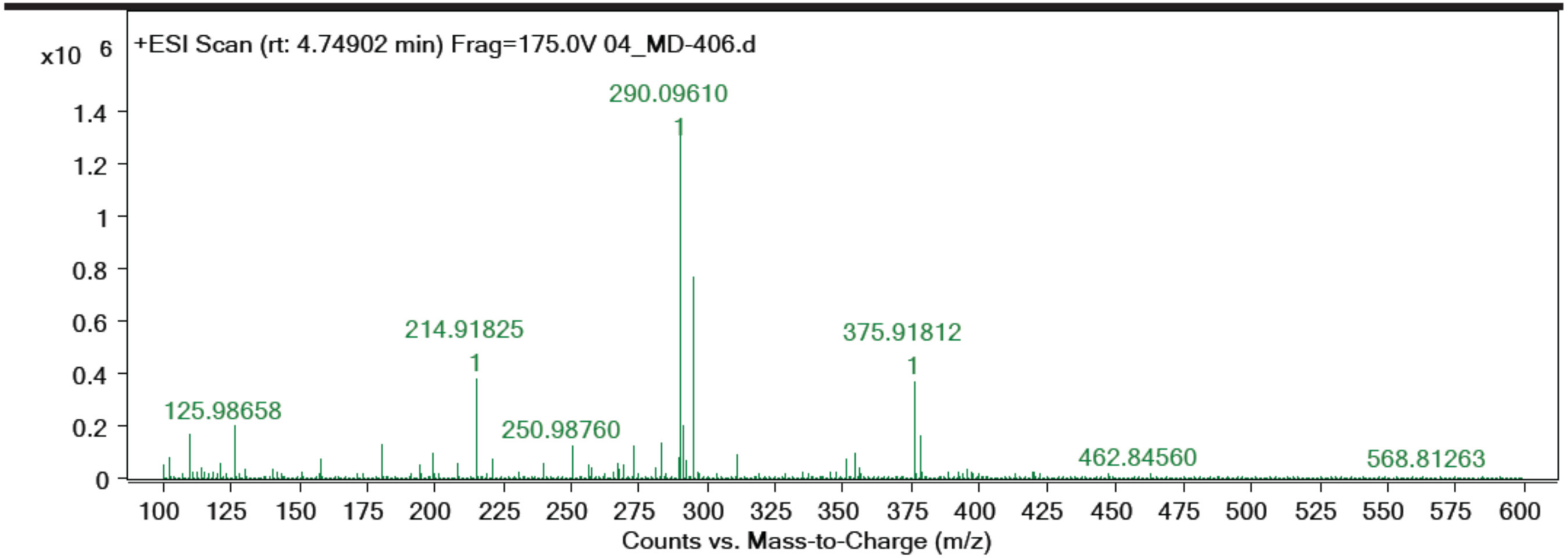

Formula Calculator Results

Formula	Best	Mass	Tgt Mass	Diff (ppm)	Ion Species	Score
C14 H15 N3 O2 S	True	289.08892	289.0885	-1.46	C14 H16 N3 O2 S	96.73

Figure S103. Mass spectrum and HRMS analysis of compound **45**.

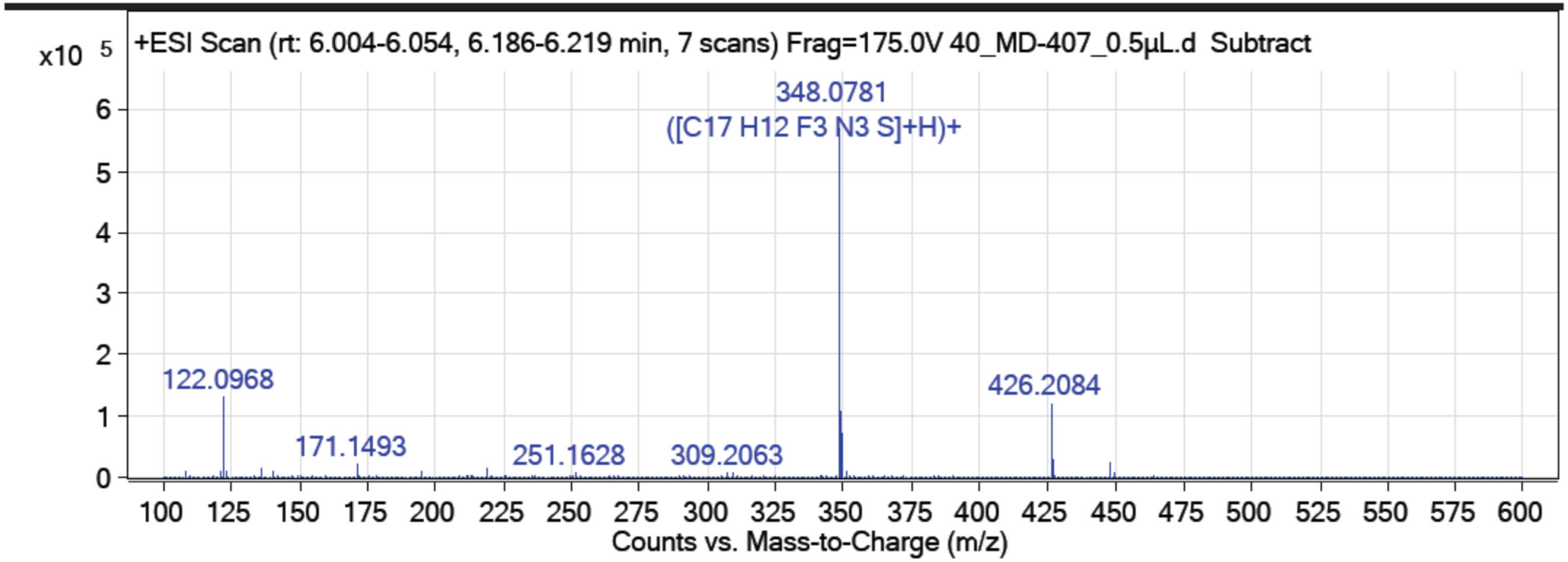

Formula Calculator Results

Formula	Best	Mass	Tgt Mass	Diff (ppm)	Ion Species	Score
C17 H12 F3 N3 S	True	347.0708	347.0704	-1.08	C17 H13 F3 N3 S	47.19

Figure S104. Mass spectrum and HRMS analysis of compound **46**.

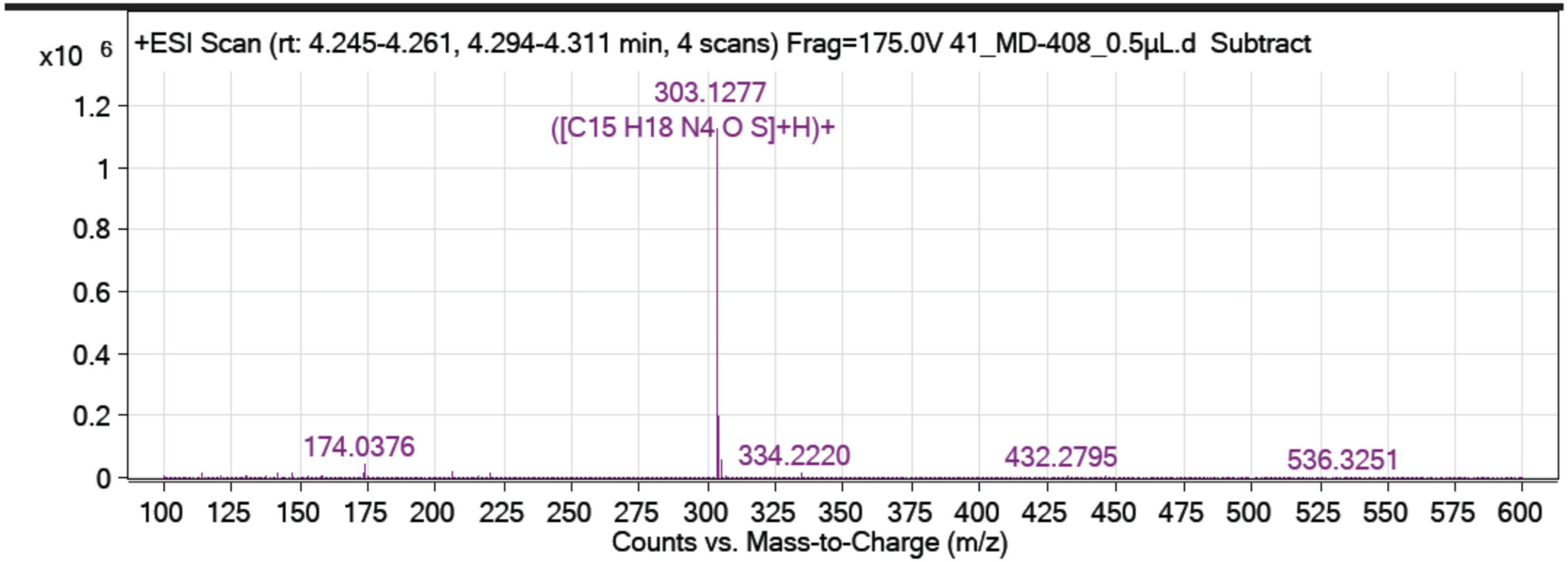

Formula Calculator Results

Formula	Best	Mass	Tgt Mass	Diff (ppm)	Ion Species	Score
C15 H18 N4 O S	True	302.1205	302.1201	-1.16	C15 H19 N4 O S	98

Figure S105. Mass spectrum and HRMS analysis of compound **47**.

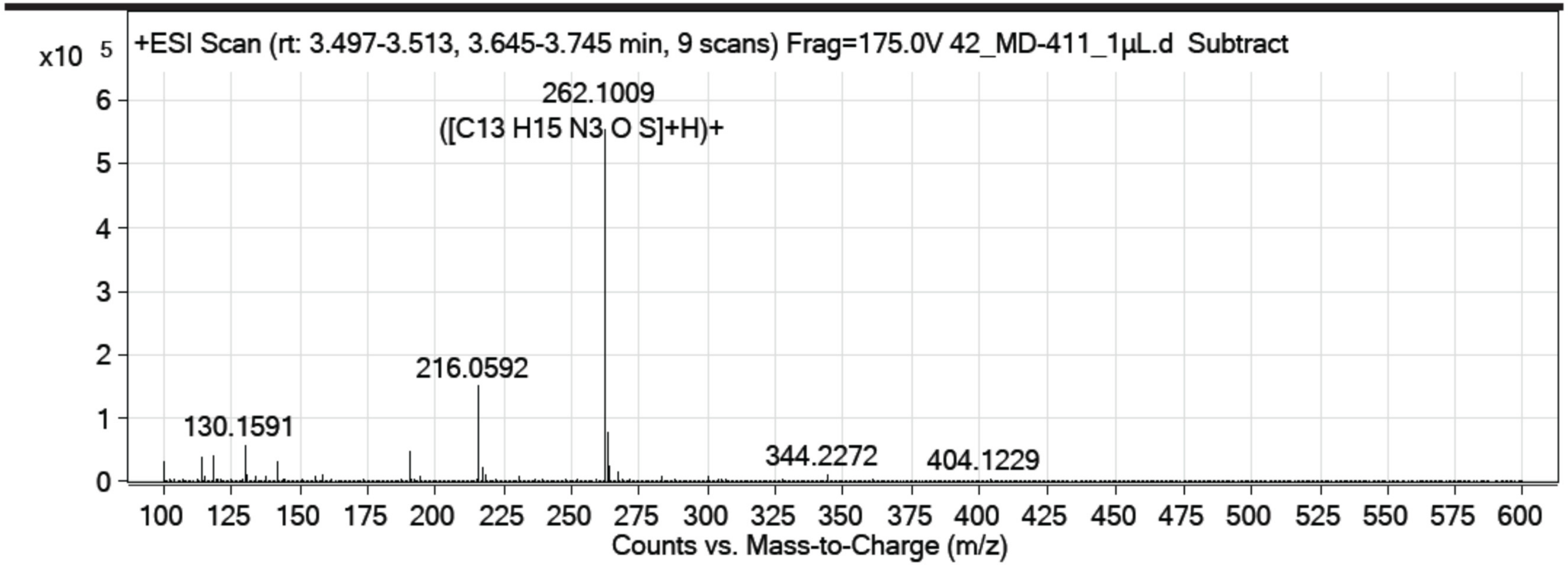

Formula Calculator Results

Formula	Best	Mass	Tgt Mass	Diff (ppm)	Ion Species	Score
C13 H15 N3 O S	True	261.0937	261.0936	-0.47	C13 H16 N3 O S	98.04

Figure S106. Mass spectrum and HRMS analysis of compound **48**.

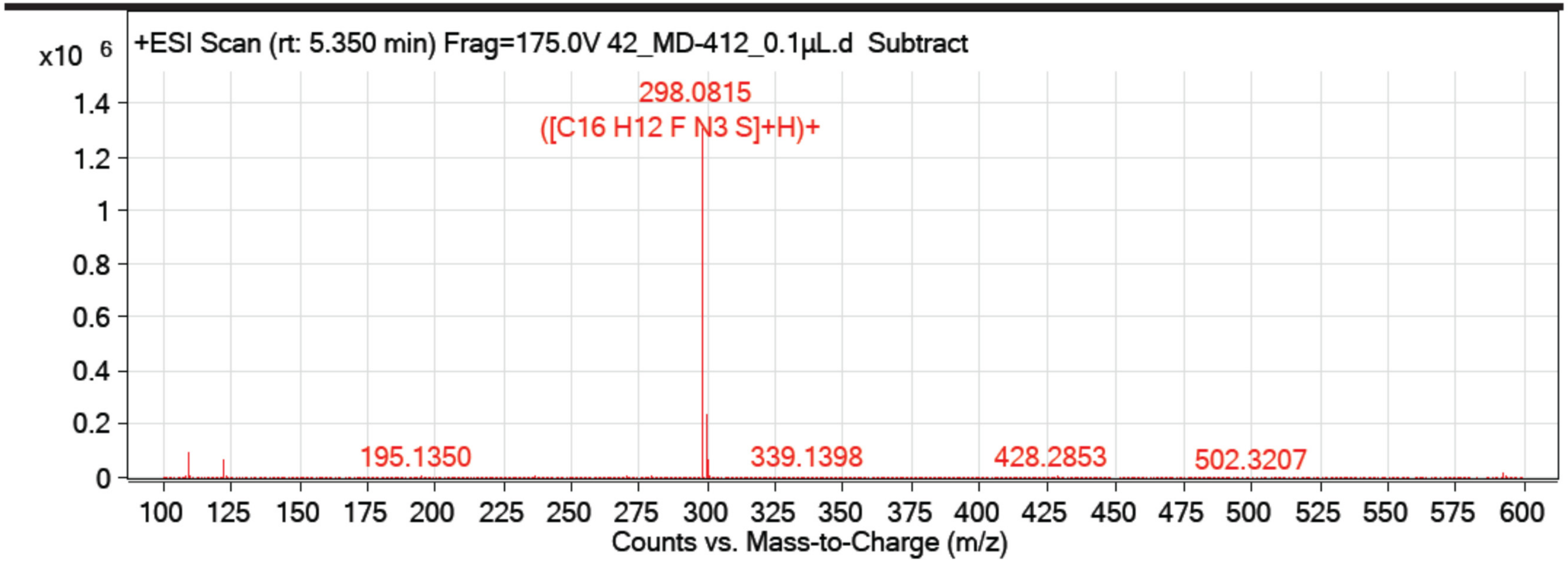

Formula Calculator Results

Formula	Best	Mass	Tgt Mass	Diff (ppm)	Ion Species	Score
C16 H12 F N3 S	True	297.0742	297.0736	-2.14	C16 H13 F N3 S	97.4

Figure S107. Mass spectrum and HRMS analysis of compound **49**.

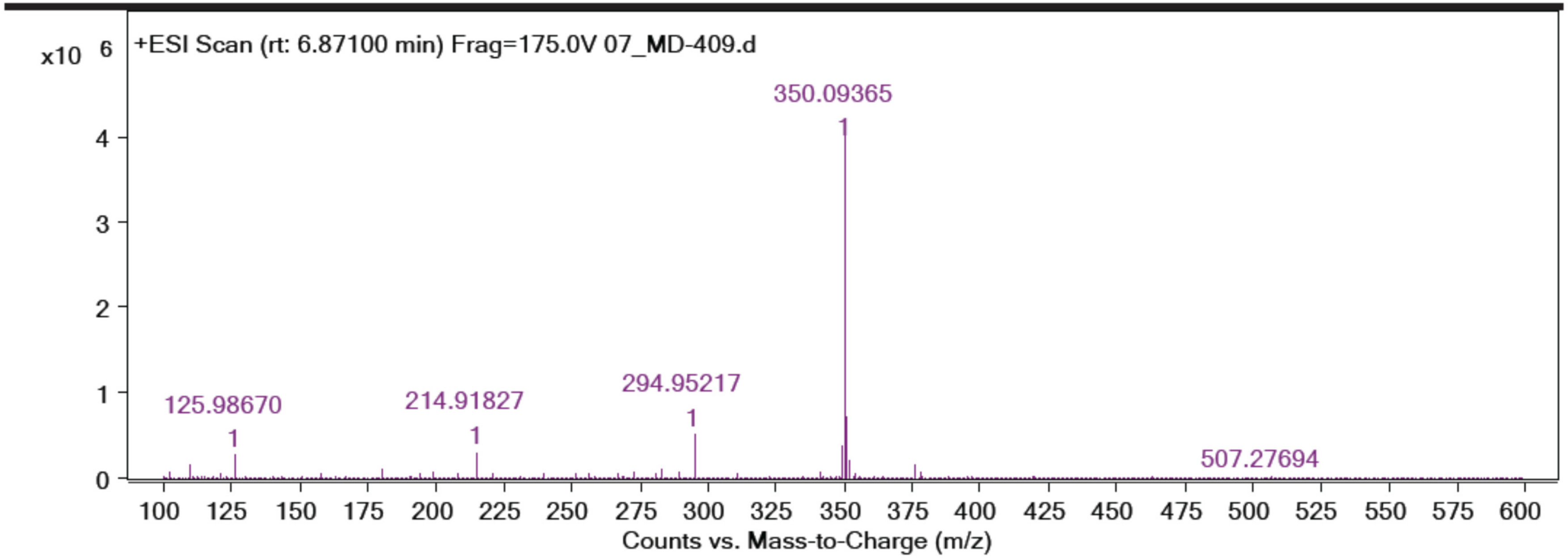

Formula Calculator Results

Formula	Best	Mass	Tgt Mass	Diff (ppm)	Ion Species	Score
C17 H14 F3 N3 S	True	349.08642	349.08605	-1.07	C17 H15 F3 N3 S	97.2

Figure S108. Mass spectrum and HRMS analysis of compound **50**.

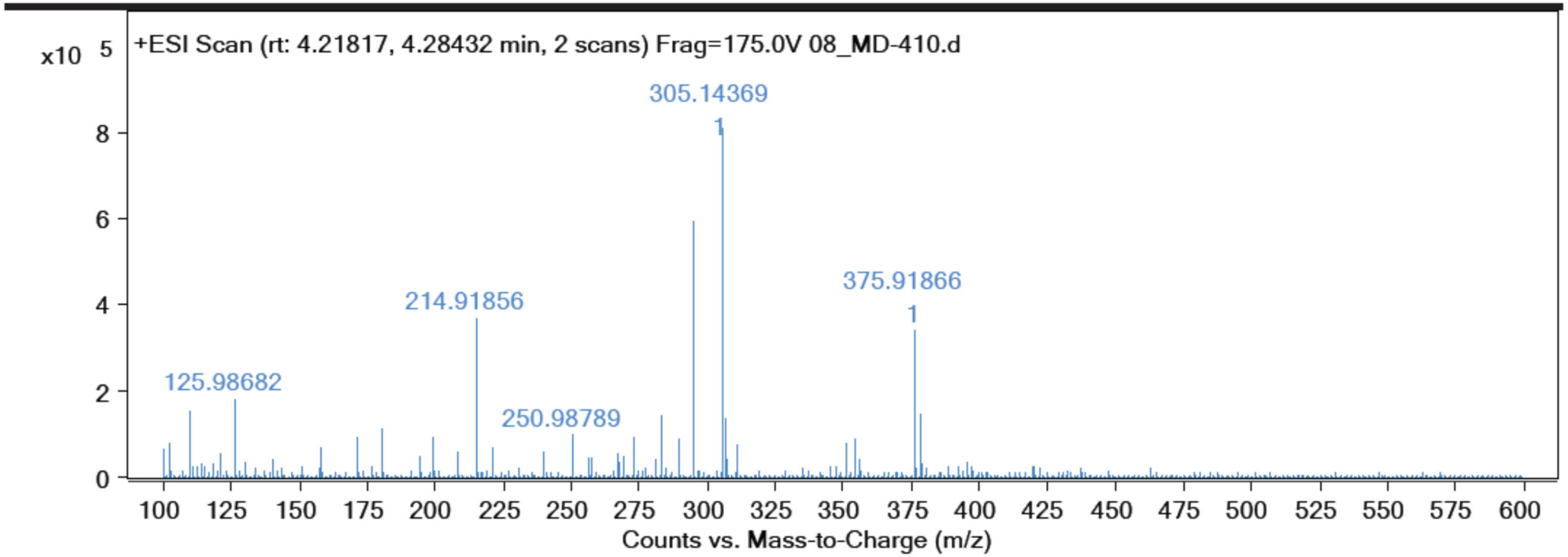

Formula Calculator Results

Formula	Best	Mass	Tgt Mass	Diff (ppm)	Ion Species	Score
C15 H20 N4 O S	True	304.13644	304.13578	-2.16	C15 H21 N4 O S	96.94

Figure S109. Mass spectrum and HRMS analysis of compound **51**.

3. Spectrophotometric data and DNA binding

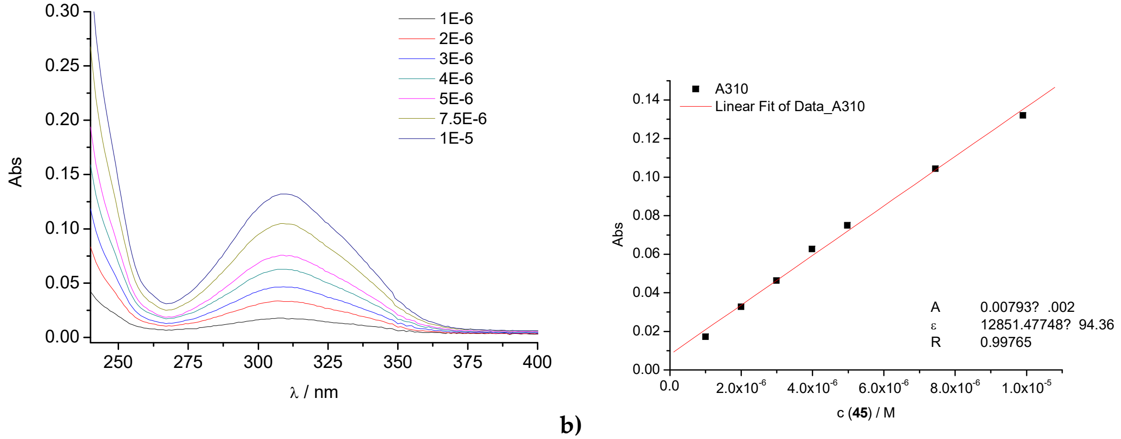

a) **b)**

Figure S110. a) The UV/vis spectra dependence of **3** on concentration, b) dependence of absorption at 310 nm on c(**45**) at 310 nm. Molar extinction coefficient (ε at 310 nm) is 12851.5 M^{-1} cm^{-1}.

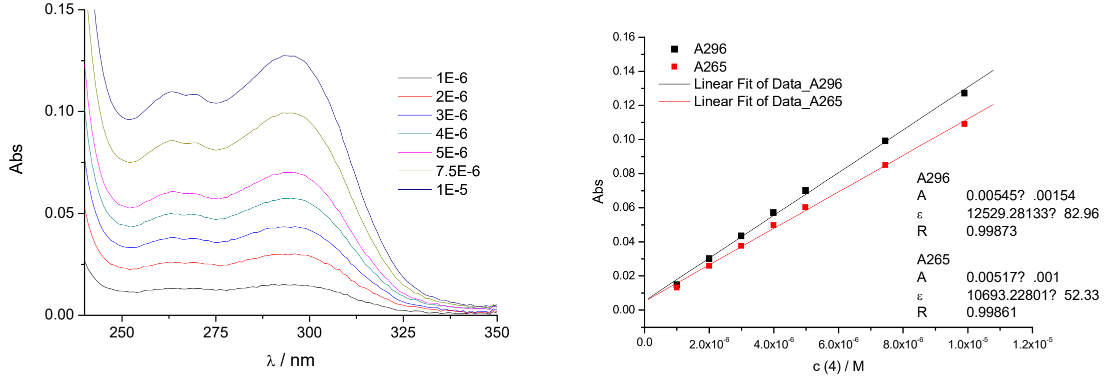

a) **b)**

Figure S111. a) The UV/vis spectra dependence of **4** on concentration. The sample shows two absorbance peaks, at 296 nm and at 265 nm, b) dependence of absorption at 296 nm on c(**50**) (black) and 265 nm (red). Molar extinction coefficient (ε) is 12529.3 M^{-1} cm^{-1} at 296 nm and 10693.2 M^{-1} cm^{-1} at 265 nm.

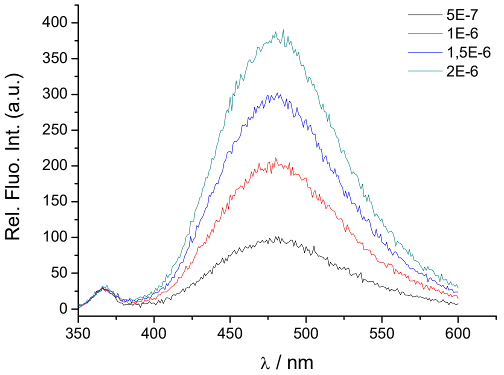

a)

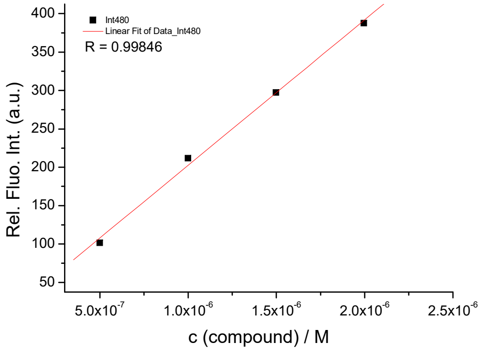

b)

Figure S112. a) fluorescence intensity dependence of **45** on concentration. The sample shows a fluorescence maximum at 480 nm, b) dependence of emission at 480 nm on c(**45**).

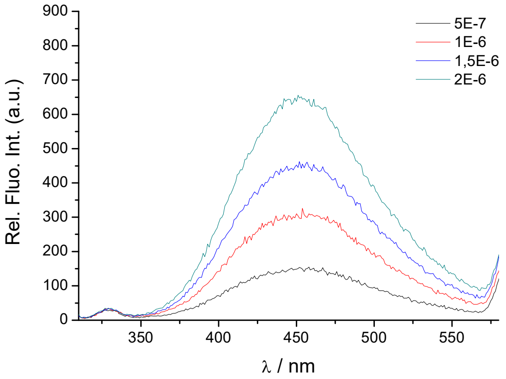

a)

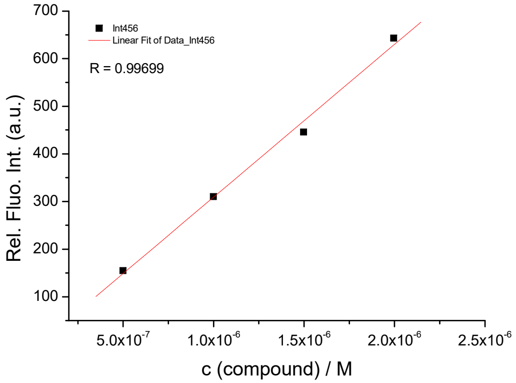

b)

Figure S113. a) fluorescence intensity dependence of **50** on concentration. The sample shows a fluorescence maximum at 456 nm, b) dependence of emission at 456 nm on c(**50**).

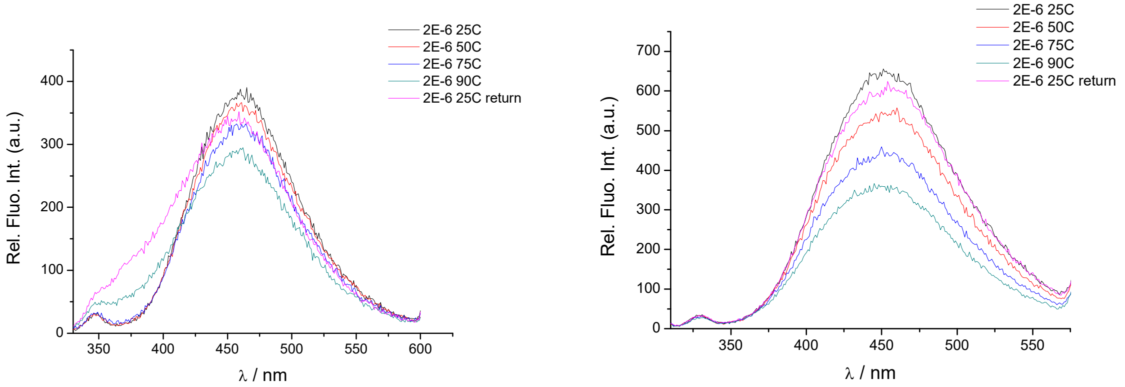

a) b)

Figure 114. a) temperature stability of **3** at 2×10^{-6} M. Decrease of fluorescence intensity upon heating and cooling is visible, along with broadening of the peak, b) temperature stability of **4** at 2×10^{-6} M. The sample shows a slight decrease in fluorescence intensity after heating.

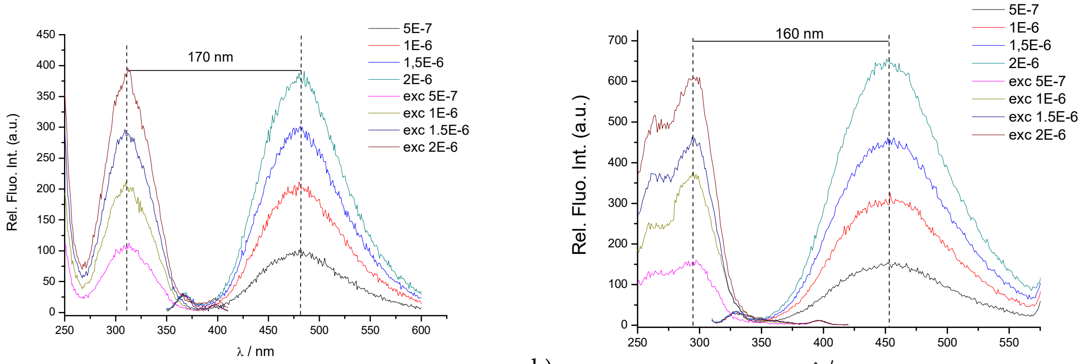

a) b)

Figure S115. a) emission and excitation (exc) spectra of **45** at increasing concentrations displaying Stokes shift of 170 nm, b) emission and excitation (exc) spectra of **50** at increasing concentrations displaying Stokes shift of 160 nm.

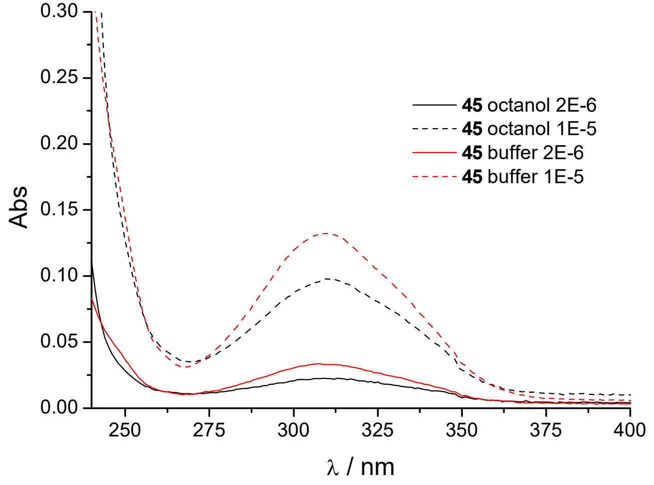

Figure S116. Comparison of UV/vis spectra of **45** at two concentrations (solid lines – $c = 2\times10^{-6}$ M, dashed lines – $c = 1\times10^{-5}$ M) in 1-octanol (black) and aqueous solution (red).

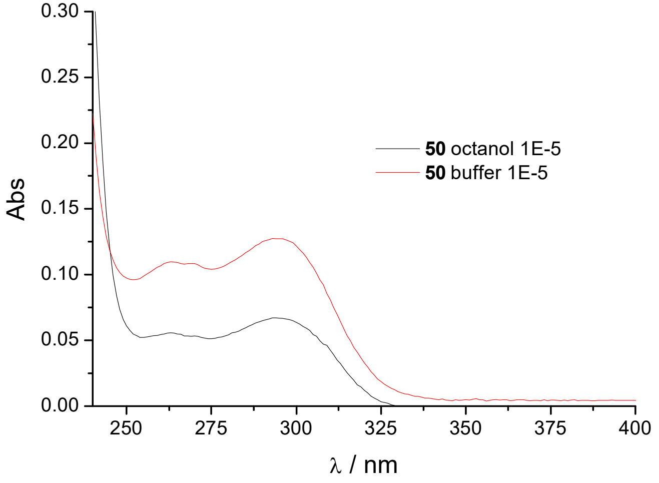

Figure S117. Comparison of UV/vis spectra of **50** at concentration $c = 10$ μM in 1-octanol (black) and aqueous solution (red).

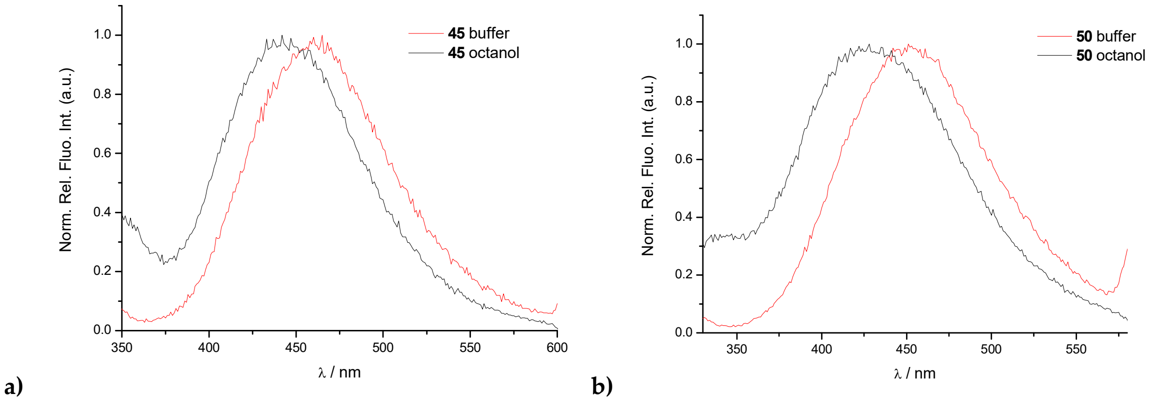

Figure S118. a) Comparison of normalised fluorescence spectra of **45 50** in 1-octanol (black) and aqueous solution (red), b) comparison of normalised fluorescence spectra of **50** in 1-octanol (black) and aqueous solution (red).

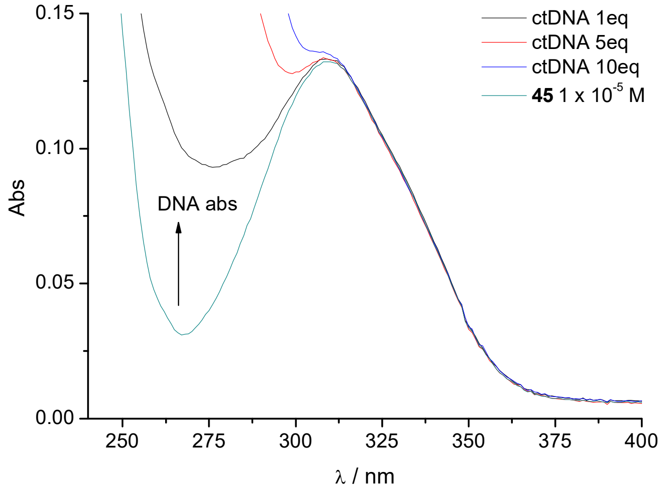

Figure S119. Difference in absorption for **45** ($c = 1{\times}10^{-5}$ M) before and after the addition of ctDNA (1eq = 1×10^{-5} M; 5eq = 5×10^{-5} M; 10eq = 1×10^{-4} M).

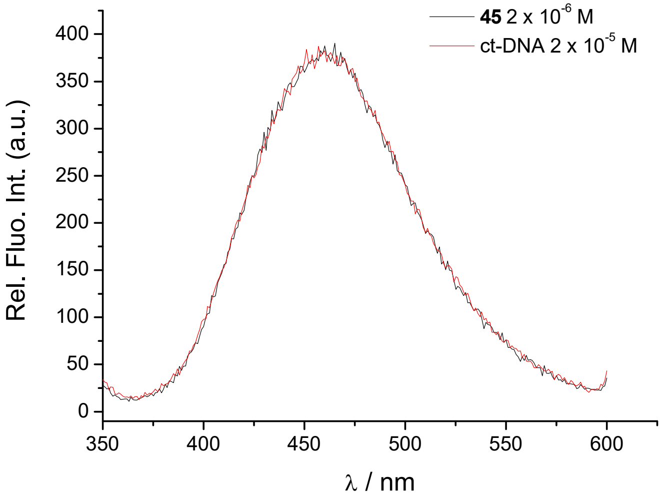

Figure S120. Impact of 10-fold *ct*-DNA addition on the fluorescence spectra for **45** (c = 2×10^{-6} M, λ_{exc} = 310 nm).

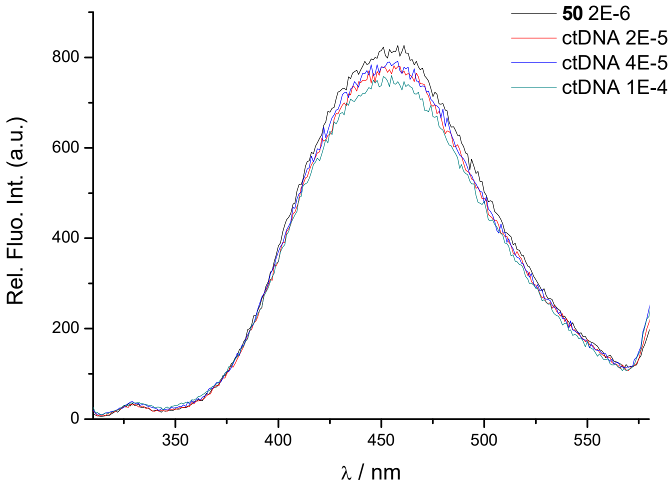

Figure S121. Impact of 10-, 20- and 50-fold *ct*-DNA addition on the fluorescence spectra for **50** (c = 2×10^{-6} M, λ_{exc} = 296 nm).

4. Cartesian coordinates of ligands docked into AChE and BChE

Table S1.
Ligand **44** docked into AChE

C	-10.331	-42.895	27.272
H	-10.996	-42.937	26.393
C	-10.570	-43.603	28.493
C	-9.562	-43.376	29.463
S	-8.345	-42.294	28.842
C	-9.182	-42.162	27.326
H	-8.819	-41.545	26.487
C	-9.562	-43.957	30.756
H	-8.740	-43.730	31.456
C	-10.578	-44.803	31.149
H	-10.595	-45.265	32.150
C	-11.594	-45.045	30.202
C	-11.603	-44.474	28.917
N	-12.730	-45.815	30.248
N	-13.415	-45.761	29.122
N	-12.743	-44.960	28.321
C	-13.224	-46.639	31.353
H	-12.645	-46.685	32.305
H	-13.379	-47.678	30.980
H	-14.270	-46.331	31.587
C	-13.245	-44.675	26.952
H	-12.525	-44.028	26.398
H	-13.199	-45.592	26.320
C	-14.653	-44.067	26.917
H	-15.445	-44.825	27.119
H	-14.846	-43.399	27.789
C	-14.862	-43.358	25.595
C	-14.490	-42.013	25.433
H	-14.019	-41.476	26.273
C	-14.705	-41.348	24.234
H	-14.405	-40.292	24.129
C	-15.304	-42.017	23.153

```
C    -15.679 -43.359  23.296
H    -16.146 -43.898  22.454
C    -15.457 -44.011  24.512
H    -15.759 -45.066  24.620
O    -15.466 -41.281  22.026
C    -16.719 -40.643  21.801
H    -16.852 -40.038  20.874
H    -16.965 -40.009  22.685
H    -17.528 -41.408  21.852
```

Ligand **45** docked into AChE

```
N   -12.903 -44.401  27.744
C   -11.701 -44.215  28.385
C   -11.759 -45.054  29.512
N   -12.988 -45.660  29.432
N   -13.671 -45.267  28.374
C   -10.565 -43.413  28.121
C    -9.526 -43.540  29.078
C    -9.593 -44.390  30.211
H    -8.741 -44.433  30.911
C   -10.710 -45.163  30.449
H   -10.779 -45.830  31.324
S    -8.180 -42.516  28.661
C    -9.004 -41.943  27.245
H    -8.568 -41.207  26.549
C   -10.244 -42.490  27.075
H   -10.916 -42.250  26.234
C   -13.580 -46.636  30.350
H   -13.003 -46.968  31.244
H   -13.881 -47.537  29.766
H   -14.570 -46.251  30.688
C   -13.355 -43.750  26.498
H   -12.484 -43.397  25.898
H   -13.838 -42.769  26.717
C   -14.272 -44.627  25.654
H   -13.955 -45.696  25.643
```

H	-15.275	-44.772	26.118
C	-14.353	-44.023	24.255
O	-14.701	-44.654	23.278
O	-13.996	-42.740	24.268
C	-15.081	-41.762	24.279
H	-15.949	-42.082	24.901
H	-14.827	-40.838	24.848
C	-15.457	-41.484	22.838
H	-16.285	-40.737	22.846
H	-15.711	-42.408	22.269
H	-14.589	-41.164	22.216

Ligand **44** docked into BChE

C	3.806	-8.577	-9.248
H	4.682	-8.527	-8.579
C	2.805	-7.558	-9.334
C	1.787	-7.869	-10.271
S	2.094	-9.412	-11.021
C	3.547	-9.617	-10.092
H	4.198	-10.503	-10.176
C	0.680	-7.029	-10.545
H	-0.071	-7.343	-11.290
C	0.530	-5.824	-9.892
H	-0.325	-5.157	-10.092
C	1.529	-5.490	-8.954
C	2.632	-6.316	-8.676
N	1.700	-4.397	-8.141
N	2.792	-4.488	-7.406
N	3.354	-5.635	-7.723
C	0.845	-3.215	-8.018
H	0.984	-2.327	-7.358
H	-0.174	-3.604	-7.788
H	0.745	-2.808	-9.051
C	4.620	-6.057	-7.070
H	5.134	-6.837	-7.679

H	4.408	-6.648	-6.149
C	5.577	-4.898	-6.759
H	5.301	-4.363	-5.821
H	5.454	-4.047	-7.469
C	7.000	-5.416	-6.726
C	8.066	-4.571	-6.372
H	7.858	-3.524	-6.094
C	9.375	-5.030	-6.366
H	10.196	-4.349	-6.085
C	9.658	-6.361	-6.717
C	8.608	-7.218	-7.071
H	8.815	-8.266	-7.346
C	7.296	-6.737	-7.073
H	6.474	-7.416	-7.355
O	10.967	-6.712	-6.677
C	11.879	-5.870	-5.980
H	12.955	-6.159	-5.947
H	11.512	-5.725	-4.937
H	11.795	-4.834	-6.384

Ligand **45** docked into BChE

N	2.259	-7.245	-8.798
C	2.765	-6.082	-8.266
C	4.083	-6.399	-7.894
N	4.235	-7.719	-8.241
N	3.142	-8.222	-8.781
C	2.232	-4.787	-8.062
C	3.136	-3.873	-7.463
C	4.465	-4.201	-7.092
H	5.108	-3.432	-6.632
C	4.963	-5.470	-7.300
H	5.993	-5.744	-7.017
S	2.385	-2.314	-7.257
C	0.905	-2.892	-7.956
H	0.009	-2.261	-8.081
C	0.955	-4.202	-8.336

```
H     0.113  -4.744  -8.798
C     5.417  -8.569  -8.080
H     6.341  -8.144  -7.623
H     5.679  -9.006  -9.072
H     5.125  -9.480  -7.508
C     0.902  -7.456  -9.342
H     0.154  -6.842  -8.788
H     0.529  -8.475  -9.088
C     0.804  -7.203 -10.842
H     1.387  -6.310 -11.167
H    -0.205  -6.845 -11.153
C     1.226  -8.477 -11.567
O     2.030  -8.490 -12.478
O     0.599  -9.541 -11.067
C     1.417 -10.728 -10.830
H     0.973 -11.656 -11.261
H     2.362 -10.730 -11.422
C     1.654 -10.825  -9.336
H     2.278 -11.731  -9.155
H     0.709 -10.823  -8.744
H     2.098  -9.897  -8.905
```

Table S2. Free energies of binding, ΔG_{bind} obtained by molecular docking of ligands **44** and **45** into the active site of AChE (4EY7.pdb), along with the number of conformational clusters and distribution of conformations.

Ligand	ΔG_{bind}/kcal mol^{-1}		Number of distinctive conformational clusters	Distribution of conformations within clusters with $n > 1$ (n = cluster population)
	lowest	highest		
**44**	−9.47	−8.41	9	8, 2, 7, 3
**45**	−8.23	−7.11	11	3, 2, 2, 6, 2, 4, 2,
Galantamine	−10.10	−10.10	1	25

Table S3. Free energies of binding, ΔG_{bind} obtained by molecular docking of ligands **44** and **45** into the active site of BChE (3DJY.pdb), along with the number of conformational clusters and distribution of conformations.

Ligand	ΔG_{bind}/kcal mol^{-1}		Number of distinctive conformational clusters	Distribution of conformations within clusters with $n > 1$ (n = cluster population)
	lowest	highest		
**44**	−8.57	−8.38	3	15, 6, 4
**45**	−6.89	−6.06	10	10, 2, 6
Galantamine	−7.31	−7.28	1	25
